# Screening Privileged Alkyl Guanidinium Motifs under Host-Mimicking Conditions Reveals a Novel Antibiotic with an Unconventional Mode of Action

**DOI:** 10.1021/jacsau.4c00449

**Published:** 2024-07-16

**Authors:** Dominik Schum, Franziska A. V. Elsen, Stuart Ruddell, Kenji Schorpp, Howard Junca, Mathias Müsken, Shu-Yu Chen, Michaela K. Fiedler, Thomas Pickl, Dietmar H. Pieper, Kamyar Hadian, Martin Zacharias, Stephan A. Sieber

**Affiliations:** † TUM School of Natural Sciences, Department of Bioscience, Chair of Organic Chemistry II, Center for Functional Protein Assemblies (CPA), Technical University of Munich (TUM), Ernst-Otto-Fischer Str. 8, Garching 85748, Germany; ‡ Helmholtz Zentrum München, Research Unit Signaling and Translation, Ingolstädter Landstraße 1, Neuherberg, Munich 85764, Germany; § Helmholtz Centre for Infection Research, Microbial Interactions and Processes, Inhoffenstraße 7, 38124 Braunschweig, Germany; ∥ Helmholtz Centre for Infection Research, Central Facility for Microscopy, Inhoffenstraße 7, 38124 Braunschweig, Germany; ⊥ TUM School of Natural Sciences, Department of Bioscience, Theoretical Biophysics (T38), Center for Functional Protein Assemblies (CPA), Technical University of Munich (TUM), Ernst-Otto-Fischer Str. 8, Garching 85748, Germany; # TUM School of Natural Sciences, Department of Chemistry, Catalysis Research Center (CRC), Technical University of Munich (TUM), Ernst-Otto-Fischer Str. 1, Garching 85748, Germany

**Keywords:** antibiotic development, high-throughput screen, host-mimicking conditions, guanidinium compounds, proteomics, target identification, signal peptidase

## Abstract

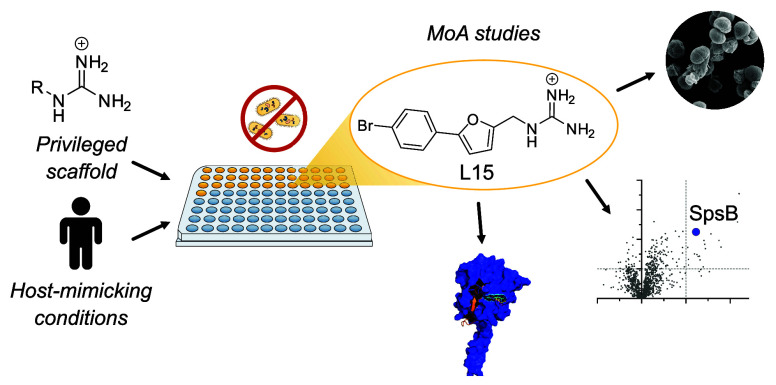

Screening large molecule libraries against pathogenic bacteria is often challenged by a low hit rate due to limited uptake, underrepresentation of antibiotic structural motifs, and assays that do not resemble the infection conditions. To address these limitations, we present a screen of a focused library of alkyl guanidinium compounds, a structural motif associated with antibiotic activity and enhanced uptake, under host-mimicking infection conditions against a panel of disease-associated bacteria. Several hit molecules were identified with activities against Gram-positive and Gram-negative bacteria, highlighting the fidelity of the general concept. We selected one compound (**L15**) for in-depth mode of action studies that exhibited bactericidal activity against methicillin-resistant *Staphylococcus aureus* USA300 with a minimum inhibitory concentration of 1.5 μM. Structure-activity relationship studies confirmed the necessity of the guanidinium motif for antibiotic activity. The mode of action was investigated using affinity-based protein profiling with an **L15** probe and identified the signal peptidase IB (SpsB) as the most promising hit. Validation by activity assays, binding site identification, docking, and molecular dynamics simulations demonstrated SpsB activation by **L15**, a recently described mechanism leading to the dysregulation of protein secretion and cell death. Overall, this study highlights the need for unconventional screening strategies to identify novel antibiotics.

## Introduction

The immediate threat posed by multiresistant pathogenic bacteria requires fast and efficient strategies for the discovery of novel antibiotics with unprecedented modes of action (MoAs).^1−3^ Historically, the majority of antimicrobial drugs that are in use today have been discovered in the 1960s and were largely based on natural products.^4^ With the decline of effective antibiotics over decades, new campaigns for their discovery were launched, however, with limited success.^5^ One reason is the restricted uptake of small molecules by bacteria, especially in Gram-negative strains, that possess two cell membranes representing an almost insurmountable permeability barrier.^6−9^ Thus, screening of large chemical libraries that were predominantly designed for applications in human cells often failed to reveal hits with sufficient activity.^10^ Their physical properties are often insufficient for uptake, e.g., due to limited hydrophilicity for the permeation through porin transporters.^11^ The eNTRy rules^12,13^ were recently postulated as guidelines for the enhanced uptake of small molecules in Gram-negative bacteria. In fact, introducing polar moieties such as primary amine^13^ or guanidinium groups^14^ enable the conversion of previously inactive drugs into potent antibiotics, overcoming the membrane barrier. Both primary amine and guanidinium groups are part of antibiotically active molecules, including trimethoprim and streptomycin.^15,16^ The guanidinium group is thus a privileged motif in medicinal chemistry due to its ability to bind targets via H-bonds to negatively charged groups.^16^ Importantly, it is positively charged at physiological pH, facilitating uptake into bacterial cells.^16^

In the past, standard antimicrobial susceptibility testing was performed in rich media, which enabled the optimal growth of bacteria under limited stress conditions.^17^ However, these conditions do not resemble the *in vivo* settings of bacterial infections in the host environment.^18^ In particular, the high level of sodium bicarbonate in human extracellular fluid has been overlooked as a potentiator of antibacterial activity until recently.^19^ Sodium bicarbonate is known to cause changes in the bacterial gene expression and structure, and further dissipates the transmembrane pH gradient, thereby affecting the proton motive force (PMF).^19,20^ Antibiotics that depend on the PMF for activity and/or uptake unleash their full potential solely under bicarbonate supplementation. Further studies suggest an inhibition of bacterial efflux pumps by sodium bicarbonate, thereby increasing the intracellular concentration of the compound.^20^ Thus, screens performed in the presence of bicarbonate may reveal previously unrecognized antibiotics and provide an alternative strategy for increasing the hit rate of molecular screens.

Considering recent findings, we here introduce a streamlined platform for fast and reliable identification of antibiotics with novel MoAs. This strategy is based on three pillars: the preselection of alkyl guanidinium compounds as privileged antibacterial scaffolds, the screen under host-mimicking conditions, and subsequent target identification by chemical proteomics. Applying this platform, we identified a potent antibiotic hit molecule (**L15**), gained insights into crucial structural motifs driving its antibiotic activity, and analyzed its respective MoA.

## Results and Discussion

### Screening of 246 Organic Guanidinium Compounds under Host-Mimicking Conditions Reveals Potent Antibiotic Hits

**Figure 1:**
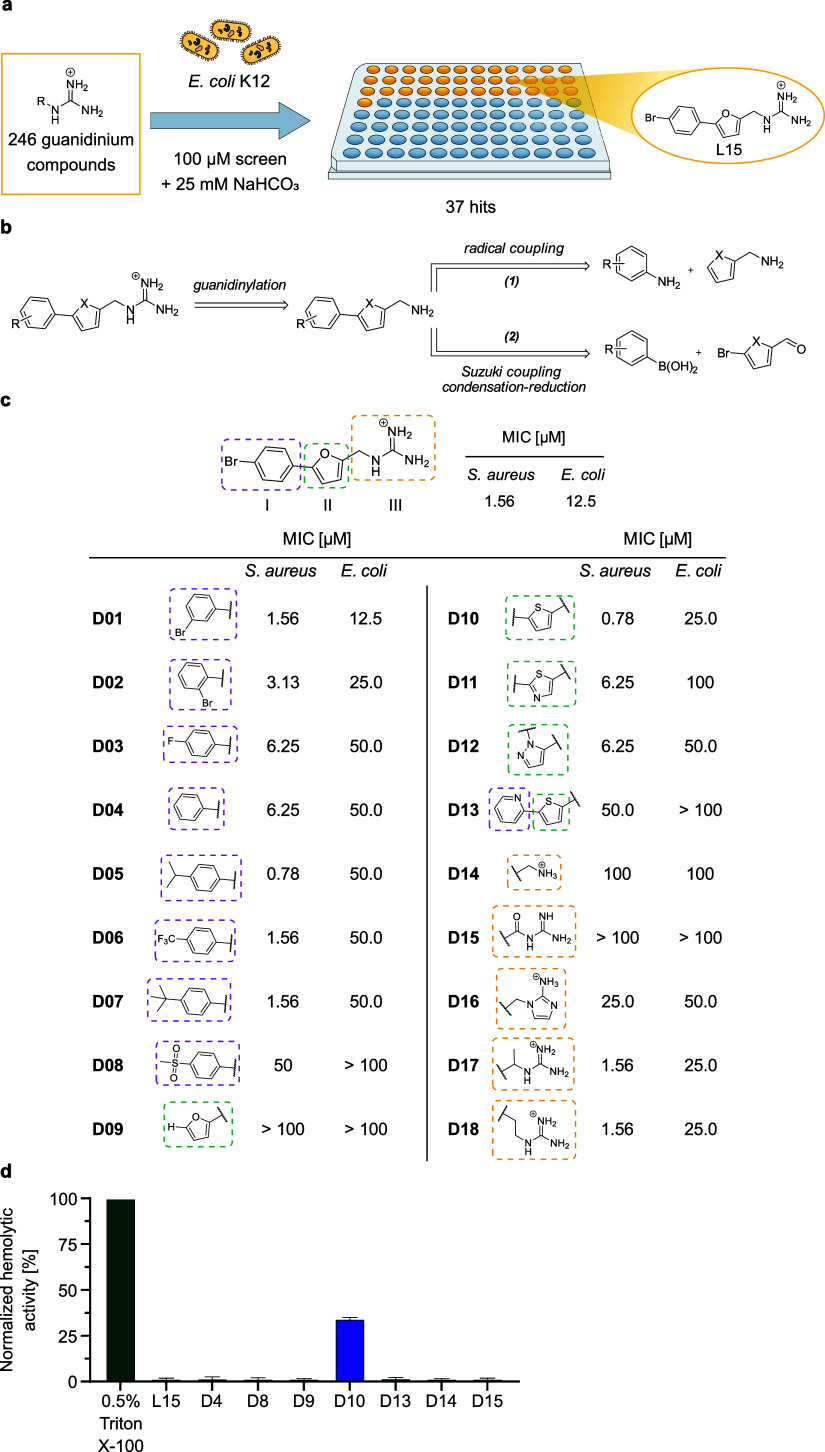
High-throughput screen (HTS) with a guanidinium library and structure-activity relationship (SAR) studies of **L15**. (a) Hit compound **L15** originates from a guanidinium-based HTS in *E. coli* K12 using sodium bicarbonate as an additive. (b) Two retrosynthetic routes to access the panel of **L15** derivatives are shown. X = O, S. (c) SAR studies of **L15** and derivatives assessing their antibiotic activity (MIC) in *S. aureus* USA300 Lac (JE2) and *E. coli* 536. The data represent average values of *n* = 3 biologically independent experiments per compound. (d) Hemolytic activity (%) of **L15** and derivatives in sheep blood measured at OD_540nm_ and normalized to 0.5% Triton X-100 as a positive control. The data represent mean values ± s.d. of averaged triplicates of *n* = 3 biologically independent experiments per compound.

By searching for commercially available organic guanidinium compounds, we compiled a library of 246 molecules with diverse chemical scaffolds. In a prescreen, the molecules were tested against *Escherichia coli* K12 at 100 μM concentration in the presence and absence of NaHCO_3_ (25 mM), resembling physiological host conditions.^19,20^ In the absence of NaHCO_3_, we were already able to identify six hit molecules suggesting that preselecting guanidinium molecules indeed enhanced the discovery rate. Importantly, adding bicarbonate increased the number of hit compounds to 37, demonstrating the need for mimicking host conditions to further enhance the potential of antibiotic libraries (Figure 1a). The four most potent compounds, **L15** (Figure 1a), **H03**, **J08**, and **L09** (Figure S1), exhibited minimum inhibitory concentrations (MICs) in *E. coli* K12 ranging from 12.5 to 50.0 μM (Table S1). We additionally tested these molecules against a panel of 12 Gram-positive and Gram-negative pathogens (Table S1). Compound **H03** displayed low micromolar activity (3.13–6.25 μM) against all tested Gram-positive strains and moderate activity (25.0–50.0 μM) against Gram-negative pathogens. However, we did not investigate this compound further due to its similar structure to a known FtsZ-targeting antimicrobial.^21^ Overall, compound **L15** stood out due to its excellent activity against *Staphylococcus aureus*, including methicillin-resistant *S. aureus* USA300 Lac (JE2), hereinafter referred to as *S. aureus*, with an MIC of 1.56 μM, which is in the range of antibiotically approved drugs against this strain.^22^

**L15**’s narrow activity spectrum indicates either limited uptake or a rather strain-specific mode of action (Table S1). The effect of bacterial uptake was thus investigated in membrane-deficient (ΔBamB) and efflux pump-deficient (ΔTolC) strains of *E. coli* BW25113 as well as in the presence of polymyxin B nonapeptide (PMBN) as a known membrane permeabilizer (Table S2).^23^ Interestingly, antibiotic activity was not increased in the mutant strains, indicating already sufficient target engagement. Moderately enhanced activity of **L15** by 2- or 4-fold was observed in the presence of PMBN, suggesting impaired uptake despite the guanidinium motif.

### Dissection of the L15 Scaffold Highlights Key Structural Motifs Driving Antibiotic Activity

To unravel structural hallmarks relevant for the antibacterial effect of **L15**, we systematically synthesized derivatives for SAR studies. For this, we divided the molecule into three major parts comprising the phenyl ring (I), the five-membered heterocycle (II) as well as the functional alkyl guanidinium moiety (III). The **L15** derivatives were mainly obtained via two distinct routes depending on the derivatized scaffolds (Figure 1b). In both cases, the guanidinium motif was introduced using the parent amines and 1*H*-pyrazole-1-carboximidamidehydrochloride as the guanidinylation reagent. To obtain the amines for the last synthesis step, the phenyl and heterocycle cores were coupled either in a one-step radical coupling (1) with furfurylamine and an aniline derivative or in a three-step coupling comprising a *Suzuki* cross-coupling followed by a condensation and a reduction step (2). In total, 18 derivatives (**D01**–**D18**) were synthesized and studied for their antibiotic activity in *S. aureus* and *E. coli* 536, hereinafter referred to as *E. coli*. Derivatives (**D12**, **D15**, and **D16**) were obtained via similar synthetic strategies.

Shifting the bromo substituent at the phenyl ring from *para* to the *meta* position (**D01**) had no effect on antibiotic activity (Figure 1c). However, the bromo substituent in *ortho* position (**D02**), a *para* substitution with fluoro (**D03**) or omitting the substituent (**D04**) led to a slight drop of MIC in *S. aureus* and *E. coli* (6.25 and 50 μM, respectively). Interestingly, diverse alkyl substituents in the *para* position, including isopropyl (**D05**), trifluoromethyl (**D06**), and *tert*-butyl (**D07**), slightly enhanced or retained biological activity allowing variability at this position. The addition of a methylsulfonyl substituent in *para* position (**D08**) or removal of the phenyl ring itself (**D09**) were not tolerated (50.0 to > 100 μM). While exchanging the furan ring for thiazole (**D11**) or pyrazole (**D12**) resulted in an activity drop activity (6.25 μM in *S. aureus* and >50.0 μM in *E. coli*), a thiophene ring (**D10**) led to an increase of antibiotic activity in *S. aureus* (0.78 μM) and a slight drop in *E. coli* (25.0 μM). However, an attempt to further enhance the molecular rigidity^22,23^ of the thiophene analog by the introduction of a neighboring pyridine substituent (**D13**) decreased activity against *S. aureus* (50.0 μM) and fully abolished antibiotic effects in *E. coli* (>100 μM).

The guanidinium motif was chosen in this study to enhance the uptake in Gram-negative bacteria and strengthen interactions with protein pockets via H-bonds.^14,16^ Based on the eNTRy rules, the introduction of primary amines is often sufficient to enable uptake into Gram-negative bacteria.^12^ Interestingly, the respective amine derivative of **L15** (**D14**) shows significantly lower antibiotic activity against *E. coli* and *S. aureus* (100 μM), suggesting that the guanidinium moiety not only facilitates uptake but is rather crucial for target binding. This was further corroborated by susceptibility testing of **D14** in the membrane-deficient *E. coli* ΔBamB strain which showed no increase in biological activity (100 μM). In addition, an acyl guanidine (**D15**) and a 2-aminoimidazole (**D16**) derivative with reduced basicity^24,25^ lacked any activity (>100 and 25.0–50.0 μM, respectively). Conversely, modifications of the guanidinium alkyl chain, including an adjacent methyl substituent (**D17**) or a methylene extension (**D18**), retained the initial potency (1.56 μM in *S. aureus*; 25.0 μM in *E. coli*).

All compounds were subsequently tested for toxicity against human HeLa cells via a proliferation assay. All tested derivatives showed IC_50_ values ranging from 1.2 to >100 μM and thereby indicating some general toxicity that correlates with their antibacterial activity (Table S3). However, since no hemolytic activity was observed for **L15** (Figure 1d), the observed cytotoxicity is most likely not caused by membrane disruption. In contrast, **D10** showed hemolytic activity (35% hemolysis at 100 μM) (Figure 1d), which indicates that only minor changes on the furan ring can cause adverse effects in human cells. Overall, **L15** showed the best activity profile making it a suitable candidate for further studies.

### L15 Is Bactericidal and Does Not Directly Act on the Bacterial Membrane

**Figure 2:**
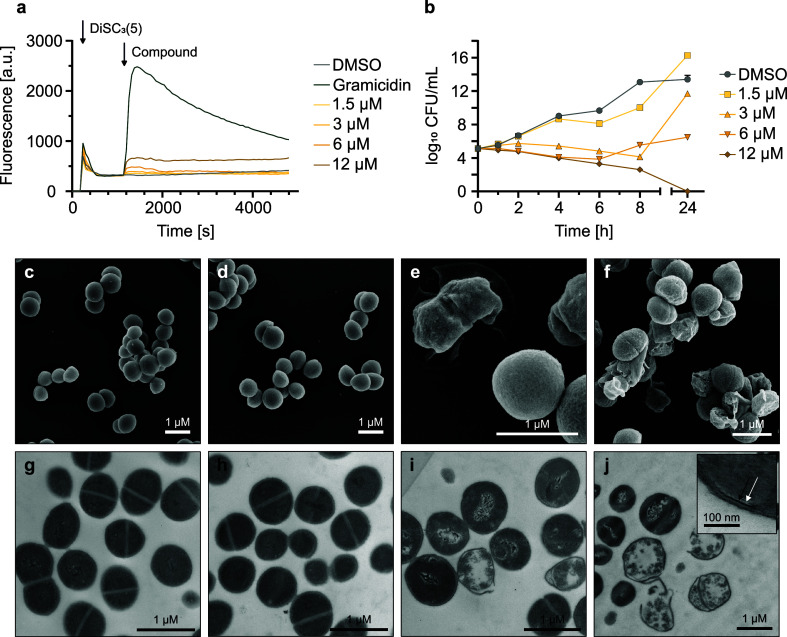
Biological profiling of **L15**. (a) Measured fluorescence intensity of the membrane potential-sensitive dye 3,3′-dipropylthiadicarbocyanine iodide [DiSC_3_(5)] in *S. aureus* USA300 Lac (JE2) cells treated with **L15**. Black arrows indicate addition of DiSC_3_(5) and **L15**, respectively. 1 μM gramicidin was used as a positive control. (b) Time-kill curves of *S. aureus* USA300 Lac (JE2) cells treated with different **L15** concentrations of *n* = 2 biologically independent experiments per concentration. (c–j) Scanning electron microscopy and transmission electron microscopy images of *S. aureus* USA300 Lac (JE2) cells treated with DMSO (c,g) or **L15** [3 μM (d,h), 12.5 μM (e,i), and 48 μM (f,j)]. Cells treated with DMSO (c) or 3 μM **L15** (d) show round and smooth coccal shape. Cell morphology changes at higher **L15** concentrations (e,f). Close-up window (j), the black arrow shows the intermediate layer, and the white arrow shows the electron transparent lipid layer of the cell membrane. The EM data represent *n* = 2 biologically independent experiments per condition. The scale bars represent 1 μm and 100 nm [detail (j)].

Based on the drastically more potent activity of **L15** against *S. aureus* compared to *E. coli*, we prioritized further in-depth MoA studies in this strain. First, membrane targeting properties were investigated by a membrane depolarization assay (Figure 2a).^26^ Among the tested compounds, only **L15**, **D04**, and **D10** showed membrane depolarization at higher concentrations (6 and 12 μM, Figure S2), with the strongest observed effect for **D10**. In contrast, **D13** and other derivatives (**D08**, **D09**, **D14**, and **D15**) lacking biological activity in *S. aureus* and HeLa cells showed no depolarization effect (Figure S2).

To gain detailed insights into the kinetics of antibiotic action, a time-kill assay was performed, revealing a bactericidal effect of **L15** (Figure 2b). In addition, the minimum bactericidal concentration (MBC) of **L15** (MBC = 3.13 μM) could be determined, with an MBC to MIC ratio of 2 (MBC/MIC ratios of ≤4 are considered bactericidal).^21,27^ Bacteria started to regrow after 24 h when treated with lower **L15** concentrations. We additionally performed frequency of resistance (FoR) experiments to evaluate **L15**’s potency to trigger resistance development which would be an undesired trait of a novel antibiotic. Satisfyingly, we obtained a FoR in the range of 2.3 × 10^–8^ to 2.8 × 10^–8^ at 6 μM **L15**, which indicates a range for low resistance development (Table S4).^28^ The reduced susceptibility of the generated mutants was further confirmed by enhanced MIC values (Table S5).

In the next step, we investigated the potential for morphological changes in *S. aureus* cells upon **L15** treatment by electron microscopy (EM) (Figure 2c–j). At a low compound concentration (3 μM, Figure 2d,h), no pronounced morphological changes were visible. Increasing **L15** concentrations resulted in a deformed cell shape (12.5 μM, Figure 2e,i), DNA rearrangement (Figure 2i,j), and even cell death with the release of cytoplasmic content (48 μM, Figure 2f,j). High compound concentrations also affected the lipid layer of the cytoplasmic membrane (Figure 2j, white arrow).

### Mechanism of Resistance Is Not Directly Linked to the Antibacterial Mode of Action

Genomic sequencing of the **L15**-resistant strains revealed a single conserved mutation in the multidrug efflux pump NorA,^29−33^ whereas no change was detected in any of the wildtype strain replicates. The mutation observed is a single nucleotide polymorphism altering C to T in position 755513 of the reference genome CP000255 leading to an amino acid change in position 366 from S to L in the protein NorA (UniProt ID: A0A0H2XGK0). This specific amino acid position is otherwise completely conserved in all *S. aureus* genomes reported so far. We thus further investigated the susceptibility of the NorA mutant strain in the presence of the fluoroquinolones **ciprofloxacin** and **norfloxacin**. Indeed, the MIC of both fluoroquinolones did not significantly change (2-fold increase), suggesting an insignificant effect of this **L15**-derived mutant on fluoroquinolone resistance (Table S6). In contrast, an *S. aureus* ΔnorA transposon strain [*S. aureus* ΔnorA USA300 Lac (JE2)]^34^ led to lower MIC values for **L15** (Table S6), demonstrating the overall relevance of this pump for reducing antibiotic drug concentrations.^35^ The unique mutation of NorA may thus selectively increase efflux of **L15**. Despite the discovery of NorA as a driver of resistance, no consistent gene mutations in putative targets were obtained that could provide a first glimpse into **L15**’s mode of action.

### Chemical Proteomics Unravels Essential Cellular Targets

**Figure 3:**
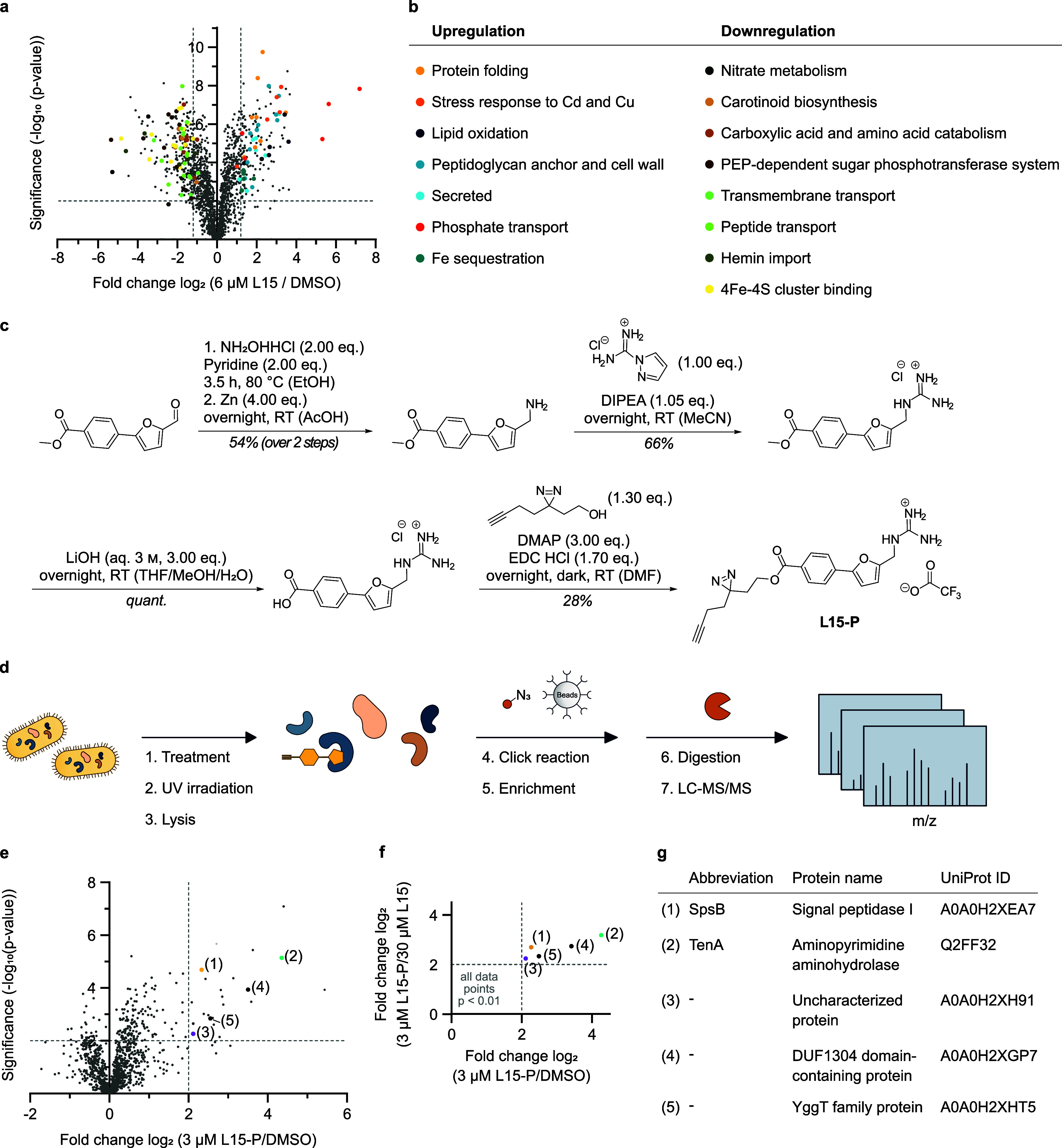
Target identification by chemical proteomics in *S. aureus* USA300 Lac (JE2) cells. (a) Volcano plot of *S. aureus* full proteome treated with 6 μM **L15** compared to DMSO. The vertical and horizontal dashed lines represent a log_2_-fold change ratio of 1.2 and a −log_10_*p*-value of 2, respectively. Colored dots show functional enriched proteins that were up- or downregulated. (b) Table of functionally enriched up- and downregulated proteins using STRING^37^ analysis. (c) Synthesis route of **L15-P**. (d) Schematic representation of an affinity-based protein profiling (A*f*BPP) approach for **L15** target identification. Intact cells were treated with **L15-P** or DMSO as control, following UV irradiation and cell lysis. The labeled proteins were clicked to biotin for enrichment on streptavidin beads, enzymatically digested and analyzed by mass spectrometry. (e,f) A*f*BPP experiments with **L15-P**. Volcano plot of *S. aureus* treated cells (3 μM **L15-P**) compared to DMSO (**e**). The vertical and horizontal dashed lines represent a log_2_-fold enrichment ratio of 2 and a −log_10_*p*-value of 2, respectively. (f) Scatter plot of **L15-P**-treated *S. aureus* cells vs DMSO compared to competitive A*f*BPP data (Figure S4). (g) Table of enriched proteins in both A*f*BPP and competitive A*f*BPP experiments, including two essential proteins [(1) and (3)] and three nonessential proteins [(2), (4), and (5)]. A two-sample students’ *t*-test, including permutation-based multiple testing correction (FDR = 0.05), was performed for all relevant comparisons to calculate the fold-change and statistical relevance. The data represent *n* = 4 biologically independent replicates. PEP = phosphoenolpyruvate, THF = tetrahydrofuran, DMF = *N,N*-dimethylformamide.

We further investigated proteomic changes in *S. aureus* upon **L15** treatment. Full proteome data showed a global response in protein up- and downregulation (Figure 3a,b). Many of the upregulated proteins were involved in overall stress response, including chaperones and the two-component system VraSR (Figure S6 and Table S8). In addition, proteins involved in phosphate import, iron sequestration, secretion, cell wall remodeling, lipid oxidation, and virulence, including staphyloferrin B, were upregulated (Figure 3b and Table S8). In contrast, downregulation was observed for proteins important for transport systems, nitrate, and carboxylic acid catabolism, staphyloxanthin biosynthesis, and iron-sulfur cluster binding (Table S8).

To directly decipher the protein targets of **L15**, we performed affinity-based protein profiling (A*f*BPP) with a corresponding **L15** photoprobe (**L15-P**). The photoprobe was equipped with a photo-crosslinking diazirine for covalent protein binding and an alkyne handle to subsequently attach functionalized azide tags via click chemistry for downstream analysis. Based on our SAR studies, we selected the *para* bromo position of the phenyl ring that tolerates functionalization to introduce a minimal photo-crosslinker. In brief, the synthesis of **L15-P** started with an aldehyde building block which was converted into the guanidinium analogously to synthesis route 2 (Figure 1b). Upon ester hydrolysis, the photoreactive warhead was introduced via *Steglich* esterification with the commercially available diazirine alcohol (Figure 3c).

The probe was tested for its antibiotic activity and retained biological activity with an MIC of 1.56 μM in *S. aureus*. In initial A*f*BPP studies, probe-treated *S. aureus* cells were UV irradiated, lyzed, and clicked to rhodamine azide to visualize target proteins by fluorescent SDS-PAGE. The experiment revealed concentration-dependent labeling of several bands that were successfully out-competed by a 10-fold excess of **L15** (Figure S4). For quantitative A*f*BPP studies, the probe-treated proteome was clicked to biotin azide and enriched on streptavidin beads. Tryptically digested peptides were analyzed by LC-MS/MS via label-free quantification in data-independent acquisition mode (Figure 3d). Proteins were identified as hits if they displayed a *p*-value of < 0.01 and log_2_-fold enrichment of > 2, as visualized in the corresponding volcano plot (Figure 3e). To account for the unspecific binding of the photo-crosslinker, we performed competitive A*f*BPP with a 10-fold excess of the parent compound **L15** (Figure S5). Only five proteins were enriched in both experiments (Figures 3e and S5), including two that have an essential and three that have a nonessential role in *S. aureus* viability (Figure 3f,g). Among those, aminopyrimidine aminohydrolase (Uniprot ID: Q2FF32) was highly enriched in both experiments and was thus tested for a putative role in the mode of action. However, its corresponding transposon mutant revealed no significant MIC shift in the presence of **L15**, excluding it as a relevant antibiotic target (Table S7). We thus shifted our attention to the two essential hits comprising an uncharacterized protein (UniProt ID: A0A0H2XH91) and the signal peptidase IB (SpsB, UniProt ID: A0A0H2XEA7).^36^ While the uncharacterized protein lacks any functional assignment, we prioritized target validation of SpsB.

### L15 Activates the Essential Protein SpsB

SpsB is a crucial enzyme in protein secretion that cleaves the substrate proteins’ signal peptide. The inhibition of SpsB is lethal, as shown by previous antibiotic inhibitors such as the arylomycins.^39−41^ We recently came across the first activator of SpsB, termed **PK150**, which enhances enzyme turnover up to threefold in an *in vitro* assay and results in an uncontrolled release of autolysins and subsequent cell lysis in *S. aureus* cells.^42^

**Figure 4:**
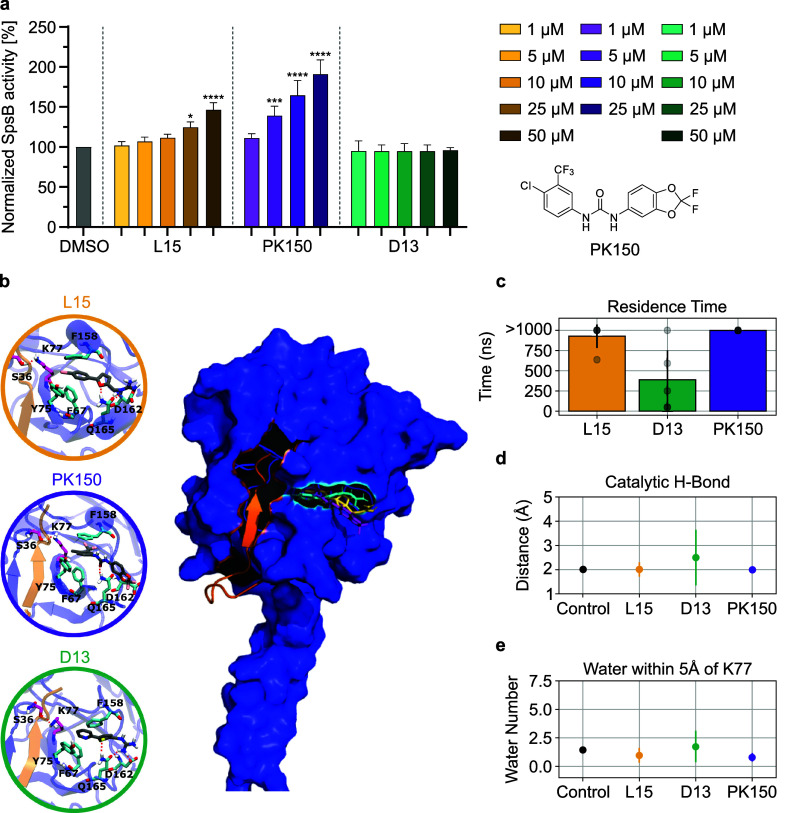
SpsB target validation by *in vitro* FRET assay and molecular dynamics (MD). (a) **L15**-, **D13**-, and **PK150**-induced concentration-dependent cleavage of FRET substrate by membrane-bound wildtype SpsB (50 μg mL^–1^ total membrane protein concentration). Membranes were extracted from *E. coli* BL21(DE3)pLysS cells harboring pET-55-DEST-SpsB. Substrate cleavage rates were normalized to DMSO-treated samples from the induced membranes. Background activity from noninduced membranes was subtracted before normalization. The data represent mean values ± s.d. of averaged triplicates of *n* = 3 biologically independent experiments per group. *p*-values were calculated with one-way ANOVA statistical testing for compound- vs DMSO-treated groups: *p*-value < 0.05 (*), < 0.01 (**), < 0.001 (***), and < 0.0001 (****). (b) Molecular docking of **L15**, **D13**, and **PK150** into the allosteric pocket of SpsB rendered from visiual molecular dynamics (VMD).^38^**PK150** showed high fluctuation during the simulation due to the lack of the second H-bond (D162). In this figure, one possible docking pose of **PK150** is depicted. The red dashed lines highlight the formed H-bonds crucial for binding (D162 and Q165) and catalytic activity (S36 and K77). (c) Calculated residence time of **L15**, **D13**, and **PK150** in the allosteric pocket of SpsB. Residence times are depicted with the mean (bar plot) and standard deviation to the mean (error bar) from *n* = 5 independently sampled residence times (transparent data points). (d) Change in the distance of the H-bond in the catalytic dyad (S36 and K77) due to compound binding. (e) Calculated number of water molecules in vicinity of K77. Filled circles and error bars in (d) and (e) show the average and standard deviation of the mean across *n* = 5 independent simulations.

Given the well-established and validated assay systems for determining *S. aureus* SpsB activation or inhibition, we tested different concentrations of **L15** in comparison to **D13**, a structurally similar but antibiotically inactive derivative and **PK150** as a positive control (Figure 4a). While **D13** had no effect on enzyme turnover, **L15** concentration-dependently enhanced SpsB activity up to 150%. Further structural derivatives (**D10**, **D14**, and **D16**) also showed SpsB activation in the FRET assay, correlating with their observed antibiotic activity (Figures 1c and S7). Importantly, **L15** and **PK150** retained enzyme activation in the presence of various detergents below their critical micellar concentrations to prevent compound aggregation, demonstrating a compound-specific effect on SpsB turnover (Figure S8).

Recently, the binding site and mechanism of activation of **PK150** in SpsB were deciphered by our group.^43^ According to this model, **PK150** binds to an allosteric pocket of SpsB by interaction with crucial residues F67, Y75, and F158, restricting access of water molecules to the active site. This stabilizes the catalytic geometry, thus enhancing enzyme activity. Measuring enzyme turnover of the crucial SpsB mutants (F67A, Y75A, and F158A) in the presence of **L15** abolished activation identically to **PK150**, while Q165A, a control mutation that does not affect binding, still showed activation (Figure S9). To rationalize how **L15** mediates activation, we utilized **L15-P** for the binding site identification of recombinantly expressed maltose-binding-protein tagged SpsB (Figure S9) via mass spectrometry using isotopically labeled desthiobiotin (isoDTB) azide tags.^44,45^ Two modified peptides, VAVNIVGYK (1) and AFGLIDEDQIVGK (2), were found to be labeled by **L15-P** (Tables S9 and S10). Peptide 1 is located in the transmembrane region inaccessible in the native protein. However, peptide 2 is in proximity to the previously identified allosteric pocket (F67, Y75, and F158) essential for SpsB activation.^43^

To validate this activation mechanism, we modeled **L15** into the allosteric pocket and investigated enzyme activation via MD simulation (Figure 4b–e). Higher residence times in holo SpsB (no substrate bound) were observed for **L15** and correlated with a strong activation effect as previously observed for **PK150** (Figure 4c).^42^ Additionally, **L15** shows the optimal required catalytic distance of the H-bond in the catalytic dyad formed by S36 and K77 (Figure 4d) and a reduced number of water molecules in the active site (Figure 4e) similar to **PK150**, enhancing the overall catalytic efficiency.^43^ Contrary, **D13** fails to stabilize the active site geometry of the holo SpsB enzyme, emphasizing the specific activating mechanism of **L15** (Figure 4c). Of note, the guanidinium group forms two H-bonds with D162 and Q165, thereby strengthening the interaction with SpsB and highlighting the necessity of this functional group for target binding (Figure S10).

Although we cannot exclude a polypharmacological mode of action of **L15**, enzyme activation represents a promising, so far underrepresented, and unconventional strategy to dysregulate bacterial physiology by the uncontrolled secretion of proteins.

## Conclusions

The success of antibiotic discovery programs is still limited by the selection of compounds and screening conditions, both often not tailored toward bacterial applications. We could show that the success rate can be significantly enhanced by preselecting a privileged scaffold known to be associated with antibiotic activity. Moreover, mimicking the host environment was critical for enhancing the hit rate. Here, 37 out of 246 compounds (15%) showed activity in our initial screen (100 μM) under host-mimicking conditions, while in regular, rich medium, only six compounds (2%) were identified.

Based on the promising biological activity of **L15**, this study focused on the MoA analysis in *S. aureus*. While membrane depolarization and morphological anomalies at the cell wall were observed at high compound concentrations, chemical proteomics additionally revealed two essential protein targets at lower concentrations. Validation of the bacterial signal peptidase SpsB, which is essential for the secretion of bacterial proteins, including autolysins, provided an intriguing and unconventional mechanism on how **L15** could kill the cell. Comparable to the previously identified SpsB activating antibiotic (**PK150**), our data suggest that **L15** binds to an allosteric pocket and enhances turnover by restricting water influx into the active site. Thus, regulating SpsB activity by small molecules may represent a more commonly observed phenomenon that may also have a physiological role in *S. aureus*. Together with the sequencing results of **L15**-resistant strains that did not reveal mutations in essential targets, a polypharmacological mode of action of **L15** is likely responsible for bacterial cell death.

Overall, our study stimulated the rethinking of antibiotic development by designing out-of-the-box approaches combining preselected antibiotic motifs with host infection-mimicking assay conditions as well as consolidated target identification platforms for rapid, in-depth MoA analysis.^46^

## Supporting Information Available

Data and code availability, chemical structures of screening hits, membrane depolarization of **L15** derivatives, visualization of single nucleotide polymorphism in norA gene, fluorescence SDS-PAGE and volcano plot results of competitive A*f*BPP experiment, relative LFQ intensities of vraRS, compound-induced cleavage by SpsB, H-bonds of **L15**’s guanidinium group with SpsB, biological activity results (MIC, MTT) of tested compounds, CFUs for FoR determination, functional enrichment of proteins from the whole proteome, expected and detected mass shifts and modified closed search analysis of isoDTB binding site experiment, experimental part of biological experiments and synthetic procedures, and compound characterization data (PDF)
