# Supplementary material for: Screening Privileged Alkyl Guanidinium Motifs under Host-Mimicking Conditions Reveals a Novel Antibiotic with an Unconventional Mode of Action
Source: JACS Au. 2024 Jul 16;4(8):3125–34. doi: 10.1021/jacsau.4c00449 (PMC11350587; doi:10.1021/jacsau.4c00449)
Supplement: Supplementary file 1 — au4c00449_si_001.pdf [file au4c00449_si_001.pdf]

Screening privileged alkyl guanidinium motifs under host-mimicking conditions reveals a novel antibiotic with an unconventional mode of action

Dominik Schum*[a], Franziska A. V. Elsen*[a], Stuart Ruddell[a], Kenji Schorpp[b], Howard Junca[c], Mathias Müsken[d], Shu-Yu Chen[e], Michaela K. Fiedler[a], Thomas Pickl[f], Dietmar H. Pieper[c], Kamyar Hadian[b], Martin Zacharias[e], Stephan A. Sieber[+a]

Affiliations

a TUM School of Natural Sciences, Department Biosciences, Chair of Organic Chemistry II, Center for Functional Protein Assemblies (CPA), Technical University of Munich (TUM), Ernst-Otto-Fischer Str. 8, Garching, 85748, Germany

b Helmholtz Zentrum München, Research Unit Signaling and Translation, Ingolstädter Landstraße 1, 85764 Neuherberg, Germany

c Helmholtz Centre for Infection Research, Microbial Interactions and Processes, Inhoffenstraße 7, 38124 Braunschweig, Germany

d Helmholtz Centre for Infection Research, Central Facility for Microscopy, Inhoffenstraße 7, 38124 Braunschweig, Germany

e TUM School of Natural Sciences, Department Biosciences, Theoretical Biophysics (T38), Center for Functional Protein Assemblies (CPA), Technical University of Munich (TUM), Ernst-Otto-Fischer Str. 8, Garching, 85748, Germany

f TUM School of Natural Sciences, Department of Chemistry, Catalysis Research Center (CRC), Technical University of Munich (TUM), Ernst-Otto-Fischer Str. 1, Garching, 85748, Germany

* contributed equally to this work

[+] corresponding author (stephan.sieber@tum.de)

Table of Content

Data and Code Availability 3

Supplementary Figures S1 – S10 4

Supplementary Tables S1 – S10 11

Methods 19

Chemical Synthesis and Analytical Data 34

References 110

Data Availability

All data are available at request from the authors. The mass spectrometry proteomics data have been deposited to the ProteomeXchange Consortium via the PRIDE[1] partner repository with the dataset identifier PXD051986. Whole genome sequencing data and metadata are available on the SRA archive under NCBI BioProject number PRJNA1103407. Molecular dynamics simulations, including input, output, and trajectory files, are available on request from the authors. Source data, initial PDB files, and the code for rating generate the figures, which are available at Zenodo DOI: 10.5281/zenodo.10950173. Bacterial strains, plasmids, and chemical compounds used in this work are available upon request from the authors or can be purchased commercially as stated.

Code availability

Custom code for performing MD simulations and analysis is available from the cited references in the paper. In house analysis code is available upon request from the authors. The details on the versions and parameters can be found in Supplementary Information.

Supplementary Figures S1 – S10

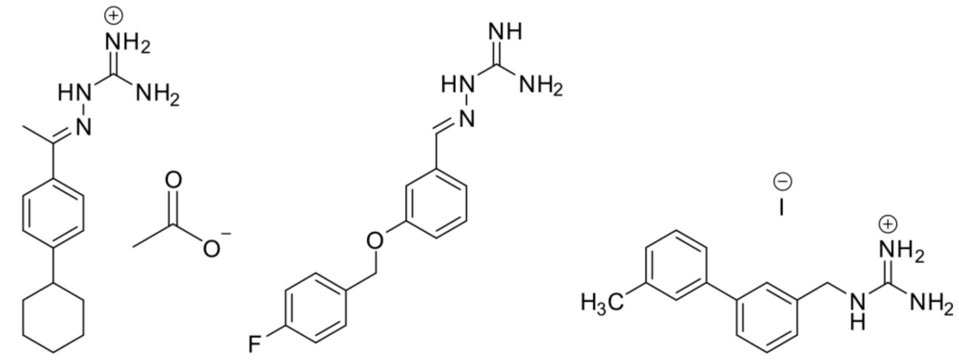

H03 J08 L09

Figure S1: Chemical structures of **H03**, **J08** and **L09**.

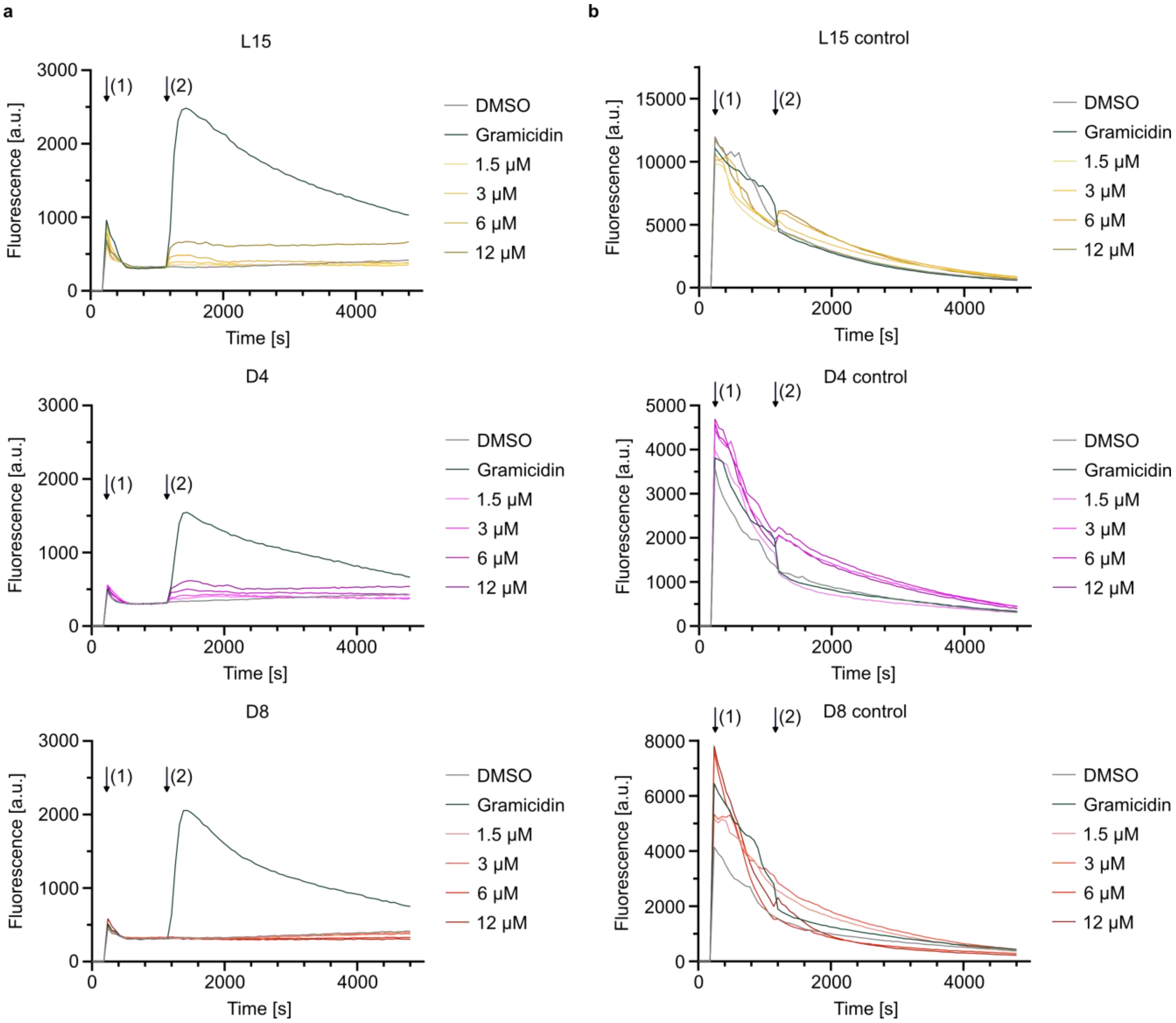

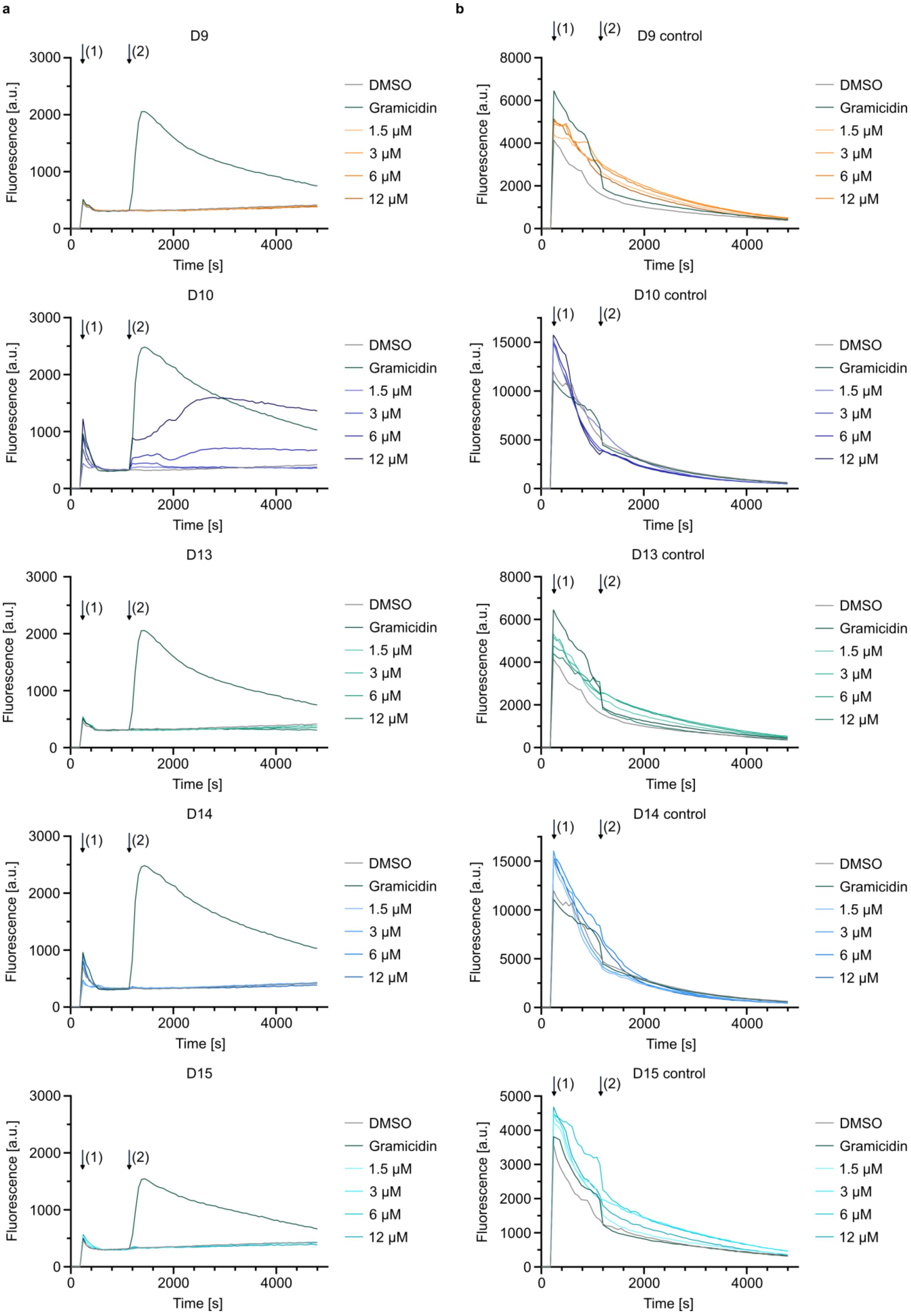

a
b
D9
D10
D13
D14
D15
D9 control
D10 control
D13 control
D14 control
D15 control
(1)
(2)
Fluorescence [a.u.]
Time [s]
DMSO
Gramicidin
1.5 µM
3 µM
6 µM
12 µM

Figure S2: Membrane depolarization of **L15** and derivatives in *S. aureus* USA300 Lac (JE2) cells. (**a**) Fluorescence of membrane potential-sensitive dye 3,3'-dipropylthiadicarbocyanine iodide ($DiSC_3(5)$) was recorded at λ_{ex} = 610 nm and λ_{em} = 660 nm. The black arrows indicate the addition of DiSC3(5) (1) and compound (2), respectively. 1 µM gramicidin was used as a positive control. (**b**) Fluorescence of $DiSC_3(5)$ in the absence of cells, but otherwise, it is in the same conditions as in (**a**). Data are representative for n = 3 biologically independent experiments per condition.

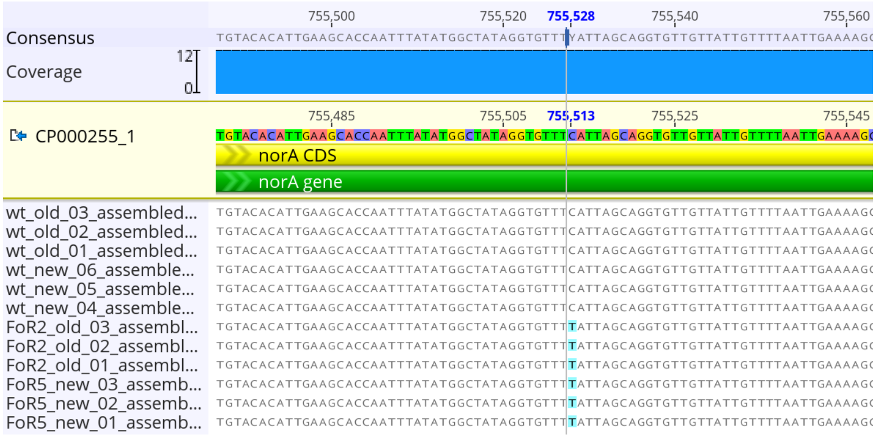

Figure S3: Visualization of single nucleotide polymorphism (SNP) in norA gene of *S. aureus* USA300 Lac (JE2) in mutants (labeled FoR2 or FoR5 *old* and *new*) obtained from FoR assays compared to wildtypes (labeled wt *old* and *new*). The SNP shows a change from C to T at position 755513. Data show results from n = 3 biologically independent replicates from two independent experiments. The mutated nucleotide is highlighted in cyan. Visualization was done with Minimap2.[2].

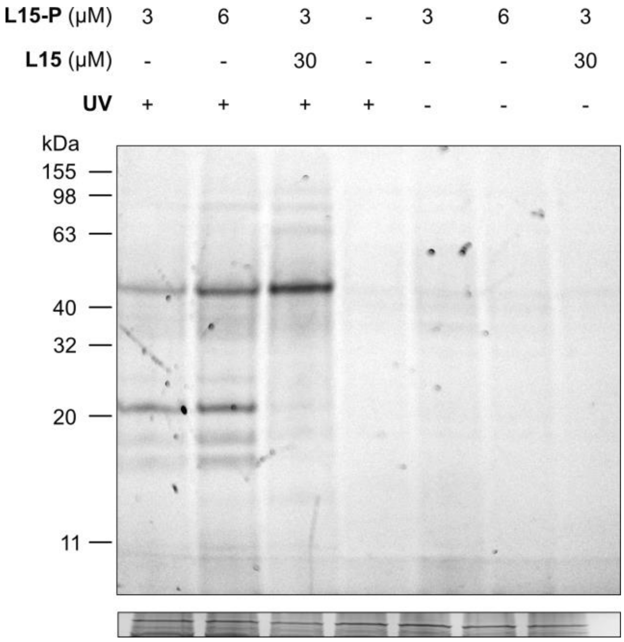

Figure S4: Fluorescence SDS-PAGE of *S. aureus* USA300 Lac (JE2)-labeled cells with **L15-P** and competition with a ten-fold excess of **L15**. The data are representative for n = 3 biologically independent experiments per condition.

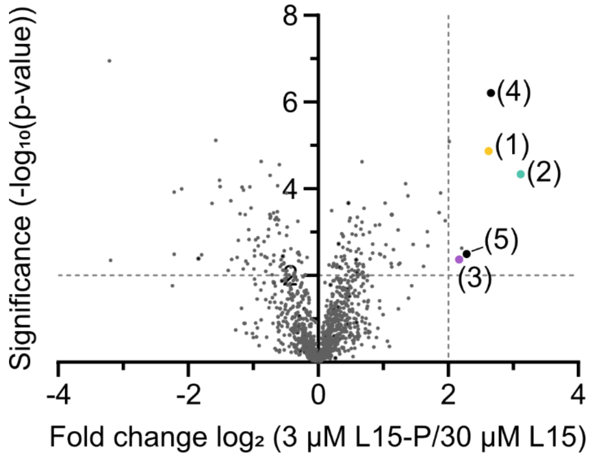

Figure S5: Target identification by chemical proteomics in *S. aureus* USA300 Lac (JE2) cells in a competitive A*f*BPP experiment. Vulcano plot of *S. aureus* treated cells (3 µM **L15-P** and 30 µM **L15**) compared to DMSO. The vertical and horizontal dashed lines represent a $\log_2$-fold enrichment ratio of 2 and a $-\log_{10}$ p-value of 2, respectively. The yellow and purple dots represent both essential proteins SpsB (1) and an uncharacterized protein (3), respectively. The cyan dot represents an aminopyrimidine aminohydrolase (2) for which the respective transposon mutant was tested for MIC shifts of **L15** (**Table S7**). Proteins (4) and (5) are two additional non-essential proteins that were also found in non-competitive A*f*BPP experiments (**Figure 3**, **c**). Numbers indicate enriched proteins in A*f*BPP and competitive A*f*BPP experiments and are summarized in a table (**Figure 2**, **d**). A two-sample students' t-test, including permutation-based multiple testing correction (FDR = 0.05), was performed to calculate the fold-change and statistical relevance. The data represent n = 4 biologically independent replicates.

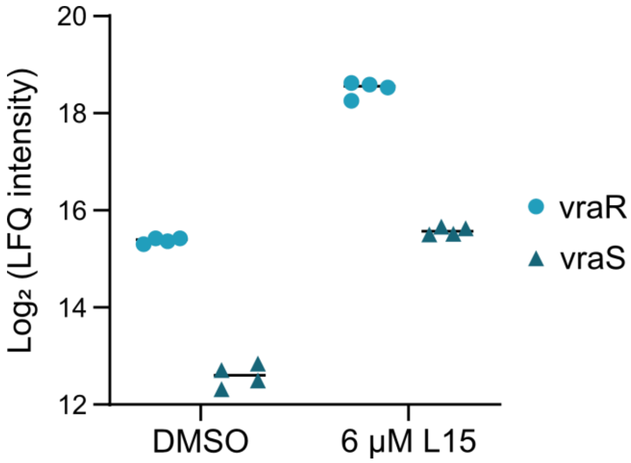

Figure S6: Relative LFQ intensities of vraR (UniProt ID: A0A0H2XGC9) and vraS (UniProt ID: A0A0H2XJC3) of DMSO and **L15**-treated (6 µM) *S. aureus* USA300 Lac (JE2) cells. The data represent n= 4 biologically independent replicates.

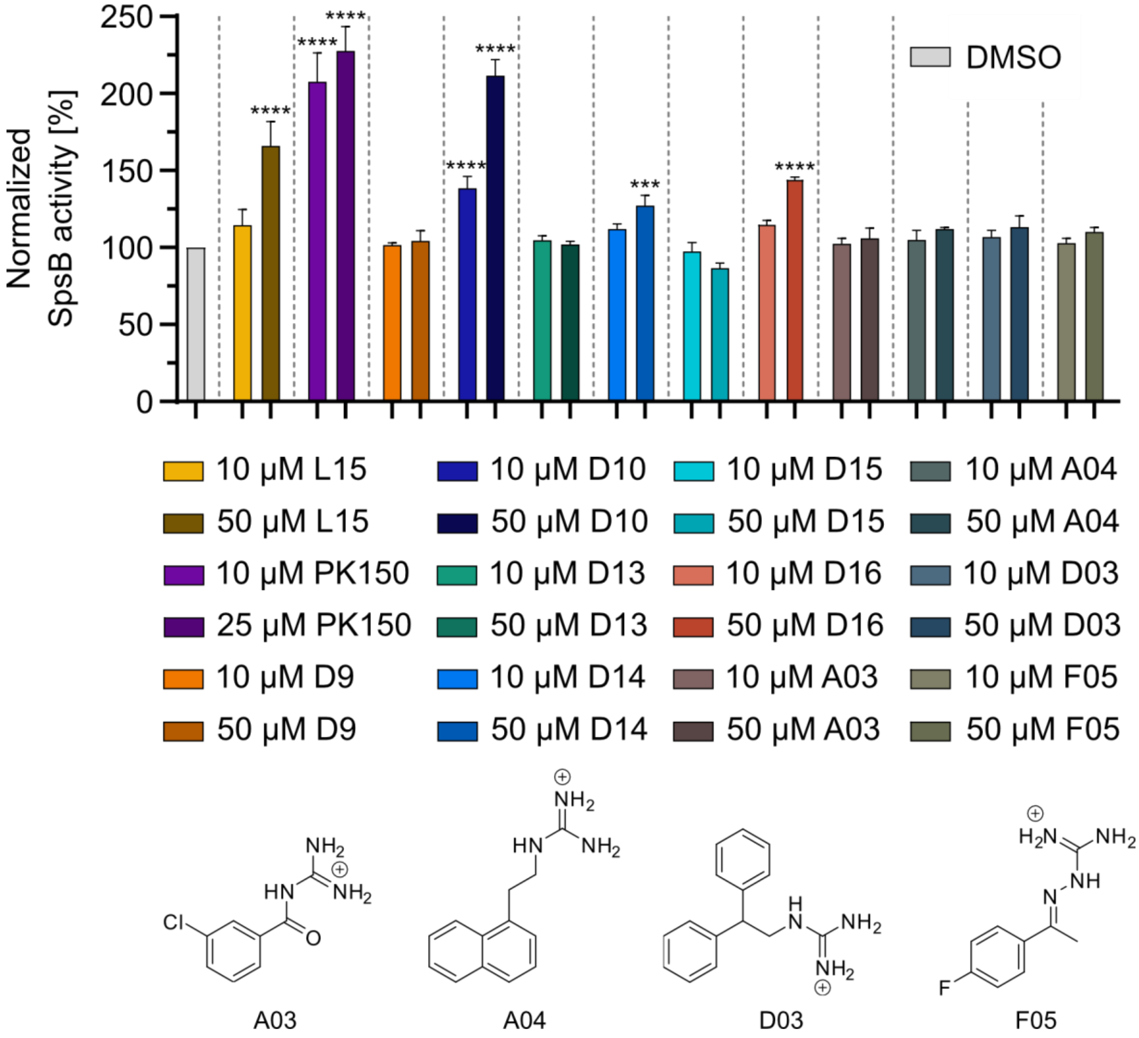

Figure S7: Compound-induced (10 µM and 50 µM) cleavage of FRET substrate by membrane-bound wildtype SpsB (50 µg mL^{-1} total membrane protein concentration). Membranes were extracted from *E. coli* BL21(DE3) pLysS cells harboring pET-55-DEST-SpsB. Substrate cleavage rates are normalized to DMSO-treated samples from the induced membranes. Background activity from non-induced membranes was subtracted before normalization. The data represent mean values ± s.d. of averaged technical duplicates. N = 3 biologically independent experiments per group were performed. P-values were calculated with one-way ANOVA statistical testing for compound- versus DMSO-treated groups: p-value < 0.05 (*), < 0.01 (**), < 0.001 (***), and < 0.0001 (****). Structures of **A03**, **A04**, **D03**, and **F05** originated from the HTS screen and were chosen as additional control compounds for the FRET assay.

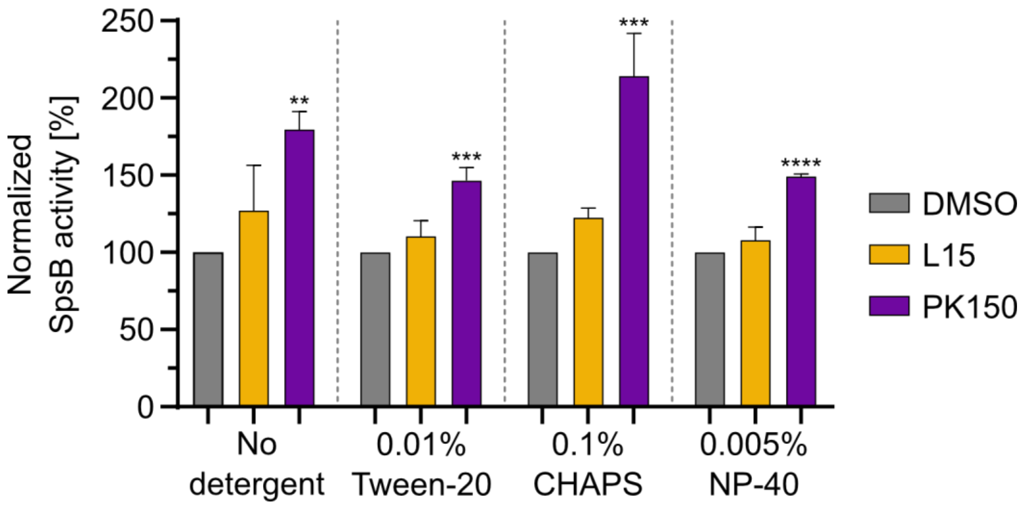

Figure S8: **L15**- and **PK150**-induced (10 µM) cleavage of FRET substrate by membrane-bound wildtype SpsB (50 µg mL^{-1} total membrane protein concentration) with or without the addition of detergents (below critical micellar concentration (cmc): 0.01% Tween[3], 0.001% NP-40[4], 0.1% CHAPS = 1.6 mM < (5.4 – 11) mM [5, 6]. Membranes were extracted from *E. coli* BL21(DE3) pLysS cells harboring pET-55-DEST-SpsB. Substrate cleavage rates are normalized to DMSO-treated samples from the induced membranes. Background activity from non-induced membranes was subtracted before normalization. The data represent mean values ± s.d. of averaged triplicates of n = 3 biologically independent experiments per group. P-values were calculated with one-way ANOVA statistical testing for compound- versus DMSO-treated groups: p-value < 0.05 (*), < 0.01 (**), < 0.001 (***), and < 0.0001 (****).

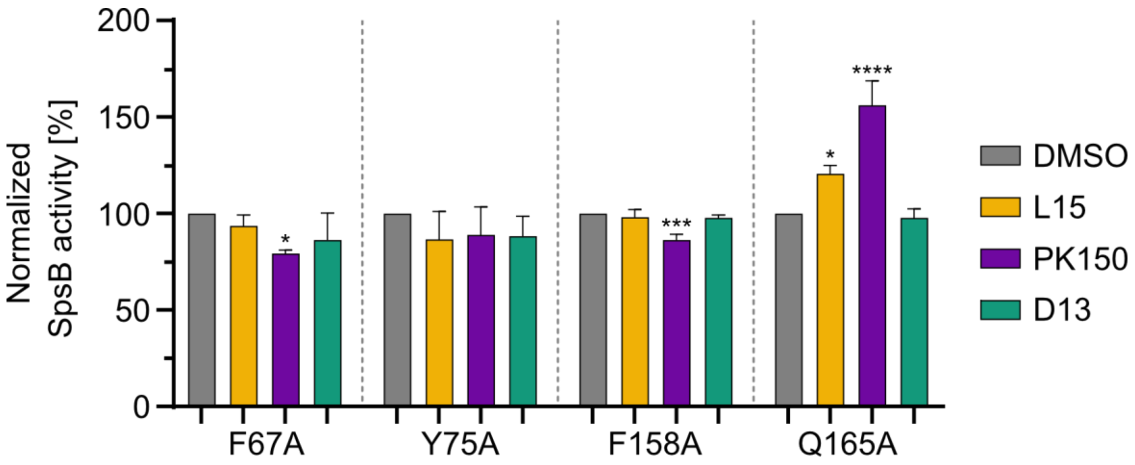

Figure S9: **L15**-, **D13**- and **PK150**-induced (10 µM) cleavage of FRET substrate by membrane-bound wildtype SpsB or respective mutants[7] (50 µg mL^{-1} total membrane protein concentration). Membranes were extracted from *E. coli* BL21(DE3) pLysS cells harboring pET-55-DEST-SpsB. Substrate cleavage rates are normalized to DMSO-treated samples from the induced membranes. Background activity from non-induced membranes was subtracted before normalization. The data represent mean values ± s.d. of averaged triplicates of n = 3 biologically independent experiments per group. P-values were calculated with one-way ANOVA statistical testing for compound- versus DMSO-treated groups: p-value < 0.05 (*), < 0.01 (**), < 0.001 (***), and < 0.0001 (****).

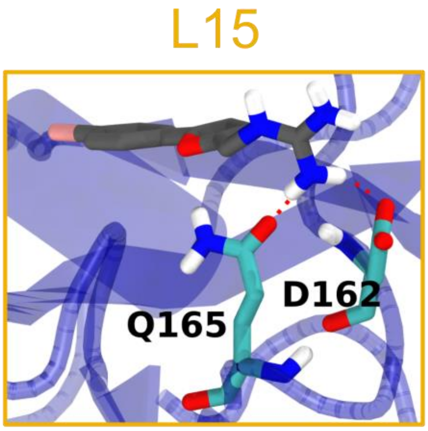

Figure S10: H-bonds of **L15**'s guanidinium group with SpsB residues D162 and Q165.

Supplementary Tables S1 – S10

Table S1: Biological activity of **L15**, **H03**, **J08**, and **L09** in a panel of pathogenic bacterial strains, including ESKAPE pathogens, in the presence of 25 mM $NaHCO_3$(*). Activity in the absence of $NaHCO_3$ is shown in brackets for *E. coli* 536 and *S. aureus* USA300 Lac (JE2). The data represent average values of n = 3 biologically independent experiments per compound.

Bacterial strain	MIC [µM]* **L15**	MIC [µM]* **H03**	MIC [µM]* **J08**	MIC [µM]* **L09**
***Escherichia coli* K12**	**12.5**	**50.0**	**50.0**	**25.0**
Escherichia coli 536	12.5 (100)	50.0 (25.0)	50.0 (50.0)	25.0 (100)
Escherichia coli CFT073	25.0	50.0	50.0	25.0
Pseudomonas aeruginosa PAO1	>100	100	>100	>100
Acinetobacter baumannii DSM 30007	>100	50.0	25.0	>100
Klebsiella pneumoniae DSM 30104	100	25.0	50.0	50.0
Enterobacter cloacae DSM 30054	50.0	25.0	50.0	100
Salmonella enterica serovar enteritidis	>100	100	100	>100
Staphylococcus aureus USA300 Lac (JE2)	1.56 (12.5)	6.25 (12.5)	12.5 (50.0)	1.56 (25.0)
Enterococcus faecium DSM 20477	100	6.25	12.5	100
Enterococcus faecium DSM 17050	100	6.25	50.0	>100
Enterococcus faecalis V583	100	6.25	50.0	>100
Listeria monocytogenes EGD-e	50.0	3.13	6.25	100

Table S2: Biological activity of **L15** in the membrane- and efflux pump-deficient E. coli strains in the presence of membrane permeabilizer polymyxin B nonapeptide (PMBN) and 25 mM $NaHCO_3$(*). The data represent the average values of n = 3 biologically independent experiments per condition.

Bacterial strain	MIC [µM]* **L15**	
	no PMBN	+ 1 µg/mL PMBN
E. coli BW25113	6.25	3.13
E. coli BW25113 ΔTolC, JW5503	12.5	3.13
E. coli BW25113 ΔBamB, JW2496	6.25	6.25

Table S3: Biological activity of **L15**, synthesized derivatives **D01** – **D18**, **H03**, **J08** and **L09** in HeLa cells. The data represent average values of n = 3 biologically independent experiments per compound.

Compound	IC_{50} [µM]
**L15**	4.0 – 5.7
**H03**	1.6 – 1.8
**J08**	3.0 – 3.6
**L09**	2.2 – 6.1
**D01**	2.9 – 5.6
**D02**	2.0 – 3.4
**D03**	4.7 – 11.5
**D04**	5.6 – 22.5
**D05**	2.3 – 3.3
**D06**	1.6 – 2.2

**D07**	2.0 – 6.3
**D08**	> 100
**D09**	> 100
**D10**	0.8 – 1.7
**D11**	7.1 – 14.5
**D12**	8.8 – 25.7
**D13**	> 100
**D14**	9.6 – 14.9
**D15**	> 50
**D16**	6.3 – 9.1
**D17**	3.0 – 5.6
**D18**	1.6 – 2.2

Table S4: CFUs for frequency of resistance (FoR) determination. The data are representative of n = 2 biologically independent experiments.

CFU/mL of test inoculum	Colonies on 6 µM **L15**-containing plate	Frequency of resistance (FoR)
0.64×10^9 CFU/mL	18	2.8×10^{-8}
0.62×10^9 CFU/mL	14	2.3×10^{-8}

Table S5: Biological activity of **L15** in *S. aureus* USA300 Lac (JE2) mutants generated by frequency of resistance (FoR) assay in the presence of 25 mM $NaHCO_3$(*). The data represent average values of n = 3 biologically independent experiments per mutant.

S. aureus USA300 Lac (JE2) FoR mutant	MIC [µM]* **L15**
Mutant_1	12.5
Mutant_2	12.5
Mutant_3	12.5

Table S6: Biological activity of **L15**, **ciprofloxacin** and **norfloxacin** in *S. aureus* USA300 Lac (JE2), *S. aureus* USA300 Lac (JE2) mutants generated by frequency of resistance assay (FoR mutant) and *S. aureus* USA300 Lac (JE2) Δ*norA* transposon mutant (Δ*norA* transposon mutant) (Nebraska transposon mutant library[8], with and without 25 mM $NaHCO_3$(*). The data represent average values of n = 3 biologically independent experiments per condition.

Bacterial strain	MIC [µM] **L15**		MIC [µM] **Ciprofloxacin**		MIC [µM] **Norfloxacin**	
	25 mM $NaHCO_3$*	No $NaHCO_3$	25 mM $NaHCO_3$*	No $NaHCO_3$	25 mM $NaHCO_3$*	No $NaHCO_3$
S. aureus USA300 Lac (JE2)	1.56	12.5	50.0	25.0	>100	100
FoR mutant	6.25	25.0	25.0	12.5	100	25.0
Δ*norA* transposon mutant	0.78	12.5	50.0	25.0	100	50.0

Table S7: Biological activity of **L15** in *S. aureus* USA300 Lac (JE2) and *S. aureus* USA300 Lac (JE2) Δ*tenA* transposon mutant (Δ*tenA* transposon mutant) (Nebraska transposon mutant library)[8] in presence of 25 mM $NaHCO_3$(*). The data represent average values of n = 3 biologically independent experiments per group.

Bacterial strain	MIC [µM]* **L15**
S. aureus USA300 Lac (JE2)	1.56
Δ*tenA* transposon mutant	1.56

Table S8: Functional enrichment of proteins from whole proteome of *S. aureus* USA300 Lac (JE2) cells treated with 6 µM **L15**. Only proteins with a $\log_2$-fold enrichment ratio of ≥1.2 or ≤-1.2 (with some exceptions, highlighted with two asterisks (**)) and a $-\log_{10}$ p-value of ≥2 (two sample t-test over normalized protein ratios) were analyzed for protein-protein interactions with STRING.[9] The table includes functional enrichment by biological process (BP), molecular function (MF), cellular component (CC), local network cluster (CL) (STRING), KEGG pathways[10], and annotated keywords (KW) (UniProt)[11, 12]. Proteins labeled with an asterisk (*) were not directly included in the functional enrichment by STRING. Functional enrichment terms ‘Fe sequestration’, ‘Peptide transport’, and ‘Hemin import’ were not annotated by STRING. The data represent n = 4 biologically independent replicates.

Functional enrichment	UniProt ID	Gene name	Protein description	$\log_2$ fold change
**Upregulation**				
Protein folding (BP, CL)	A0A0H2XFR7	clpB	Chaperone protein ClpB	3.47
	Q2FGE4	dnaJ	Chaperone protein DnaJ	2.31
	Q2FGE2	grpE	Protein GrpE	2.20
	Q2FGE3	dnaK	Chaperone protein DnaK	2.05
	Q2FF95	groEL	Chaperonin GroEL	2.00
	Q2FF94	groES	Co-chaperonin GroES	1.92
	Q2FGE1	hrcA	Heat-inducible transcription repressor HrcA	1.76
	Q2FFQ5	prsA	Foldase protein PrsA	1.38
Stress response to Cd and Co (BP, KW)	A0A0H2XEU5	SAUSA300_0508 (mcsA)	Excinuclease ABC subunit B	3.24
	Q2FJB6	mcsB	Protein-arginine kinase	3.17
	Q2FJB5	clpC	ATP-dependent Clp protease ATP-binding subunit ClpC	3.02
	Q2FJB8	ctsR	Transcriptional regulator CtsR	2.54
	Q2FDV0*	copA	Copper-exporting P-type ATPase	1.01**
Lipid oxidation and fatty acid degradation (CL)	A0A0H2XJY2	fadE	Putative long chain fatty acid-CoA ligase VraA	3.61
	A0A0H2XGT7	SAUSA300_0229 (fadX)	Putative acyl-CoA transferase FadX	3.42
	A0A0H2XHZ4	fadD	Acyl-CoA dehydrogenase FadD	2.66
	A0A0H2XFB6	SAUSA300_0226	Enoyl-CoA hydratase	2.62

	A0A0H2XKL6	SAUSA300_0225 (vraB)	Probable acetyl-CoA acyltransferase	2.26
	A0A0H2XIA4	SAUSA300_0559 (vraA)	Putative long chain fatty acid-CoA ligase VraA	1.38
Peptidoglycan ancor and cell wall (KW)	A0A0H2XGC9*	vraR	DNA-binding response regulator	3.12
	A0A0H2XHE4*	SAUSA300_1867	Cell wall-active antibiotics response LiaF-like C-terminal domain-containing protein	3.04
	A0A0H2XJC3*	vraS	Sensor protein VraS	2.98
	A0A0H2XHK2	clfB	Clumping factor B	2.62
	Q2FFM1*	mgt	Monofunctional glycosyltransferase	2.08
	A0A0H2XF70	SAUSA300_2581 (sasF)	Putative surface anchored protein	2.01
	Q2FHV0	isdC	Iron-regulated surface determinant protein C	1.73
	A0A0H2XG16	clfA	Clumping factor A	1.68
	Q2FJ77	sdrE	Serine-aspartate repeat-containing protein E	1.66
	Q2FJ79	sdrC	Serine-aspartate repeat-containing protein C	1.54
	Q2FG07	isdH	Iron-regulated surface determinant protein H	1.37
Secreted (KW)	A0A0H2XHK2	clfB	Clumping factor B	2.62
	A0A0H2XF37	lytM	Lysostaphin	2.45
	A0A0H2XF70	SAUSA300_2581 (sasF)	Putative surface anchored protein	2.01
	A0A0H2XEH7	SAUSA300_1890 (sspP)	Staphopain A	1.97
	Q2FF31	sceD	Probable transglycosylase SceD	1.97
	A0A0H2XEL7	ssaA	Secretory antigen SsaA	1.97
	Q2FHV0	isdC	Iron-regulated surface determinant protein C	1.73
	A0A0H2XI01	SAUSA300_2579	N-acetylmuramoyl-L-alanine amidase domain protein	1.82
	A0A0H2XG16	clfA	Clumping factor A	1.68
	Q2FJ77	sdrE	Serine-aspartate repeat-containing protein E	1.66
	Q2FJ79	sdrC	Serine-aspartate repeat-containing protein C	1.54
	Q2FHS7	flr	FPRL1 inhibitory protein	1.51
	Q2FG07	isdH	Iron-regulated surface determinant protein H	1.37
Phosphate transport (KW)	Q2FH48	pstS	Phosphate-binding protein PstS	7.20
	A0A0H2XIK6	phoU	Phosphate-specific transport system accessory protein PhoU	5.64

	Q2FH51	pstB	Phosphate import ATP-binding protein PstB	5.32
	A0A0H2XI00*	phoP	Alkaline phosphatase synthesis transcriptional regulatory protein PhoP	1.42
	A0A0H2XFS9*	phoR	Sensor protein kinase WalK	1.27
Fe sequestration	A0A0H2XF13	sbnC	Siderophore biosynthesis protein	1.86
	A0A0H2XG97	SAUSA300_0123 (sbnF)	Siderophore biosynthesis protein	1.36
	A0A0H2XHK0	sirA	Iron compound ABC transporter	1.36
	A0A0H2XEB0	SAUSA300_2136	Iron compound ABC transporter	1.34
	A0A0H2XH47	SAUSA300_0125 (sbnH)	Pyridoxal-dependent decarboxylase	1.34
	A0A0H2XFQ8	SAUSA300_0119 (sbnB)	Ornithine cyclodeaminase	1.26
**Downregulation**				
Nitrate metabolic process (BP, CL)	A0A0H2XI54	nirD	Nitrite reductase [NAD(P)H], small subunit	-5.27
	A0A0H2XHU0	nirB	Nitrite reductase [NAD(P)H], large subunit	-3.46
	A0A0H2XJ07	narJ	Respiratory nitrate reductase, delta subunit	-2.44
	Q2FEA5*	nreB	Oxygen sensor histidine kinase NreB	-2.23
	A0A0H2XDS6	narI	Respiratory nitrate reductase, gamma subunit	-1.76
	A0A0H2XJV6	narH	Respiratory nitrate reductase, beta subunit	-1.56
	A0A0H2XEE7	SAUSA300_2343	Nitrate reductase (quinone)	-1.44
Carotinoid biosynthetic process (BP, CL, KEGG, KW)	A0A0H2XJF9*	SAUSA300_0677	Putative deoxyribodipyrimidine photolyase	-2.19
	Q2FDU6	crtN	4,4'-Diapophytoene desaturase	-2.10
	Q2FDU3	crtP	4,4'-Diaponeurosporene oxygenase	-1.92
	Q2FDU4	crtQ	4,4'-Diaponeurosporenoate glycosyltransferase	-1.77
	Q2FDU5	crtM	4,4'-Diapophytoene synthase	-1.59
	Q2FDU2	crtO	Glycosyl-4,4'-diaponeurosporenoate acyltransferase	-1.02**
Carboxylic acid and cellular amino acid catabolism (BP)	A0A0H2XHB0	SAUSA300_0177	Acyl-CoA dehydrogenase	-1.81
	Q2FKP8	hutH	Histidine ammonia-lyase	-1.66
	Q2FEG6	hutI	Imidazolonepropionase	-1.66
	Q2FJU9	nanA	N-acetylneuraminate lyase	-1.57
	Q2FG29	ald2	Alanine dehydrogenase 2	-1.52
	A0A0H2XHP8	putA	Proline dehydrogenase	-1.51

	A0A0H2XDW3	hutU	Urocanate hydratase	-1.13**
	A0A0H2XHC2	gudB	Glutamate dehydrogenase	-0.99**
Phosphoenolpyruvate (PEP)-dependent sugar phosphotransferase system (BP, CL)	A0A0H2XHI0	SAUSA300_0331	Uncharacterized protein	-5.33
	A0A0H2XGP6	mtlA	Mannitol-specific phosphotransferase enzyme IIA component	-3.71
	A0A0H2XI57	SAUSA300_2106	Putative transcriptional regulator	-2.67
	A0A0H2XII5	SAUSA300_2576	Phosphotransferase system, fructose-specific IIABC component	-2.44
	A0A0H2XGD3	SAUSA300_0330 (sgaT)	Ascorbate-specific PTS system EIIC component	-2.27
	A0A0H2XDH0	glvC	PTS system, arbutin-like IIBC component	-2.04
	A0A0H2XI14	SAUSA300_2575	Transcriptional antiterminator	-2.03
	A0A0H2XK78	SAUSA300_0208	Putative maltose ABC transporter	-1.89
	A0A0H2XIE4	pfoR	Perfringolysin O regulator protein	-1.67
	A0A0H2XHA7	SAUSA300_1809	Putative membrane protein	-1.29
Transmembrane transport (BP, MF)	A0A0H2XE61	SAUSA300_2399	ABC transporter	-3.17
	A0A0H2XGF4	SAUSA300_0308 (tcyP)	L-cystine uptake protein TcyP	-1.83
	Q2FFH9	sdsC	Sodium-dependent dicarboxylate transporter SdcS	-1.79
	A0A0H2XKL1	glpT	Glycerol-3-phosphate transporter	-1.69
	A0A0H2XJ46	SAUSA300_0314	Sodium:solute symporter family protein	-1.52
	A0A0H2XEN8	lctP	L-lactate permease	-1.40
	Q2FII2	metN2	Methionine import ATP-binding protein MetN 2	-1.34
	A0A0H2XGA6	SAUSA300_2313	L-lactate permease	-1.32
	Q2FFJ3	putP	Sodium/proline symporter	-0.94**
Peptide transport	A0A0H2XFS6	SAUSA300_0979	Thiamine ABC transporter permease	-2.78
	A0A0H2XI71	SAUSA300_0200	Peptide ABC transporter	-2.44
	A0A0H2XIJ9	SUASA300_0978	ABC transporter	-1.81
	A0A0H2XJ57	oppF	Oligopeptide ABC transporter	-1.75
	A0A0H2XJ10	oppD	Oligopeptide ABC transporter	-1.71
	A0A0H2XK20	SAUSA300_0977	Cobalt transport family protein	-1.67
	A0A0H2XID7	oppB	Oligopeptide ABC transporter	-1.63
	A0A0H2XEU4	oppC	Oligopeptide ABC transporter	-1.50
	A0A0H2XHT0	SAUSA300_2453	ABC transporter	-1.50
	Q2FII2	metN2	Methionine import ATP-binding protein MetN 2	-1.34

Hemin import	Q2FED6	hrtB	Putative hemin transport system permease protein HrtB	-4.59
	Q2FED7	hrtA	Putative hemin import ATP-binding protein HrtA	-3.65
	A0A0H2XJF7	SAUSA300_2398	Putative membrane protein	-3.63
	A0A0H2XE61	SAUSA300_2399	ABC transporter	-3.17
	A0A0H2XI53	SAUSA300_2557	ABC transporter protein	-1.90
	A0A0H2XFY2	SAUSA300_0271	ABC transporter	-1.47
	A0A0H2XG18	SAUSA300_0309	Putative hemin import ATP-binding protein HrtA	-1.10**
	A0A0H2XFR0	SAUSA300_0272	ABC-2 type transporter transmembrane domain-containing protein	-1.06**
4Fe-4S cluster binding (BP, KW)	A0A0H2XI54	nirD	Nitrite reductase [NAD(P)H], small subunit	-5.27
	Q2FK43	pflA	Pyruvate formate-lyase-activating enzyme	-4.83
	A0A0H2XHU0	nirB	Nitrite reductase [NAD(P)H], large subunit	-3.46
	A0A0H2XFV6	queG	Epoxyqueuosine reductase	-3.41
	Q2FEI5	SAUSA300_2258	Putative formate dehydrogenase	-3.29
	A0A0H2XH15	SAUSA300_1858	Radical SAM core domain-containing protein	-2.31
	Q2FEA5	nreB	Oxygen sensor histidine kinase NreB	-2.23
	A0A0H2XIP6	queH	Epoxyqueuosine reductase QueH	-2.08
	Q2FHM0	rlmN	Probable dual-specificity RNA methyltransferase RlmN	-1.91
	A0A0H2XH44	mutY	Adenine DNA glycosylase	-1.85
	Q2FIE9	lipA	Lipoyl synthase	-1.79
	A0A0H2XEL8	SAUSA300_1536	tRNA (N(6)-L-threonylcarbamoyladenosine (37)-C(2))-methylthiotransferase	-1.68
	A0A0H2XIJ0	sdaAA	L-serine dehydratase	-1.62
	A0A0H2XEE7	SAUSA300_2343	Nitrate reductase (quinone)	-1.44
	Q2FEM4	moaA	GTP 3',8-cyclase	-1.44
	Q2FHE6	miaB	tRNA-2-methylthio-N(6)-dimethylallyladenosine synthase	-1.21

Table S9: Expected and detected mass shifts obtained from the MSFragger OpenSearch[13]. Detected mass shifts were filtered for shifts >482 Da and mass shift pairs with an exact mass difference of 6.0075 ± 0.0010 Da.

Expected mass shift (Da)	Detected mass shifts (Da)
838.4419 (**L15-P** + heavy tag)	838.4426

832.4344 (**L15-P** + light tag)	832.436
	597.357
	591.3506

Table S10 Modified closed search analysis using FragPipe[13-17] for binding site identification of **L15-P** to SpsB.

Identifier	Modified Peptide	Log_2 R Replicate 1	Log_2 R Replicate 2	Log_2 R Replicate 3	Log_2 R Replicate 4	Log_2 R Average
Q2FZT7_D126	AFGLID*EDQIVGK	0.176133	0.352867			0.2645
Q2FZT7_I125	AFGLI*DEDQIVGK			0.343479		0.343479
Q2FZT7_E127	AFGLIDE*DQIVGK				0.028252	0.028252
Q2FZT7_G17	VAVNIVG*YK	0.125478	0.22203	-0.04267	-0.05759	0.061813

Methods

Room temperature (RT) is defined as 22-25 °C.

HTS Guanidinium Library and Screen

All guanidinium compounds were commercially purchased from *Enamine Ltd*. Our customized HTS library consisted of 246 compounds with a guanidinium moiety. The library was initially screened in 384 well format with either LB medium or LB medium supplemented with $NaHCO_3$ (25 mM), and susceptibility towards each compound was assessed by optical density readout.

Chemical Compounds

PK150 ((4-Chloro-3-(trifluoromethyl)phenyl)-3-(2,2-difluorobenzo[d][1,3]dioxol-5-yl)urea) was previously synthesized according to a published procedure.[18] Heavy and light isoDTB (isotopically labeled desthiobiotin) tags were kindly provided by Dr. Stephan Hacker (Leiden University).[19] All other chemical compounds used within this work were synthesized as described (see Methods) or commercially available (ENAMINE) and used without further purification.

Cell Culture

Cell culture media and supplements were obtained from *Sigma Life Science* and *Life Technologies*. Hela cells were cultured in Dulbecco's Modified Eagle Medium–high glucose (4.5 g/L) supplemented with 10% fetal bovine serum (FBS) and 2 mM L-glutamine. Cells were maintained at 37 °C in a humidified 5% CO_2 atmosphere and detached with trypsin-EDTA.

Bacterial Strains

Available strains were obtained from the following suppliers: Horizon Discovery Ltd, UK (E. coli Keio Knockout: *E. coli* Parent Strain BW25113 (OEC5042), *E. coli* BW25113 ΔTolC JW5503 (OEC4987-213607439) and *E. coli* BW25113 ΔBamB JW2496 (OEC4987-200827790)), Institute Pasteur, France (*Listeria monocytogenes* EGD-e, *Pseudomonas aeruginosa* PAO1), American Type Culture Collection ATCC, USA (*E. faecalis* V583/ATCC 700802, *S. aureus* USA300 Lac JE(2)), Deutsche Sammlung von Mikroorganismen und Zellkulturen DSMZ, Germany (*Acinetobacter baumannii* DSM 30007, *Enterobacter cloacae subsp. cloacae* DSM 30054, *Enterococcus faecium* DSM 20477, *Enterococcus faecium* DSM 17050, *Klebsiella pneumoniae* DSM 30104). *Escherichia coli* CFT073 was a kind gift from Dr. Guiseppe Magistro (Department of Urology, Hospital of the Ludwig-Maximilians-Universität Munchen, Germany). Nebraska Transposon Mutant Library (NTML) was kindly provided by the Network on Antimicrobial Resistance in *Staphylococcus aureus* (NARSA; Medical Center, University of Nebraska, USA).[8] *Escherichia coli* K12, *Escherichia coli* 536, *Escherichia coli* BL21(DE3) and pLysS strains, and *Salmonella enterica* serovar enteritidis were provided by the Chair of Organic Chemistry II (TUM).

For cultivation of bacteria the following media were used: B medium (Lysogeny broth with 0.1% K_2HPO_4, 10 g/L casein peptone, 5 g/L NaCl, 5 g/L yeast extract, 1 g/L K_2HPO_4, pH 7.5) for all *S. aureus* strains. The addition of 25 mM $NaHCO_3$ to the media leads to a change in the pH to 8.0.LB medium (Lysogeny broth, 10 g/L casein peptone, 5 g/L NaCl, 5 g/L yeast extract, pH 7.5) was used for all *E. coli* strains, *P. aeruginosa* and *E. faecium* DSM 20477, BHI medium (Brain heart infusion, 7.5 g/L brain infusion, 10 g/L heart infusion, 10 g/L casein peptone, 5 g/L NaCl, 2.5 g/L Na_2HPO_4, 2 g/L glucose, pH 7.4) for *L. monocytogenes*, *K. pneumoniae, A baumannii, E. cloacae*, E. *faecalis*, *E. faecium* DSM 17050, and *S. enterica* serovar enteritidis..

E. coli strains used within this work (BL21(DE3) and BL21(DE3) pLysS) were cultivated in LB medium (Lysogeny Broth; 10 g/L casein peptone, 5 g/L NaCl, 5 g/L yeast extract, pH 7.5) and supplemented with respective antibiotics if indicated.

Plasmids

Plasmids and their characteristics used for the methods in the following sections, including protein purification of MBP-tagged SpsB, site-directed mutagenesis of full-length SpsB, and generation of *E. coli* membranes with full-length SpsB (wildtype and mutants) are summarized in a previous study.[7]

Minimum Inhibitory Concentration (MIC) Assay

Antibacterial activity against various bacteria was determined using minimum inhibitory concentration (MIC) assay by broth microdilution method in 96-well plates (Transparent Nunc 96-well flat bottom, *Thermo Fisher Scientific*). The bacterial inoculum was $5x10^5$ CFU/mL.[20] Briefly, a 0.5 MacFarland of bacterial overnight culture was diluted 100x in fresh medium with $NaHCO_3$ (50 mM). 50 µL of each cell suspension was added to 96-well plates containing serially diluted concentrations of each compound in 50 µL, supplemented with 2% DMSO (1% final DMSO concentration). In addition, a negative control with no compound was prepared (growth control). After 24 h of incubation at 37 °C and 200 rpm, the 96-well plates were analyzed for microbial growth, indicated by turbidity. The read-out was performed via optical density at λ = 600 nm using an Infinite™ M Nano Tecan 200Pro plate reader. MIC values were considered to be the lowest compound concentration where no bacterial growth was observed by eye. Each measurement was performed in n = 3 biologically independent replicates per compound and concentration with three technical replicates each.

Hemolysis Assay in Sheep Blood

Hemolytic activity, and a possible effect on the cell membrane, were assessed in sheep blood by liquid hemolysis assay in 96-well plates (Transparent Nunc 96-well flat bottom, *Thermo Fisher Scientific*). 5 mL of sheep blood (100%) was washed three times with 10 mL cold PBS for 5 min at 500 x *g* and RT, following dilution to 3.1% with cold PBS and $NaHCO_3$ (32.25 mM). 80 µL of this dilution was carefully transferred to each well of the 96-well plate, followed by 20 µL of each compound (final concentration of erythrocytes = 2.5%). As a positive control, 0.5% Triton X-100 was used. After 30 min of incubation at 37 °C and 5% CO_2, cells were centrifuged at 500 x *g* and RT for 10 min, and 50 µL of the supernatant was carefully transferred into a new 96-well plate. The read-out was performed via optical density at λ = 540 nm using an Infinite™ M Nano Tecan 200Pro plate reader. Each measurement was performed in n = 3 biologically independent replicates per compound and concentration with three technical replicates each. Each value was baseline-corrected with the lowest compound concentration to determine hemolytic activity before normalization to Triton X-100.

Human Metabolic Activity Assay (MTT)

Cytotoxicity was assessed in human epithelial HeLa cells by MTT assay in 96-well plates (Transparent Nunc 96-well flat bottom, *Thermo Fisher Scientific*). 4000 HeLa cells in Dulbeccos Modified Eagle Medium (DMEM) high glucose with 10% FBS and 2 mM L-glutamine were seeded per well and incubated for 24 h at 37 °C and 5% CO2. The medium was then replaced by 100 µL of fresh DMEM with the respective compound concentrations ranging from 0.39 µm to 100 µm or DMSO as a control (1% final DMSO concentration). After 24 h of incubation, 20 µL 3-(4,5-dimethyl-

2-thiazolyl)-2,5-diphenyl-2H- tetrazolium bromide solution (MTT, 5 mg/mL in PBS) was added to each well and cells were further incubated for 2 h. The supernatant was removed, and formazan crystals were solubilized with 200 µL DMSO. The read-out was performed via optical density at λex = 570 nm and λem = 630 nm (background) using an Infinite™ M Nano Tecan 200Pro plate reader. Each measurement was performed in at least n = 3 biologically independent replicates per compound and concentration with three technical replicates each. To calculate IC50 values (concentration at which 50% viability is reached), background absorbance was first subtracted, and each value was baseline-corrected with the highest compound concentration before normalizing to DMSO. The data were fitted as log(inhibitor) versus response variable slope (four parameters) non-linear regression using *GraphPad*Prism 10.01.

Membrane Depolarization Assay

The protocol was adapted from a published literature procedure.[21] *S. aureus* USA300 Lac (JE2) overnight culture was diluted to OD_{600} = 0.05 in 20 mL medium with 25 mM $NaHCO_3$. Cells were grown until the early exponential phase until they reached to OD_{600} = 0.5 and adjusted to OD_{600} = 0.3 in 5 mM HEPES supplemented with 20 mM glucose and 25 mM $NaHCO_3$. Fluorescence was recorded at λ_{ex} = 610 nm and λ_{em} = 660 nm using an Infinite™ M Nano Tecan 200Pro plate reader for 3 min, following the addition of the fluorescence membrane potential-sensitive dye 3,3'-dipropylthiadicarbocyanine iodide ($DiSC_3(5)$) (1 µM final concentration). The dye was incubated, and the measurement continued for an additional 15 min in order to reach equilibrium. Subsequently, compounds (1.5 µM, 3 µM, 6 µM, and 12 µm) or DMSO as a control (1% final DMSO concentration) were added, and fluorescence was recorded for 1 h. As a positive control, 1 µM gramicidin was used. Each measurement was performed in n = 3 biologically independent replicates per compound and concentration with three technical replicates each. In addition, the same measurements were performed without any bacterial cells to see our compounds' possible quenching effects.

Electron Microscopy

Electron microscopy was performed as previously published with slight modification.[18] An overnight culture of *S. aureus* USA300 Lac(JE2) was used to inoculate 10 mL B medium with 25 mM $NaHCO_3$ to an OD_{600} = 0.05 for each condition tested. Cells were grown until they reached to OD_{600} = 0.5 at 200 rpm and 37 °C before compound treatment (1% final DMSO concentration). DMSO was used as a control. After incubation for either 3 or 8 h, treatment was stopped by adding 2 mL of 25% formaldehyde (5% final concentration) and 0.8 mL of 25% glutaraldehyde (2% final concentration) to 7.2 mL of cell suspension. Scanning electron microscopy (SEM) samples were washed in TE buffer and dehydrated in a graded series of acetone. Critical point drying was performed with an automated CPD300 (*Leica Microsystems*) before sample coating with gold/palladium using the sputter coater SCD 500 (*Bal-Tec*). Images were acquired at an acceleration voltage of 5 kV with the field emission scanning electron microscope Merlin (*Zeiss*) using both, the Everhart Thornley HESE2-detector and the inlens SE-detector.

After aldehyde fixation, transmission electron microscopy (TEM) samples were further treated with 1% osmium tetroxide in TE buffer at RT for 1 h. After washing, the samples were dehydrated in a graded series of EtOH, including an incubation step with 2% uranyl acetate at 70% EtOH. Samples were infiltered with LR White (1:1, 2:1, 100% LR White/EtOH) and polymerized at 55 °C for two days. Ultrathin sections of approx. 60 nm thickness were received cutting with a diamond knife in an Ultramicrotome Ultracut (*Reichelt*/*Leica*), and further counterstained with 4% aqueous uranyl acetate. Images were acquired with a transmission electron microscope Libra 120 (*Zeiss*) at an

acceleration voltage of 120 kV and at calibrated magnifications. EM experiments were performed in n = 2 biologically independent experiments per condition.

Time-Kill Assay

Time-kill experiments were performed as previously described.[22] An overnight culture of *S. aureus* USA300 Lac (JE2) was diluted to a final OD_{600} = 0.05 in B medium with 25 mM $NaHCO_3$. Cells were grown further until they reached OD_{600} = 0.5 (early exponential phase). Cells were then diluted to $1x10^6$ CFU/mL in a culture tube (5 mL B medium with 25 mM $NaHCO_3$) with **L15** (1.5 µM, 3 µM, 6 µM, and 12 µM) or DMSO as a control (1% final DMSO concentration), and incubated at 200 rpm and 37 °C. For each time point (1 h, 2 h, 4 h, 6 h, 8 h, and 24 h), a serial dilution of each sample was plated on agar plates with 25 mM $NaHCO_3$ for subsequent CFU calculation. Time-kill experiments were performed in n = 2 biologically independent replicates per concentration with four technical replicates each.

Minimum Bactericidal Concentration (MBC)

Minimum bactericidal concentration (MBC) was calculated based on MIC assay. The MIC was first determined according to the MIC assay. For the MBC calculation, CFUs were determined from the previously prepared MIC plate for (a) the growth control (without compound), (b) the MIC concentration, and (c) the next two higher compound concentrations by plating each 10 µL of the respective serially diluted samples on agar plates with 25 mM $NahCO_3$. The MBC is defined as the lowest concentration that reduces more than 99.9% of the viability of the initial inoculum (3x log_{10} reduction). The MBC was determined in n = 3 biologically independent replicates with three technical replicates each.

Frequency of Resistance (FoR) Assay

Spontaneous **L15**-resistant mutants were generated via frequency of resistance (FoR) assay, using a large inoculum approach.[23, 24] An overnight culture of *S. aureus* USA300 Lac (JE2) (B medium with 25 mM $NaHCO_3$) was diluted to OD_{600} = 200. Subsequently, 10 µL of this cell suspension was plated on agar plates containing 6 µM of **L15** or DMSO as a control (1% final DMSO concentration). To calculate CFU/mL, a serial dilution of the same cell suspension was plated on agar plates with 25 mM $NaHCO_3$. After incubation for 24 h at 37 °C, colonies were counted, and FoR was calculated by dividing the number of resistant colonies that grew on **L15**-containing agar plates by the CFU of the initial cell inoculum. FoR values were determined in n = 2 biologically independent replicates.

Bacterial Genomics (Sequencing of FoR mutants)

S. aureus USA300 Lac containing plasmids pUSA01 and pUSA02, the plasmid-free derivative *S. aureus* USA300 Lac (JE2), as well as mutants exhibiting increased MIC resistance towards **L15** from three replicates of frequency of resistance (FoR) assays, were subjected to total DNA extraction using the FastDNA Spin Kit for Soil (*MP Biomedicals*) following the manufacturer's instructions. DNA libraries were constructed using the NEBNext® Ultra™ II FS DNA Library Prep Kit (*New England Biolabs*) without fragment size selection and were sequenced on a Illumina MiSeq sequencer with a 300-bp paired-end protocol. On average, 1.2 million reads were obtained. Paired reads were merged by overlap (jgi.BBmerged, Version 38.84)[25]. The most closely related genome was identified by BlastN searches against RefSeq genome database (Release 221, November 6, 2023) using *de novo* generated contigs by SPAdes[26]. The two closest nearly identical reference genomes in all cases were the USA300 genomes with accession numbers CP000255 and CP020619. Plasmids pUSA01 and pUSA02 (CP000256 and CP000257) were also identified in the

corresponding case. In order to detect all single nucleotide polymorphisms (SNPs), the datasets of merged reads were mapped to the reference chromosome CP000255 with Geneious Prime Mapper (Geneious Prime 2023.1.1, https://www.geneious.com) using medium sensitivity and 5 iterations. We identified a single SNP altering C to T in position 755513, corresponding to a change in codon TCA to TTA found in all FoR mutants only. This resulted in an amino acid change in position 366 from S to L in the 'quinolone-resistant protein NorA (UniProt ID: A0A0H2XGK0, protein SAUSA300_0680). This gene is highly conserved and part of the core genome defined for reference strains of *S. aureus.* The mutation is not found in any of the reference NorA proteins of *S. aureus* strains. Raw sequence datasets of evolved **L15**-resistant mutants and the initial wildtypes of the strain USA300 LAC used in the experiment can be found in the SRA archive NCBI BioProject number PRJNA1103407. Strains for sequencing were recovered from different stocks of USA300 strain and labeled as either *old* or *new*. Sequencing experiments in two independent experiments with n = 3 biologically independent replicates each.

Gel-based Fluorescent Labeling

For each biological replicate, a 1:100 dilution of *S. aureus* USA300 Lac (JE2) overnight cultures was prepared in 25 mL B medium supplemented with 25 mM $NaHCO_3$. Cells were grown for 10 h at 37 °C and 200 rpm, and OD_{600} was adjusted to 40 with sterile PBS. For each labeling condition (A*f*BPP: 3 µM **L15-P**, 6 µM **L15-P**, and DMSO; competitive A*f*BPP: 3 µM **L15-P** and 30 µM **L15**), 200 µL of cell suspension was transferred to an Eppendorf tube and treated with the respective compound(s) (1% final DMSO concentration). The treatment lasted for 30 min. Post-treatment, the cells were transferred to a 12-well plate (Transparent 12-well tissue culture plate flat bottom, *Avantor VWR*), and UV-irradiated (FL8BL-B lamps, *Hitachi*) for 5 min while cooling. Non-UV samples underwent the same procedure without UV exposure. Afterwards, cells were transferred to Protein LoBind tubes (Eppendorf), centrifuged for 5 min at 6,000 x g and 4 °C, and the pellets were washed two times with 500 µL PBS. Lysis was done enzymatically with 200 µL PBS supplemented with 0.05 mg/mL lysostaphin (*Sigma Life Science*) for 30 min at 37 °C and 1,000 rpm. Subsequently, 4 µL of 20% SDS (0.4% final SDS concentration) was added to each sample, followed by sonication for 10 s at 20% intensity (Sonopuls HD 2070 ultrasonic rod, *Bandelin electronic GmbH*). The lysate was cleared by centrifugation for at least 30 min at 21,000 x *g* and RT, and the supernatant was transferred into new LoBind tubes. The protein concentration of each sample was determined using BCA assay (Roti Quant, *Roth*) and adjusted to 2 mg/mL. A click mix solution was prepared by combining 1 µL of 10 mM rhodamine azide in DMSO, 3 µL of 1.67 mM tris(benzyltriazoylmethyl)amine (TBTA) in a solvent mixture of 80% tBuOH and 20% DMSO, 1 µL of 50 mM $CuSO_4$ in water, and 1 µL of 100 mM tris(2-carboxyethyl)phosphine (TCEP) in water. For each protein sample, 50 µL was transferred into individual Eppendorf tubes and mixed with 7 µL of the click mix solution. Incubation was done for 60 min at RT. Proteins were precipitated by the addition of 500 µL ice-cold acetone for at least 2 h at -20 °C. After incubation, the proteins were pelleted by centrifugation for 10 min at 13,000 x *g* and 4 °C, and the resulting pellet was washed two times with 500 µL ice-cold MeOH via mild sonication 10 s at 10% intensity (5x cycle) (Sonopuls HD 2070 ultrasonic rod, *Bandelin electronic GmbH*). The supernatant was removed, and the protein pellets were air-dried for 10 min. The dried pellets were dissolved in Laemmli buffer by sonication for 10 s at 10% intensity. These samples were then subjected to SDS-PAGE using 15% polyacrylamide gels. Fluorescence was recorded with a Fujifilm Las-4000 Luminescent Image Analyzer equipped with a Fujinon VRF43LMD3 lens and a 575DF20 filter. Coomassie staining of the gels was performed to serve as a loading control. Gel-based labeling was performed in n = 3 biologically independent experiments per condition.

Preparative Labeling for LC-MS/MS Analysis

Labeling, Lysis, and Click.

For each biological replicate, a 1:100 dilution of S. aureus USA300 Lac (JE2) overnight cultures was prepared in 25 mL of B medium supplemented with 25 mM NaHCO3. Cells were grown for 10 h at 37 °C and 200 rpm, and OD_{600} was adjusted to 40 with sterile PBS. For each labeling condition (A*f*BPP: 3 µM **L15-P**, 6 µM **L15-P** and DMSO; competitive A*f*BPP: 3 µM **L15-P** and 30 µM **L15**), 200 µL of cell suspension was transferred to an Eppendorf tube and treated with the respective compound(s) (1% final DMSO concentration). The treatment lasted for 30 min. Post-treatment, the cells were transferred to a 12-well plate (Transparent 12-well tissue culture plate flat bottom, *Avantor VWR*), and UV-irradiated (FL8BL-B lamps, *Hitachi*) for 5 min while cooling. Non-UV samples underwent the same procedure without UV exposure. After photocrosslinking, the cells were transferred to Protein LoBind tubes (Eppendorf), centrifuged at 6,000 x g for 5 min at 4 °C, and the pellets were washed two times with 500 µL PBS. Enzymatic lysis was performed with 200 µL PBS containing 0.05 mg/mL lysostaphin (Sigma Life Science) for 30 min at 1,000 rpm and 37 °C. Subsequently, 4 µL of 20% SDS (final concentration 0.4%) was added to each sample, followed by 10 s of sonication at 20% intensity (Sonopuls HD 2070 ultrasonic rod, Bandelin electronic GmbH). The lysate was cleared by centrifugation for at least 30 min at 21,000 x *g* and RT, and the supernatant was transferred into new LoBind tubes. The protein concentration of each sample was determined using BCA assay (Roti Quant, *Roth*), and 45 µL of each protein sample (2.23 mg/mL) was transferred to a 96-well plate (Polypropylene, V-bottom, Greiner cat. 651201). A click mix solution was prepared by combining 0.6 µL of 20 mM biotin azide in DMSO, 2.5 µL of 1.67 mM tris(benzyltriazoylmethyl)amine (TBTA) in 80% tBuOH and 20% DMSO, 1.2 µL of 50 mM $CuSO_4$ in water, and 0.6 µL of 100 mM tris(2-carboxyethyl)phosphine (TCEP) in water. To each protein sample, 4.9 µL of the click mix solution was added, and the plate was incubated for 90 min at RT and 950 rpm. The reaction was quenched by adding 65 µL of 8 M urea in water with 10 mM TCEP and 20 mM iodoacetamide (IAA) for 15 min at 950 rpm and RT. The excess IAA was then quenched by adding 2 µL of 500 mM DTT per sample.

Enrichment and Digestion.

All reagents used were of LC-MS grade. Sample processing followed an adapted protocol for magnetic bead enrichment.[27] To each sample, 10 µL of a 2-fold concentrated 1:1 mix of washed (3x with H_2O) hydrophobic and hydrophilic carboxylate-coated magnetic beads (*Cytiva*, cat# 65152105050250 and 45152105050250) was added. This was followed by the addition of 175 µL EtOH to precipitate the proteins onto the beads. The subsequent steps were carried out using an automated liquid handling system (Hamilton Microlab Prep, *Hamilton*). The plate was then incubated for 5 min at 500 rpm and RT, following washing of the beads. Each washing step was performed with the help of a 96-well ring magnet (Alpaqua, *Magnum FLX*). For this, the plate was placed on it, and the supernatant was removed slowly (20 µL/s) to avoid removing any beads. Washing involved removing the plate from the magnet, adding the respective washing solution, and shaking for 1 min at 800 rpm and RT. The samples were washed three times with 180 µL of 80% EtOH and once with 180 µL acetonitrile. Proteins were eluted from the beads by adding 75 µL of 0.2% SDS in PBS, followed by 5 min of incubation at 800 rpm and 40 °C. The plate was placed back on the magnet in order to transfer the supernatant into new wells. This elution step was repeated, resulting in a total volume of 150 µL of eluted proteins. Next, to each well containing the eluted protein samples, 50 µL of washed (3x with 0.2% SDS in PBS) streptavidin magnetic beads (New England Biolabs, cat#

S1420S) were added. The plate was sealed and incubated for 1 h at 800 rpm and RT in a plate shaker with a heated lid (ThermoMixer C, *Eppendorf*) to allow binding of the labeled proteins to the streptavidin beads. After this incubation step, the plate was further processed with the liquid handling system. Washing of the beads was done three times with 180 µL of 0.1% NP-40 in PBS, two times with 180 µL of 6 M urea, and three times with 200 µL H_2O. Protein digestion was performed with 1 µL trypsin (trypsin/protein ratio 1:100, 0.5 µg/µL, sequencing grade, Promega) in 100 µL of 50 mM TEAB overnight at 800 rpm and 37 °C in a plate shaker with a heated lid. The plate was tightly sealed during digestion. The next day, peptides were eluted from the beads in the liquid handling system with 50 µL of 3% FA and transferred into new wells for desalting. Desalting was performed using pre-equilibrated stage tips containing two layers of styrenedivinylbenzene-reverse phase sulfonate (SDB-RPS) disks (Empore, *3M*) as previously described.[28] The stage tips were equilibrated with 150 µL wash buffer 1 (1% TFA in isopropanol) before loading the samples. Samples were loaded for 10 min at 500 x g, followed by washing with 150 µL wash buffer 1 for 10 min at 800 x g, and another wash with 150 µL wash buffer 2 (0.2% TFA in H_2O). Peptides were eluted with 50 µL elution buffer (1% ammonia, 80% acetonitrile) for 5 min at 300 x g, followed by 5 min at 800 x g. Eluted peptide samples were dried using a centrifugal evaporator (Concentrator Plus, *Eppendorf*), following reconsititution in 35 µL of 1% FA. 4 µL of each sample was subjected for LC-MS/MS measurements on a timsTOF Pro mass spectrometer (*Bruker*) in data-independent acquisition (DIA) mode. Preparative MS samples were performed in n = 4 biologically independent replicates.

Full Proteome Analysis

For each biological replicate, an *S. aureus* USA300 Lac (JE2) overnight culture was inoculated to OD_{600} = 0.06 in 5 mL B medium supplemented with 6 µM **L15** or DMSO as a control (1% final DMSO concentration) and 25 mM $NaHCO_3$. Cells were grown for 5 h at 37 °C and 200 rpm, and adjusted to OD_{600} = 2 with PBS. 1 mL of the adjusted OD_{600} was used, and cells were washed with PBS by centrifugation for 5 min at 6,000 x *g* and 4 °C. Lysis was done with 150 µL PBS supplemented with 0.5% SDS and 1% Triton X-100. Each sample was first sonicated for 10 s at 30% intensity (Sonopuls HD 2070 ultrasonic rod, *Bandelin electronic GmbH*), followed by a mechanical cell disruption. For this, the cell suspension was transferred into bead-mill tubes filled with 0.1 mm zirconium beads, and the cells were lyzed with a bead beater homogenizer (Precellys 24 Homogenizer, *Bertin Technologies*) in three cycles: 30 s at 6,500 rpm, followed by cooling for 30 s. Afterwards, the tubes were centrifuged for 10 min at 10,000 x g and RT, and the supernatant was transferred into a new Eppendorf tube, followed by an additional centrifugation for at least 30 min at 21,000 x *g* and RT. The supernatant was transferred into new LoBind tubes (*Eppendorf*), and the protein concentration of each sample was determined using BCA assay (Roti Quant, *Roth*). All samples were adjusted to a total protein amount of 15 µg in a final volume of 80 µL and transferred to a 96-well plate (Polypropylene, V-bottom, *Greiner* cat. 651201). For protein alkylation, a 1:2 mixture of tris(2-carboxyethyl)phosphine (TCEP) and iodoacetamide (IAA) of each 500 mM was prepared, and 3 µL was added to each sample. After incubation for 15 min at 950 rpm and RT, the excess of IAA was quenched by the addition of 2 µL 500 mM DTT per sample. To each sample, 10 µl of a 1:1 mix of washed (3x with H_2O) hydrophobic and hydrophilic carboxylate-coated magnetic beads (*Cytiva*, cat# 65152105050250 and 45152105050250) was added. This was followed by the addition of 150 µL EtOH to precipitate the proteins onto the beads. The subsequent steps were carried out using an automated liquid handling system (Hamilton Microlab Prep, *Hamilton*). The plate was then incubated for 5 min at 500 rpm and RT, following washing of the beads. Each washing step was performed with the help of a 96-well ring magnet (Alpaqua, *Magnum FLX*). For this, the plate was placed on it,

and the supernatant was removed slowly (20 µL/s) to avoid removing any beads. Washing involved removing the plate from the magnet, adding the respective washing solution, and shaking for 1 min at 800 rpm and RT. The samples were washed three times with 180 µL of 80% EtOH and once with 180 µL acetonitrile. Protein digestion was performed with 0.2 µL trypsin (trypsin/protein ratio 1:100, 0.5 µg/µL, sequencing grade, *Promega*) in 100 µL of 50 mM TEAB overnight at 800 rpm and 37°C in a plate shaker with a heated lid. The plate was tightly sealed during digestion. The next day, peptides were eluted from the beads in the liquid handling system with 50 µL of 3% FA and transferred into new wells for desalting. Desalting was performed using pre-equilibrated stage tips containing two layers of styrenedivinylbenzene-reverse phase sulfonate (SDB-RPS) disks (Empore, *3M*) as previously described.[28] The stage tips were equilibrated with 150 µL wash buffer 1 (1% TFA in isopropanol) before loading the samples. Samples were loaded for 10 min at 500 x g, followed by washing with 150 µL wash buffer 1 for 10 min at 800 x g, and another wash with 150 µL wash buffer 2 (0.2% TFA in H_2O). Peptides were eluted with 50 µL elution buffer (1% ammonia, 80% acetonitrile) for 5 min at 300 x g, followed by 5 min at 800 x g. Eluted peptide samples were dried using a centrifugal evaporator (Concentrator Plus, *Eppendorf*), following reconsititution in 50 µL of 1% FA. 1 µL of each sample was subjected for LC-MS/MS measurements on a timsTOF Pro mass spectrometer (*Bruker*) in data-independent acquisition (DIA) mode. Full proteome samples were performed in n = 4 independent replicates.

LC-MS measurements and data analysis timsTOF Pro

<u>LC-MS measurements on timsTOF Pro.</u>

Peptides were analyzed and separated onlineusing an UltiMate 3000 nano HPLC system (*Dionex*) linked to a *Bruker* timsTOF Pro mass spectrometer via a CaptiveSpray nano-electrospray ion source and *Sonation* column oven. Peptides were initially loaded onto the trap column (Acclaim PepMap 100 C18, 75 µm ID x 2 cm, 3 µm particle size, *Thermo Fisher Scientific*), and washed with 0.1% formic acid in water for 7 min at a flow rate of 5 µL/min. They were then transferred to the separation column (Aurora C18 column, 25 cm × 75 µm, 1.7 µm, *IonOpticks*) and separated using a gradient: 5% to 17% B over 36 minutes, then 17% to 25% B over 18 minutes, followed by 25% to 37% B over 6 minutes, and finally held at 95% B for 10 minutes before re-equilibration, with a flow rate of 400 nl/min. Mobile phase A was 0.1% (v/v) formic acid in water, while mobile phase B was 0.1% (v/v) formic acid in acetonitrile. The timsTOF Pro operated in data-independent dia-PASEF mode, with the dual TIMS analyzer set to equal accumulation and ramp times of 100 ms each and a 1/K0 ion mobility range from 0.60 to 1.60 V × s/cm² for MS1 scans. For fragmentation, dia-PASEF settings included a mass range of 400 to 1,201 m/z and an ion mobility range of 0.60 to 1.43 V × s/cm². Each dia-PASEF scan comprised two ion mobility isolation windows of 26 m/z widths. With 32 isolation windows and 1 m/z overlaps, the setup covered the mass range, resulting in 16 dia-PASEF scans per MS1 scan and a total cycle time of approximately 1.80 s (Table **S11**). Collision energy was ramped linearly from 59 eV at 1/K0 = 1.3 V × s/cm² to 20 eV at 1/K0 = 0.85 V × s/cm². TIMS elution voltages were linearly calibrated using three Agilent ESI-L Tuning Mix ions (m/z 622, 922, and 1,222) spiked into the CaptiveSpray Source inlet filter in order to obtain the reduced ion mobility coefficients (1/K0).

Table S11: DIA-PASEF scan windows including ion mobility range ($1/K_0$) and scan width (m/z).

MS Type	Scan	Start IM [1/K0]	End IM [1/K0]	Start Mass [m/z]	End Mass [m/z]
MS1	0	0.6	1.6	100	1700

dia-PASEF	1	0.9	1.2	800	826
dia-PASEF	1	0.6	0.9	400	426
dia-PASEF	2	0.92	1.22	825	851
dia-PASEF	2	0.62	0.92	425	451
dia-PASEF	3	0.93	1.23	850	876
dia-PASEF	3	0.63	0.93	450	476
dia-PASEF	4	0.95	1.25	875	901
dia-PASEF	4	0.65	0.95	475	501
dia-PASEF	5	0.96	1.26	900	926
dia-PASEF	5	0.66	0.96	500	526
dia-PASEF	6	0.98	1.28	925	951
dia-PASEF	6	0.68	0.98	525	551
dia-PASEF	7	0.99	1.29	950	976
dia-PASEF	7	0.69	0.99	550	576
dia-PASEF	8	1.01	1.31	975	1001
dia-PASEF	8	0.71	1.01	575	601
dia-PASEF	9	1.02	1.32	1000	1026
dia-PASEF	9	0.72	1.02	600	626
dia-PASEF	10	1.04	1.34	1025	1051
dia-PASEF	10	0.74	1.04	625	651
dia-PASEF	11	1.06	1.36	1050	1076
dia-PASEF	11	0.76	1.06	650	676
dia-PASEF	12	1.07	1.37	1075	1101
dia-PASEF	12	0.77	1.07	675	701
dia-PASEF	13	1.09	1.39	1100	1126
dia-PASEF	13	0.79	1.09	700	726
dia-PASEF	14	1.1	1.4	1125	1151
dia-PASEF	14	0.8	1.1	725	751
dia-PASEF	15	1.12	1.42	1150	1176
dia-PASEF	15	0.82	1.12	750	776
dia-PASEF	16	1.13	1.43	1175	1201
dia-PASEF	16	0.83	1.13	775	801

Data analysis of timsTOF Pro measurements.

MS data were processed using DIA-NN[29] (version 1.8.1) in library-free mode. The UniProt reference proteome for S. aureus USA300 (taxon identifier: 367830, downloaded on 30.06.2022) was used for library generation. The settings for precursor ion generation included the creation of a library and the use of deep-learning algorithms to predict spectra, retention times (RTs), and ion mobilities (IMs). Trypsin/P was specified as the protease, allowing for a maximum of two missed cleavages. The method involved excising N-terminal methionine and applying carbamidomethylation to cysteines as a fixed modification, with no variable modifications. The peptide lengths were set to range from 7 to 30 residues, and the precursor charges were selected to be between 2 and 4. The precursor m/z range was established from 300 to 1,800, and the fragment m/z range was set from 200 to 1,800 for TIMS data. The precursor false discovery rate (FDR) was established at 0.01. Settings for mass accuracy, MS1 accuracy, and scan window were all configured to 0. Features such as isotopologues, match-between-runs (MBR), and removal of likely interferences were activated. The neural network classifier was operated in single-pass mode, conducting protein inference at the gene level with heuristic protein inference enabled (--relaxed-prot-inf). Quantification was carried out using the robust LC (high precision) strategy. Cross-run normalization was dependent on RT, smart profiling was employed for library generation, and optimal settings were used for both speed and RAM usage. After DIA-NN analysis, LFQ quantities for all protein groups were analyzed using Perseus software[30] (version 2.03.1). LFQ intensities were $\log_2$ transformed, and protein groups were filtered to retain those with at least three valid values in one group. Missing values in **L15-P** enrichment samples were imputed from a normal distribution with default settings (width = 0.3 and down shift = 1.8 for total matrix), while no imputation was done for full proteome samples. A two-sample Student's t-test with permutation-based multiple testing correction (FDR = 0.05) was used for all relevant comparisons to determine fold change values and statistical significance. Results tables were exported, and graphs were generated using GraphPad Prism 10.01.

Expression and Purification of MBP-tagged *S. aureus* SpsB in *E. coli*

Protein expression and purification of MBP-tagged S. aureus SpsB was done by M. K. von Wrisberg as previously described.[7]

The vector pETMBP-1a-His-MBP-SpsB was transformed in *E. coli* BL21 (DE3) for subsequent protein expression. An overnight culture (LB medium supplemented with 50 µg/mL kanamycin) of the transformed strain was diluted 1:100 in fresh LB medium (1 l culture supplemented with 50 µg/mL kanamycin) and incubated at 200 rpm 37 °C until OD_{600} = 0.45 – 0.6. Subsequently, protein expression was induced with 0.3 mM isopropyl-β-D-1-thiogalactopyranoside (IPTG), and incubation was prolonged for 3 h at 200 rpm and 25 °C. Cells were harvested by centrifugation for 10 min at 5,000 x *g* and 4 °C. Protein purification was performed at 4 – 8 °C or on ice unless otherwise noted, using two purification steps:(1) MBP-affinity chromatography followed by (2)size-exclusion chromatography (SEC), both carried out on an ÄKTA-FPLC system (*GE Healthcare*, now *Cytiva*). Cell lysis was done by resuspending the pelletin 30 mL lysis buffer (20 mM Tris, pH 8, 200 mM NaCl, 1 mM EDTA, 5% (w/v) glycerol, 1 mM dithiotreitol (DTT)), containing 0.1 mg/mL DNase I (*AppliChem*) and one tablet cOmplete TM ULTRA EDTA-free protease inhibitor tablet (*Roche*), followinghomogenization using an EmulsiFlexC5 (*Avestin Inc.)*. The lysate was then obtained by centrifugation for 30 min at 25,000 x *g* and 4 °C. Before loading onto a MBPTrap HP column (*Cytiva*), the lysate was filtered using a Whatman TM folded filter (*GE Healthcare*, now *Cytiva*). After loading, the column was washed with wash buffer (20 mM Tris, pH 8, 200 mM NaCl, 1 mM EDTA, 5% (v/v) glycerol, 1 mM DTT), before eluting the proteinwith elution buffer (20 mM Tris pH 8, 200 mM NaCl, 1 mM EDTA, 5% (v/v) glycerol, 1 mM DTT, 10 mM maltose). The respective fractions with

MBP-SpsB were pooled and concentrated with an Amicon Ultracell Centrifugal filter unit (MWCO 10 kDa, *Merck Millipore*). Afterwards, the second purification step was done by loading the concentrated sample onto a equilibrated HiLoad Superdex 75 (16/60) column (*Cytiva*). Column equilibration was done withSEC buffer (20 mM Tris-HCl pH 8, 200 mM NaCl, 5% (v/v) glycerol, 1 mM DTT). Fractions with pure MBP-SpsB were pooled and concentrated. For further usage protein aliquots were snap-frozen in liquid nitrogen and stored at -80 °C. SDS-PAGE analysis confirmed protein purity (molecular weight: 62,033.20 Da).[7]

Binding Site Identification by isoDTB

The binding site of **L15-P** to SpsB was determined by a mass spectrometry workflow using isotopically labeled desthiobiotin azide tags (isoDTB)[19, 31] as described previously.[7] All reagents used were LC-MS grade.

Sample Preparation.

Eight samples of the recombinantly purified MBP-tagged extracellular domain of SpsB were diluted in PBS to a final concentration of 5 µM. Each sample, with a final volume of 100 µL, was placed in a well of a transparent, flat-bottom 96-well plate (Nunc, Thermo Fisher Scientific). **L15-P** was then added to each sample to achieve a concentration of 5 µM, and the samples were incubated on a rolling plate at RT for 45 min. Following incubation, the samples were UV-irradiated using FL8BL-B lamps (Hitachi) for 5 min, and subsequently transferred to Protein LoBind tubes (*Eppendorf*).

Click, Reduction, Alkylation, and Digestion.

Two click mix solutions with either heavy or light isoDTB (desthiobiotin azide) azide tags were prepared (36 µL 0.9 mg/mL tris(benzyltriazoylmethyl)amine (TBTA) in 80% *t*BuOH and 20% DMSO, 12 µL 13 mg/mL tris(2-carboxyethyl)phosphine (TCEP) in H_2O, 12 µL 50 mM CuSO4 in H_2O). To each click mix, 6 µL of heavy or light isoDTB (5 mM in DMSO) was added. 12 µL of final click mix solution was added to the SpsB samples (4x heavy and 4x light), followed by incubation for 1 h at RT. For each replicate, a heavy and a light sample was combined, and proteins were precipitated with 800 µL ice-cold acetone, following incubation for at least 2 h at -20 °C. Afterwards, proteins were pelleted by centrifugation for 10 min at 13,000 x *g* and 4 °C, and the resulting pellet was washed two times with 500 µL ice-cold MeOH via mild sonication for 10 s at 10% intensity (5x cycle) (Sonopuls HD 2070 ultrasonic rod, *Bandelin electronic GmbH*). The supernatant was carefully removed, and the protein pellet was allowed to air-dry for 10 min. The pellet was then dissolved in 60 µL 8 M urea (in 0.1 M triethylammonium bicarbonate buffer (TEAB) via mild sonication for 10 s at 10% intensity (5x cycle). Each sample was reduced with 3 µL 31 mg/mL dithiothreitol (DTT) in H_2O for 45 min at 850 rpm and 37 °C (ThermoMixer C, *Eppendorf*). Free cysteines were subsequently carbamidomethylated with 3 µL 74 mg/mL iodoacetamide (IAA) in H_2O for 30 min at 850 rpm and 37 °C. The remaining IAA was quenched with 3 µL 31 mg/mL DTT in H_2O for 30 min at 850 rpm and 37 °C. Digestion was done with 1 µL trypsin (ratio trypsin/protein 1:100, 0.5 µg/µL, sequencing grade, *Promega*) in 180 µL 0.1 M TEAB overnight at 220 rpm and 37 °C (Incubator Shaker, *Eppendorf New Brunswick*).

Enrichment.

600 µL of washed (3x 0.1% NP-40 in PBS) high-capacity streptavidin agarose beads (*Fisher Scientific*) were added to each digested sample and incubated for 1 h at RT while rotating (disc rotator). Samples were centrifuged for 2 min at 1,000 x *g* and RT to remove the supernatant. 600 µL 0.1% NP-40 in PBS was added to each sample and transferred onto centrifugation columns

(*Fisher Scientific* Pierce™). The beads were washed with 1x 600 µL 0.1% NP-40 in PBS, 3x 600 µL PBS and 3x 600 µL H_2O. Elution was done with 1x 200 µL 0.1% trifluoroacetic acid (TFA) in 50% aqueous H_2O, followed by 2x 100 µL of the same elution buffer. Samples were dried in a centrifugal evaporator (Concentrator Plus, *Eppendorf)* and reconstituted in 12.5 µL 0.1% TFA for LC-MS measurements on Orbitrap Eclipse Tribrid mass spectrometer (*Thermo Fisher Scientific*) in data-dependent acquisition (DDA).

LC-MS measurements on Orbitrap Eclipse Tribrid.

IsoDTB samples were analyzed according to a published procedure.[31]

Peptides were analyzed using HPLC-MS/MS on a Vanquish Neo UHPLC system (Thermo Fisher Scientific) equipped with a PepMap™ Neo 5 µm C18 300 µm x 5 mm Trap Cartridge (Thermo Fisher Scientific) and Aurora Ultimate™ separation columns (3rd generation, 20 cm nanoflow UHPLC compatible, *Ionopticks*), alongside a Nanospray Flex Ion Source (Thermo Fisher Scientific) connected to an Orbitrap Eclipse Tribrid instrument (Thermo Fisher Scientific). The Vanquish Neo UHPLC operated in Trap-and-Elute-Injection mode, loading samples onto the trap column. Subsequent separation occurred at a flow rate of 400 nl/min using buffer A (0.1% formic acid in water) and buffer B (0.1% formic acid in acetonitrile). The separation column was maintained at 40 °C. Peptides were separated over a 75 min gradient, starting from 5% to 40% buffer B for 60 min, then reaching 60% B over 5 min, followed by a 10 min isocratic wash with 90% B. For washing and equilibration, the Vanquish Neo system settings were: 5% B, fast equilibration enabled, equilibration factor set to 3; for the trap column: fast wash, equilibration, and zebra wash enabled (two zebra wash cycles, automatic equilibration factor). The Orbitrap Eclipse mass spectrometer operated in data-dependent acquisition (DDA) mode with internal real-time mass calibration using a user-defined lock mass (m/z = 445.12003, positive). Full MS scans in the orbitrap covered a range of 300-1500 m/z at a resolution of 120,000 with an AGC target of 4e5 and maximum injection time set to auto. The top 10 intense ions (charge states 2-7) were selected for MS2 scans with a minimum intensity threshold of 5.0e3, isotope exclusion, and dynamic exclusion (30 s exclusion duration). Peaks with unassigned charges or a charge of +1 were excluded. MS2 spectra were collected at a resolution of 15,000 with an AGC target of 5e4. The maximum injection time was kept in the default setting. Isolation in the quadrupole was performed with a 1.6 m/z window. Fragments were generated using higher-energy collision-induced dissociation (HCD) with a normalized collision energy of 30% and detected in the orbitrap. Data acquisition was conducted using Thermo Scientific Foundation software version 3.1sp9 and Xcalibur version 4.6. IsoDTB samples were performed in n = 4 independent replicates.

Data analysis of Orbitrap Eclipse measurements.

The data analysis was performed as previously described[7, 31] and adjusted for binding site identification studies. Results and filtered results after performing open and modified closed searches are summarized and accessible in the PRIDE[1] partner repository with the dataset identifier PXD051986.

General setup of analysis software

The raw data from LC-MS/MS analyses were transformed into mzML format using the MSconvert tool (version 3.0.21193-ccb3e0136) from ProteoWizard software (version 3.0.21193, 64-bit) with default settings and vendor peak picking enabled. For subsequent data analysis, the FragPipe interface (version 20.0) was employed, incorporating MSFragger (version 3.8), Philosopher (version 5.0.0), IonQuant (version 1.9.8), and Python (version 3.7.3). The UniProt reference proteome for S.

aureus NCTC8325 (taxon identifier: 93061, downloaded on 22.08.2023, with an annotation error in SpsB, Q2FZT7 missing amino acids 1-36) was utilized. Reverse sequences were manually appended to the FASTA databases.

Open Search Analysis of mass of modifications with FragPipe[13-17, 32, 33]

The observed mass shifts in the peptides of MBP-SpsB were analyzed using an *Open Search* with MSFragger.[13-17, 32, 33] The following parameters were applied: precursor mass tolerance ranged from -150 to 1,000 Da, initial fragment mass tolerance was set to 20 ppm, and both mass calibration and parameter optimization were enabled. The isotope error was set to '0', and the enzyme was specified as trypsin with cleavage occurring after 'KR' but not before 'P'. Enzymatic cleavage allowed for up to two missed cleavages, and N-terminal clipping was enabled. Peptide lengths ranged from 7 to 50 amino acids, and peptide masses ranged from 500 to 5,000 Da, with no variable or fixed modifications. Other settings were kept at their default values. Crystal-C14 was enabled.[14] PeptideProphet[32] was executed with the following parameters: '--nonparam --expectscore --decoyprobs --masswidth 1,000.0 --clevel -2'. PTMProphet was not used. ProteinProphet[32] was run with the setting '--maxppmdiff 2,000,000'. Report generation was enabled with the options '--sequential --razor –mapmods --prot 0.01'; MS1 quantification and TMT-integrator were disabled. PTM-Shepherd[16] was activated with these settings: smoothing factor '2', precursor tolerance '0.01 Da', prominence ratio '0.3', peak picking width '0.002 Da', localization background '4', and annotation tolerance '0.01 Da'. A custom mass shift list was utilized, including only UniMod modifications with molecular weights less than 400 Da, as previously published. [31] Ion types for modifications were set to 'b' and 'y', with mass fragment charge set to '2'. The generation of a spectral library was disabled. For downstream data analysis, the 'global.modsummary.tsv' file was searched for mass shifts greater than 482 Da (with the exact masses of light tag: 482.2834 Da and heavy tag: 488.2909 Da) and mass shift differences of 6.0075 ± 0.0010 Da between heavy and light isoDTB tags linked to **L15-P**. The filtered results are summarized in **Table S9**.

Modified Closed Search Analysis for Binding site identification studies with FragPipe

A modified *Closed Search* was conducted to pinpoint the binding site of **L15-P** using MSFragger with the following parameters: precursor mass tolerance of -20 to 20 ppm, fragment mass tolerance of 20 ppm, and enabled mass calibration and parameter optimization. The isotope error settings were configured to '0/1/2'. Trypsin was selected as the enzyme, and cleavage was designated to occur after 'K' but not before 'P', allowing for up to two missed cleavages. N-terminal clipping was enabled. Peptide lengths were set to range from 7 to 50 amino acids, and peptide masses ranged from 500 to 5,000 Da, with no mass offsets. Other settings were maintained at their default values. Fixed and variable modifications were assigned to the masses 832.436 and 838.4426, corresponding to the **L15-P**-isoDTB tag adducts identified in the Open Search. Crystal-C was disabled. PeptideProphet was executed with the following parameters: '--decoyprobs --ppm --accmass --nonparam –expectscore'. PTMProphet was not utilized. ProteinProphet was run with the setting '--maxppmdiff 2,000,000'. Report generation was activated with '--sequential --prot 0.01'. PTM-Shepherd was disabled. MS1 quantification was performed with IonQuant enabled, including MaxLFQ with a minimum of 2 ions. Labeling-based quantification was done using detected masses from Table **S10** for all amino acids (*), with re-quantify enabled, the top 3 ions considered, a minimum frequency of 0.5, at least 1 scan, and a minimum of 2 isotopes. Normalization was disabled, the retention time (RT) window was set to 0.4 minutes, and the m/z window was set to 10 ppm. TMT-Integrator was disabled. Spectral library generation was not conducted. For the

quadruplicate runs, each was analyzed as a separate experiment. The 'ion_label_quant.tsv' files from the four experiments were analyzed individually. For each entry, the 'Modified Peptide' was determined as either the 'Light Modified Peptide' or the 'Heavy Modified Peptide' based on the higher 'PeptideProphet Probability'. The modification masses in the 'Modified Peptide' were replaced with '*', and any carbamidomethylation mass (57.0215 Da) was removed. The complete protein sequence was linked to the table. Peptide sequences that did not uniquely occur in the same protein were excluded, and the modified residue's position was determined. The 'Identifier' was formatted as 'UniProtCode'_*residue number, where * represents the modified amino acid's one-letter code. The average 'Log2 Ratio HL', representing the log2-transformed ratio of heavy and light ions, was calculated as the weighted average of all corresponding ions' 'Log2 Ratio HL' values, weighted by the ion's 'Total Intensity'. This value was excluded if the standard deviation of 'Log2 Ratio HL' values exceeded 1.41 for all ions of the same 'Identifier'. For each 'Identifier', 'Total Intensity', 'Total Light Intensity', and 'Total Heavy Intensity' were summed across all individual ions. If multiple 'Modified Peptides' were found for the same 'Identifier', the shortest sequence was retained. Data for all four replicates were compiled into one table. The 'Log2 Ratio HL' values for the replicates were labeled as 'Log2 R Replicate 1', 'Log2 R Replicate 2', 'Log2 R Replicate 3', and 'Log2 R Replicate 4'. Their average was calculated as 'Log2 R Average', excluding values where the standard deviation between replicates exceeded 1.41. Values were retained if the identifier was quantified in at least one replicate. The peptide sequence consistently modified across all four replicates was designated as the **L15-P** binding site to SpsB (Table **S12**).

Preparation of Membrane Fractions from *E. coli*

E. *coli* membrane fractions harboring overexpressed *S. aureus* SpsB were prepared as previously described.[18, 34]

E. coli BL21(DE3) pLysS cells transformed with pET-55-DEST-fl-SpsB or respective mutant plasmids (F67A, Y75A, F158A or Q165A) were grown in LB medium supplemented with 100 µg/mL ampicillin and 34 µg/mL chloramphenicol at 200 rpm and 37°C until they reached OD_{600} = 0.6. Subsequently, protein overexpression was induced with 0.5 mM isopropyl-1-thio-β-galactopyranoside (IPTG) and cells were further incubated for 3 h at 200 rpm and 22 °C. Cells were harvested by centrifugation for 10 min at 6000 x *g* and 4 °C. The cell pellet was washed with PBS and resuspended in 5 mL 50 mM Tris-HCl buffer pH = 7.5. Lysis was done with a bead beater homogenizer (Precellys Ceramic Kit CK01L, 7.0 mL tubes; Precellys 24 Homogenizer, *Bertin Technologies*) in three cycles: 45 s at 5,500 rpm, followed by cooling for 30 s. In order to remove cell debris, the lysate was centrifuged for 10 min at 12,000 x *g* and 4 °C. The supernatant was then centrifuged for 75 min at 39,000 x *g* and 4 °C to collect the cell membranes. Membranes were resuspended in 50 mM ice-cold sodium phosphate buffer pH 7.5. The protein concentration was determined using the Pierce BCA Protein assay kit (Roti Quant, *Roth*). Membranes were snap-frozen in liquid nitrogen and stored at -80 °C for further usage.

For preparation of the respective *E. coli* membrane control fractions, the same procedure was performed without IPTG induction.

FRET-based SpsB assay with membrane-bound SpsB

SpsB activity of overexpressed full-length *S. aureus* SpsB or mutants (F67A, Y75A, F158A or Q165A) in *E. coli* membranes were measured by Förster Resonance Energy Transfer (FRET) assay as previously described.[18, 35]

FRET assays were performed with overexpressed wildtype SpsB (wt) or mutant (F67A, Y75A, F158A or Q165A) in *E. coli* membranes. Background activity was measured with control membranes without IPTG induction. A synthetic peptide based on SceD (DABCYL-AGHDAHASET-EDANS, #AS-64916, *AnaSpec*) was utilized as a FRET substrate. This peptide was modified with 4-(4-dimethylaminophenylazo)benzoic (DABCYL) acid and 5-((2-aminoethyl)amino)-1-naphthalenesulfonic (EDANS) acid. 100 µL of 50 µg/mL respective membranes in 50 mM sodium phosphate buffer pH 7.5 were treated with each compound or DMSO as a control for 5 min at 37 °C. Detergents were added to the sodium phosphate buffer if needed for the experiment: 0.1% CHAPS, 0.001% NP-40 or 0.1% Tween. 1 µL of 10 mM FRET substrate in DMF was added to each sample, and fluorescence was recorded with an Infinite™ M Nano Tecan 200Pro plate reader at λ_{ex} = 340 nm and λ_{em} = 510 nm for at least 2 h at 37 °C. Background activity of non-induced *E. coli* membranes was subtracted, and the initial substrate cleavage velocities within the first 1,000 s were determined via simple linear regression using *GraphPad* Prism 10.01. Each sample was normalized to DMSO, which is referred to as 100% of substrate cleavage. Each measurement was performed in n = 3 biologically independent replicates per condition with three technical replicates each. Statistical significance was determined using ordinary one-way ANOVA.

Molecular dynamics simulations

The ternary complexes of the SpsB-substrate-**L15** and SpsB-substrate-**D13** were generated by docking **L15** or **D13** to the substrate-bound SpsB modeled as described in Chen *et al*.[7] using Autodock vina[36]. For further simulations and analysis best-scored binding poses were selected. The two ternary complexes were embedded in dimyristoyl phosphatidylglycerol bilayer and solvated in water and 0.15 M NaCl using CHARMM-GUI online server[37]. Interactions between atoms were described by ff19SB[38] for proteins, lipid21[39] for membrane, GAFF2[40] for **L15** and **D13**, and OPC[41] for water molecules. Point charges of **L15** and **D13** were assigned according to the AM1-BCC charging method[42] implemented in antechamber[43]. For each ternary complex five simulations of each 1 µs with randomly assigned initial atomic velocities were generated followed by energy minimization and equilibration as described previously[7] using cuda-accelerated PMEMD in Amber 22 package[44]. Langevin thermostat[45] and Berendsen barostat[46] were used to maintain the temperature and pressure at 303.15 K and 1 bar, respectively. A time step of 4 fs was allowed with the SHAKE algorithm[47] and hydrogen mass repartitioning[48]. Water accessibilities and geometric measurements between the atoms were calculated using PYTRAJ and CPPTRAJ[49] and the data of **PK150**-bound simulations are retrieved from a previous study.[7]

Chemical Synthesis

General Remarks

Air and moisture sensitive reactions were handled under argon atmosphere using standard Schlenk technique. All chemicals and solvents were obtained from commercial suppliers, stored as indicated and used without further purification.

Analytical Methods

Thin Layer and Column Chromatography

Retention factors (R_f) were determined by thinlayer chromatography (TLC) on aluminium plates with silica coating (*Merck*, silica 60 F254). Detection was done either by UV (λ = 254 nm or λ = 266 nm) or by staining with ninhydrin solution [ninhydrin (16.8 mM) in *n*-butanol with acetic acid (5v/v%)], dinitrophenylhydrazine (DNP) stain [DNP (20.5 mM), conc. sulfuric acid (17.5 v/v%), water (23.5 v/v%) in ethanol] or potassium permanganate ($KMnO_4$) stain [K_2CO_3 (360 mM), $KMnO_4$ (47.5 mM) in NaOH (18.75 mM)] and subsequent heating. Silica gel (*Merck*, particle size 40-63 µm) was used as the stationary phase for preparative column chromatography.

High-Resolution Mass Spectrometry (HR-MS)

High-resolution mass spectra were recorded on an LTQ-Orbitrap XL or QExactive Plus mass spectrometer (Thermo Fisher Scientific) coupled to a Dionex Ultimate 3000 system (Thermo Fisher Scientific) using a positive electron spray ionization (ESI) mode. Mass spectrometry data were processed using Xcalibur 2.2 software (*Thermo Fisher Scientific*).

High-Performance Liquid Chromatography (HPLC)

Preparative, reversed-phase HPLC was performed for compound purification using a *Waters* 2545 quaternary gradient module with a *Waters* 2998 photodiode array detector and fraction collector. Compound separation was achieved using a *YMC* Triart C18 column (250 × 10 mm, 3.5 µm particle size) at a flow rate of 10 mL/min (column 1) or a *Waters* XBridge C18 column (150 × 30 mm, 5 µm particle size) at a flow rate of 50 mL/min (column 2). A gradient of water containing trifluoroacetic acid (0.1 v/v%) (A) and HPLC-grade acetonitrile containing trifluoroacetic acid (0.1 v/v%) (B) was used as mobile phase and method parameters as shown in **table S13.**

Table S13. The HPLC gradient parameters for compound separation. A = water/ trifluoroacetic acid (0.1 v/v%), B = acetonitrile/ trifluoroacetic acid (0.1 v/v%), compositions are given in [v/v%].

t [min]	Method																	
	1		2		3		4		5		6		7		8		9	
	A	B	A	B	A	B	A	B	A	B	A	B	A	B	A	B	A	B
0	98	2	98	2	98	2	70	30	80	20	98	2	80	20	98	2	98	2
1	80	20																
2															90	10		
3					85	15												
12			50	50											65	35		
14			2	98											2	98		
15	45	55																
16			2	98											2	98	80	20
17	2	98	98	2														
18															98	2	2	98

19	2	98	98	2														
20	98	2									60	40			98	2	2	98
21											2	98						
22	98	2															98	2
23					65	35					2	98						
24																	98	2
25					2	98	50	50	45	55	98	2						
26							2	98										
27					2	98			2	98	98	2						
28							2	98										
29					98	2			2	98								
30							98	2					50	50				
31					98	2			98	2								
32							98	2					2	98				
33									98	2								
34													2	98				
36													98	2				
38													98	2				

Nuclear Magnetic Resonance Spectroscopy (NMR). The nuclear magnetic resonance spectra were recorded on AVHD 300, AVHD 400, AVHD 500, and AV 500-cryo spectrometers (*Bruker*) at 25 °C. Spectra evaluation was performed using the program MestReNova x64 (*Mestrelab Research*). The spectroscopic data are given as follows: chemical shift (multiplicity, coupling constants, integral). The chemical shift δ is given in *parts per million* [ppm], referring to the residual proton signal of the deuterated solvent used as reference. In the ^{1}H spectra, the residual proton signal of dimethyl sulfoxide-d_6 (δ = 2.50 ppm), chloroform-d (δ = 7.26 ppm), or deuterium oxide (D2O, (δ = 4.79 ppm), were used as reference.[50] Dimethyl sulfoxide-d_6 (δ = 39.52 ppm) and chloroform-d (δ = 77.16 ppm) were referenced in the ^{13}C spectra via the ^{13}C-D coupling.[50] ^{19}F spectra are given without reference. Abbreviations used to indicate multiplicity are defined as follows: s - singlet, d - doublet, t - triplet, q - quartet, quin - quintet, sept – septet, m - multiplet, b - broad. The coupling constants *J* are reported in *Hertz* [Hz] and given as the average of the experimentally determined coupling and back couplings.

General Procedures

General Procedure I: Radical Arylation. According to a modified literature procedure[51], a solution of 4-bromoaniline (1.00 eq.) in water (667 mM) and hydrochloric acid (aqueous, 2 M, 3.00 eq.) was degassed by bubbling argon through the solution and cooled down to 0 °C. A sodium nitrite solution (aqueous, 2 M, 1.00 eq.) was added dropwise over 10 min, and the reaction mixture was stirred for one hour at 0 °C. The obtained diazonium salt solution was added dropwise over 15 min to a solution of furfurylamine (5.00 eq.) and titanium(III)chloride (1 M in 3 M in hydrochloric acid, 2.00 eq.) in water (6.67 M) and hydrochloric acid (aqueous, 2 M, 3.75 eq.). The reaction mixture was allowed to warm up to RT and stirred overnight. Subsequently, sodium carbonate solution (aqueous, saturated) and sodium sulfite solution (aqueous, saturated) were added until pH 9 was reached, the reaction mixture was filtered over celite, and the aqueous phase was extracted with ethyl acetate (3 ×). The organic phase was washed with sodium chloride solution (aqueous, saturated, 1 ×) and filtered through hydrophobic filter paper or dried over sodium sulfate. Afterwards, the solvent was evaporated, and

the product was purified via column chromatography.
Note: The products of this reaction are prone to oxidation.

General Procedure II: Guanidinylation. According to a modified literature procedure[52], the amine (1.00 eq.) was dissolved in acetonitrile (1 M) before *N*, *N*-diisopropyl-ethylamine (1.05 eq.) and 1*H*-pyrazole-1-carboxamidin hydrochloride (1.00 eq.) were added. The flask was flushed with argon, and the reaction mixture was stirred overnight at RT. The product was obtained after solvent evaporation and preparative HPLC.

General Procedure III: Oxime Formation. The aldehyde (1.00 eq.), hydroxylamine hydrochloride (2.00 eq.), and pyridine (2.00 eq.) were dissolved in ethanol (200 mM). The reaction mixture was stirred for 10 min at RT and heated to 80 °C for 2.5 to 3 h. After complete conversion, the solvent was evaporated, and the crude product was directly used in the following reaction without further purification and characterization.

General Procedure IV: Oxime Reduction. The oxime (1.00 eq.) was dissolved in acetic acid (460-650 mM), and zinc (4.00 eq.) was added. The reaction mixture was stirred overnight at RT, filtered over celite, and washed with dichloromethane or ethyl acetate (2 ×). The phases were separated. The aqueous phase was basified with sodium carbonate solution (aqueous, saturated) up to pH 8-9 and extracted with dichloromethane or ethyl acetate (3 ×), washed with brine (1 ×) and dried with sodium sulfate. The amine was obtained after solvent evaporation and purification via column chromatography.

Derivative Synthesis

(5-(4-Bromophenyl)furan-2-yl)methanamine (1)

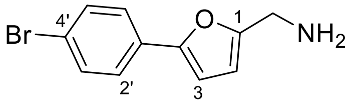

$C_{11}H_{10}BrNO$
252.11 g/mol
1

The general procedure I was followed using 4-bromoaniline (200 mg, 1.16 mmol, 1.00 eq.) and furfurylamine (777 mg, 706 µL, 8.00 mmol, 6.90 eq.) as described. The amine **1** was obtained after purification via column chromatography (DCM/EtOH = 96/4 with 1 v/v% triethylamine (TEA)) as a light orange oil (106 mg, 421 µmol, 36%).

TLC: R_f = 0.14 (DCM/EtOH= 96/4 with 1 v/v% TEA) [UV].

^{1}H-NMR (400 MHz, DMSO-d$_6$): δ [ppm] = 3.72 (s, 2 H), 6.33 (d, ^3J = 3.3 Hz, 1 H), 6.91 (d, ^3J = 3.3 Hz, 1 H), 7.58 (dq$_{A\text{-}B}$, ^3J = 8.6 Hz, $J_{A\text{-}B}$ = 4.2 Hz, 1 H), 7.62 (dq$_{A\text{-}B}$, ^3J = 8.6 Hz, $J_{A\text{-}B}$ = 4.2 Hz, 1 H).

^{13}C{^{1}H}-NMR (125 MHz, DMSO-d$_6$): [ppm] = 38.8 (s), 107.4 (s), 107.7 (s), 119.9 (s), 125.1 (s,), 129.8 (s), 131.8 (s), 150.5 (s), 157.8 (s).

HR-MS (ESI): calc. for $[C_{11}H_8{}^{79}BrO]^+$ ($[M\text{-}NH_2]^+$): 234.9759, found 234.9756; calc. for $[C_{11}H_8{}^{81}BrO]^+$ ($[M\text{-}NH_2]^+$): 236.9738, found 236.9732.

Analytical data are in accordance with the literature.[53]

Amino(((5-(4-bromophenyl)furan-2-yl)methyl)amino)methaniminium 2,2,2-trifluoro-acetate (L15)

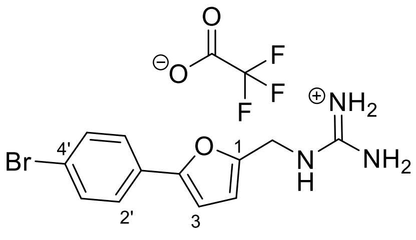

$C_{14}H_{13}BrF_3N_3O_3$
408.18 g/mol
L15

The general procedure II was followed using amine **1** (100 mg, 397 µmol, 1.05 eq.) as described. The guanidine **L15** was obtained after purification via preparative HPLC (column 2, method 1, λ = 290 nm) as a light yellow solid (78.6 mg, 193 µmol, 51%).

^{1}H-NMR (400 MHz, DMSO-d$_6$): δ [ppm] = 4.45 (d, ^3J = 6.0 Hz, 2 H), 6.51 (d, ^3J = 3.4 Hz, 1 H), 7.00 (d, ^3J = 3.4 Hz, 1 H), 7.61-7.66 (m, 4 H), 7.95 (t, ^3J = 6.0 Hz, 1 H).

^{13}C{^{1}H}NMR (125 MHz, DMSO-d$_6$): δ [ppm] = 37.7 (s), 107.5 (s), 110.4 (s), 120.6 (s), 125.3 (s), 129.3 (s), 131.9 (s), 150.5 (s), 151.8 (s), 156.7 (s).
^{19}F-NMR (375 MHz, DMSO-d$_6$): δ [ppm] = - 73.9 (s).

HR-MS (ESI): calc. for $[C_{11}H_8{}^{79}BrO]^+$ ($[M-CH_5N_3]^+$): 234.9759, found 234.9756; calc. for $[C_{11}H_8{}^{79}BrO]^+$ ($[M-CH_5N_3]^+$): 236.9738, found 236.9731.

(5-(3-Bromophenyl)furan-2-yl)methanamine (2)

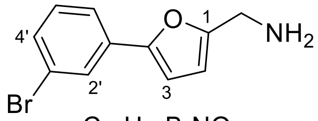

$C_{11}H_{10}BrNO$
252.11 g/mol
2

The general procedure I was followed using 3-bromoaniline (275 mg, 174 mL, 1.60 mmol, 1.00 eq.) and furfurylamine (777 mg, 706 µL, 8.00 mmol, 5.00 eq.) as described. The amine **2** was obtained after purification via column chromatography (DCM/MeOH= 9/1 with 0.1 v/v% TEA) as a light orange oil (116 mg, 461 mmol, 29%).

TLC: R_f = 0.29 (DCM/MeOH = 9/1) [UV].

^{1}H-NMR (500 MHz, DMSO-d$_6$): δ [ppm] = 3.73 (s, 2 H), 6.34 (dt, ^3J = 3.3 Hz, ^4J = 0.8 Hz, 1 H), 6.98 (d, ^3J = 3.3 Hz, 1 H), 7.36 (*virt.* t, $^3J \approx {}^3J$ = 7.9 Hz, 1 H), 7.44 (ddd, ^3J = 7.9 Hz, ^4J = 2.0, 1.4 Hz, 1 H), 7.67 (*virt.* dt, ^3J = 7.9 Hz, $^4J \approx {}^4J$ = 1.4 Hz, 1 H), 7.86 (*virt*. t, $^4J \approx {}^4J$ = 1.4 Hz, 1 H).

^{13}C{^{1}H}NMR (100 MHz, DMSO-d$_6$): δ [ppm] = 38.8 (s), 107.6 (s), 108.0 (s), 122.0 (s), 122.3 (s), 125.4 (s), 129.6 (s), 131.0 (s), 132.8 (s), 149.8 (s), 158.3 (s).

HR-MS (ESI): calc. for $[C_{11}H_8{}^{79}BrO]$ ($[M-NH_2]^+$): 234.9759, found 234.9754; calc. for $[C_{11}H_8\,{}^{81}BrO]$($[M-NH_2]^+$): 236.9738, found 236.9730.

Amino(((5-(3-bromophenyl)furan-2-yl)methyl)amino)methaniminium 2,2,2-trifluoro-acetate (D01)

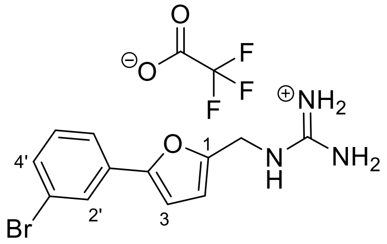

$C_{14}H_{13}BrF_3N_3O_3$
408.18 g/mol
D01

The general procedure II was followed using amine **2** (67.0 mg, 266 µmol, 1.05 eq.) as described. The crude product was dissolved in a minimal amount of methanol and precipitated by adding an excess of diethyl ether. The guanidine **D01** was obtained after purification via preparative HPLC (column 1, method 2, λ = 286 nm) as an off-white solid (74.4 mg, 182 µmol, 72%).

^{1}H-NMR (500 MHz, DMSO-d$_6$): δ [ppm] = 4.31 (s, 2 H), 6.36 (d, ^3J = 3.4 Hz, 1 H), 6.69 (d, ^3J = 3.4 Hz, 1 H), 7.20 (*virt.* t, $^3J \approx ^3J$ = 8.0 Hz, 1 H), 7.34 (d, ^3J = 8.0 Hz, 1 H), 7.53 (d, ^3J = 8.0 Hz, 1 H), 7.76 (bs, 1 H).

^{13}C{^{1}H}NMR (125 MHz, DMSO-d$_6$): δ [ppm] = 109.1 (s), 112.2 (s), 124.0 (s), 124.2 (s), 127.9 (s), 132.2 (s), 132.5 (s), 133.7 (s), 151.5 (s), 153.7 (s), 158.4 (s). The ^{13}C signal of the CH_2 group overlaps with the solvent signal and can, therefore, not be localized.

^{19}F-NMR (375 MHz, D_2O): δ [ppm] = - 75.3 (s).

HR-MS (ESI): calc. for $[C_{11}H_8{}^{79}BrO]^+$ ($[M\text{-}CH_5N_3]^+$): 234.9759, found 234.9754; calc. $[C_{11}H_8{}^{81}BrO]^+$ ($[M\text{-}CH_5N_3]^+$): 236.9738, found 236.9729; calc. for $[C_{12}H_{13}{}^{79}BrN_3O]^+$ ($[M+H]^+$): 294.0237, found 294.1235; calc. for $[C_{12}H_{13}{}^{81}BrN_3O]^+$ ($[M+H]^+$): 296.0216, found 296.0213.

(5-(2-Bromophenyl)furan-2-yl)methanamine (3)

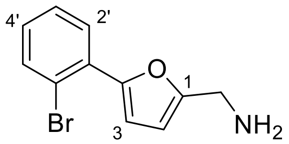

$C_{11}H_{10}BrNO$
252.11 g/mol
3

The general procedure I was followed using 2-bromoaniline (275 mg, 181 mL, 1.60 mmol, 1.00 eq.) and furfurylamine (777 mg, 706 µL, 8.00 mmol, 5.00 eq.) as described. The amine **3** was obtained after purification via column chromatography (DCM/MeOH= 98/2 with 0.1 v/v% TEA) as an orange oil (214 mg, 848 mmol, 53%).

TLC: R_f = 0.32 (DCM/MeOH = 98/2 with 0.1 v/v% TEA) [UV].

^{1}H-NMR (500 MHz, DMSO-d$_6$): δ [ppm] = 3.75 (s, 2 H), 6.39 (d, ^3J = 3.3 Hz, 1 H), 7.08 (d, ^3J = 3.3 Hz, 1 H), 7.23 (*virt.* td, $^3J \approx ^3J$ = 7.8 Hz, ^4J = 1.5 Hz, 1 H), 7.46 (*virt.* td, $^3J \approx ^3J$ = 7.8 Hz, ^4J = 1.0 Hz, 1 H), 7.71 (dd, ^3J = 7.8 Hz, ^4J = 1.0 Hz, 1 H), 7.79 (dd, ^3J = 7.8 Hz, ^4J = 1.5 Hz, 1 H).

^{13}C{^{1}H}NMR (125 MHz, DMSO-d$_6$): δ [ppm] = 38.8 (s), 107.3 (s), 111.5 (s), 118.5 (s), 128.1 (s), 128.5 (s), 128.9 (s), 130.6 (s), 134.1 (s), 148.9 (s), 157.8 (s).

HR-MS (ESI): calc. for $[C_{11}H_8{}^{79}BrO]$ ($[M{-}NH_2]^+$): 234.9759, found 234.9752; calc. for $[C_{11}H_8{}^{81}BrO]$ ($[M{-}NH_2]^+$): 236.9730, found 236.9728.

Amino(((5-(2-bromophenyl)furan-2-yl)methyl)amino)methaniminium 2,2,2-trifluoro-acetate (D02)

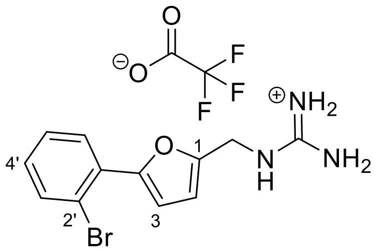

$C_{14}H_{13}BrF_3N_3O_3$
408.18 g/mol
D02

The general procedure II was followed using amine **3** (106 mg, 421 µmol, 1.05 eq.) as described. The crude product was dissolved in a minimal amount of methanol and precipitated by adding an excess of diethyl ether. The guanidine **D03** was obtained after purification via preparative HPLC (column 2, method 1, λ = 284 nm) as a brownish solid (111 mg, 272 µmol, 68%).

^{1}H-NMR (500 MHz, DMSO-d$_6$): δ [ppm] = 4.48 (d, ^3J = 6.0 Hz, 2 H), 6.56 (d, ^3J = 3.4 Hz, 1 H), 7.11 (d, ^3J = 3.4 Hz, 1 H), 7.28 (ddd, ^3J = 7.9 Hz, ^3J = 7.5 Hz, ^4J = 1.7 Hz, 1 H), 7.49 (*virt.* dt, $^3J \approx {}^3J$ = 7.8 Hz, ^4J = 1.2 Hz, 1 H), 7.75 (dd, ^3J = 8.1 Hz, ^4J = 1.2 Hz, 1 H), 7.75 (dd, ^3J = 7.8 Hz, ^4J = 1.7 Hz, 1 H), 7.96 (t, ^3J = 6.0 Hz, 1 H).

^{13}C{^{1}H}NMR (125 MHz, DMSO-d$_6$): δ [ppm] = 37.7 (s), 109.9 (s), 111.5 (s), 118.8 (s), 128.1 (s), 128.7 (s), 129.5 (s), 130.2 (s), 134.2 (s), 150.3 (s), 150.5 (s), 156.8 (s).

^{19}F-NMR (375 MHz, DMSO-d$_6$): δ [ppm] = - 73.4 (bs).

HR-MS (ESI): calc. for $[C_{11}H_8{}^{79}BrO]^+$ ($[M-CH_5N_3]^+$): 234.9754, found 234.9760; calc. for $[C_{11}H_8{}^{81}BrO]^+$ ($[M-CH_5N_3]^+$): 236.9729, found 236.9734; calc. for $[C_{12}H_{13}{}^{79}BrN_3O]^+$ ($[M+H]^+$): 294.0237, found 294.1242; calc. for $[C_{12}H_{13}{}^{81}BrN_3O]^+$ ($[M+H]^+$): 296.0216, found 296.0220.

(5-(4-Fluorophenyl)furan-2-yl)methanamine (4)

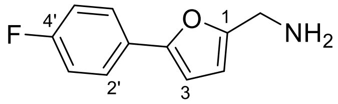

$C_{11}H_{10}FNO$
191,21 g/mol
4

The general procedure I was followed using 4-fluoroaniline (178 mg, 155 mL, 1.60 mmol, 1.00 eq.) and furfurylamine (563 mg, 512 µL, 5.80 mmol, 5.00 eq.) as described. The amine **4** was obtained after purification via column chromatography (DCM/MeOH= 95/5) as an orange solid (81.9 mg, 428 mmol, 27%).

TLC: R_f = 0.17 (DCM/MeOH= 95/5) [UV].

^{1}H-NMR (500 MHz, DMSO-d$_6$): δ [ppm] = 3.72 (s, 2 H), 6.32 (d, ^3J = 3.3 Hz, 1 H), 6.83 (d, ^3J = 3.3 Hz, 1 H), 7.21-7.27 (m, 1 H), 7.68-7.72 (m, 1 H).

^{13}C{^{1}H}NMR (125 MHz, DMSO-d$_6$): δ [ppm] = 38.7 (s), 106.3 (s), 107.6 (s), 115.8 (d, $^2J_{C-F}$ = 22.8 Hz), 125.2 (d, $^3J_{C-F}$ = 8.0 Hz), 127.3 (d, $^4J_{C-F}$ = 3.2 Hz), 150.8 (s), 157.0 (s), 161.2 (d, $^1J_{C-F}$ = 244 Hz).

^{19}F-NMR (375 MHz, DMSO-d$_6$): δ [ppm] = - 114.9 (*virt.* sept, J_{H-F} = 5.3 Hz).

HR-MS (ESI): calc. for $[C_{11}H_8FO]^+$ ($[M-NH_2]^+$): 175.0559, found 175.0553.

Analytical data are in accordance with the literature.[54]

Amino(((5-(4-fluorophenyl)furan-2-yl)methyl)amino)methaniminium 2,2,2-trifluoroacetate (D03)

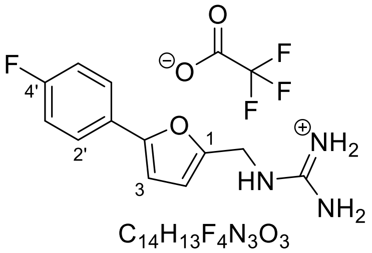

$C_{14}H_{13}F_4N_3O_3$
347.27 g/mol
D03

The general procedure II was followed using amine **4** (50.0 mg, 262 µmol, 1.05 eq.) as described. The crude product was dissolved in a minimal amount of methanol and precipitated by adding excess ofdiethyl ether. The guanidine **D03** was obtained after purification via preparative HPLC (column 1, method 1, λ = 281 nm) as an off-white solid (62.8 mg, 181 µmol, 73%).

^{1}H-NMR (500 MHz, DMSO-d$_6$): *δ* [ppm] = 4.45 (d, ^3J = 5.9 Hz, 2 H), 6.49 (d, ^3J = 3.3 Hz, 1 H), 6.90 (d, ^3J = 3.3 Hz, 1 H), 7.25-7.31 (m, 1 H), 7.69-7.75 (m, 1 H), 7.97 (bs, 1 H).

^{13}C{^{1}H}-NMR (125 MHz, DMSO-d$_6$): *δ* [ppm] = 107.6 (s), 112.2 (s), 117.6 (d, $^2J_{C\text{-}F}$ = 22.2 Hz), 127.2 (d, $^3J_{C\text{-}F}$ = 8.0 Hz), 128.2 (d, $^4J_{C\text{-}F}$ = 3.6 Hz), 150.8, (s), 154.5 (s), 158.3 (s), 163.7 (d, $^1J_{C\text{-}F}$ = 246.3 Hz). The ^{13}C signal of the CH_2 group overlaps with the solvent signal and can, therefore, not be localized.

^{19}F-NMR (375 MHz, D_2O: *δ* [ppm] = - 75.2 (s), - 113.8 (bs).

HR-MS (ESI): calc. for $[C_{11}H_8FO]^+$ ($[M\text{-}CH_5N_3]^+$): 175.0559, found 175.0553.

5-Phenylfuran-2-yl)methanamine (5)

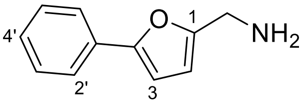

$C_{11}H_{11}NO$
173,22 g/mol
5

The general procedure I was followed using aniline (149 mg, 146 mL, 1.60 mmol, 1.00 eq.) and furfurylamine (777 mg, 706 µL, 8.00 mmol, 5.00 eq.) as described. The amine **5** was obtained after purification via column chromatography (DCM/MeOH = 95/5) as an orange solid (76.2 mg, 440 mmol, 27%).

TLC: R_f = 0.11 (DCM/MeOH = 95/5) [UV].

^{1}H-NMR (500 MHz, DMSO-d$_6$): *δ* [ppm] = 3.75 (s, 2 H), 6.33 (d, ^3J = 3.3 Hz, 1 H), 6.85 (d, ^3J = 3.3 Hz, 1 H), 7.26 (tt, ^3J = 7.5 Hz, ^4J = 1.3 Hz, 1 H), 7.40 (*virt.* t, $^3J \approx {}^3J$ = 7.7 Hz, 2 H), 7.67 (dd, ^3J = 8.4 Hz, ^4J = 1.3 Hz, 2 H).

HR-MS (ESI): calc. for $[C_{11}H_9O]^+$ ($[M\text{-}NH_2]^+$): 157.0653, found 157.0647.

Complete characterization could not be performed due to the oxidation sensitivity of the product. Analytical data are in accordance with the literature.[55]

Amino(((5-phenylfuran-2-yl)methyl)amino)methaniminium 2,2,2-trifluoroacetate (D04)

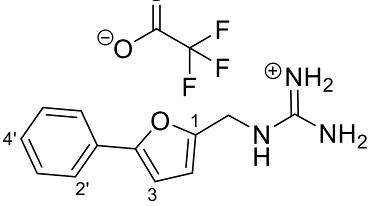

$C_{14}H_{14}F_3N_3O_3$
329.28 g/mol
D04

The general procedure II was followed using amine **5** (55.4 mg, 320 µmol, 1.05 eq.) as described. The crude product was dissolved in a minimal amount of methanol and precipitated by adding an excess of diethyl ether. The guanidine **D04** was obtained after purification via preparative HPLC (column 1, method 1, λ = 284 nm) as a brown oil (55.9 mg, 170 µmol, 56%).

^{1}H-NMR (500 MHz, DMSO-d$_6$): *δ* [ppm] = 4.46 (d, ^3J = 6.0 Hz, 2 H), 6.50 (d, ^3J = 3.3 Hz, 1 H), 6.92 (d, ^3J = 3.3 Hz, 1 H), 7.30 (tt,

^{3}J = 7.4 Hz, ^{4}J = 1.2 Hz, 1 H), 7.43 (*virt.* t, $^{3}J \approx {}^{3}J$ = 7.5 Hz, 2 H), 7.69 (dd, ^{3}J = 8.3 Hz, ^{4}J = 1.2 Hz, 2 H), 7.93 (t, ^{3}J = 6.0 Hz, 1 H).

^{13}C{^{1}H}NMR (125 MHz, DMSO-d$_6$): δ [ppm] = 37.8 (s), 106.6 (s), 110.3 (s), 123.3 (s), 127.7 (s), 129.0 (s), 130.1 (s), 150.0 (s), 152.9 (s), 156.7 (s).

^{19}F-NMR (375 MHz, DMSO-d$_6$): δ [ppm] = - 73.7 (s).

HR-MS (ESI): calc. for $[C_{11}H_9O]^+$ ($[M\text{-}CH_5N_3]^+$): 157.0653, found 157.0647.

(5-(4-Isopropylphenyl)furan-2-yl)methanamine (6)

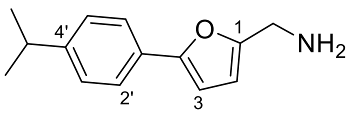

$C_{14}H_{17}NO$
215.30 g/mol
6

The general procedure I was followed using 4-isopropylaniline (270 mg, 285 µL, 2.00 mmol, 1.00 eq.) and furfurylamine (991 mg, 944 µL, 10.0 mmol, 5.00 eq.) as described. The amine **6** was obtained after purification via column chromatography (DCM/MeOH = 98/2 with 0.1 v/v% TEA) as a yellow solid (79.7 mg,370 µmol, 19%).

TLC: R_f = 0.15 (DCM/MeOH = 95/5) [UV/Ninhydrin].

^{1}H-NMR (500 MHz, DMSO-d$_6$): δ [ppm] = 1.21 (d, ^{3}J = 7.0 Hz, 6 H), 2.88 (sept, ^{3}J = 7.0 Hz, 1 H), 3.72 (s, 2 H), 6.29 (d, ^{3}J = 3.2 Hz, 1 H), 6.75 (d, ^{3}J = 3.2 Hz, 1 H), 7.26 (dq$_{A\text{-}B}$, ^{3}J = 8.3 Hz, $J_{A\text{-}B}$ = 4.2 Hz, 2 H), 7.57 (dq$_{A\text{-}B}$, ^{3}J = 8.3 Hz, $J_{A\text{-}B}$ = 4.2 Hz, 2 H).

^{13}C{^{1}H}NMR (125 MHz, DMSO-d$_6$): δ [ppm] = 23.9 (s), 33.2 (s), 38.8 (s), 105.8 (s), 107.5 (s), 123.3 (s), 126.8 (s), 128.4 (s), 147.4 (s), 151.9 (s), 156.7 (s).

HR-MS (ESI): calc. for $[C_{14}H_{15}O]^+$ ($[M\text{-}NH_2]^+$): 199.1123, found 199.1118.

Amino(((5-(4-isopropylphenyl)furan-2-yl)methyl)amino)methaniminium 2,2,2-trifluoroacetate (D05)

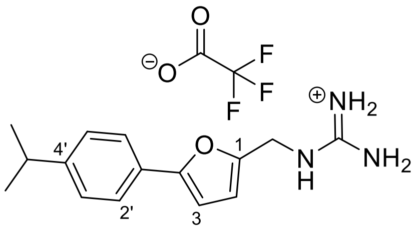

$C_{17}H_{20}F_3N_3O_3$
371.36 g/mol
D05

The general procedure II was followed using amine **6** (56.0 mg, 260 µmol, 1.00 eq.) as described. After purification via preparative HPLC (column 1, method 1, λ = 286 nm), the guanidine **D05** was obtained as a brown solid (81.7 mg, 220 µmol, 85%).

^{1}H-NMR (500 MHz, DMSO-d$_6$): δ [ppm] = 1.21 (d, ^{3}J = 7.0 Hz, 6 H), 2.90 (sept, ^{3}J = 7.0 Hz, 1 H), 4.45 (d, ^{3}J = 6.0 Hz, 2 H), 6.47 (d, ^{3}J = 3.4 Hz, 1 H), 6.83 (d, ^{3}J = 3.4 Hz, 1 H), 7.30 (d, ^{3}J = 8.2 Hz, 2 H), 7.59 (d, ^{3}J = 8.2 Hz, 2 H), 7.96 (t, ^{3}J = 6.0 Hz, 1 H).

^{13}C{^{1}H}NMR (125 MHz, DMSO-d$_6$): δ [ppm] = 23.8 (s), 33.2 (s), 37.8 (s), 105.8 (s), 110.2 (s), 123.5 (s), 126.9 (s), 127.8 (s), 148.0 (s), 149.6 (s), 153.2 (s), 156.8 (s).

^{19}F-NMR (375 MHz, DMSO-d$_6$): δ [ppm] = 73.8 (s).

HR-MS (ESI): calc. for $[C_{14}H_{15}O]^+$ ($[M\text{-}CH_5N_3]^+$): 199.1123, found 199.1118; calc. for $[C_{15}H_{20}N_3O]^+$ ($[M+H]^+$): 258.1601, found 258.1601.

(5-(4-(Trifluoromethyl)phenyl)furan-2-yl)methanamine (7)

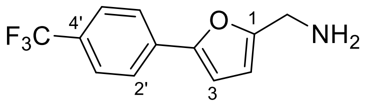

$C_{12}H_{10}F_3NO$
241.21 g/mol
7

The general procedure I was followed using 4-(trifluoromethal)aniline (326 mg, 245 µL, 2.00 mmol, 1.00 eq.) and furfurylamine (991 mg, 944 µL, 10.0 mmol, 5.00 eq.) as described. The amine **7** was obtained after purification via column chromatography (DCM/MeOH = 98/2 with 0.1 v/v% TEA) as an orange-yellow solid (196 mg, 816 µmol, 41%).

TLC: R_f = 0.41 (DCM/MeOH = 95/5) [UV/Ninhydrin].

^{1}H-NMR (500 MHz, DMSO-d$_6$): δ [ppm] = 3.75 (s, 2 H), 6.39 (d, ^3J = 3.2 Hz, 1 H), 7.07 (d, ^3J = 3.2 Hz,1 H), 7.75 (d, ^3J = 8.4 Hz, 2 H), 7.87 (d, ^3J = 8.4 Hz, 2 H).

^{13}C{^{1}H}NMR (125 MHz, DMSO-d$_6$): δ [ppm] = 107.8 (s), 109.2 (s), 123.5 (s), 124.4 (q, $^1J_{C\text{-}F}$ = 272.0 Hz) 125.9 (q, $^3J_{C\text{-}F}$ = 3.8 Hz), 126.9 (q, $^2J_{C\text{-}F}$ = 31.8 Hz), 134.2 (s), 150.1 (s), 159.1 (s). The ^{13}C signal of the CH_2 group overlaps with the solvent signal and can, therefore, not be localized.

^{19}F-NMR (375 MHz, DMSO-d$_6$): δ [ppm] = - 60.9 (s).

HR-MS (ESI): calc. for $[C_{12}H_8F_3O]^+$ ($[M\text{-}NH_2]^+$): 225.0527, found 225.0520.

Amino(((5-(4-(trifluoromethyl)phenyl)furan-2-yl)methyl)amino)methaniminium 2,2,2-trifluoroacetate (D06)

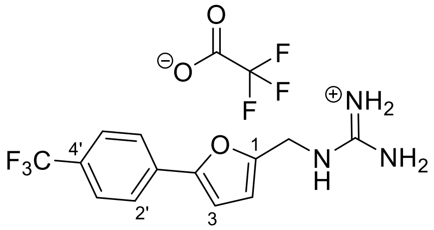

$C_{15}H_{13}F_6N_3O_3$
397.28 g/mol
D06

The general procedure II was followed using amine **7** (79.6 mg, 330 µmol, 1.00 eq.) as described. After purification via preparative HPLC (column 1, method 1, λ = 292 nm), the guanidine **D06** was obtained as a yellowish solid (101 mg, 254 µmol, 77%).

^{1}H-NMR (500 MHz, DMSO-d$_6$): δ [ppm] = 4.49 (d, ^3J = 6.0 Hz, 2 H), 6.56 (d, ^3J = 3.3 Hz, 1 H), 7.17 (d, ^3J = 3.3 Hz, 1 H), 7.80 (d, ^3J = 8.3 Hz, 2 H), 7.89 (d, ^3J = 8.3 Hz, 2 H), 8.06 (bs, 1 H).

^{13}C{^{1}H}NMR (125 MHz, DMSO-d$_6$): δ [ppm] = 37.8 (s), 109.2 (s), 110.5 (s), 123.7 (s), 124.3 (q, $^1J_{C\text{-}F}$ = 272.8 Hz), 126.0 (q, $^3J_{C\text{-}F}$ = 3.8 Hz), 127.5 (q, $^2J_{C\text{-}F}$ = 32.0 Hz), 133.6 (s), 151.4 (s), 151.5 (s), 156.8 (s).

^{19}F-NMR (375 MHz, DMSO-d$_6$): δ [ppm] = - 61.9 (s), - 73.7 (s).

HR-MS (ESI): calc. for $[C_{12}H_8F_3O]^+$ ($[M\text{-}CH_5N_3]^+$): 225.0527, found 225.0521; calc. for $[C_{13}H_{13}F_3N_3O]^+$ ($[M+H]^+$): 284.1005, found 284.1004.

(5-(4-(*tert*-Butyl)phenyl)furan-2-yl)methanamine (8)

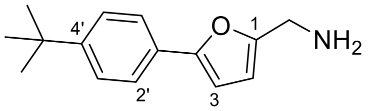

$C_{15}H_{19}NO$
229.32 g/mol
8

The general procedure I was followed using 4-*tert*-butylaniline (298 mg, 316 µL, 2.00 mmol, 1.00 eq.) and furfurylamine (991 mg, 944 µL, 10.0 mmol, 5.00 eq.) as described. The amine **8** was obtained after purification via column chromatography (DCM/MeOH = 98/2→95/5 with 0.1 v/v% TEA) as a yellow solid (52.5 mg, 229 µmol, 11%).

TLC: R_f = 0.26 (DCM/MeOH = 95/5) [UV/Ninhydrin].

¹H-NMR (500 MHz, DMSO-d$_6$): δ [ppm] = 1.29 (s, 9 H), 3.72 (s, 2 H), 6.29 (d, ^3J = 3.3 Hz, 1 H), 6.76 (d, ^3J = 3.3 Hz, 1 H), 7.41 (dq$_{A-B}$, ^3J = 8.5 Hz, J_{A-B} = 4.4 Hz, 2 H), 7.58 (dq$_{A-B}$, ^3J = 8.5 Hz, J_{A-B} = 4.4 Hz, 2 H).

^{13}C{^{1}H}NMR (125 MHz, DMSO-d$_6$): δ [ppm] = 31.1 (s), 34.4 (s), 105.8 (s), 107.2 (s), 122.9 (s), 125.6 (s), 128.0 (s), 149.5 (s), 151.7 (s), 157.3 (s). The ^{13}C signal of the CH_2 group overlaps with the solvent signal and can, therefore, not be localized.

HR-MS (ESI): calc. for $[C_{15}H_{17}O]^+$ ($[M-NH_2]^+$): 213.1279, found 213.1273.

Amino(((5-(4-(*tert*-butyl)phenyl)furan-2-yl)methyl)amino)methaniminium 2,2,2-trifluoro-acetate (D07)

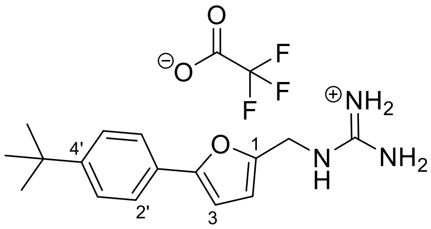

$C_{18}H_{22}F_3N_3O_3$
385.39 g/mol
D07

The general procedure II was followed using amine **8** (50.0 mg, 218 µmol, 1.00 eq.) as described. After purification via preparative HPLC (column 1, method 1, λ = 280 nm), the guanidine **D07** was obtained as an orange solid (58.4 mg, 152 µmol, 70%).

¹H-NMR (500 MHz, DMSO-d$_6$): δ [ppm] = 1.29 (s, 9 H), 4.46 (d, ^3J = 5.8 Hz, 1 H), 6.48 (d, ^3J = 3.3 Hz, 1 H), 6.85 (d, ^3J = 3.3 Hz, 1 H), 7.45 (d, ^3J = 8.5 Hz, 2 H), 7.60 (d, ^3J = 8.5 Hz, 2 H), 7.93 (t, ^3J = 5.8 Hz, 1H).

^{13}C{^{1}H}NMR (125 MHz, DMSO-d$_6$): δ [ppm] = 31.1 (s), 34.4 (s), 37.8 (s), 105.9 (s), 110.2 (s), 123.2 (s), 125.7 (s), 127.4 (s), 149.7 (s), 150.2 (s), 153.1 (s), 156.8 (s).

^{19}F-NMR (375 MHz, DMSO-d$_6$): δ [ppm] = - 73.5 (s).

HR-MS (ESI): calc. for $[C_{15}H_{17}O]^+$ ($[M-CH_5N_3]^+$): 213.1279, found 213.1273; calc. for $[C_{16}H_{22}N_3O]^+$ ($[M+H]^+$): 272.1757, found 272.1756.

(5-(4-(Methylsulfonyl)phenyl)furan-2-yl)methanamine (9)

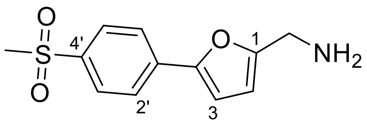

$C_{12}H_{13}NO_3S$
251.30 g/mol
9

The general procedure I was followed using 4-(methylsulfonyl) aniline (349 mg, 243 µL, 2.00 mmol, 1.00 eq.) and furfurylamine (991 mg, 944 µL, 10.0 mmol, 5.00 eq.) as described. The amine **9** was obtained after purification via column chromatography (DCM/MeOH = 98/2 with 0.1 v/v% TEA) as a yellow solid (144 mg, 573 µmol, 29%).

TLC: R_f = 0.22 (DCM/MeOH = 9/1) [UV/Ninhydrin].

¹H-NMR (300 MHz, DMSO-d$_6$): δ [ppm] = 3.22 (s, 3 H), 3.76 (s, 2 H), 6.40 (d, ^3J = 3.3 Hz, 1 H), 7.13 (d, ^3J = 3.3 Hz, 1 H), 7.87-7.96 (m, 4 H).

^{13}C{^{1}H}NMR (125 MHz, DMSO-d$_6$): δ [ppm] = 38.8 (s), 43.6 (s), 108.1 (s), 110.0 (s), 123.4 (s), 127.8 (s), 134.9 (s), 138.5 (s), 150.0 (s), 159.1 (s).

HR-MS (ESI): calc. for $[C_{12}H_{11}O_3S]^+$ ($[M-NH_2]^+$): 235.0429, found 235.0424.

Amino(((5-(4-(methylsulfonyl)phenyl)furan-2-yl)methyl)amino)methaniminium 2,2,2-trifluoroacetate (D08)

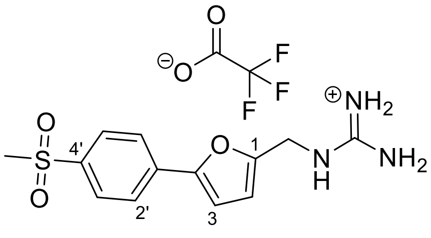

$C_{15}H_{16}F_3N_3O_5S$
407.36 g/mol
D08

The general procedure II was followed using amine **9** (126 mg, 500 µmol, 1.00 eq.) with a reaction time of 48 h. After the addition of further *N*, *N*-diisopropyl-ethylamine (6.51 mg, 9.00µL, 52.5 µmol, 0.10 eq.) and 1*H*-pyrazole-1-carboxamidin hydrochloride (7.30 mg, 50.0 µmol, 0.10 eq.), the reaction was allowed to stir for further 72 h at RT. After purification via preparative HPLC (column 1, method 3, λ = 307 nm), the guanidine **D08** was obtained as a yellow solid (127 mg, 312 µmol, 62%).

^{1}H-NMR (500 MHz, DMSO-d$_6$): δ [ppm] = 3.24 (s, 3 H), 4.50 (d, ^{3}J = 6.1 Hz, 2 H), 6.57 (d, ^{3}J = 3.4 Hz, 1 H), 7.21 (d, ^{3}J = 3.4 Hz, 1 H), 7.92 (d, ^{3}J = 8.6 Hz, 2 H), 7.97 (d, ^{3}J = 8.6 Hz, 2 H), 8.03 (bs, 1 H).

^{3}C{^{1}H}NMR (125 MHz, DMSO-d$_6$): δ [ppm] = 37.8 (s), 43.5 (s), 109.9 (s), 110.6 (s), 123.7 (s), 127.9 (s), 134.4 (s), 139.1 (s), 151.2 (s), 151.8 (s), 156.8 (s).

^{19}F-NMR (375 MHz, DMSO-d$_6$): δ [ppm] = - 73.5 (s).

HR-MS (ESI): calc. for $[C_{12}H_{11}O_3S]^+$ ($[M-CH_5N_3]^+$): 235.0429, found 235.0425; calc. for $[C_{13}H_{16}N_3O_3S]^+$ ($[M+H]^+$): 294.0907, found 294.0908.

Amino((furan-2-ylmethyl)amino)methaniminium 2,2,2-trifluoroacetate (D09)

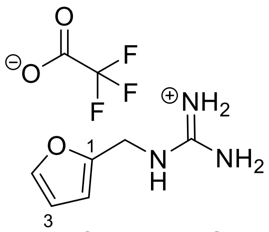

$C_8H_{10}F_3N_3O_3$
253.18 g/mol
D09

The general procedure II was followed using furfurylamine (70.0 mg, 706 µmol, 1.00 eq.) as described. After purification via preparative HPLC (column 1, method 9, λ = 223 nm), the guanidine **D06** was obtained as a white solid (16.8 mg, 66.3 µmol, 9%).

^{1}H-NMR (500 MHz, DMSO-d$_6$): δ [ppm] = 4.38 (d, ^{3}J = 5.9 Hz, 2 H), 6.39 (dd, ^{3}J = 3.2 Hz, ^{4}J = 0.7 Hz, 1 H), 6.45 (dd, ^{3}J = 3.2 Hz, ^{4}J = 1.9 Hz, 1 H), 7.67 (dd, ^{3}J = 1.9 Hz, ^{4}J = 0.7 Hz, 1 H), 7.92 (t, ^{3}J = 5.9 Hz, 1 H).

^{13}C{^{1}H}NMR (125 MHz, DMSO-d$_6$): δ [ppm] = 37.5 (s), 108.1 (s), 110.6 (s), 143.1 (s), 150.1 (s), 156.7 (s).

^{9}F-NMR (375 MHz, DMSO-d$_6$): δ [ppm] = - 73.5 (s).
HR-MS (ESI): calc. for $[C_5H_5O]^+$ ($[M-CH_5N_3]^+$): 81.0340, found 81.0339; calc. for $[C_6H_{10}N_3O]^+$ ($[M+H]^+$): 140.0818, found 140.0815.

5-(4-Bromophenyl)thiophene-2-carbaldehyde (10)

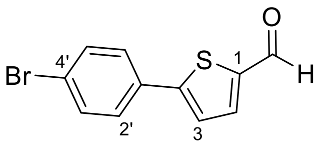

$C_{11}H_7BrOS$
267.14 g/mol
10

According to a modified literature procedure[56], to a solution of 5-bromothiophene-2-carbaldehyde (247 mg, 154 mL, 1.29 mmol, 1.30 eq.) in 1,2-dimethoxyethane (2 mL, 500 mM), tetrakis(triphenylphosphine)-palladium(0) (57.5 mg, 49.8 µmol, 5 mol-%) and sodium carbonate solution (aqueous, 2 M, 996 µL) were added. After flushing the flask with argon, the reaction mixture was stirred for 15 min at RT. Then, (4-bromophenyl)boronic acid (200 mg, 996 mmol, 1.00 eq.) in ethanol (2 mL,

500 mM) was added, and the reaction mixture was degassed with argon and stirred for 16 h at 90 °C. 1,2-Dimethoxyethane (1 mL) and ethanol (1 mL) were added, and the reaction mixture was stirred for 5 h at 90 °C. After evaporation of the solvent, the residue was dissolved in a minimal amount of dichloromethane and water. The phases were separated, and the aqueous phase was extracted with dichloromethane (3× 25 mL). The organic phase was dried with sodium sulfate, filtered, and the solvent was evaporated. The aldehyde **10** was obtained via column chromatography (P/EtOAc = 20/1) as a light orange solid (201 mg, 753 mmol, 76%) after purification.

TLC: R_f = 0.06 (P/ EtOAc = 40/1) [UV].

^{1}H-NMR (500 MHz, $CDCl_3$) : δ [ppm] = 7.40 (d, ^3J = 4.0 Hz, 1 H), 7.55 (dq$_{A\text{-}B}$, ^3J = 8.8 Hz, $J_{A\text{-}B}$ = 4.3 Hz, 2 H), 7.57 (dq$_{A\text{-}B}$, ^3J = 8.8 Hz, $J_{A\text{-}B}$ = 4.3 Hz, 2 H), 7.74 (d, ^3J = 4.0 Hz, 1 H), 9.90 (s, 1 H).

^{13}C{^{1}H}NMR (125 MHz, $CDCl_3$): δ [ppm] = 123.8 (s), 124.6 (s), 128.0 (s), 132.2 (s), 132.5 (s), 137.6 (s), 142.9 (s), 152.9 (s), 183.0 (s).

HR-MS (ESI): calc. for $[C_{11}H_8{}^{79}BrOS]^+$ ($[M+H]^+$): 266.9474, found 266.9477; calc. for $[C_{11}H_8{}^{81}BrOS]^+$ ($[M+H]^+$): 268.9353, found 268.9449.

Analytical data are in accordance with the literature.[57]

5-(4-Bromophenyl)thiophene-2-carbaldehyde oxime (11)

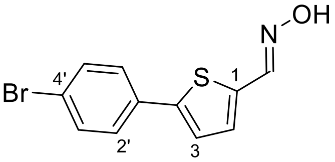

$C_{11}H_8BrNOS$
282.16 g/mol
11

The General Procedure III was followed using aldehyde **10** (103 mg, 386 mmol, 1.00 eq.) as described. Subsequently, hydroxylamine hydrochloride (13.4 mg, 193 mmol, 0.50 eq.) was added, and the reaction mixture was stirred for another hour at 80 °C. The crude product was directly used in the next reaction without further purification and characterization.

TLC: R_f = 0.34 (H/EtOAc = 1/1) [$KMnO_4$].

(5-(4-Bromophenyl)thiophen-2-yl)methanamine (12)

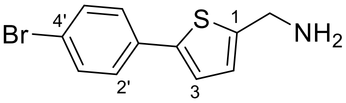

$C_{11}H_{10}BrNS$
268.17 g/mol
12

The General Procedure IV was followed using oxime **11** (109 mg, 386 mmol, 1.00 eq.) as described. After purification via column chromatography (DCM/MeOH = 95/5), the amine **12** was obtained as an orange solid (51.8 mg, 193 mmol, 50% over two steps).

TLC: R_f = 0.28 (DCM/MeOH = 9/1) [Ninhydrin].

^{1}H-NMR (500 MHz, DMSO-d$_6$): δ [ppm] = 3.90 (s, 2 H), 6.94 (d, ^3J = 3.7 Hz, 1 H), 7.38 (d, ^3J = 3.7 Hz, 1 H), 7.5-7.59 (m, 4 H).

^{13}C{^{1}H}NMR (125 MHz, DMSO-d$_6$): δ [ppm] = 40.0 (s), 120.0 (s), 123.9 (s), 124.5 (s), 126.9 (s), 132.0 (s), 133.5 (s), 139.6 (s), 149.6 (s).

HR-MS (ESI): calc. for $[C_{11}H_8{}^{79}BrS]^+$ ($[M\text{-}NH_2]^+$): 250.9530, found 250.9527; calc. for $[C_{11}H_8{}^{81}BrS]^+$ ($[M\text{-}NH_2]^+$): 252.9510, found 252.9500.

Amino(((5-(4-bromophenyl)thiophen-2-yl)methyl)amino)methaniminium 2,2,2-trifluoroacetate (D10)

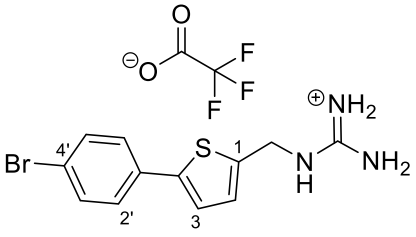

$C_{14}H_{13}BrF_3N_3O_2S$
424.24 g/mol
D10

The general procedure II was followed using amine **12** (49.6 mg, 185 µmol, 1.00 eq.) as described. The guanidine **D10** was obtained after purification via preparative HPLC (column 1, method 2, λ = 295 nm) as a white solid (48.5 mg, 114 µmol, 62%).

^{1}H-NMR (500 MHz, DMSO-d$_6$): δ [ppm] = 4.57 (d, ^3J = 6.2 Hz, 2 H), 7.09 (d, ^3J = 3.7 Hz, 1 H), 7.45 (d, ^3J = 3.7 Hz, 1 H), 7.58 (dq$_{A-B}$, ^3J = 6.6 Hz, J_{A-B} = 4.2 Hz, 2 H), 7.61 (dq$_{A-B}$, ^3J = 6.6 Hz, J_{A-B} = 4.2 Hz, 2 H), 7.97 (t, ^3J = 6.2 Hz, 1 H).

^{13}C{^{1}H}NMR (125 MHz, DMSO-d$_6$): δ [ppm] = 120.74 (s), 124.2 (s), 127.2 (s), 127.9 (s), 132.1 (s), 132.8 (s), 140.1 (s), 141.7 (s), 156.5 (s). The ^{13}C signal of the CH_2 group overlaps with the solvent signal and can, therefore, not be localized.

^{19}F-NMR (375 MHz, DMSO-d$_6$): δ [ppm] = - 73.4 (s).

HR-MS (ESI): calc. for $[C_{11}H_8{}^{79}BrS]^+$ ($[M-CH_5N_3]^+$): 250.9530, found 250.9528; calc. for $[C_{11}H_8{}^{81}BrS]^+$ ($[M-CH_5N_3]^+$): 252.9510, found 252.9500; calc. for $[C_{12}H_{13}{}^{79}BrN_3S]^+$ ($[M+H]^+$): 310.0002, found 310.0009; calc. for $[C_{12}H_{13}{}^{81}BrN_3S]^+$ ($[M+H]^+$): 311.9988, found 311.9986.

2-(4-Bromophenyl)thiazole-5-carbaldehyde (13)

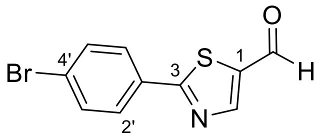

$C_{10}H_6BrNOS$
268.13 g/mol
13

According to a modified literature procedure[56], 2-bromo-5-formylthiazole (392 mg, 2.00 mmol, 1.00 eq.) was dissolved in 1,2-dimethoxyethane (6 mL, 333 mM), tetrakis(triphenylphosphine)-palladium(0) (117 mg, 100 µmol, 5 mol%) and sodium carbonate solution (2 M in water, 2 mL) were added and the reaction mixture was degassed. 4-Bromophenylboronic acid (455 mg, 2.20 mmol, 1.10 eq.) was dissolved in ethanol (4 mL), degassed and added. After stirring for 72 h at 90 °C, the reaction mixture was extracted with dichloromethane (3 × 50 mL), and the organic layer was washed with brine (50 mL) and dried over magnesium sulfate. The solvent was removed, and the aldehyde **13** was obtained after purification via column chromatography (P/EtOAc = 95/5 →9/1→8/2) as a yellow solid (116 mg, 434 µmol, 22%).

TLC: R_f = 0.67 (P/EtOAc = 8/2) [DNP].

^{1}H-NMR (500 MHz, $CDCl_3$: δ [ppm] = 7.64 (dq$_{A-B}$, ^3J = 8.7 Hz, J_{A-B} = 4.5 Hz, 2 H), 7.90 (dq$_{A-B}$, ^3J = 8.7 Hz, J_{A-B} = 4.5 Hz, 2 H), 8.43 (s, 1 H), 10.1 (s, 1 H).

^{13}C{^{1}H}NMR (125 MHz, $CDCl_3$): δ [ppm] = 126.7 (s), 128.8 (s), 131.6 (s), 132.7 (s), 139.3 (s), 152.4 (s), 174.3 (s), 182.2 (s).

HR-MS (ESI): calc. for $[C_{10}H_7{}^{79}BrNOS]^+$ ($[M+H]^+$): 267.9426, found 267.9422; calc. for $[C_{10}H_7{}^{81}BrNOS]^+$ ($[M+H]^+$): 269.9406, found 269.9399.

2-(4-Bromophenyl)thiazole-5-carbaldehyde oxime (14)

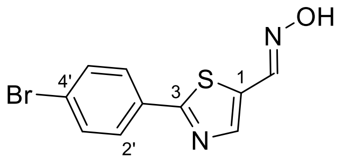

$C_{10}H_7BrN_2OS$
283.14 g/mol
14

The General Procedure III was followed using aldehyde **13** (200 mg, 746 µmol, 1.00 eq) as described. The crude product was directly used in the next reaction without further purification and characterization.

(2-(4-Bromophenyl)thiazol-5-yl)methanamine (15)

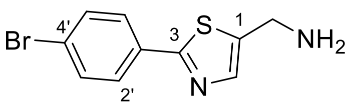

$C_{10}H_9BrN_2S$
269.1600 g/mol
15

The General Procedure IV was followed using oxime **14** (211 mg, 746 µmol, 1.00 eq.) as described. After purification via column chromatography (DCM/MeOH = 95/5 with 0.1 v/v% TEA), amine **15** was obtained as a yellowish solid (120 mg, 446 µmol, 60% over two steps).

TLC: R_f = 0.21 (DCM/MeOH=9/1) [UV/Ninhydrin].

^{1}H-NMR (500 MHz, $CDCl_3$: δ [ppm] = 3.95 (d, ^4J = 1.0 Hz, 2 H), 7.68 (dq$_{A-B}$, ^3J = 8.6 Hz, J_{A-B} = 4.6 Hz, 2 H), 7.84 (dq$_{A-B}$, ^3J = 8.6 Hz, J_{A-B} = 4.6 Hz, 2 H), 7.71 (t, ^4J = 1.0 Hz, 1 H).
^{13}C{^{1}H}NMR (125 MHz, $CDCl_3$): δ [ppm] = 38.3 (s), 123.1 (s), 127.7 (s), 132.2 (s), 132.7 (s), 139.9 (s), 144.7 (s), 164.2 (s).
HR-MS (ESI): calc. for $[C_{10}H_7{}^{79}BrNS]^+$ ($[M-NH_2]^+$): 251.9483, found 251.9481; calc. for $[C_{10}H_7{}^{81}BrNS]^+$ ($[M-NH_2]^+$): 253.9462, found 253.9458.

Amino(((2-(4-bromophenyl)thiazol-5-yl)methyl)amino)methaniminium 2,2,2-trifluoro-acetate (D11)

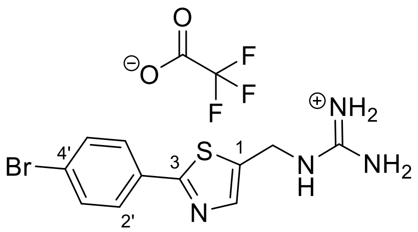

$C_{13}H_{12}BrF_3N_4O_2S$
425.22 g/mol
D11

The general procedure II was followed using amine **15** (70.0 mg, 260 µmol, 1.00 eq.) for 22 h. After the addition of further *N*, *N*-diisopropylethylamine (35.5 mg, 47.6 µL, 273µmol, 1.05 eq.), and acetonitrile (500 µL), the reaction was allowed to stir for another 20 h. The guanidine **D11** was obtained after purification via preparative HPLC (column 1, method 6, λ = 290 nm) as a colorless solid (65.5 mg, 154 µmol, 59%).

^{1}H-NMR (500 MHz, DMSO-d$_6$): δ [ppm] = 4.66 (d, ^3J = 6.2 Hz, 2 H), 7.21 (dq$_{A-B}$, ^3J = 8.5 Hz, J_{A-B} = 4.6 Hz, 2 H), 7.85-7.88 (m, 3 H), 7.71 (t, ^3J = 6.2 Hz, 1 H).

^{13}C{^{1}H}NMR (125 MHz, DMSO-d$_6$): δ [ppm] = 38.9 (s), 123.7 (s), 128.0 (s), 132.1 (s), 132.3 (s), 135.7 (s), 142.8 (s), 156.6 (s), 166.1 (s).

^{19}F-NMR (375 MHz, DMSO-d$_6$): δ [ppm] = - 74.5 (s).

HR-MS (ESI): calc. for $[C_{10}H_7{}^{79}BrNS]^+$ ($[M-CH_5N_3]^+$): 251.9483, found 251.9480; calc. for $[C_{10}H_7{}^{81}BrNS]^+$ ($[M-CH_5N_3]^+$): 253.9462, found 253.9457; calc. for $[C_{11}H_{12}{}^{79}BrN_4S]^+$ ($[M+H]^+$): 310.9961, found 310.9964; calc. for $[C_{11}H_{12}{}^{81}BrN_4S]^+$ ($[M+H]^+$): 312.9940, found 312.9941.

1-(4-Bromophenyl)-1*H*-pyrazole-5-carbaldehyde (16)

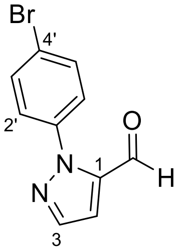

$C_{10}H_7BrN_2O$
251.08 g/mol
16

1*H*-Pyrazol-3-carbaldehyde (240 mg, 2.50 mmol, 1.00 eq.), 4-bromophenyl-boronic acid (600 mg, 2.99 mmol, 1.20 eq.), copper sulfate (136 mg, 7.50 µmol,0.30 eq.) and potassium carbonate (1.04 g, 7.50 mmol, 3.00 eq.) were dissolved in 1,2-dimethoxyethane (10 mL, 250 mM) and water (230 µL). The atmosphere was replaced by oxygen (1 atm), and the reaction mixture was stirred overnight at 70 °C before filtration over celite and washing with 1,2-dimethoxyethane (3 × 10 mL). After removal of the solvent in vacuo, the reaction mixture was purified by column chromatography (P/EtOAc = 10/1), dissolved in a minimal amount of acetonitrile and precipitated by adding excess of water. Aldehyde **16** was obtained as colorless needles (134 mg, 5.35 µmol, 21%).

TLC: R_f = 0.61 (P/EtOAc = 8/2) [DNP].

^{1}H-NMR (500 MHz, $CDCl_3$): δ[ppm] = 7.01 (d, ^3J = 2.6 Hz, 1 H), 7.62-7.68 (m, 4 H), 7.95 (d, ^3J = 2.6 Hz, 1 H), 10.1 (s, 1 H).

^{13}C{^{1}H}NMR (125 MHz, $CDCl_3$): δ[ppm] = 107.8 (s), 121.4 (s), 121.6 (s), 129.0 (s), 132.9 (s), 138.7 (s), 152.8 (s), 186.6 (s).

HR-MS (ESI): calc. for $[C_{10}H_8{}^{79}BrN_2O]^+$ ($[M+H]^+$): 250.9815, found 250.9815; calc. for $[C_{10}H_8{}^{81}BrN_2O]^+$ ($[M+H]^+$): 252.9794, found 252.9793.

1-(4-Bromophenyl)-1*H*-pyrazole-5-carbaldehyde oxime (17)

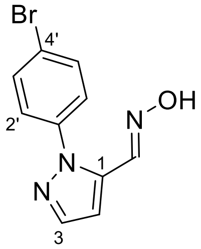

$C_{10}H_8BrN_3O$
266.10 g/mol
17

The General Procedure III was followed using aldehyde **16** (132 mg, 526 µmol, 1.00 eq) as described. The crude product was directly used in the next reaction without further purification and characterization.

TLC: R_f = 0.79 (P/EtOAc=8/2) [DNP].

(1-(4-Bromophenyl)-1*H*-pyrazol-5-yl)methanamine (18)

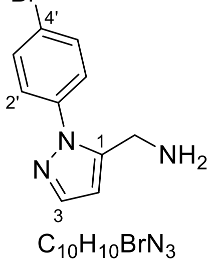

$C_{10}H_{10}BrN_3$
252.12 g/mol
18

The General Procedure IV was followed using oxime **17** (140 mg, 526 µmol, 1.00 eq.) as described. After purification via column chromatography (DCM/MeOH = 9/1 with 0.1 v/v% TEA), amine **18** (99.0 mg, 393 µmol, 75% over two steps) was obtained as a colorless solid.

TLC: R_f = 0.06 (DCM/MeOH = 9/1) [UV/Ninhydrin].

^{1}H-NMR (500 MHz, DMSO-d_6): δ[ppm] = 3.73 (s, 2 H), 6.51 (d, ^3J = 2.5 Hz, 1 H), 7.66 (dq_{A-B}, ^3J = 9.0 Hz, J_{A-B} = 4.0 Hz, 2 H), 7.77 (dq_{A-B}, ^3J = 9.0 Hz, J_{A-B} = 4.0 Hz, 2 H), 8.43 (d, ^3J = 2.5 Hz, 1 H).

^{13}C{^{1}H}NMR (125 MHz, DMSO-d$_6$): δ [ppm] = 106.6 (s), 117.9 (s), 119.8 (s), 128.4 (s), 132.3 (s), 139.0 (s), 156.9 (s). The ^{13}C signal of the CH$_2$ group overlaps with the solvent signal and can, therefore, not be localized.

HR-MS (ESI): calc. for [$C_{10}H_8{}^{79}BrN_2$]$^+$ ([M-NH$_2$]$^+$): 234.9871, found 234.9865; calc. for [$C_{10}H_8{}^{81}BrN_2$]$^+$ ([M-NH$_2$]$^+$): 236.9850, found 236.9842; calc. for [$C_{10}H_{11}{}^{79}BrN_3$]$^+$ ([M+H]$^+$): 254.0131, found 254.0129; calc. for [$C_{10}H_{11}{}^{81}BrN_3$]$^+$ ([M+H]$^+$): 252.0110, found 254.0108.

Amino(((1-(4-bromophenyl)-1*H*-pyrazol-5-yl)methyl)amino)methaniminium 2,2,2-trifluoro-acetate (D12)

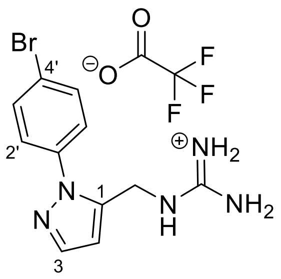

$C_{13}H_{13}BrF_3N_5O_2$
408.18 g/mol
D12

The general procedure II was followed using amine **18** (70.1 mg, 278 µmol, 1.00 eq.) as described. The guanidine **D12** was obtained after purification via preparative HPLC (column 1, method 7, λ = 283 nm) as a colorless solid (46.5 mg, 114 µmol, 41%).

^{1}H-NMR (500 MHz, DMSO-d$_6$): δ [ppm] = 4.43 (d, ^3J = 5.8 Hz, 2 H), 6.52 (d, ^3J = 2.5 Hz, 1 H), 7.70 (dq$_{A\text{-}B}$, ^3J = 9.0 Hz, $J_{A\text{-}B}$ = 4.9 Hz, 2 H), 7.80 (dq$_{A\text{-}B}$, ^3J = 9.0 Hz, $J_{A\text{-}B}$ = 4.9 Hz, 2 H), 8.53 (d, ^3J = 2.5 Hz, 1 H), 7.93 (bs, 1 H).

^{13}C{^{1}H}NMR (125 MHz, DMSO-d$_6$): δ [ppm] = 38.7 (s), 107.1 (s), 118.6 (s), 120.1 (s), 129.3 (s), 132.5 (s), 138.7 (s), 150.1 (s), 157.0 (s).

^{19}F-NMR (375 MHz, DMSO-d$_6$): δ [ppm] = - 73.5 (s).

HR-MS (ESI): calc. for [$C_{10}H_8{}^{79}BrN_2$]$^+$ ([M-CH$_5$N$_3$]$^+$): 234.9871, found 234.9866; calc. for [$C_{10}H_8{}^{81}BrN_2$]$^+$ ([M-CH$_5$N$_3$]$^+$): 236.9850, found 236.9845; calc. for [$C_{11}H_{13}{}^{79}BrN_5$]$^+$ ([M+H]$^+$): 294.0349, found 294.0349; calc. for [$C_{11}H_{13}{}^{81}BrN_5$]$^+$ ([M+H]$^+$): 296.0328, found 296.0327.

5-(Pyridin-2-yl)thiophene-2-carbaldehyde (19)

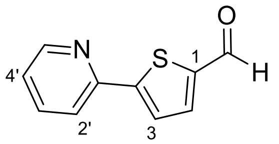

$C_{10}H_7NOS$
189.23 g/mol
19

According to a modified literature procedure[56], a solution of 2-bromopyridine (385 mg, 233 mL, 2.41 mmol, 1.00 eq.) and sodium carbonate solution (aqueous, 2 M, 24.2 µL) in 1,2-dimethoxyethane (12 mL, 20 mM) was degassed. Tetrakis(triphenylphosphine)palladium(0) (141 mg, 12.1 µmol, 5 mol%) and 5-formyl-2-thiopheneboronic acid (400 mg, 2.43 mmol, 1.01 eq.) were added and the reaction mixture was stirred at 100 °C for 40 h. The aqueous phase was extracted with dichloromethane (3 × 50 mL), the organic phase was washed with brine (50 mL), dried with sodium sulfate, filtered, and the solvent was evaporated. The aldehyde **19** (187 mg, 988 mmol, 41%) was obtained after purification via column chromatography (P/EtOAc = 8/2→7/2→6/4) as a yellow solid.

TLC: R_f = 0.23 (P/EtOAc = 8/2) [UV].

^{1}H-NMR (500 MHz, CDCl$_3$): δ [ppm] = 7.26-7.29 (m, 1 H), 7.69 (d, ^3J = 4.0 Hz, 1 H), 7.72-7.78 (m, 3 H), 8.64 (*virt.* dt, ^3J = 4.8 Hz, $^4J \approx {}^5J$ = 1.2 Hz, 1 H), 9.93 (s, 1 H).

^{13}C{^{1}H}NMR (125 MHz, CDCl$_3$): δ [ppm] = 114.9 (s), 118.8 (s), 120.2 (s), 132.0 (s), 132.1 (s), 139.3 (s), 145.2 (s), 146.3 (s), 149.1 (s), 178.3 (s).

HR-MS (ESI): calc. for [$C_{10}H_8NOS$]$^+$ ([M+H]$^+$): 190.0321, found 190.0321.

Analytical data are in accordance with the literature.[58]

5-(Pyridin-2-yl)thiophene-2-carbaldehyde oxime (20)

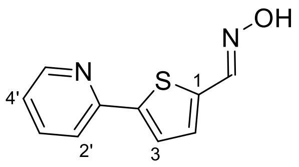

$C_{10}H_8N_2OS$
204.25 g/mol
20

The General Procedure III was followed using aldehyde **19** (214 mg, 1.13 mmol, 1.00 eq) as described. Subsequently, another portion of hydroxylamine hydrochloride (39.3 mg, 565 µmol, 0.50 eq.) was added, and the reaction mixture was stirred for another hour at 80 °C. The crude product was directly used in the following reaction without further purification and characterization.

(5-(Pyridin-2-yl)thiophen-2-yl)methanamine (21)

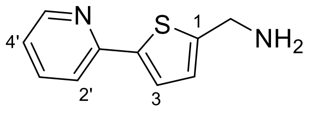

$C_{10}H_{10}N_2S$
190.26 g/mol
21

The General Procedure IV was followed using oxime **20** (231 mg, 1.13 mmol, 1.00 eq.) as described and stirred for 72 h. After the filtration over celite and adjustment of the pH as described, the impurities were separated by extraction with dichloromethane (3 × 20 mL). The aqueous phase was again extracted with ethyl acetate (3 × 20 mL), washed with brine (20 mL), and dried over sodium sulfate. After removal of the solvent in vacuo, the amine **21** (116 mg, 610 µmol, 54% over two steps) was obtained as a brown liquid without further purification.

TLC: R_f = 0.22 (DCM/MeOH = 9/1) [UV/Ninhydrin].

^{1}H-NMR (500 MHz, DMSO-d$_6$): δ [ppm] = 3.89 (d, ^4J = 0.9 Hz, 2 H), 6.96 (dt, ^3J = 3.7 Hz, ^4J = 0.9 Hz, 1 H), 7.22 (ddd, ^3J = 7.3 Hz, ^3J = 4.9 Hz, ^4J = 1.1 Hz, 1 H), 7.61 (d, ^3J = 3.7 Hz, 1 H), 7.78 (*virt*. td, $^3J \approx {}^3J$ = 7.8 Hz, ^4J = 1.7 Hz, 1 H), 7.83 (*virt*. dt, ^3J = 8.1 Hz, $^4J \approx {}^5J$ = 1.1 Hz, 1 H), 8.04 (bs, 1 H), 8.49 (ddd, ^3J = 4.9 Hz, ^4J = 1.7 Hz, ^5J = 1.1 Hz, 1 H).

^{13}C{^{1}H}NMR (125 MHz, DMSO-d$_6$): δ [ppm] = 41.2 (s), 118.2 (s), 121.9 (s), 124.3 (s), 124.9 (s), 137.0 (s), 142.1 (s), 149.3 (s), 151.6 (s), 152.2 (s).

HR-MS (ESI): calc. for $[C_{10}H_8NS]^+$ ($[M-NH_2]^+$): 174.0378, found 174.0372; calc. for $[C_{10}H_{11}N_2S]^+$ ($[M+H]^+$): 191.0638, found 191.0637.

Amino(((5-(pyridin-2-yl)thiophen-2-yl)methyl)amino)methaniminium 2,2,2-trifluoroacetate (D13)

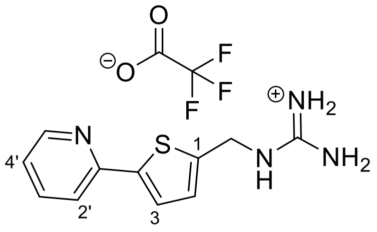

$C_{13}H_{13}F_3N_4O_2S$
346.33 g/mol
D13

The general procedure II was followed using amine **21** (70.4 mg, 370 µmol, 1.00 eq.) as described. After purification via preparative HPLC (column 1, method 8, λ = 271 nm), the guanidine **D13** (31.6 mg, 265 µmol, 72%) was obtained as an off-white solid.

^{1}H-NMR (500 MHz, DMSO-d$_6$): δ [ppm] = 4.59 (d, ^3J = 6.2 Hz, 2 H), 7.09 (d, ^3J = 3.8 Hz, 1 H), 7.27 (*virt*. dq, ^3J = 7.5 Hz, ^3J = 4.9 Hz, ^4J = 0.9 Hz, 1 H), 7.68 (d, ^3J = 3.8 Hz, 1 H), 7.83 (*virt*. dt, $^3J \approx {}^3J$ = 7.5 Hz, ^4J = 1.7 Hz, 1 H), 7.89 (*virt*. dt, $^3J \approx {}^3J$ = 7.5 Hz, ^5J = 1.0 Hz, 1 H), 8.04 (bs, 1 H), 8.51 (ddd, ^3J = 4.9 Hz, ^4J = 1.7 Hz, ^5J = 1.0 Hz, 1 H).

^{13}C{^{1}H}NMR (125 MHz, DMSO-d$_6$): δ [ppm] = 118.5 (s), 122.5 (s), 124.9 (s), 127.3 (s), 137.3 (s), 142.3 (s), 144.2 (s), 149.4 (s), 151.6 (s), 156.6 (s). The ^{13}C signal of the CH$_2$ group overlaps with the solvent signal and can, therefore, not be localized.

^{19}F-NMR (375 MHz, DMSO-d$_6$): δ [ppm] = - 74.1 (s).

HR-MS (ESI): calc. for $[C_{10}H_8NS]^+$ ($[M\text{-}CH_5N_3]^+$): 174.0378, found 174.0373; calc. for $[C_{11}H_{13}N_4S]^+$ ($[M+H]^+$): 233.0855, found 233.0855.

Ethyl 5-(4-bromophenyl)furan-2-carboxylate (22)

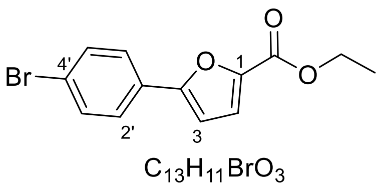

$C_{13}H_{11}BrO_3$
295.13 g/mol
22

According to a modified literature procedure[56], methyl-5-bromofuran-2-carboxylate (254 mg, 1.20 mmol, 1.20 eq.) was dissolved in 1,2-dimethoxyethane (2 mL, 600 mM), tetrakis(tri-phenylphosphine)palladium(0) (58.4 mg, 50.0 µmol, 5 mol%) and sodium carbonate solution (2 M in water, 1 mL) were added, degassed, and the reaction mixture was allowed to stir for 15 min at RT. 4-Bromophenylboronic acid (207 mg, 1.00 mmol, 1.00 eq.) was dissolved in ethanol (2 mL), degassed and added. After stirring for 92 h at 90 °C, the reaction mixture was extracted with dichloromethane (3 × 50 mL), and the organic layer was washed with brine (50 mL) and dried over sodium sulfate. The solvent was removed, and the ester 22 was obtained after purification via column chromatography (P/Et$_2$O = 98/2→95/5) as (126 mg, 425 µmol, 43%).

TLC: R_f = 0.15 (P/Et$_2$O=98/2) [UV].

^{1}H-NMR (500 MHz, CDCl$_3$: δ [ppm] = 1.40 (t, ^3J = 7.1 Hz, 3 H), 4.39 (q, ^3J = 7.1 Hz, 2 H), 6.74 (d, ^3J = 3.6 Hz, 1 H), 7.23 (d, ^3J = 3.6 Hz, 1 H), 7.55 (dq$_{A\text{-}B}$, ^3J = 8.6 Hz, $J_{A\text{-}B}$ = 4.5 Hz, 2 H), 7.65 (dq$_{A\text{-}B}$, ^3J = 8.6 Hz, $J_{A\text{-}B}$ = 4.5 Hz, 2 H).

^{13}C{^{1}H}NMR (125 MHz, CDCl$_3$): δ [ppm] = 14.5 (s), 61.2 (s), 107.4 (s), 119.9 (s), 123.1 (s), 126.4 (s), 128.6 (s), 132.2 (s), 144.3 (s), 156.4 (s), 158.9 (s).

HR-MS (ESI): calc. for $[C_{13}H_{12}{}^{79}BrO_3]^+$ ($[M+H]^+$): 294.9964, found 294.9959; calc. for $[C_{13}H_{12}{}^{81}BrO_3]^+$ ($[M+H]^+$): 296.9944, found 296.9938.

Analytical data are in accordance with the literature.[59]

5-(4-Bromophenyl)-*N*-carbamimidoylfuran-2-carboxamide (D15)

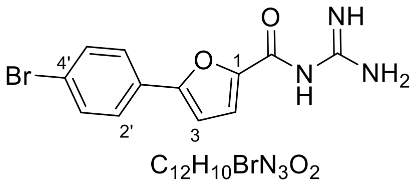

$C_{12}H_{10}BrN_3O_2$
308.14 g/mol
D15

According to a modified literature procedure[60], the ester **22** (90.0 mg, 305 µmol, 1.00 eq.) was dissolved in *N*, *N*-dimethylformamide (2 mL, 153 mM), guanidine (2 M in MeOH, 915 µL, 1.83 mmol, 6.00 eq.) was added and the reaction was allowed to stir 90 min at RT. Subsequently, brine (10 mL) was added, and the aqueous phase was extracted with ethyl acetate (3 × 30 mL), washed with brine (2 × 20 mL), and dried over sodium sulfate. The solvent was removed and the acylguanidine **D15** was obtained after purification via column chromatography (DCM/MeOH=95/5 with 0.1 v/v% TEA) as a light yellow solid (71.9 mg, 233 µmol, 77%).

TLC: R_f = 0.68 (DCM/MeOH=9/1) [UV].

^{1}H-NMR (500 MHz, DMSO-d$_6$): δ [ppm] = 7.01 (d, ^3J = 3.6 Hz, 2 H), 7.07 (d, ^3J = 3.6 Hz, 2 H), 7.66 (dq$_{A\text{-}B}$, ^3J = 8.7 Hz, $J_{A\text{-}B}$ = 4.4 Hz, 2 H), 7.70 (dq$_{A\text{-}B}$, ^3J = 8.7 Hz, $J_{A\text{-}B}$ = 4.4 Hz, 2 H).

^{13}C{^{1}H}NMR (125 MHz, DMSO-d$_6$): δ [ppm] = 108.4 (s), 115.9 (s), 121.2 (s), 125.9 (s), 129.2 (s), 132.0 (s), 153.0 (s), 162.9 (s). Two carbon signals are either overlapping or could not be resolved.

HR-MS (ESI): calc. for $[C_{11}H_6{}^{79}BrO_2]^+$ ($[M\text{-}CH_5N_3]^+$): 248.9551, found 248.9544; calc. for $[C_{11}H_6{}^{81}BrO_2]^+$ ($[M\text{-}CH_5N_3]^+$): 250.9531, found 250.9523.; calc. for $[C_{12}H_{11}{}^{79}BrN_3O_2]^+$ ($[M+H]^+$): 308.0029, found 308.0029; calc. for $[C_{12}H_{11}{}^{81}BrN_3O_2]^+$ ($[M+H]^+$): 310.0009, found 310.0006.

5-(4-Bromophenyl)furan-2-carbaldehyde (23)

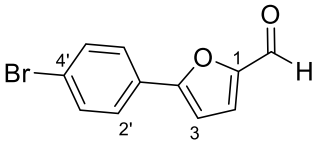

$C_{11}H_7BrO_2$
251.08 g/mol
23

According to a modified literature procedure[56], 1-bromo-4-iodobenzene (1.01 g, 3.50 mmol, 1.00 eq.), 5-formyl-2-furaneboronic acid (637 mg, 4.55 mmol, 1.30 eq.), bis(tri-phenylphosphine)palladium(II)chloride (123 mg, 175 µmol, 5 mol%) and sodium carbonate solution (2 M in water, 1.05 mL) were dissolved in 1,2-dimethoxyethane (2.1 mL, 1.67 M), degassed, and the reaction mixture was stirred for 5 min at 90 °C and further 40 min at 75 °C. After extraction with ethyl acetate (3 × 50 mL), the organic layer was washed with brine (50 mL) and dried over sodium sulfate. The solvent was removed, and the aldehyde **23** (724 mg, 2.88 µmol, 82%). was obtained after purification via column chromatography (P/EtOAc = 9/1→8/2→7/3) as a light brown solid.

TLC: R_f = 0.46 (P/EtOAc = 8/2) [UV/DNP].

^{1}H-NMR (500 MHz, DMSO-d$_6$): δ [ppm] = 7.35 (d, ^3J = 3.8 Hz, 2 H), 7.66 (d, ^3J = 3.8 Hz, 2 H), 7.72 (dq$_{A\text{-}B}$, ^3J = 8.7 Hz, $J_{A\text{-}B}$ = 4.5 Hz, 2 H), 7.82 (dq$_{A\text{-}B}$, ^3J = 8.7 Hz, $J_{A\text{-}B}$ = 4.5 Hz, 2 H), 9.61 (s, 1 H).

^{13}C{^{1}H}NMR (125 MHz, DMSO-d$_6$): δ [ppm] = 109.5 (s), 123.1 (s), 125.4 (s), 127.0 (s), 127.9 (s), 132.3 (s), 151.9 (s), 157.1 (s), 178.1 (s).

HR-MS (ESI): calc. for $[C_{11}H_8{}^{79}BrO_2]^+$ ($[M+H]^+$): 250.9702, found 250.9697, calc. for $[C_{11}H_8{}^{81}BrO_2]^+$ ($[M+H]^+$): 252.9682, found 252.9674.

Analytical data are in accordance with the literature.[56]

(5-(4-Bromophenyl)furan-2-yl)methanol (24)

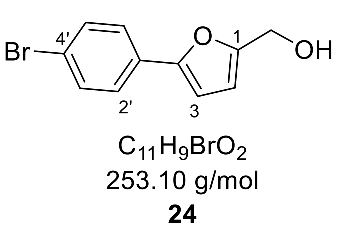

$C_{11}H_9BrO_2$
253.10 g/mol
24

The aldehyde **23** (650 mg, 2.59 mmol, 1.00 eq.) was dissolved in methanol (15 mL, 173 mM) and cooled to 0°C. Sodium borohydride (58.8 mg, 1.55 mmol, 0.60 eq.) was added portion-wise and stirred for one hour. The reaction was extracted with ethyl acetate (3 × 50 mL), the organic layer was washed with hydrochloric acid (aqueous, 1 M, 10 ML), sodium carbonate solution (aqueous, saturated, 50 mL), brine (aqueous, saturated, 50 mL) and dried over sodium sulfate. After removing the solvent in vacuo, the alcohol **24** was used without further purification and characterization.

TLC: R_f = 0.33 (P/EtOAc = 8/2) [UV].

^{1}H-NMR (500 MHz, $CDCl_3$: δ [ppm] = 4.66 (s, 2 H), 6.38 (d, ^3J = 3.3 Hz, 2 H), 6.61 (d, ^3J = 3.3 Hz, 2 H), 7.50 (dq$_{A-B}$, ^3J = 8.6 Hz, J_{A-B} = 4.3 Hz, 2 H), 7.54 (dq$_{A-B}$, ^3J = 8.6 Hz, J_{A-B} = 4.3 Hz, 2 H).

HR-MS (ESI): calc. for $[C_{11}H_8{}^{79}BrO]^+$ ($[M-OH]^+$) 234.9759, found 234.9753; calc. for $[C_{11}H_8{}^{81}BrO]^+$ ($[M-OH]^+$) 236.9738, found 236.9732.

Analytical data are in accordance with the literature.[61]

2-(4-Bromophenyl)-5-(chloromethyl)furan (25)

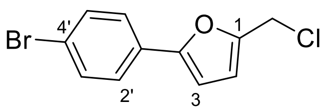

$C_{11}H_8BrClO$
271.54 g/mol
25

The alcohol **24** (200 mg, 790 µmol, 1.00 eq.) was dissolved in dichloromethane (6 mL, 132 mM) and cooled to 0°C. Thionyl chloride (113 mg, 69.1 µL, 948 µmol, 1.20 eq.) was added slowly, and the reaction was stirred for 30 min at RT. The reaction was quenched with sodium bicarbonate (aqueous, saturated, 10 mL) and extracted with ethyl acetate (3 × 50 mL). The organic layer was washed with brine (50 mL) and dried over sodium sulfate. After removing the solvent, chloride **25** (205 mg, 753 µmol, 95%) was obtained as a greyish solid and converted without further purification and characterization.

TLC: R_f = 0.86 (P/EtOAc=1/1) [UV].

^{1}H-NMR (500 MHz, $CDCl_3$: δ [ppm] = 4.64 (s, 2 H), 6.46 (d, ^3J = 3.5 Hz, 2 H), 6.61 (d, ^3J = 3.5 Hz, 2 H), 7.50-7.55 (m, 4 H).

HR-MS (ESI): calc. for $[C_{11}H_8{}^{79}BrO]^+$ ($[M-Cl]^+$) 234.9759, found 234.9754; calc. for $[C_{11}H_8{}^{81}BrO]^+$ ($[M-Cl]^+$) 236.9738, found 236.9733.

1-((5-(4-Bromophenyl)furan-2-yl)methyl)-1*H*-imidazol-2-aminium 2,2,2-trifluoro-acetate (D16)

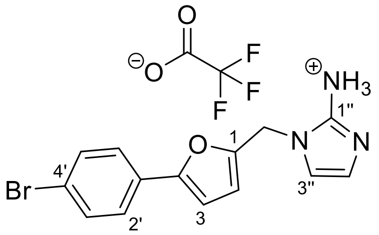

$C_{16}H_{13}BrF_3N_3O_3$
432.20 g/mol
D16

The chloride **25** (190 mg, 700 µmol, 1.00 eq.) was dissolved in *N*, *N*-dimethylformamide (7 mL, 100 mM) and 1*H*-imidazol-2-amine (291 mg, 3.59 mmol, 5.00 eq.) was added. The reaction mixture was stirred for 44 h at RT. Toluene (15 mL) was added and the solvent was removed in vacuo. The residue was dissolved in MeOH (2 mL) and precipitated by adding excess of diethyl ether. The filtrate was concentrated in vacuo, dissolved in hydrochloric acid (1 M, aqueous, 20 mL), basified with sodium carbonate solution (saturated, aqueous) and extracted with dichloromethane (3 × 25 mL). After removal of the solvent in vacuo, the crude product was purified by normal phase column chromatography (DCM/MeOH = 9/1 with 0.1 v/v% TEA) and subsequent preparative HPLC (column 1, method 4, λ = 290 nm). The 2-aminoimidazole derivative **D16** was obtained as a yellow solid (*yield could not be determined*).

^{1}H-NMR (500 MHz, DMSO-d_6): δ [ppm] = 5.19 (s, 2 H), 6.62 (d, ^3J = 3.3 Hz, 1 H), 6.96 (d, ^3J = 2.5 Hz, 1 H), 7.01 (d, ^3J = 3.3 Hz, 1 H), 7.08 (d, ^3J = 2.5 Hz, 1 H), 7.62-7.64 (m, 4 H), 7.83 (bs, 2 H).

^{13}C{^{1}H}NMR (125 MHz, DMSO-d_6): δ [ppm] = 41.2 (s), 107.5 (s), 112.0 (s), 113.3 (s), 116.3 (s), 120.9 (s), 125.4 (s), 129.0 (s), 132.0 (s), 146.2 (s), 148.3 (s), 152.5 (s).

^{19}F-NMR (375 MHz, DMSO-d_6): δ [ppm] = - 73.5 (s).

HR-MS (ESI): calc. for $[C_{11}H_8{}^{79}BrO]^+$ ($[M-C_3H_5N_3]^+$): 234.9759, found 234.9752; calc. for $[C_{11}H_8{}^{79}BrO]^+$ ($[M-C_3H_5N_3]^+$): 236.9738, found 236.97331; calc. for $[C_{14}H_{13}{}^{79}BrN_3O]^+$ ($[M+H]^+$): 318.0237, found 318.0236; calc. for $[C_{14}H_{13}{}^{81}BrN_3O]^+$ ($[M+H]^+$): 320.0216, found 320.0213.

1-(5-(4-Bromophenyl)furan-2-yl)ethan-1-amine (26)

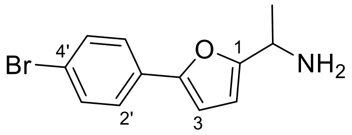

$C_{12}H_{12}BrNO$
266.14 g/mol
26

The general procedure I was followed using 4-bromoaniline (275 mg, 1.60 mmol, 1.00 eq.) and 1-(furan-1-yl)ethan-1'-amine (889 mg, 8.00 mmol, 5.00 eq.) as described. The amine **26** was obtained after purification via column chromatography (EtOAc) as a light orange oil (187 mg, 702 mmol, 44%).

TLC: R_f = 0.12 (EtOAc) [UV].

¹H-NMR (500 MHz, DMSO-d6): δ [ppm] = 1.33 (d, ^3J = 6.7 Hz, 3 H), 4.00 (q, ^3J = 6.7 Hz 1 H), 6.60 (dd, ^3J = 3.4 Hz, ^4J = 0.9 Hz, 1 H), 6.90 (d, ^3J = 3.4 Hz, 1 H), 7.58 (dq$_{A\text{-}B}$, ^3J = 8.8 Hz, $J_{A\text{-}B}$ = 4.2 Hz, 2 H), 7.62 (dq$_{A\text{-}B}$, ^3J = 8.8 Hz, $J_{A\text{-}B}$ = 4.2 Hz, 2 H).

HR-MS (ESI): calc. for $[C_{12}H_{10}{}^{79}BrO]^+$ ($[M-NH_2]^+$): 248.9915, found 248.9912; calc. $[C_{12}H_{10}{}^{79}BrO]^+$ ($[M-NH_2]^+$): 250.9895, found 250.9887.

Full characterization could not be performed due to the oxidation sensitivity of the product.

Amino((1-(5-(4-bromophenyl)furan-2-yl)ethyl)amino)methaniminium 2,2,2-trifluoro-acetate (D17)

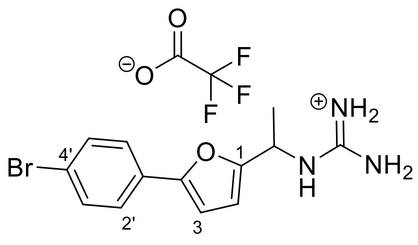

$C_{15}H_{15}BrF_3N_3O_3$
422.20 g/mol
D17

The general procedure II was followed using amine **26** (150 mg, 564 µmol, 1.05 eq.) as described. The crude product was dissolved in a minimal amount of methanol and precipitated by adding an excess of diethyl ether. The guanidine **D17** was obtained after purification via preparative HPLC (column 2, method 1, λ = 291 nm) as a light yellow solid (105 mg, 249 µmol, 44%).

¹H-NMR (400 MHz, DMSO-d6): δ [ppm] = 1.51 (d, ^3J = 6.8 Hz, 3 H), 4.92 (*virt.* quin, $^3J \approx {}^3J$ = 7.2 Hz, 1 H), 6.49 (d, ^3J = 3.4 Hz, 1 H), 6.99 (d, ^3J = 3.4 Hz, 1 H), 7.61-7.66 (m, 4 H), 8.06 (*virt.* t, $^3J \approx {}^3J$ = 8.1 Hz, 1 H).

¹³C{¹H}NMR (125 MHz, DMSO-d6): δ [ppm] = 19.1 (s), 44.6 (s), 107.4 (s), 108.6 (s), 120.6 (s), 125.3 (s), 129.3 (s), 131.9 (s), 151.5 (s), 154.4 (s), 156.1 (s).

¹⁹F-NMR (375 MHz, DMSO-d6) : δ [ppm] = - 73.4 (s).

HR-MS (ESI): calc. for $[C_{12}H_{10}{}^{79}BrO]^+$ ($[M-CH_5N_3]^+$): 248.9915, found 248.9913; calc. $[C_{12}H_{10}{}^{81}BrO]^+$ ($[M-CH_5N_3]^+$): 250.9895, found 250.9887.

2-(5-(4-Bromophenyl)furan-2-yl)ethan-1-amine (27)

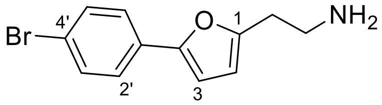

$C_{12}H_{12}BrNO$
266.14 g/mol
27

The general procedure I was followed using 4-bromoaniline (138 mg, 800 µmol, 1.00 eq.) and 2-(furan-2-yl)ethan-1-amine (356 mg, 3.20 mmol, 4.00 eq.) as described. The amine **27** was obtained after purification via column chromatography (DCM/MeOH = 95/5 with 1 v/v% TEA) as a brown oil (129 mg, 484 mmol, 61%).

TLC: R_f = 0.03 (DCM/MeOH = 95/5) [UV].

^{1}H-NMR (500 MHz,DMSO-d$_6$): δ [ppm] = 2.74 (t, ^3J = 7.0 Hz, 2 H), 2.86 (t, ^3J = 7.0 Hz, 2 H), 6.27 (d, ^3J = 3.3 Hz, 1 H), 6.91 (d, ^3J = 3.3 Hz, 1 H), 7.56 - 7.61 (m, 4 H).

^{13}C{^{1}H}NMR (125 MHz, DMSO-d$_6$): δ [ppm] = 31.7 (s), 40.3 (s), 107.6 (s), 108.4 (s), 119.8 (s), 125.0 (s), 129.8 (s), 131.8 (s), 150.4 (s), 154.7 (s).

HR-MS (ESI): calc. for $[C_{12}H_{10}{}^{79}BrO]^+$ ($[M-NH_2]^+$): 248.9915, found 248.9913; calc. for $[C_{12}H_{10}{}^{81}BrO]^+$ ($[M-NH_2]^+$): 250.9895, found 250.9888.

Amino((2-(5-(4-bromophenyl)furan-2-yl)ethyl)amino)methaniminium 2,2,2-trifluoro-acetate (D18)

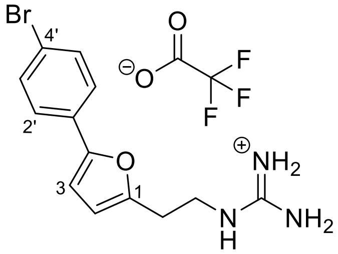

$C_{15}H_{15}BrF_3N_3O_3$
422.20 g/mol
D18

The general procedure II was followed using amine **27** (118 mg, 443 µmol, 1.00 eq.) as described. The crude product was dissolved in a minimal amount of methanol and precipitated by adding an excess of diethyl ether. The guanidine **D18** was obtained after purification via preparative HPLC (column 2, method 1, λ = 294 nm) as a beige solid (61.2 mg, 145 µmol, 33%).

^{1}H-NMR (500 MHz, DMSO-d$_6$): δ [ppm] = 2.90 (t, ^3J = 7.1 Hz, 2 H), 3.47 (*virt.* q, $^3J \approx {}^3J$ = 6.7 Hz, 2 H), 6.34 (d, ^3J = 3.3 Hz, 1 H), 6.99 (d, ^3J = 3.3 Hz, 1 H), 7.45 (t, ^3J = 5.2 Hz, 1 H), 7.60 (dq$_{A-B}$, ^3J = 6.6 Hz, J_{A-B} = 4.1 Hz, 2 H), 7.63 (dq$_{A-B}$, ^3J = 6.6 Hz, J_{A-B} = 4.1 Hz, 2 H).

^{13}C{^{1}H}NMR (125 MHz, DMSO-d$_6$): δ [ppm] = 27.5 (s), 107.6 (s), 109.2 (s), 120.0 (s), 125.1 (s), 129.6 (s), 131.8 (s), 150.9 (s), 152.7 (s), 156.7 (s). The ^{13}C signal of the C-2'' group overlaps with the solvent signal and can, therefore, not be localized.

^{19}F-NMR (375 MHz, DMSO-d$_6$): δ [ppm] = - 73.5 (s).

HR-MS (ESI): calc. for $[C_{12}H_{10}{}^{79}BrO]^+$ ($[M-CH_5N_3]^+$): 248.9915, found 248.9910; calc. for $[C_{12}H_{10}{}^{81}BrO]^+$ ($[M-CH_5N_3]^+$): 250.9895, found 250.9886; calc. for $[C_{13}H_{15}{}^{79}BrN_3O]^+$ ($[M+H]^+$): 308.0393, found 308.0393; calc. for $[C_{13}H_{15}{}^{81}BrN_3O]^+$ ($[M+H]^+$): 310.0373, found 310.0366.

Probe Synthesis

Methyl-4-(5-((hydroxyimino)methyl)furan-2-yl)benzoate (28)

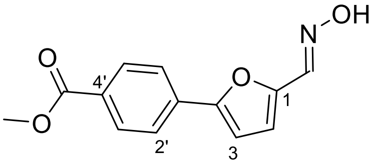

$C_{13}H_{11}NO_4$
245.23 g/mol
28

The General Procedure III was followed using methyl-4-(5-formylfuran-2-yl)benzoate (899 mg, 3.71 mmol, 1.00 eq.) as described. The crude product was taken up in a minimal amount of water/ethyl acetate; the aqueous layer was extracted with ethyl acetate (3 × 15 mL), and the organic layer was washed with brine (1 × 15 mL), dried with sodium sulfate and concentrated in vacuo. The crude product was directly used in the following reaction without further purification and characterization.

TLC: R_f = 0.73 (H/EtOAc = 1/1) [UV].

Methyl 4-(5-(aminomethyl)furan-2-yl)benzoate (29)

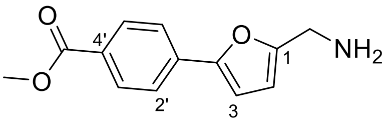

$C_{13}H_{13}NO_3$
231.25 g/mol
29

The General Procedure IV was followed using oxime **28** (912 mg, 3.72 mmol, 1.00 eq.) as described. After purification via column chromatography (DCM/MeOH = 95/5), the amine **29** was obtained as an orange oil (51.8 mg, 193 mmol, 54% over two steps).

TLC: R_f = 0.3 (DCM/MeOH = 9/1) [UV/Ninhydrin].

^{1}H-NMR (500 MHz, DMSO-d_6): δ [ppm] = 3.75 (s, 2 H), 3.85 (s, 3 H), 6.39 (d, ^3J = 3.3 Hz, 1 H), 7.07 (d, ^3J = 3.3 Hz, 1 H), 7.80 (dq$_{A\text{-}B}$, ^3J = 8.6 Hz, $J_{A\text{-}B}$ = 4.3 Hz, 1 H), 7.98 (dq$_{A\text{-}B}$, ^3J = 8.6 Hz, $J_{A\text{-}B}$ = 4.3 Hz, 2 H).

^{13}C{^{1}H}NMR (125 MHz, DMSO-d_6): δ [ppm] = 52.2 (s), 107.9 (s), 109.4 (s), 123.0 (s), 127.5 (s), 129.9 (s), 134.7 (s), 150.4 (s), 159.1 (s), 165.9 (s). The ^{13}C signal of the CH_2 group overlaps with the solvent signal and can, therefore, not be localized.

HR-MS (ESI): calc. for $[C_{13}H_{11}O_3]^+$ ($[M\text{-}NH_2]^+$): 215.0708, found 215.0705.
Analytical data are in accordance with the literature.[53]

Amino(((5-(4-(methoxycarbonyl)phenyl)furan-2-yl)methyl)amino)methaniminiumchloride (30)

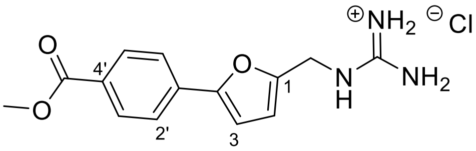

$C_{14}H_{16}ClN_3O_3$
309.75 g/mol
30

The general procedure II was followed using amine **29** (467 mg, 2.02 mmol, 1.00 eq.) as described. The crude product was dissolved in a minimal amount of methanol and precipitated by adding an excess of diethyl ether. The guanidine **30** was obtained as a brown solid (362 mg, 1.03 mol, 66%).

^{1}H-NMR (500 MHz, DMSO-d_6): δ [ppm] = 3.16 (s, 3 H), 4.50 (d, ^3J = 5.8 Hz, 2 H), 6.57 (d, ^3J = 3.4 Hz, 1 H), 7.14 (d, ^3J = 3.4 Hz, 1 H), 7.82 (dq$_{A\text{-}B}$, ^3J = 8.4 Hz, $J_{A\text{-}B}$ = 4.3 Hz, 1 H), 7.98 (dq$_{A\text{-}B}$, ^3J = 8.4 Hz, $J_{A\text{-}B}$ = 4.3 Hz, 2 H), 8.07 (t, ^3J = 5.8 Hz, 1 H).

^{13}C{^{1}H}NMR (125 MHz, DMSO-d_6): δ [ppm] = 37.8 (s), 52.2 (s), 109.3 (s), 110.6 (s), 123.3 (s), 128.1 (s), 130.0 (s), 134.1 (s), 151.5 (s), 151.8 (s), 157.0 (2 s). 165.9 (s).

HR-MS (ESI): calc. for $[C_{13}H_{11}O_3]^+$ ($[M-CH_5N_3]^+$): 215.0708, found 215.0698; calc. for $[C_{14}H_{16}N_3O_3]^+$ ($[M+H]^+$): 274.1186, found 274.1180.

Amino(((5-(4-carboxyphenyl)furan-2-yl)methyl)amino)methaniminium chloride (31)

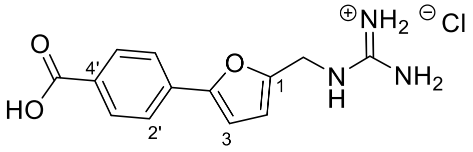

$C_{13}H_{14}ClN_3O_3$
295.72 g/mol
31

The ester **30** (104 mg, 381 µmol, 1.00 eq.) was dissolved in THF / MeOH (1 : 1, 5 mL), and the flask was flushed with argon. A solution of lithium hydroxide (aqueous, 1 M, 1.00 mL) was added, and the reaction mixture was stirred for 3 h. Afterwards, the solvent was evaporated, the residue was dissolved in hydrochloric acid (aqueous, 4 M, 3 mL), and the guanidine **31** was obtained as a brownish solid after filtration. This compound was converted without further purification and characterization.

^{1}H-NMR (500 MHz, DMSO-d$_6$): δ [ppm] = 4.49 (d, ^3J = 5.8 Hz, 2 H), 6.55 (d, ^3J = 3.4 Hz, 1 H), 7.12 (d, ^3J = 3.4 Hz, 1 H), 7.80 (d, ^3J = 8.5 Hz, 1 H), 8.00 (d, ^3J = 8.5 Hz, 2 H), 8.16 (bs, 1 H).

HR-MS (ESI): calc. for $[C_{12}H_9O_3]^+$ ($[M-CH_5N_3$): 201.0552, found 201.0542; calc. for $[C_{13}H_{14}N_3O_3]^+$ ($[M+H$): 260.1030, found 260.1025.

Amino(((5-(4-((2-(3-(but-3-yn-1-yl)-3*H*-diazirin-3-yl)ethoxy)carbonyl)phenyl)furan-2-yl)-methyl)amino)methaniminium 2,2,2-trifluoroacetate (L15-P)

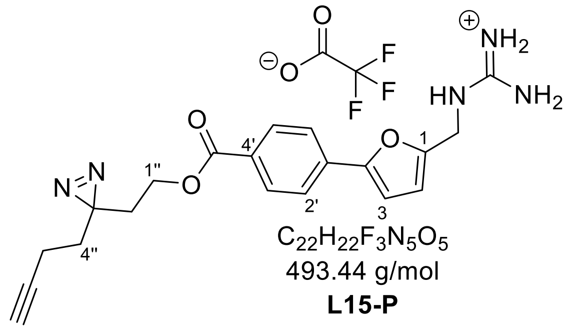

$C_{22}H_{22}F_3N_5O_5$
493.44 g/mol
L15-P

To a solution of guanidine salt, **31** (50.0 mg, 169 µmol, 1.00 eq.) and 2-(3-(But-3-yn-1-yl)-3*H*-diazirin-3-yl)ethan-1-ol (30.4 mg, 220 µmol, 1.30 eq.) in *N*, *N*-dimethylformamide (1 mL, 169 mM), 4-(dimethylamino)pyridine (61.9 mg, 507 µmol, 3.00 eq.) and 1-Ethyl-3-(3-dimethylaminopropyl)-carbodiimide hydrochloride (55.1 mg, 287 µmol, 1.70 eq.) were added. The reaction mixture was stirred at RT for 20 h in the dark. Subsequently, the solvent was evaporated, and the residue was washed with toluene (2 × 5 mL). The diazirine was obtained after purification via preparative HPLC (column 1, method 5 in the dark) as a light yellow solid (23.4 mg, 47.4 µmol, 28% over two steps).

^{1}H-NMR (500 MHz, DMSO-d$_6$): δ [ppm] = 1.68 (t, ^3J = 7.3 Hz, 2 H), 1.90 (t, ^3J = 6.0 Hz, 2 H), 2.05 (*virt.* dt, $^3J \approx {}^3J$ = 7.3 Hz, ^4J = 2.8 Hz, 2 H), 2.84 (t, ^4J = 2.8 Hz, 2 H), 4.18 (t, ^3J = 6.0 Hz, 2 H), 4.50 (d, ^3J = 6.2 Hz, 2 H), 6.56 (d, ^3J = 3.4 Hz, 1 H), 7.15 (d, ^3J = 3.4 Hz, 1 H), 7.85 (dq$_{A\text{-}B}$, ^3J = 8.5 Hz, $J_{A\text{-}B}$ = 4.3 Hz, 2 H), 7.96 (bs, 1 H), 8.04 (dq$_{A\text{-}B}$, ^3J = 8.5 Hz, $J_{A\text{-}B}$ = 4.3 Hz, 2 H).

^{13}C{^{1}H}NMR (125 MHz, DMSO-d$_6$): δ [ppm] = 12.7 (s), 27.0 (s), 31.36 (s), 31.41 (s), 37.8 (s), 59.9 (s), 71.9 (s), 83.2 (s), 109.4 (s), 110.6 (s), 123.3 (s), 128.1 (s), 130.0 (s), 134.2 (s), 151.5 (s), 151.8 (s), 156.8 (s), 165.1 (s).

^{19}F-NMR (375 MHz, DMSO-d$_6$): δ [ppm] = - 73.5 (s).

HR-MS (ESI): calc. for $[C_{19}H_{17}O_3]^+$ ($[M-CH_5N_5$): 293.1178, found 293.1165; calc. for $[C_{19}H_{17}N_2O_3]^+$ ($[M-CH_5N3$): 321.1239, found 321.1227; calc. for $[C_{20}H_{22}N_5O_3]^+$ ($[M+H$): 380.1717, found 380.1708.

(5-(4-Bromophenyl)furan-2-yl)methanamine (1)

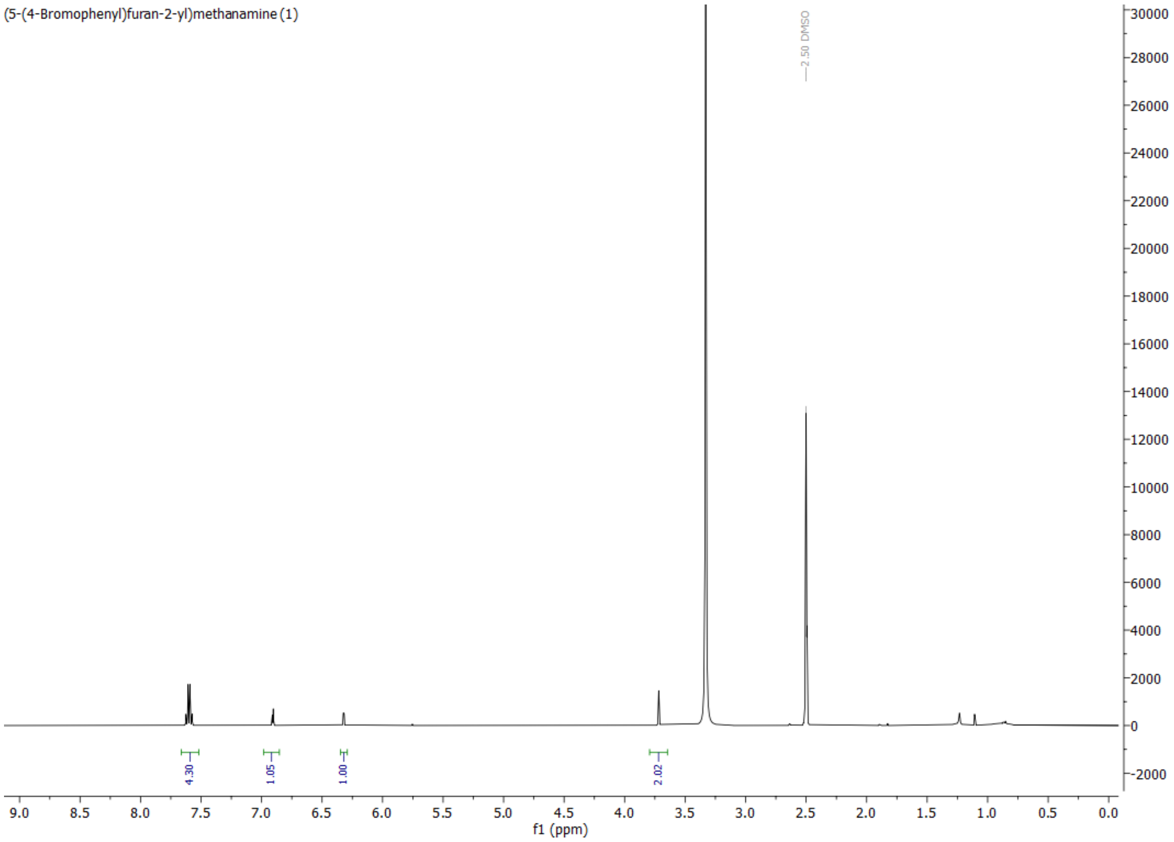

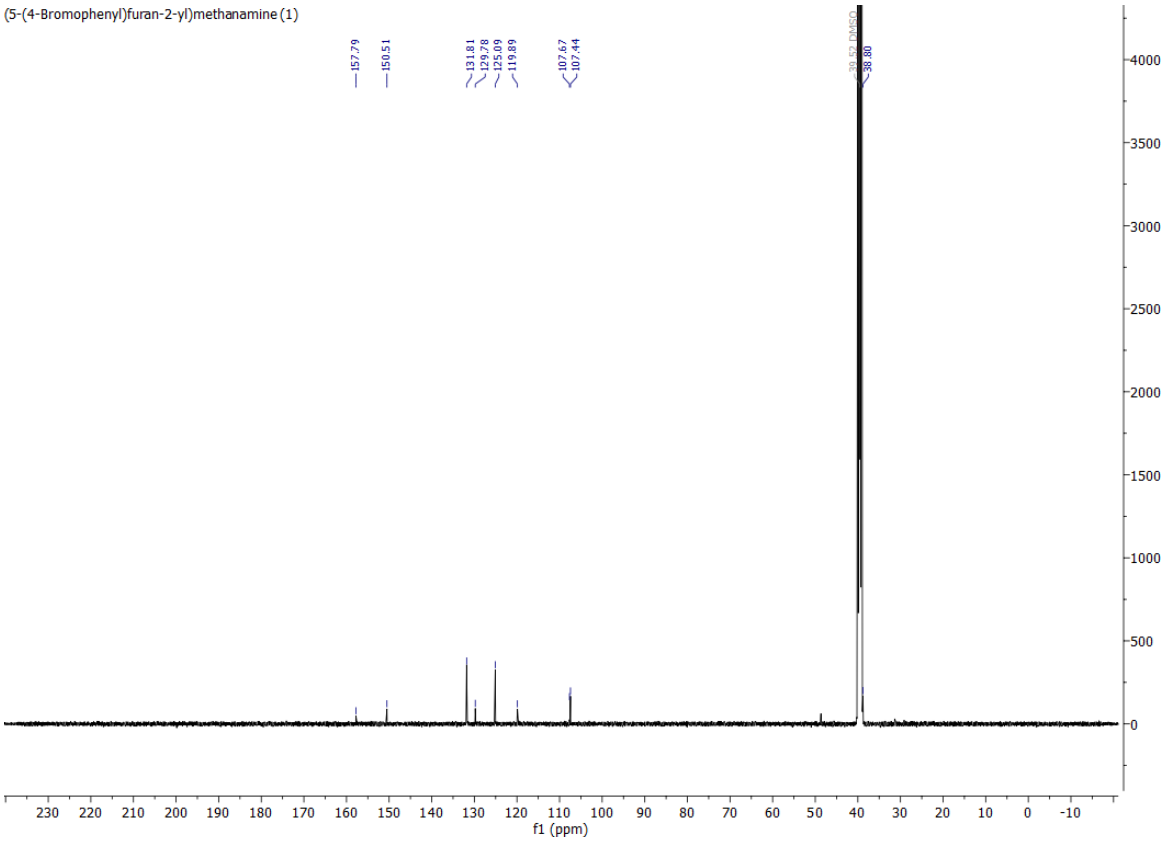

Amino(((5-(4-bromophenyl)furan-2-yl)methyl)amino)methaniminium 2,2,2-trifluoro-acetate (L15)

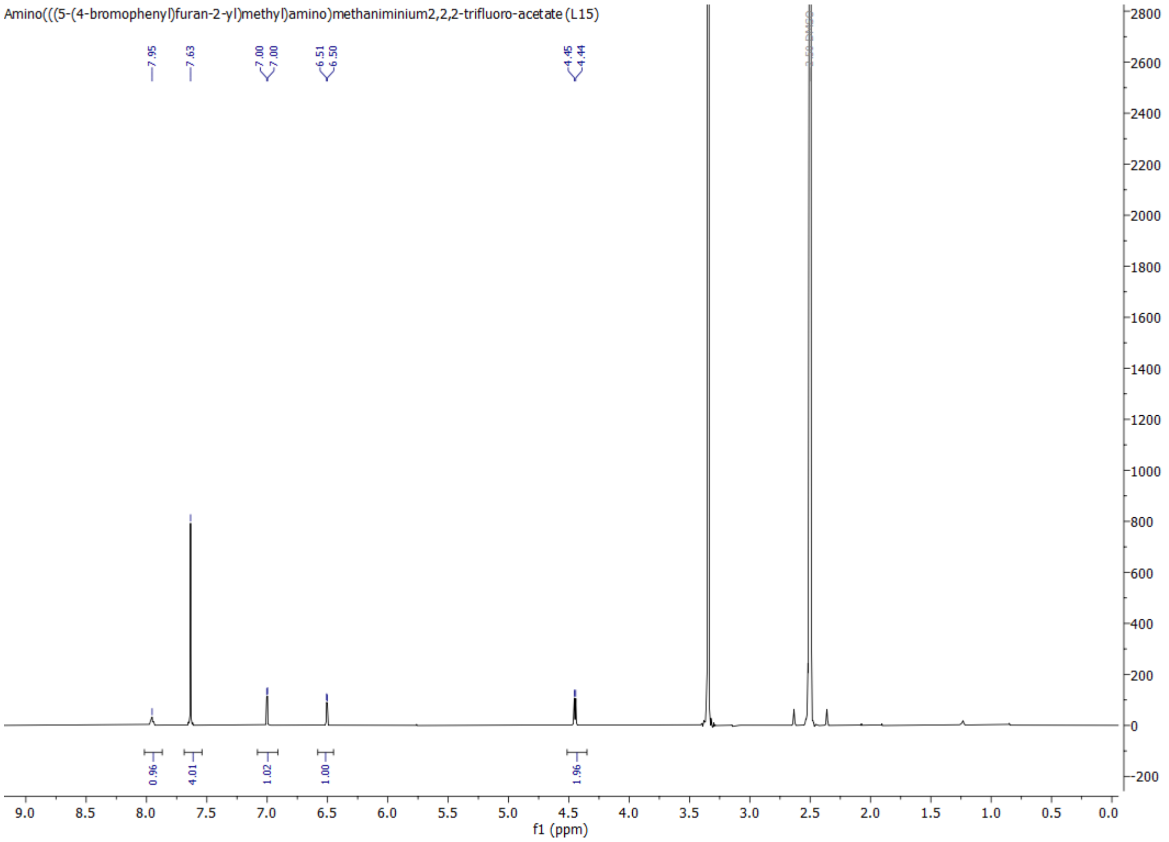

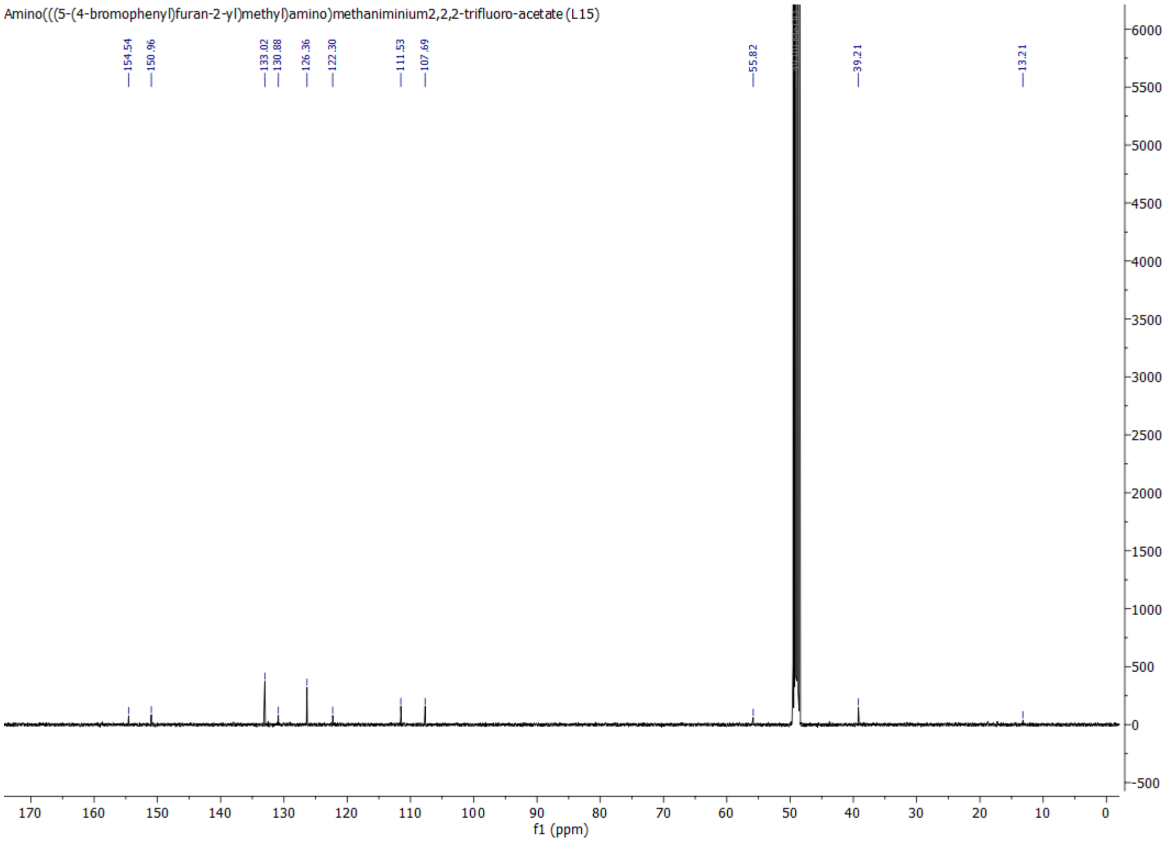

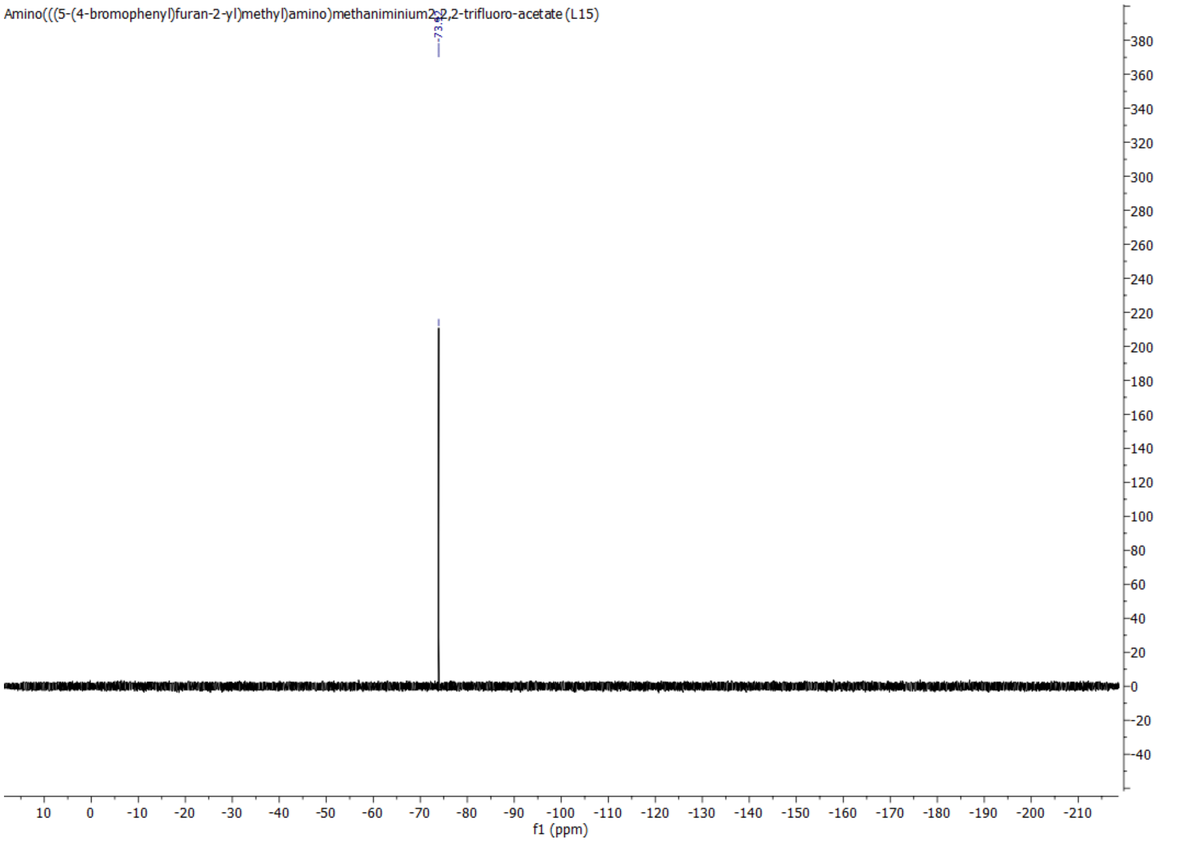

(5-(3-Bromophenyl)furan-2-yl)methanamine (2)

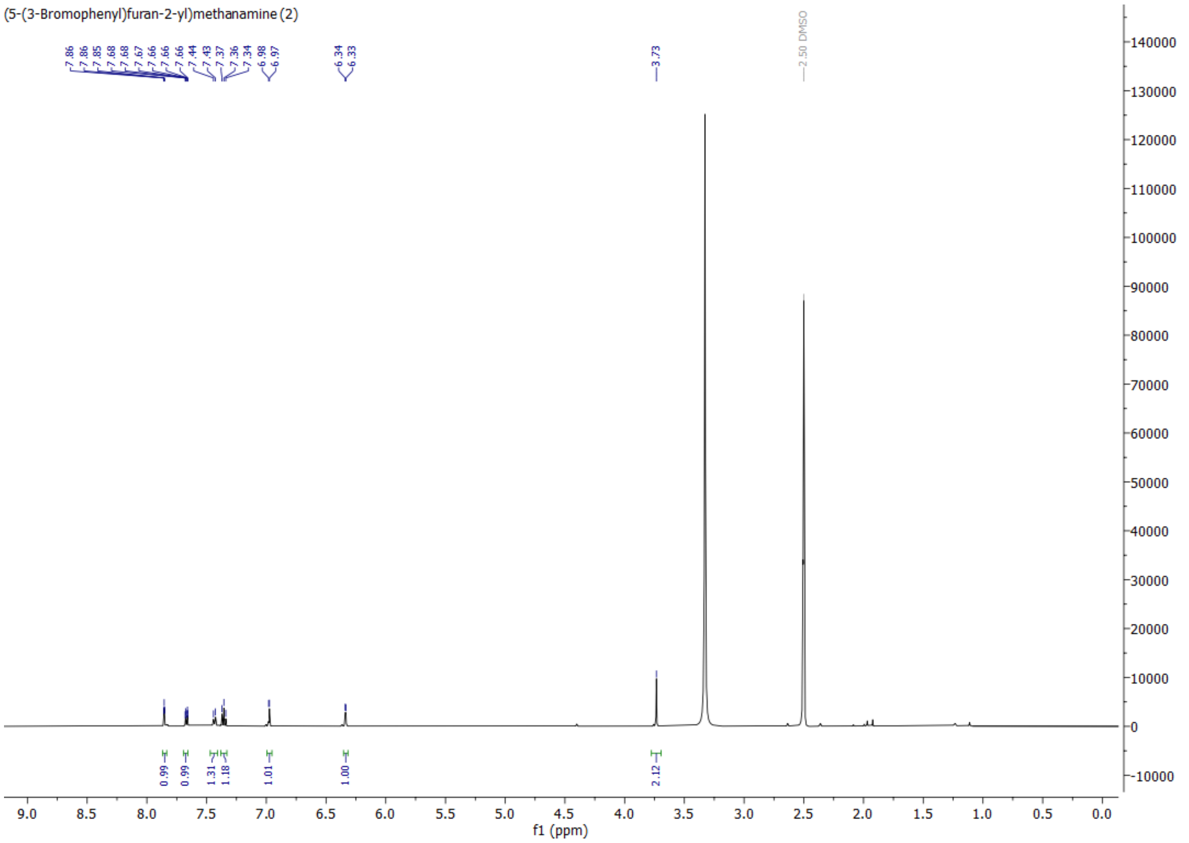

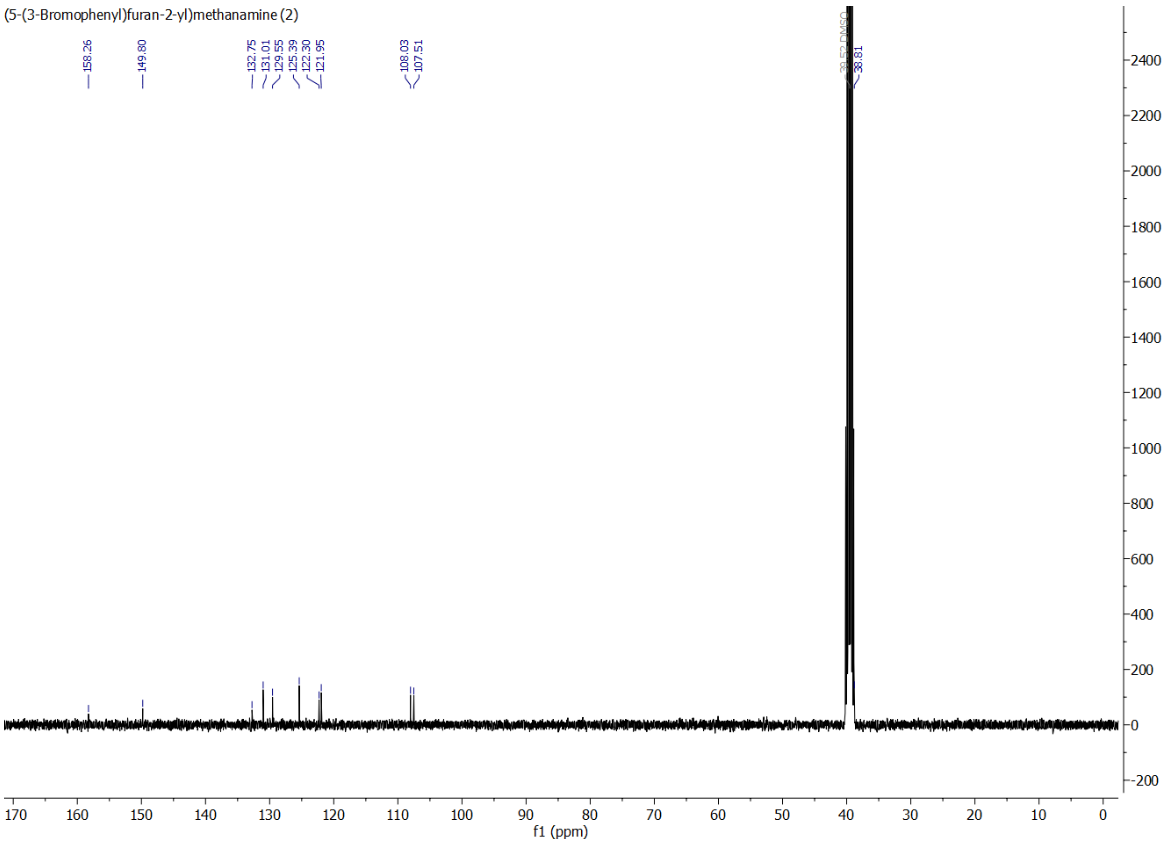

Amino(((5-(3-bromophenyl)furan-2-yl)methyl)amino)methaniminium 2,2,2-trifluoro-acetate (D01)

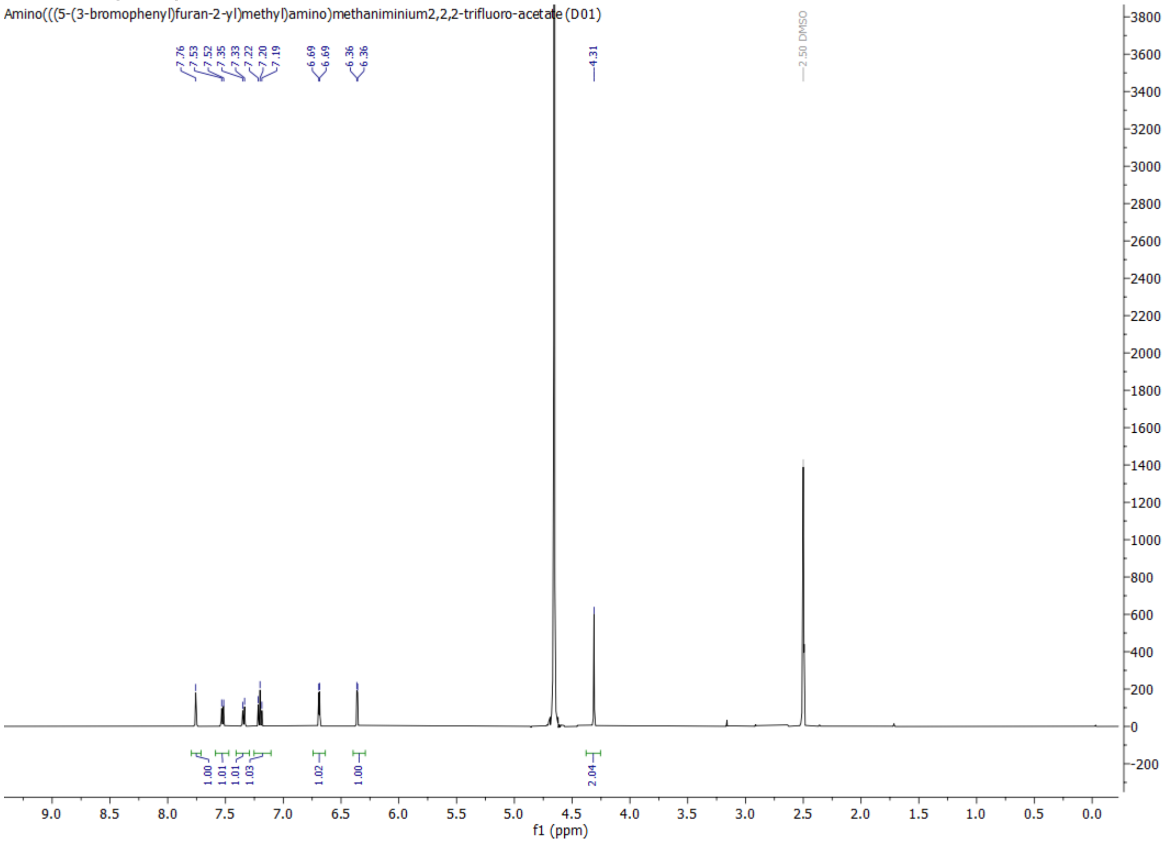

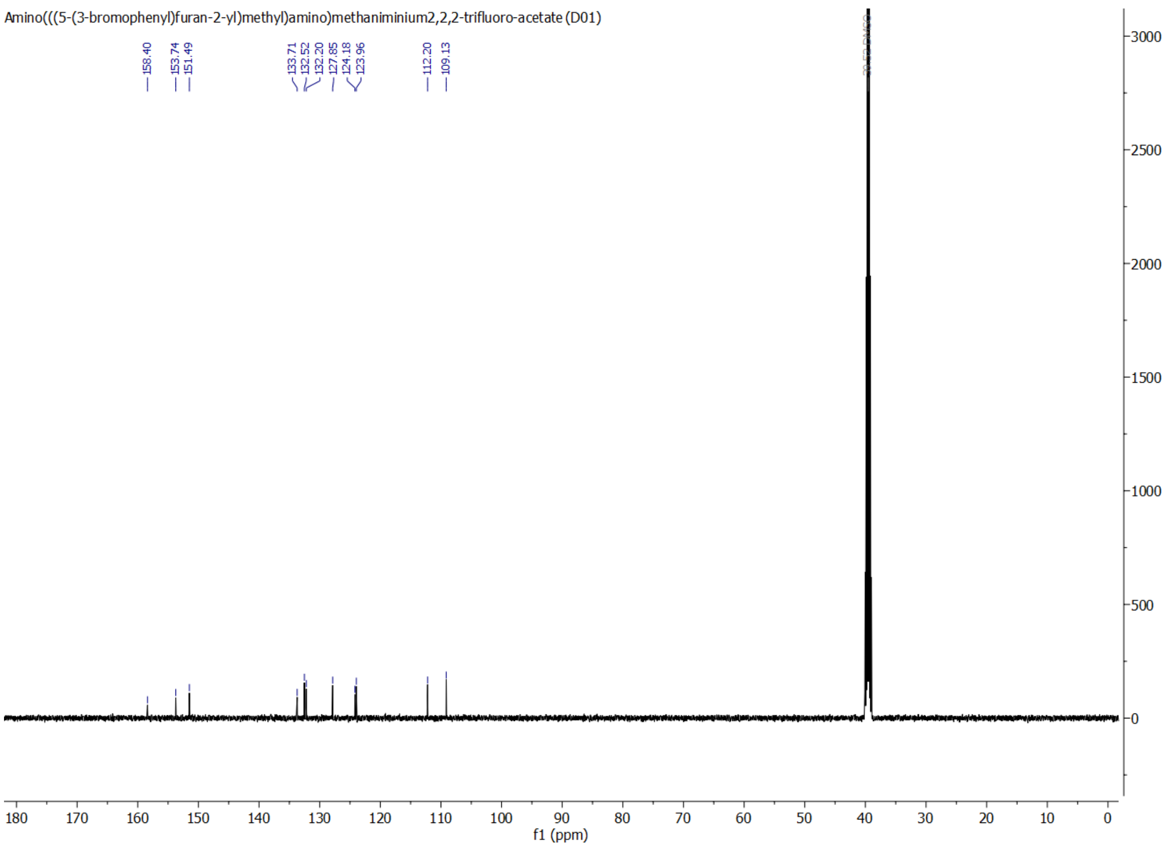
Amino(((5-(3-bromophenyl)furan-2-yl)methyl)amino)methaniminium2,2,2-trifluoro-acetate (D01)
158.40
153.74
151.49
133.71
132.52
132.20
127.85
124.18
123.96
112.20
109.13
f1 (ppm)

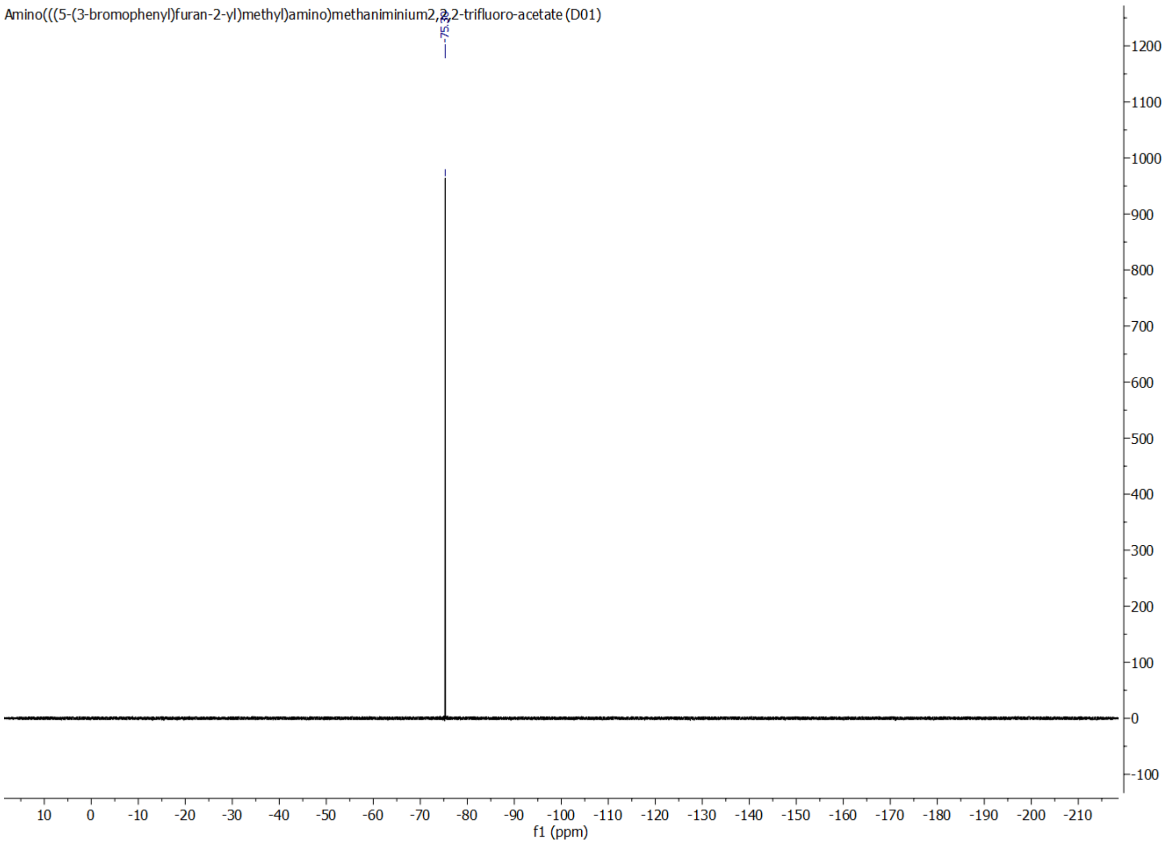
Amino(((5-(3-bromophenyl)furan-2-yl)methyl)amino)methaniminium2,2,2-trifluoro-acetate (D01)
f1 (ppm)

(5-(2-Bromophenyl)furan-2-yl)methanamine (3)

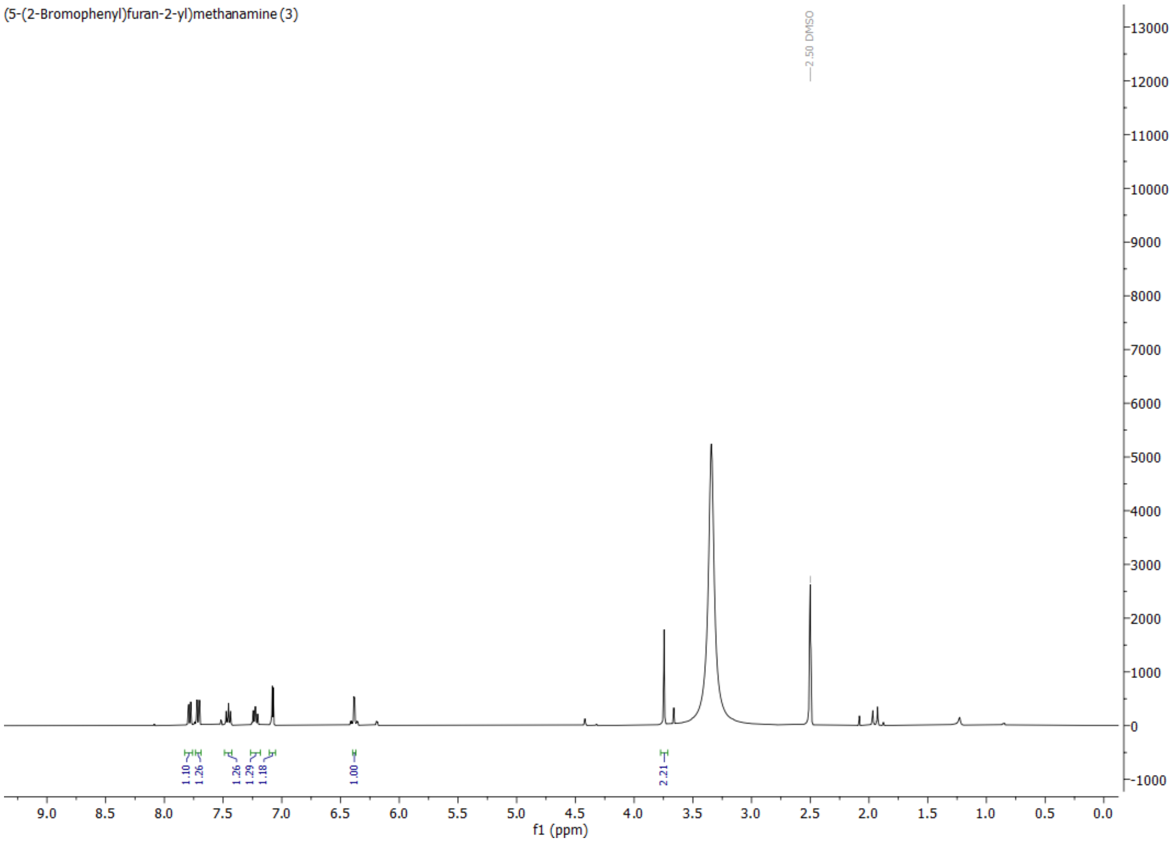

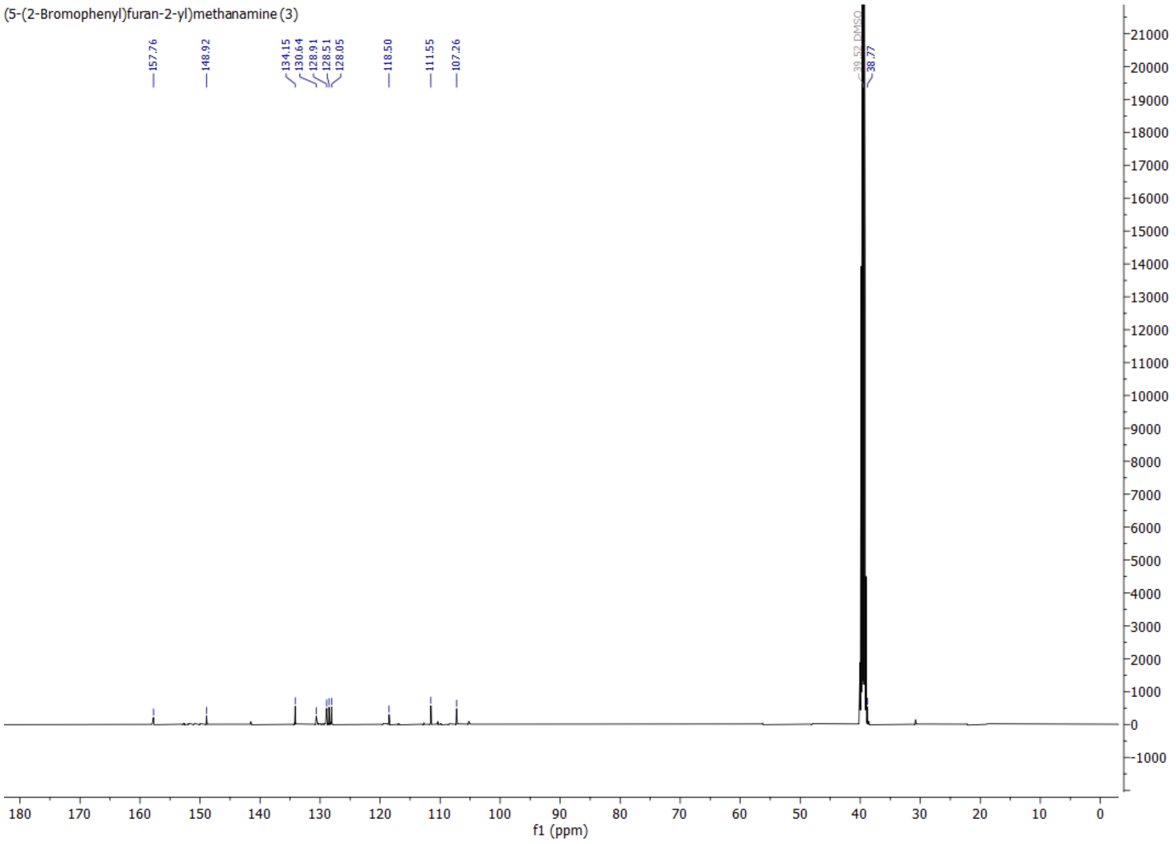

Amino(((5-(2-bromophenyl)furan-2-yl)methyl)amino)methaniminium 2,2,2-trifluoro-acetate (D02)

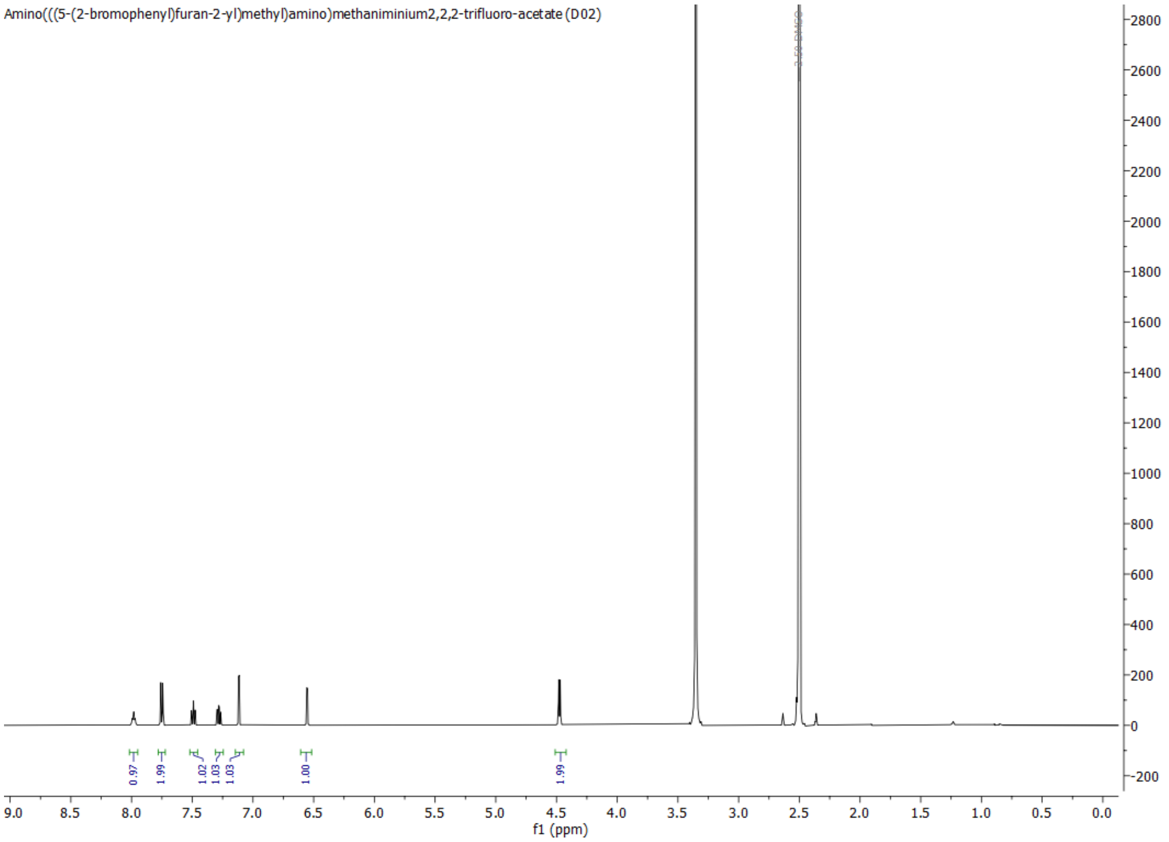

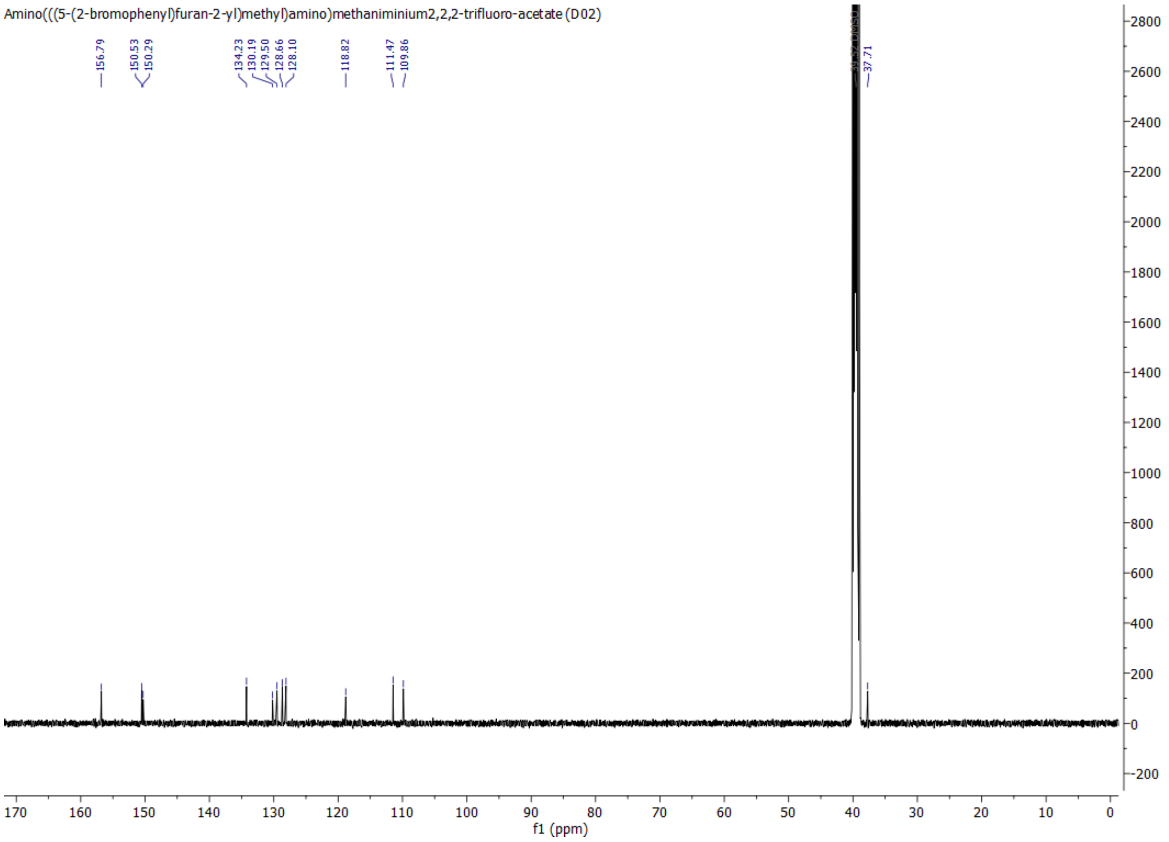

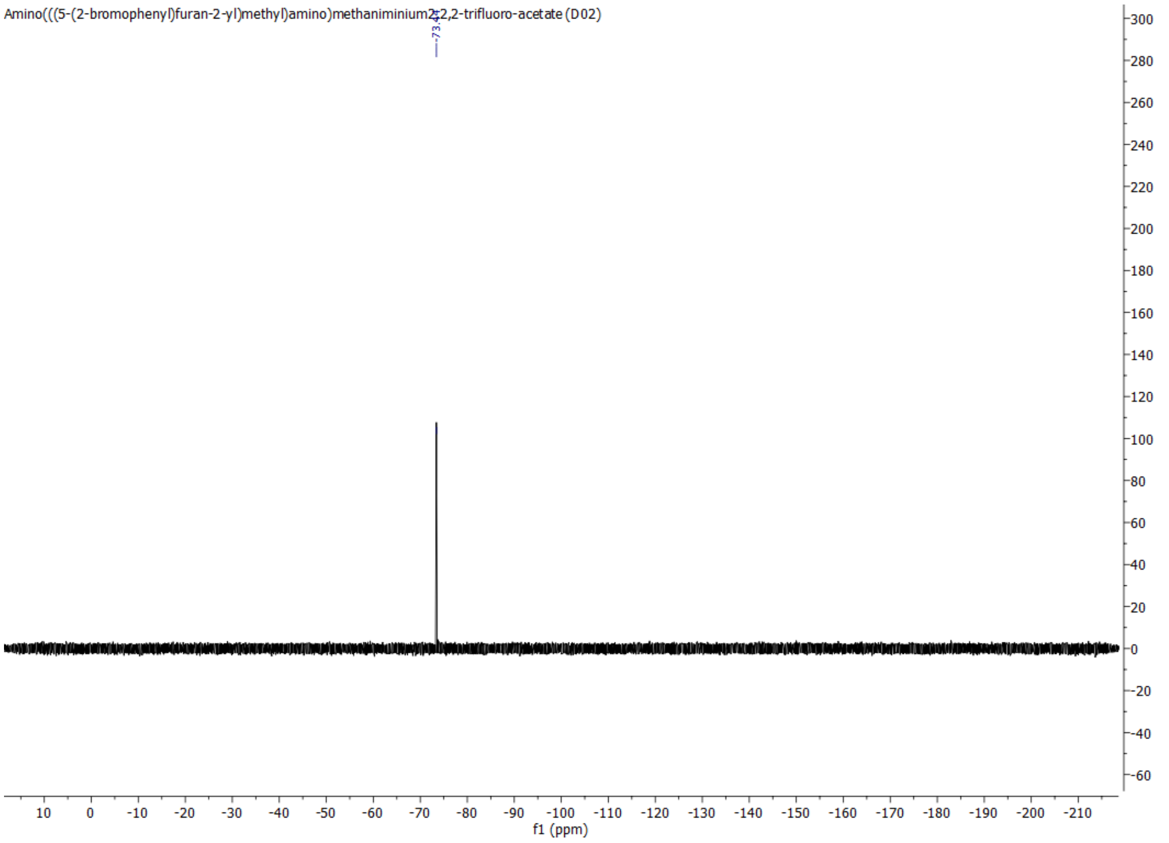

(5-(4-Fluorophenyl)furan-2-yl)methanamine (4)

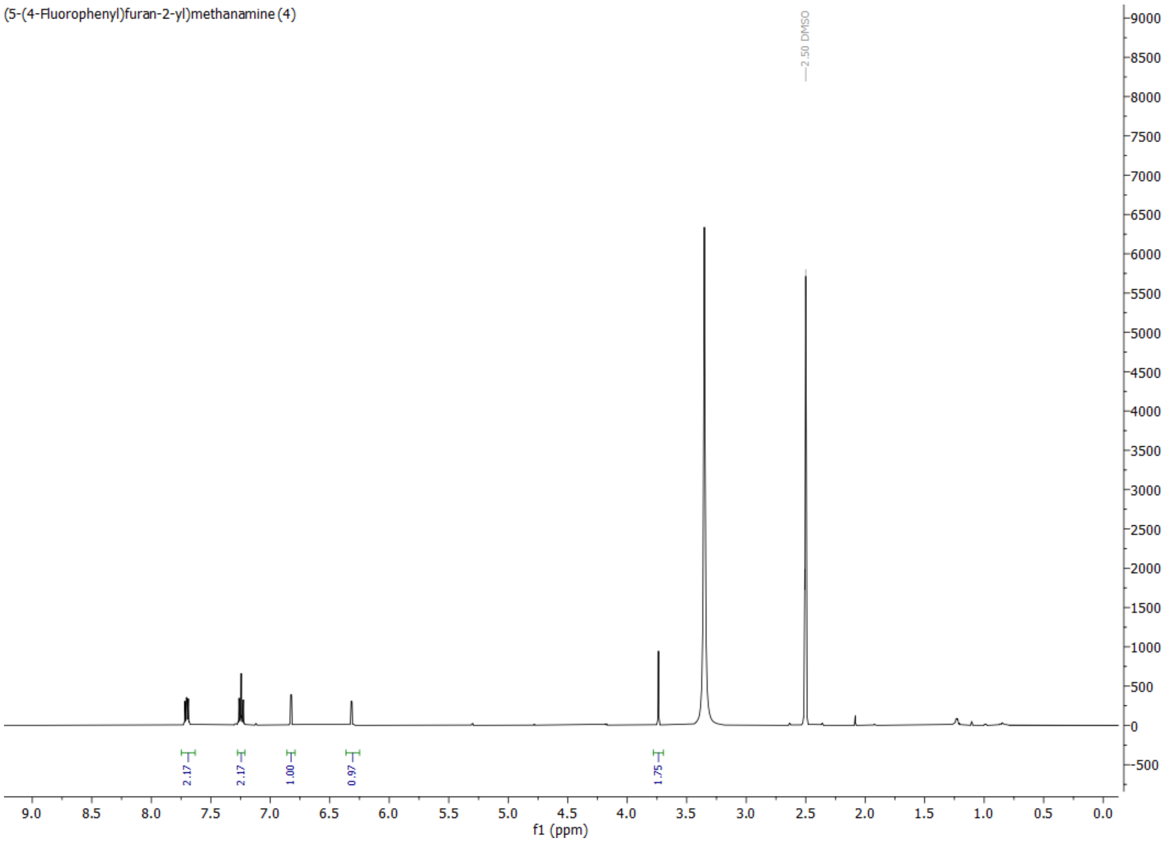

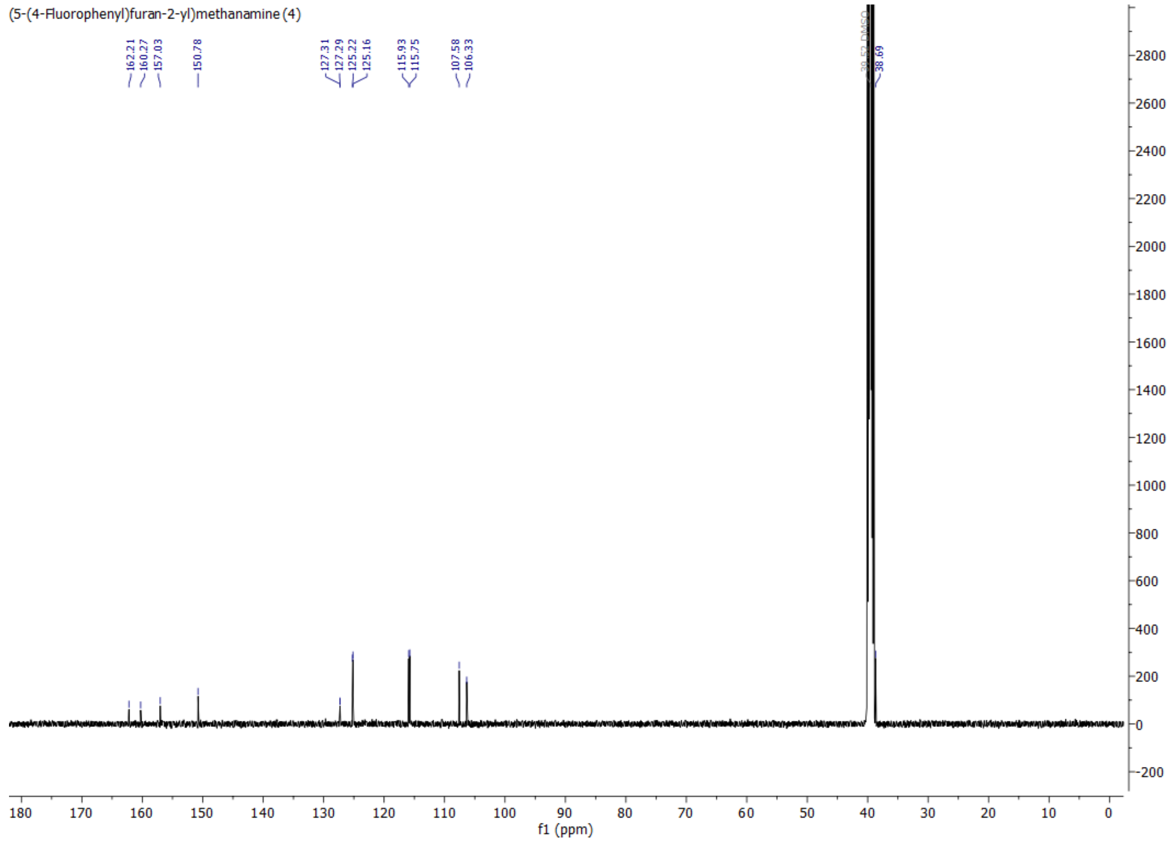
(5-(4-Fluorophenyl)furan-2-yl)methanamine (4)
162.21
160.27
157.03
150.78
127.31
127.29
125.22
125.16
115.93
115.75
107.58
106.33
39.52 DMSO
38.69
f1 (ppm)

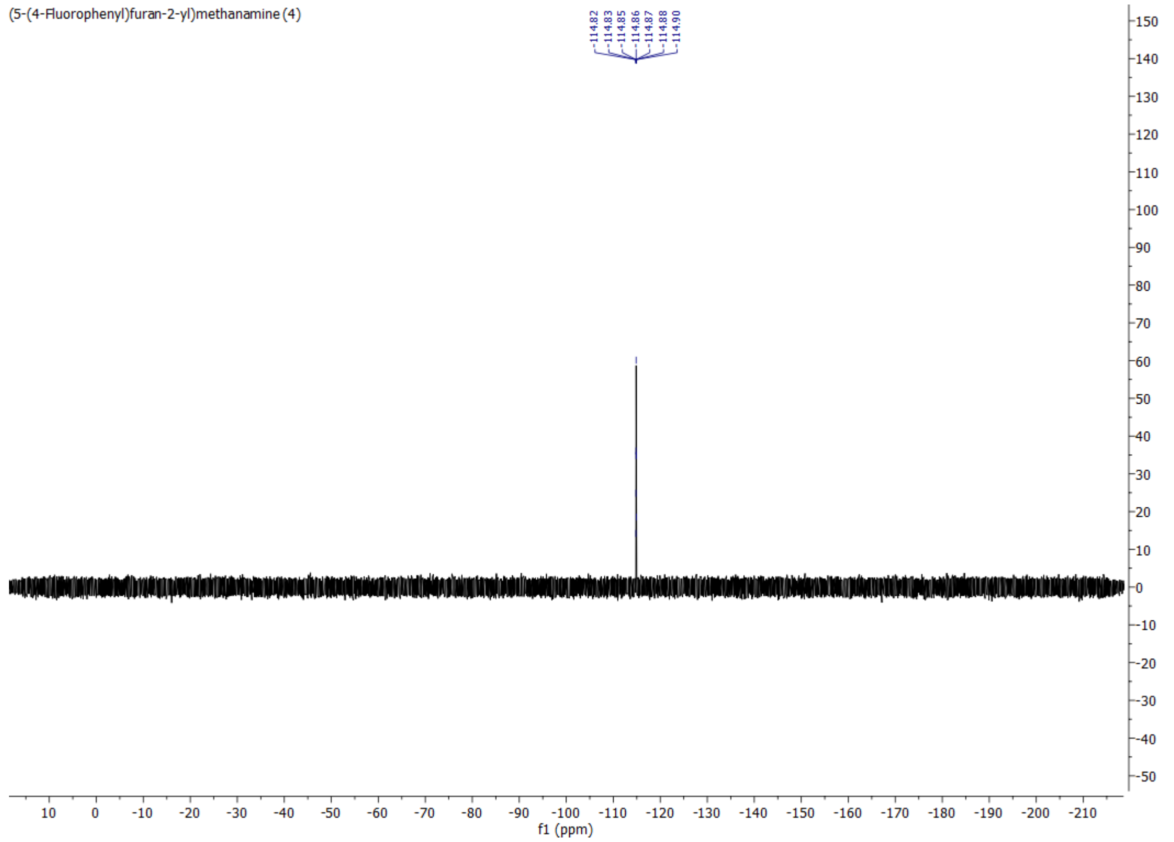
(5-(4-Fluorophenyl)furan-2-yl)methanamine (4)
-114.82
-114.83
-114.85
-114.86
-114.87
-114.88
-114.90
f1 (ppm)

Amino(((5-(4-fluorophenyl)furan-2-yl)methyl)amino)methaniminium 2,2,2-trifluoroacetate (D03)

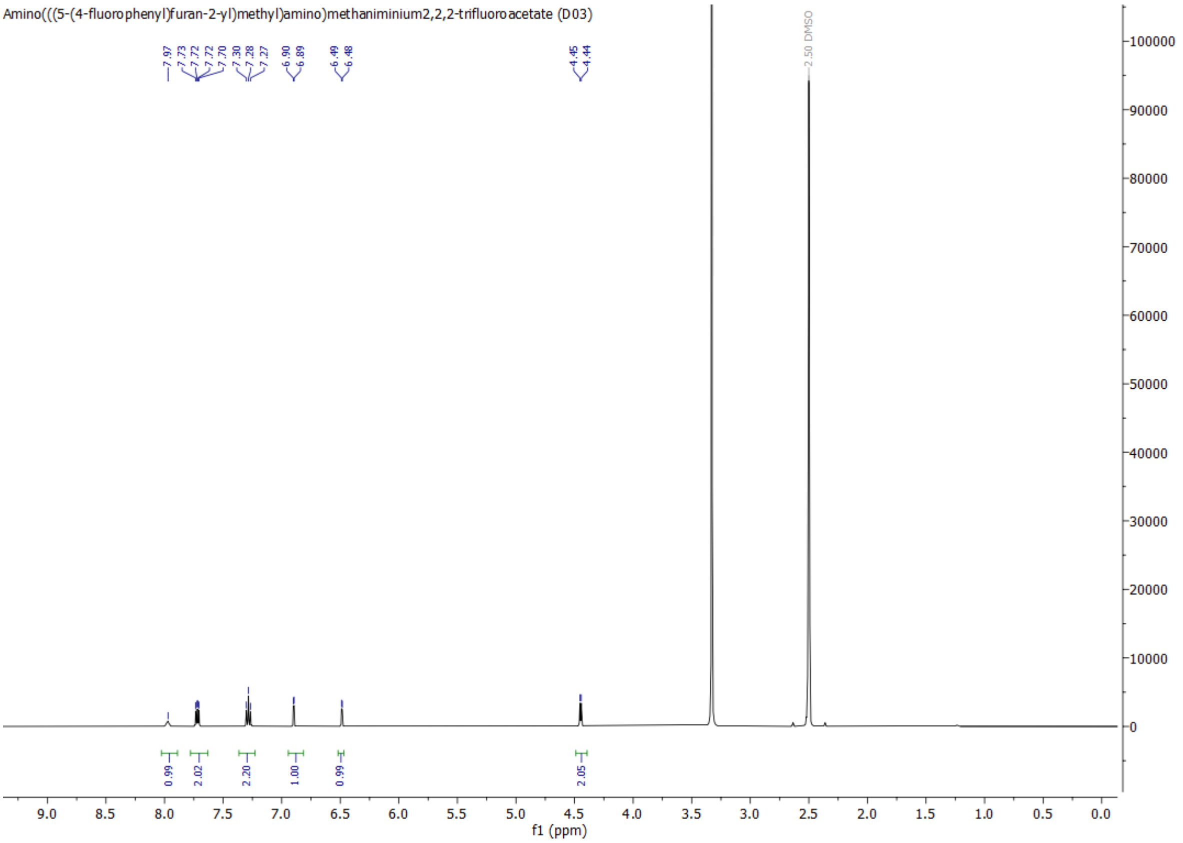

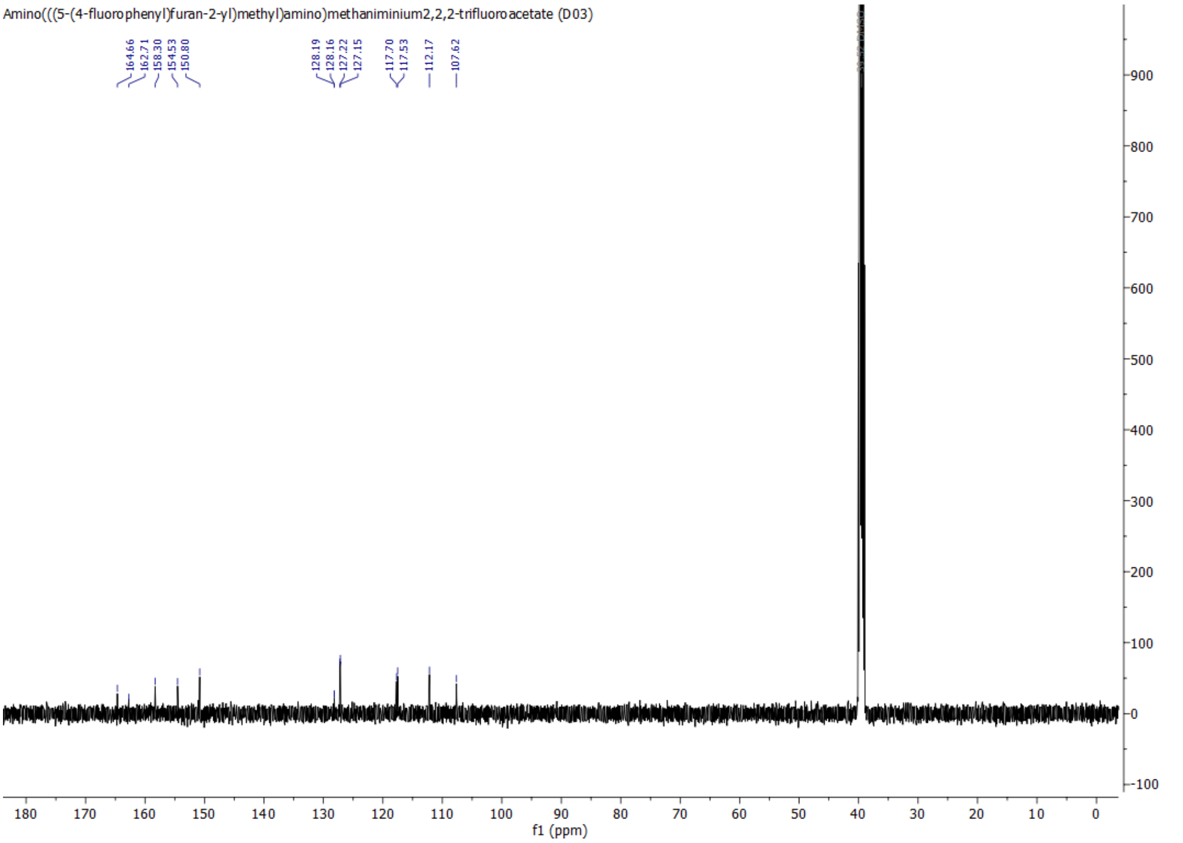

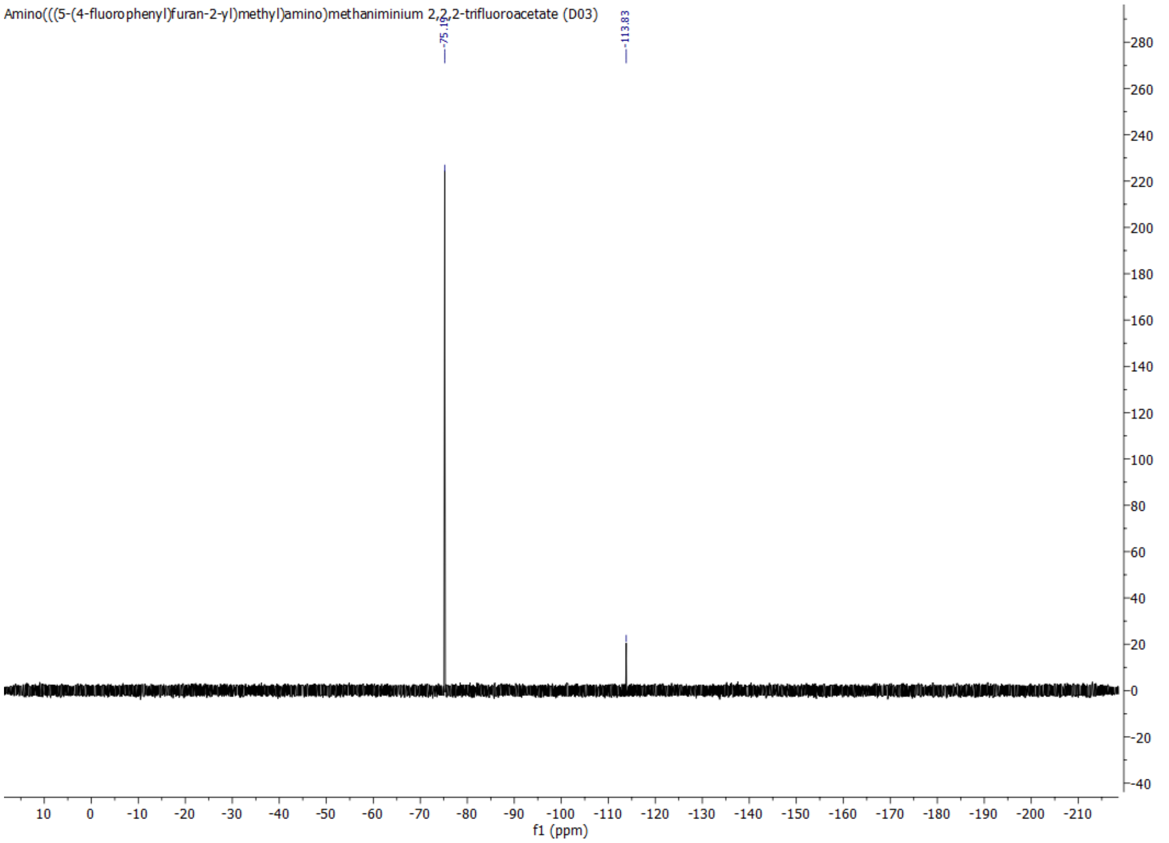

Amino(((5-phenylfuran-2-yl)methyl)amino)methaniminium 2,2,2-trifluoroacetate (D04)

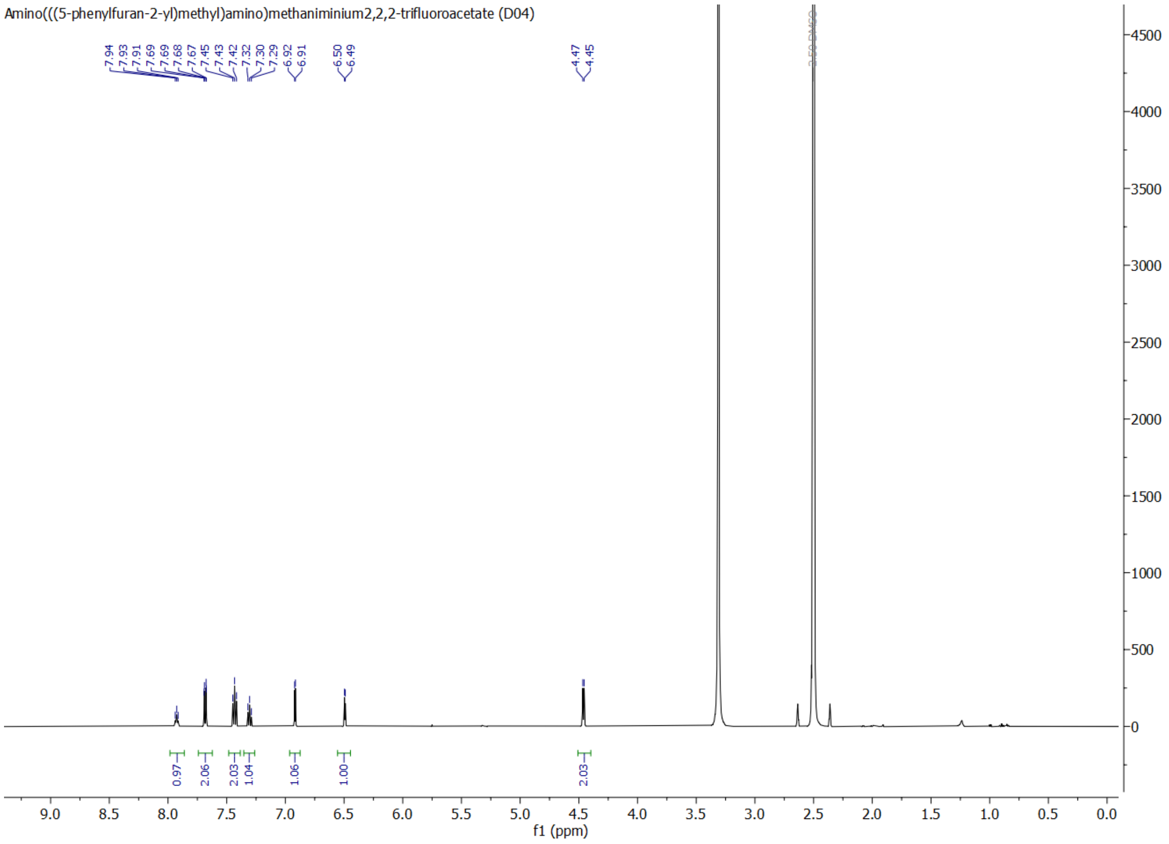

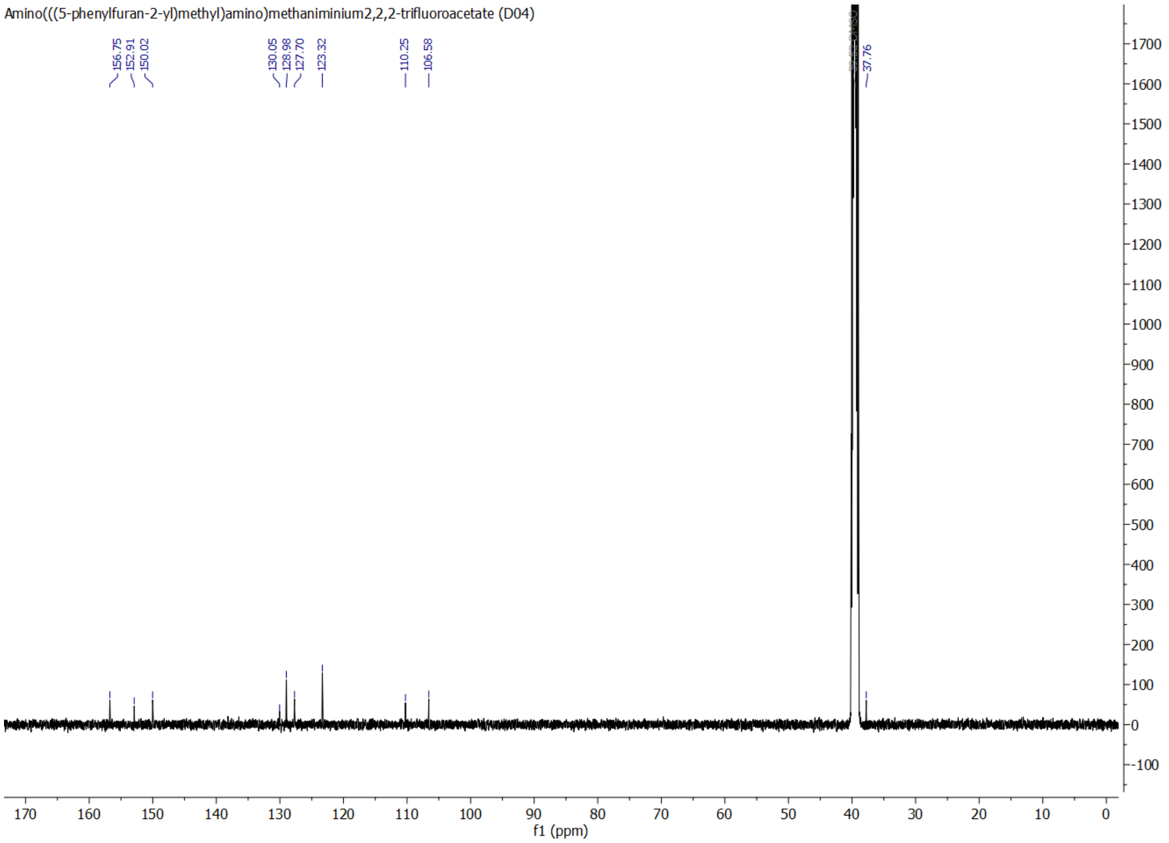
Amino(((5-phenylfuran-2-yl)methyl)amino)methaniminium2,2,2-trifluoroacetate (D04)
156.75
152.91
150.02
130.05
128.98
127.70
123.32
110.25
106.58
37.76
f1 (ppm)

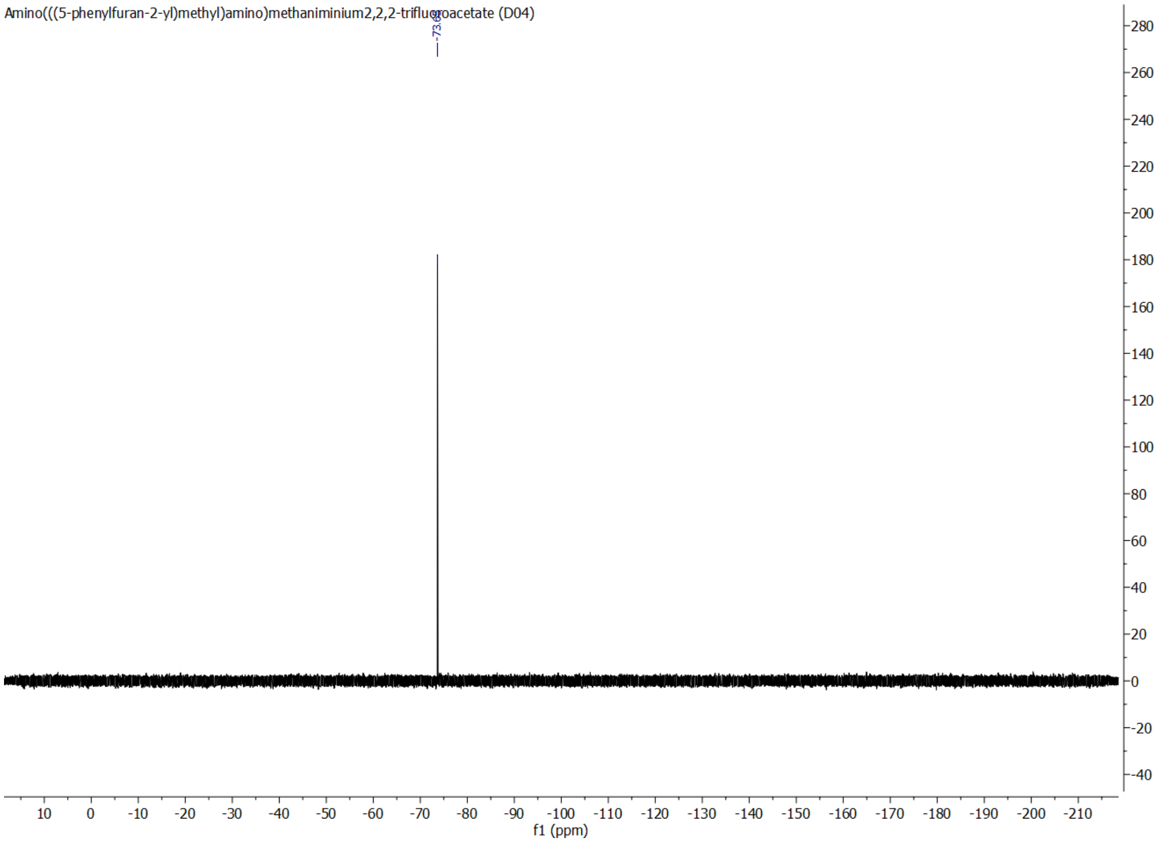
Amino(((5-phenylfuran-2-yl)methyl)amino)methaniminium2,2,2-trifluoroacetate (D04)
f1 (ppm)

(5-(4-Isopropylphenyl)furan-2-yl)methanamine (6)

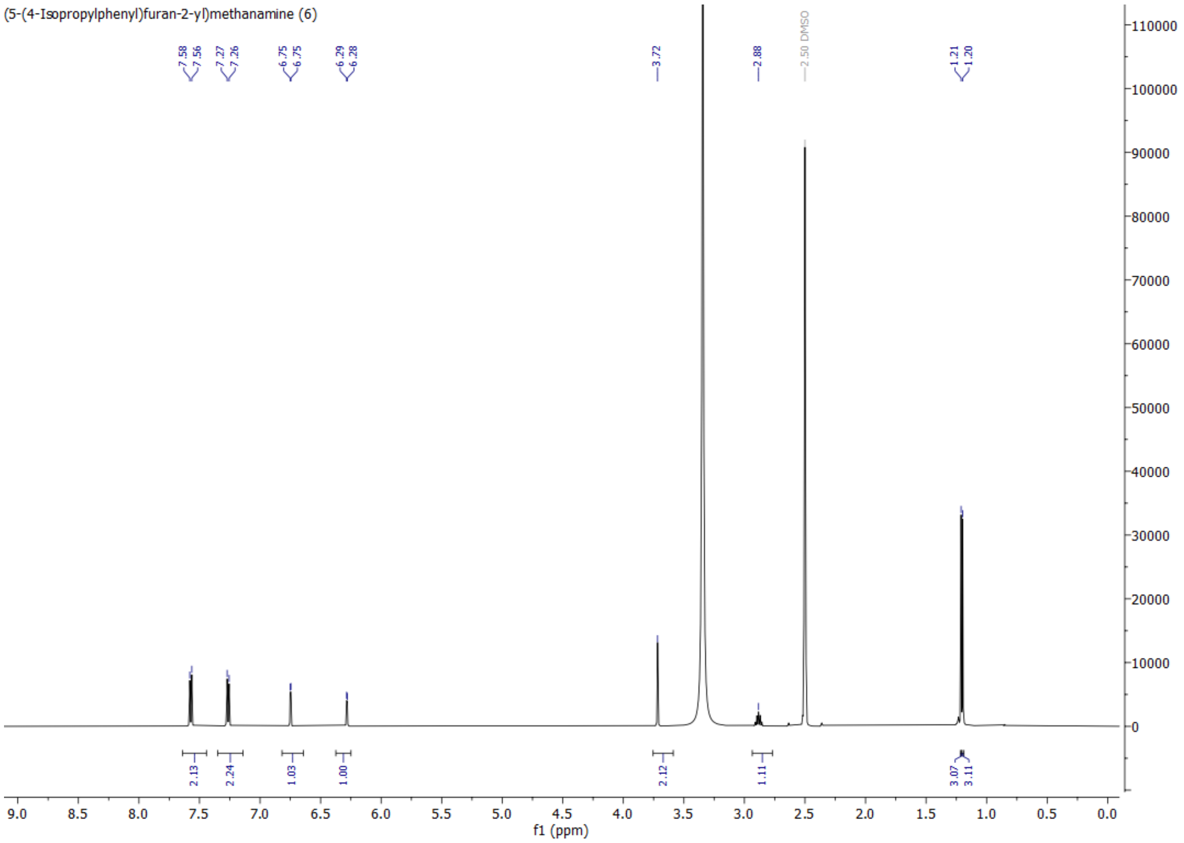

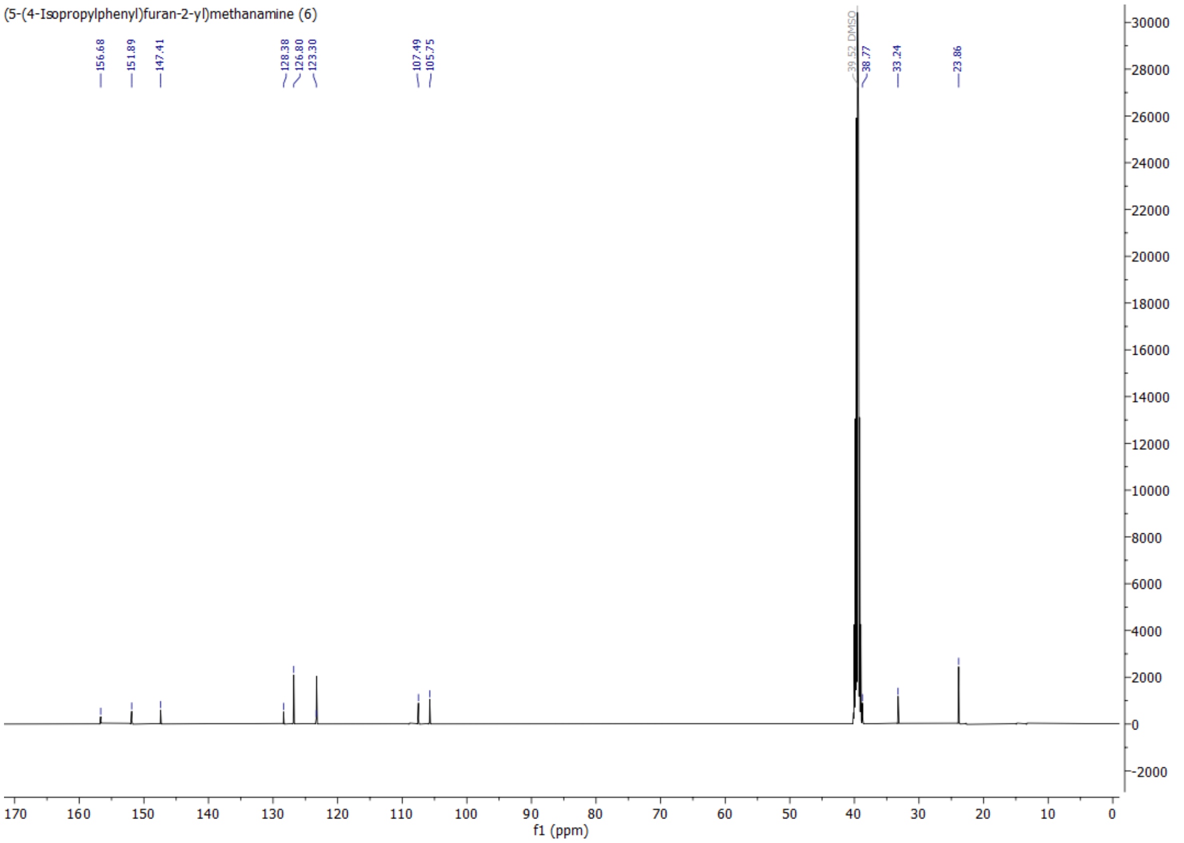

Amino(((5-(4-isopropylphenyl)furan-2-yl)methyl)amino)methaniminium 2,2,2-tri-fluoroacetate (D05)

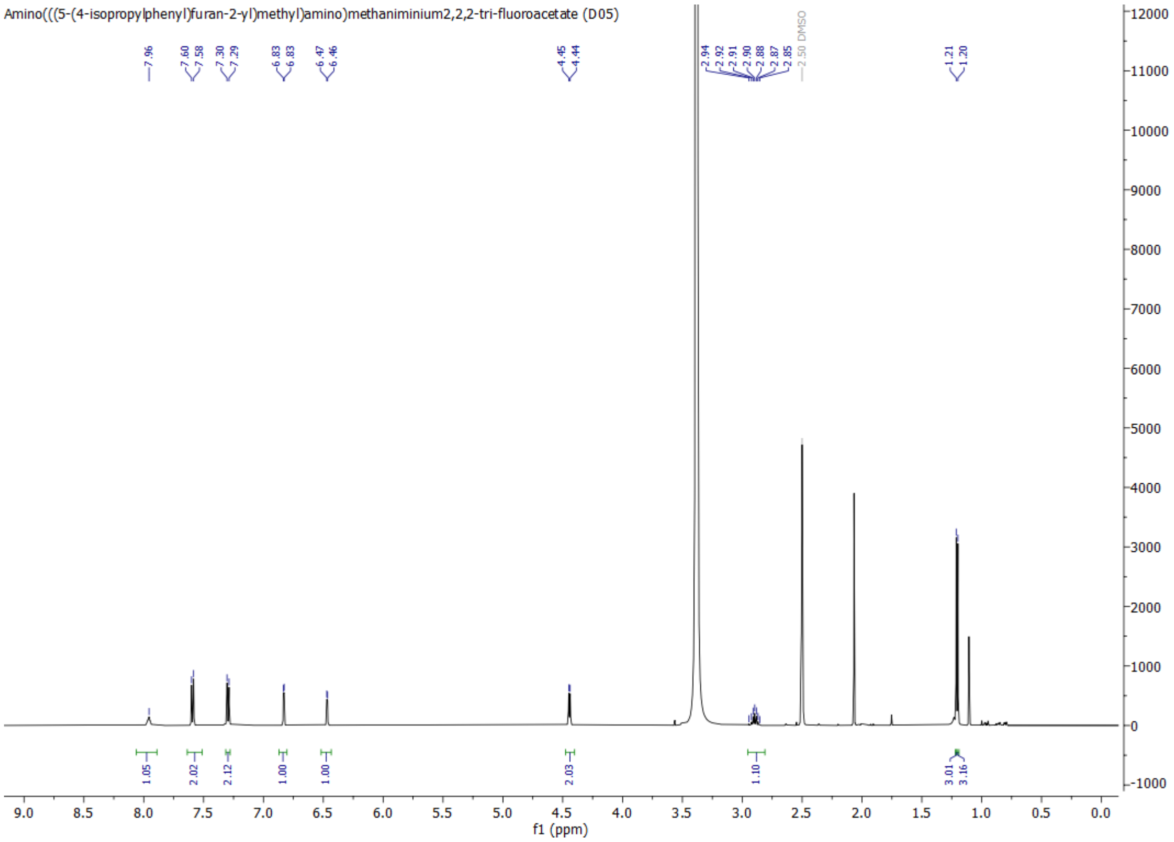

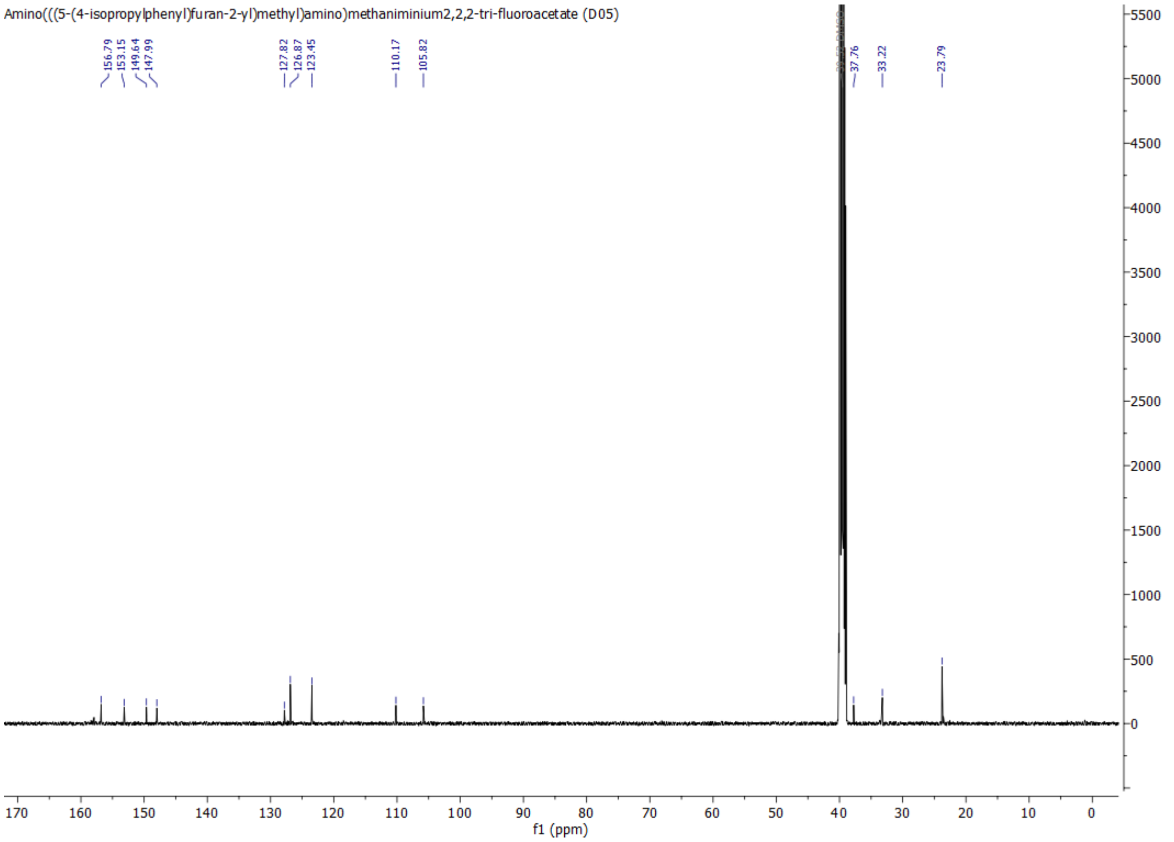

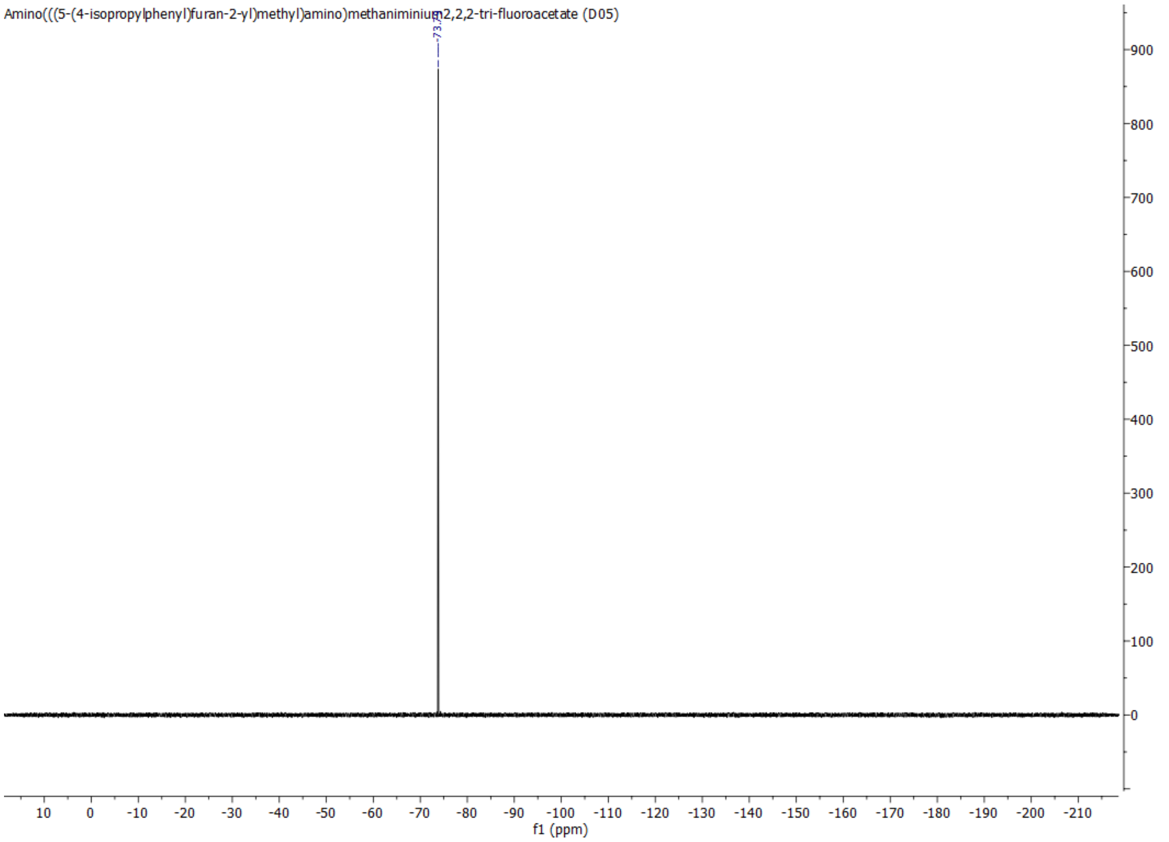

(5-(4-(Trifluoromethyl)phenyl)furan-2-yl)methanamine (7)

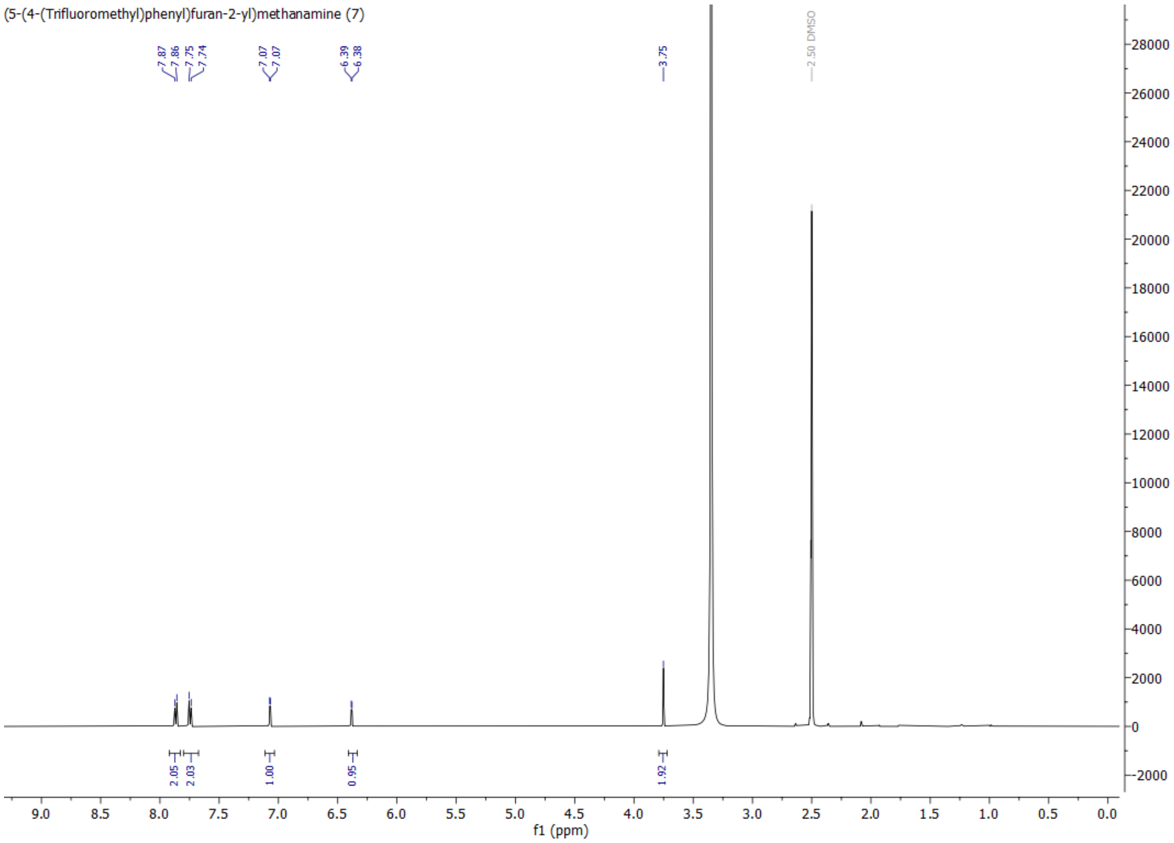

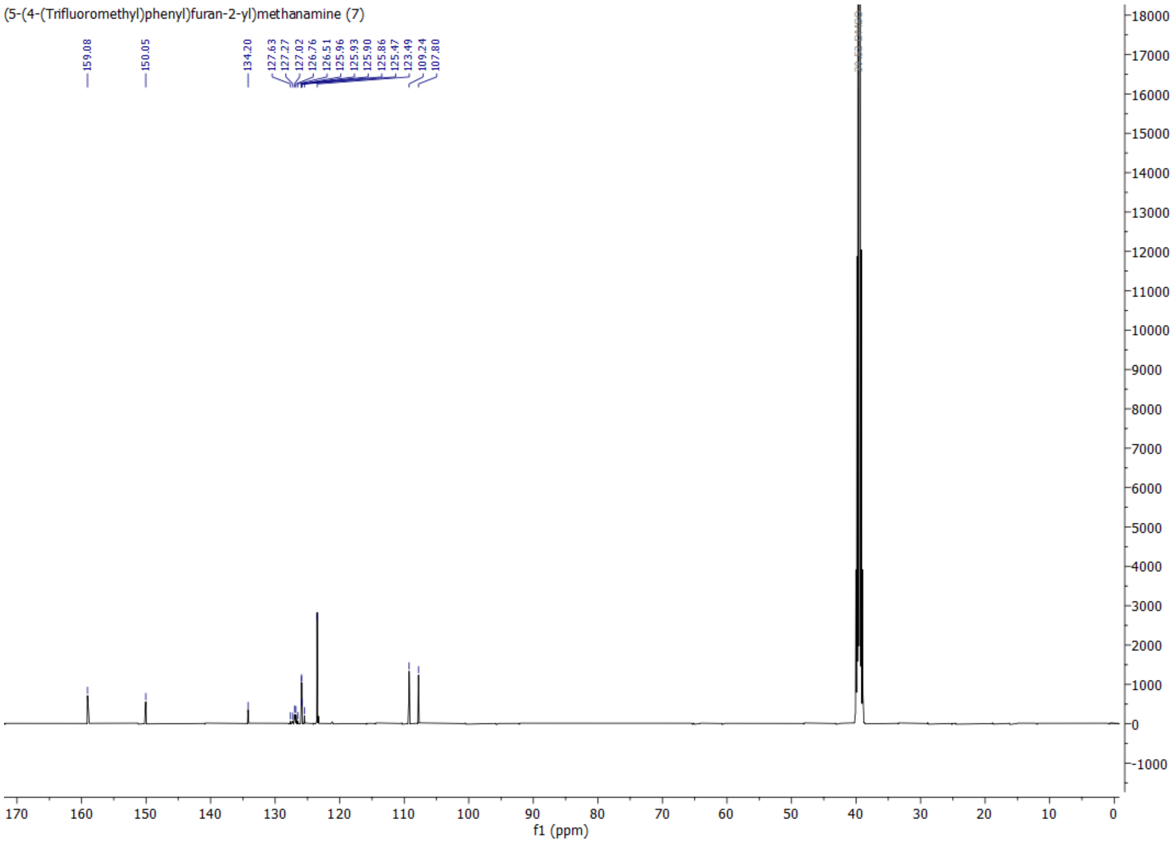
(5-(4-(Trifluoromethyl)phenyl)furan-2-yl)methanamine (7)
159.08
150.05
134.20
127.63
127.27
127.02
126.76
126.51
125.96
125.93
125.90
125.86
125.47
123.49
109.24
107.80
f1 (ppm)

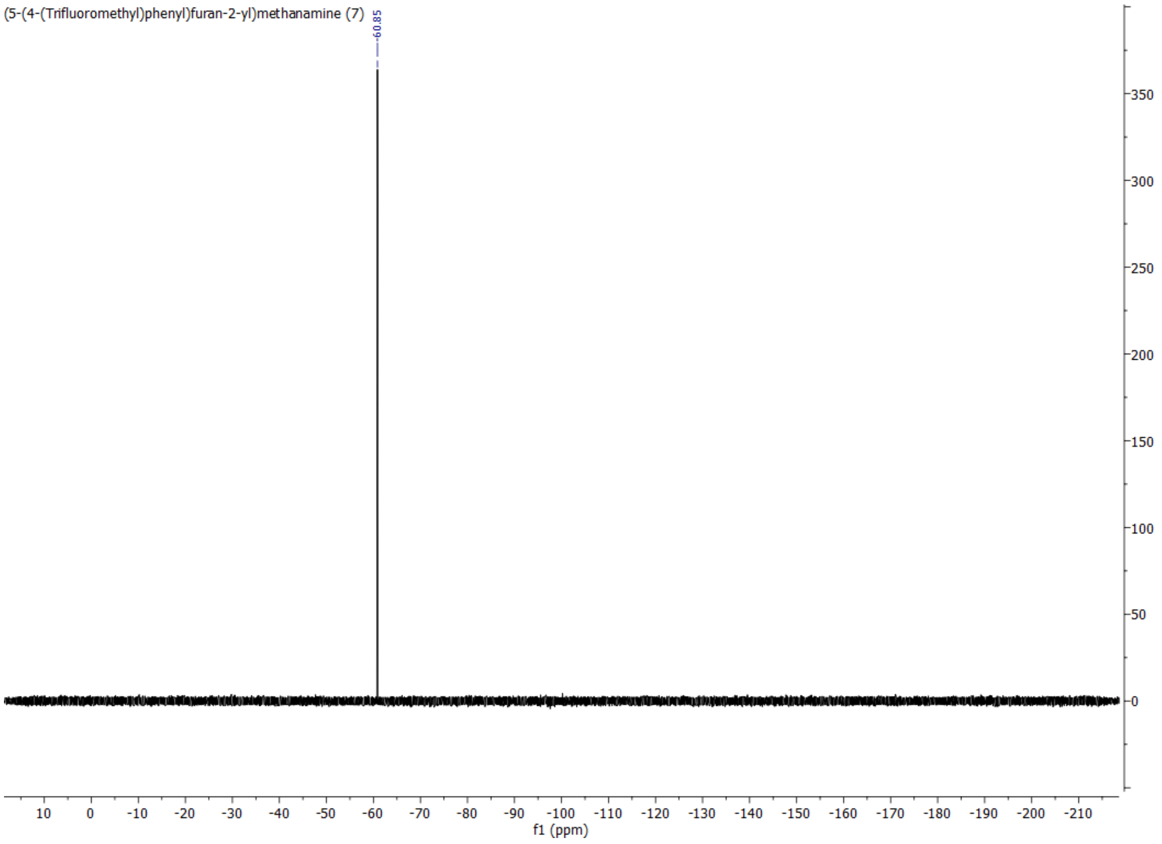
(5-(4-(Trifluoromethyl)phenyl)furan-2-yl)methanamine (7)
-60.85
f1 (ppm)

Amino(((5-(4-(trifluoromethyl)phenyl)furan-2-yl)methyl)amino)methaniminium 2,2,2-trifluoroacetate (D06)

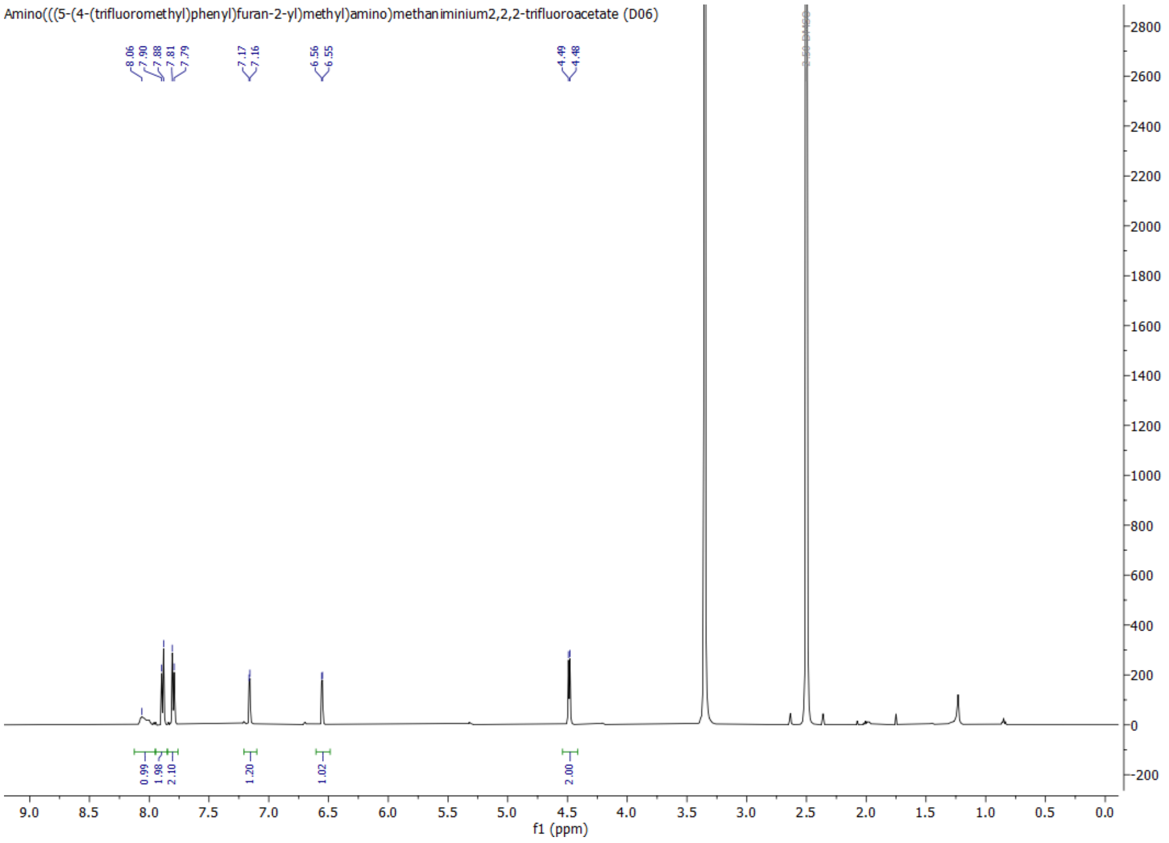

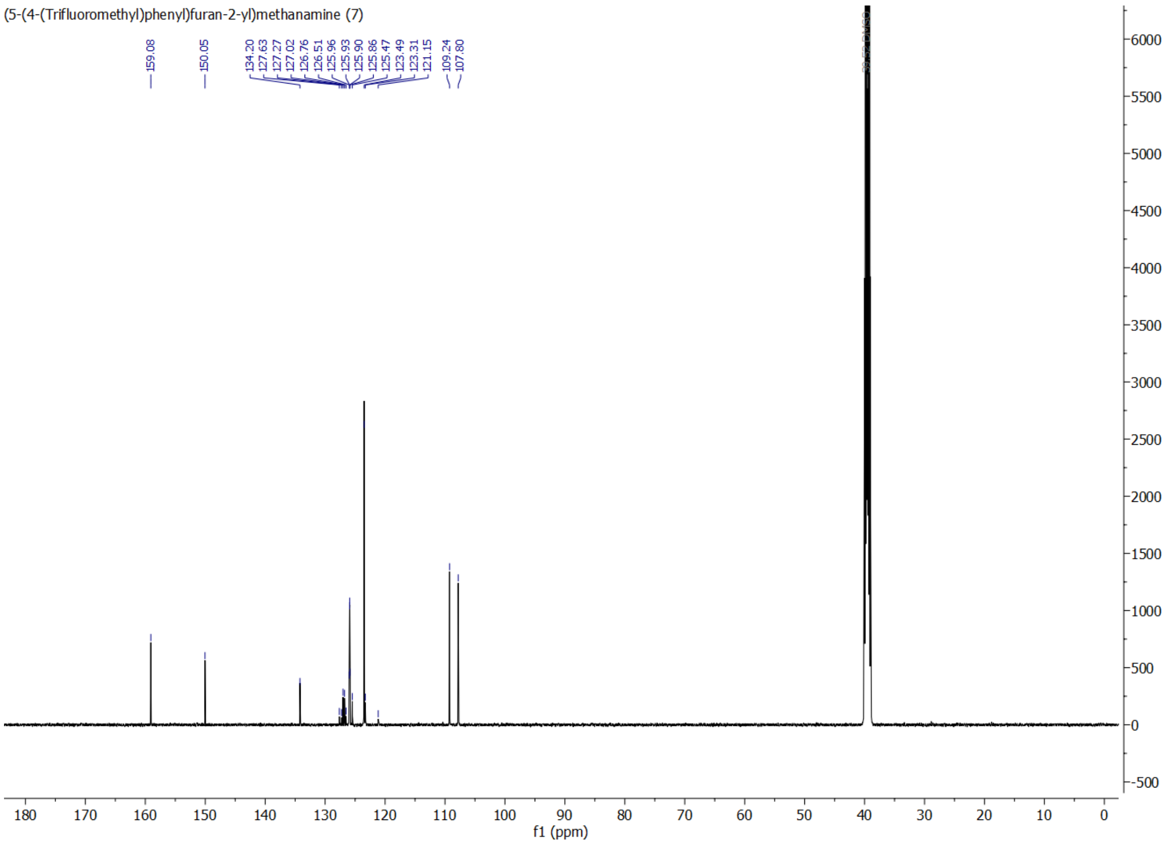

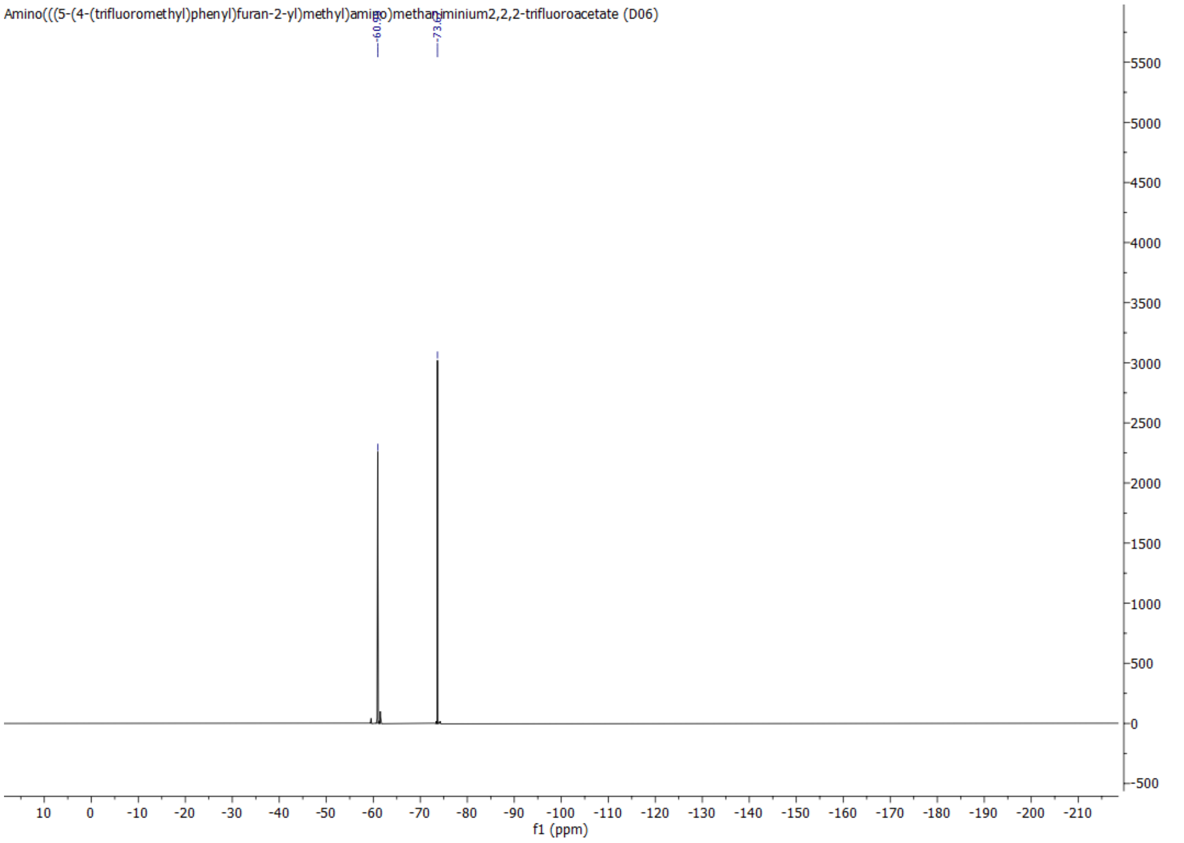

(5-(4-(*tert*-Butyl)phenyl)furan-2-yl)methanamine (8)

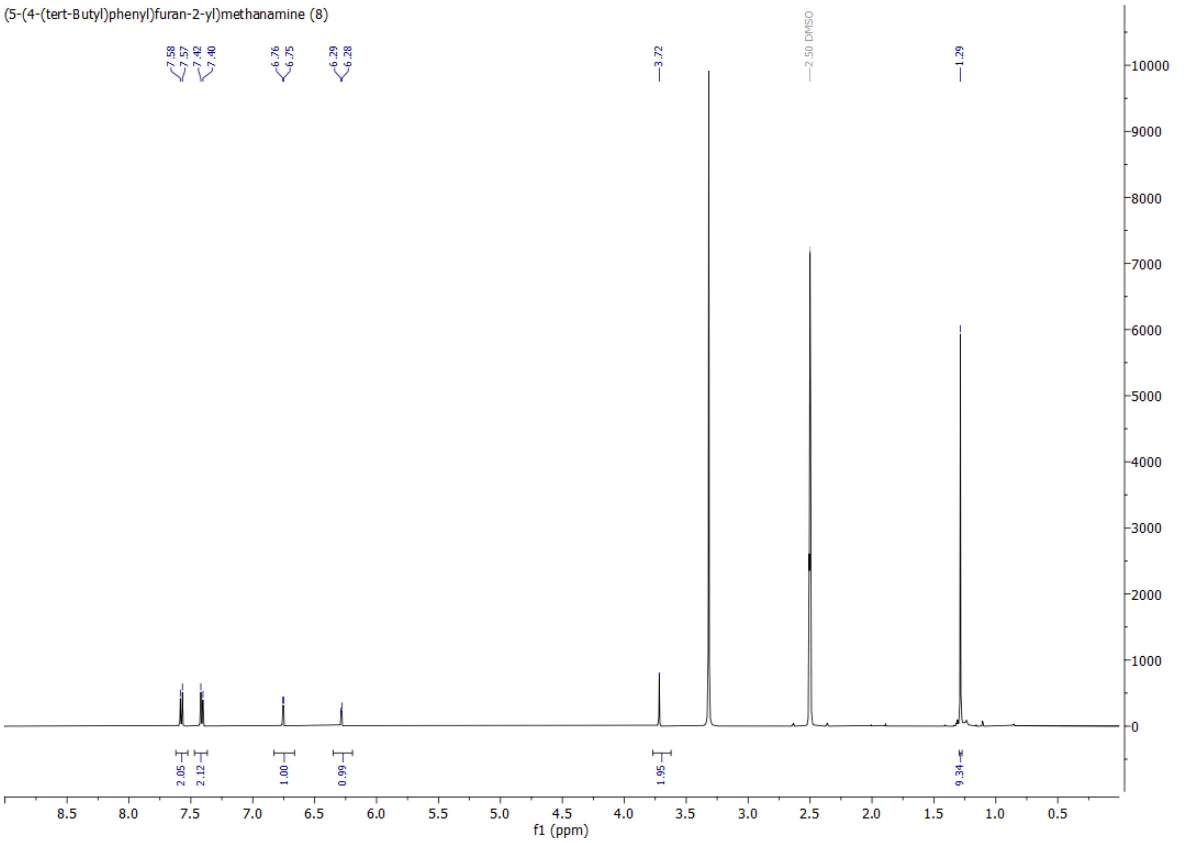

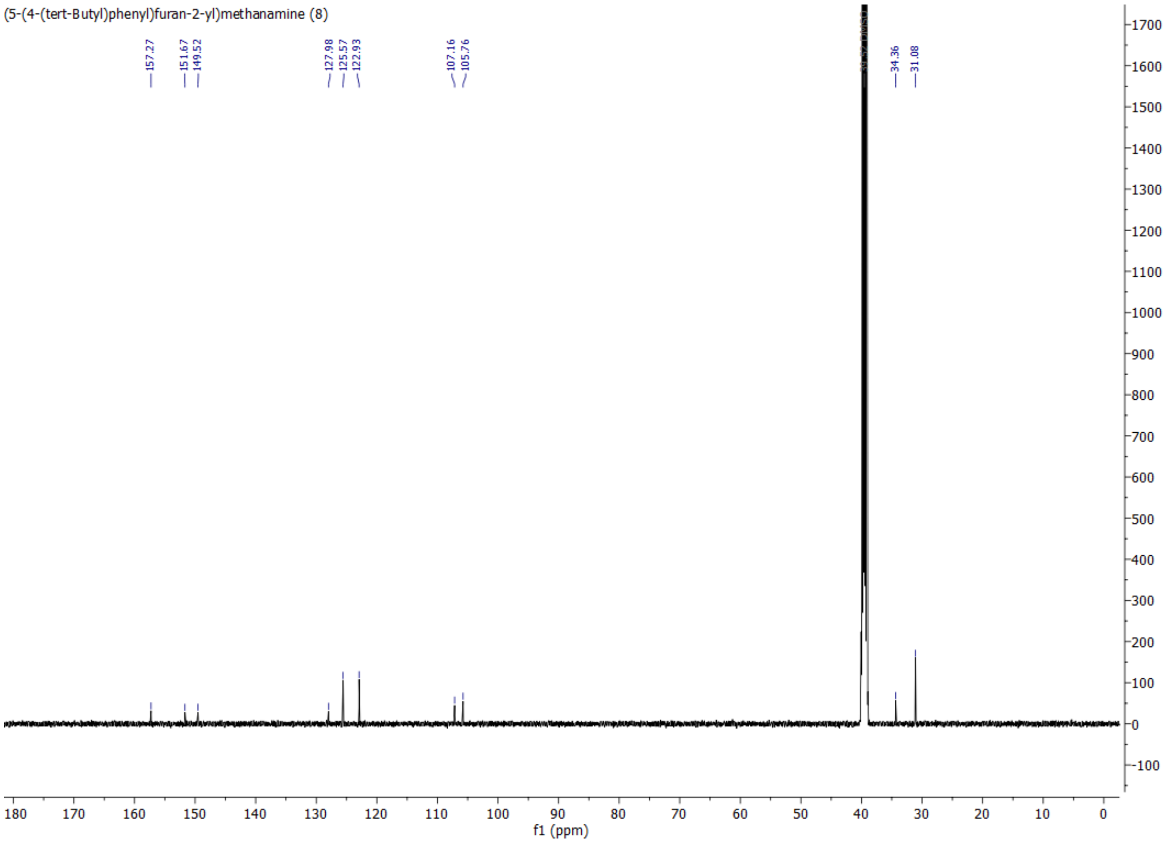

Amino(((5-(4-(*tert*-butyl)phenyl)furan-2-yl)methyl)amino)methaniminium 2,2,2-trifluoroacetate (D07)

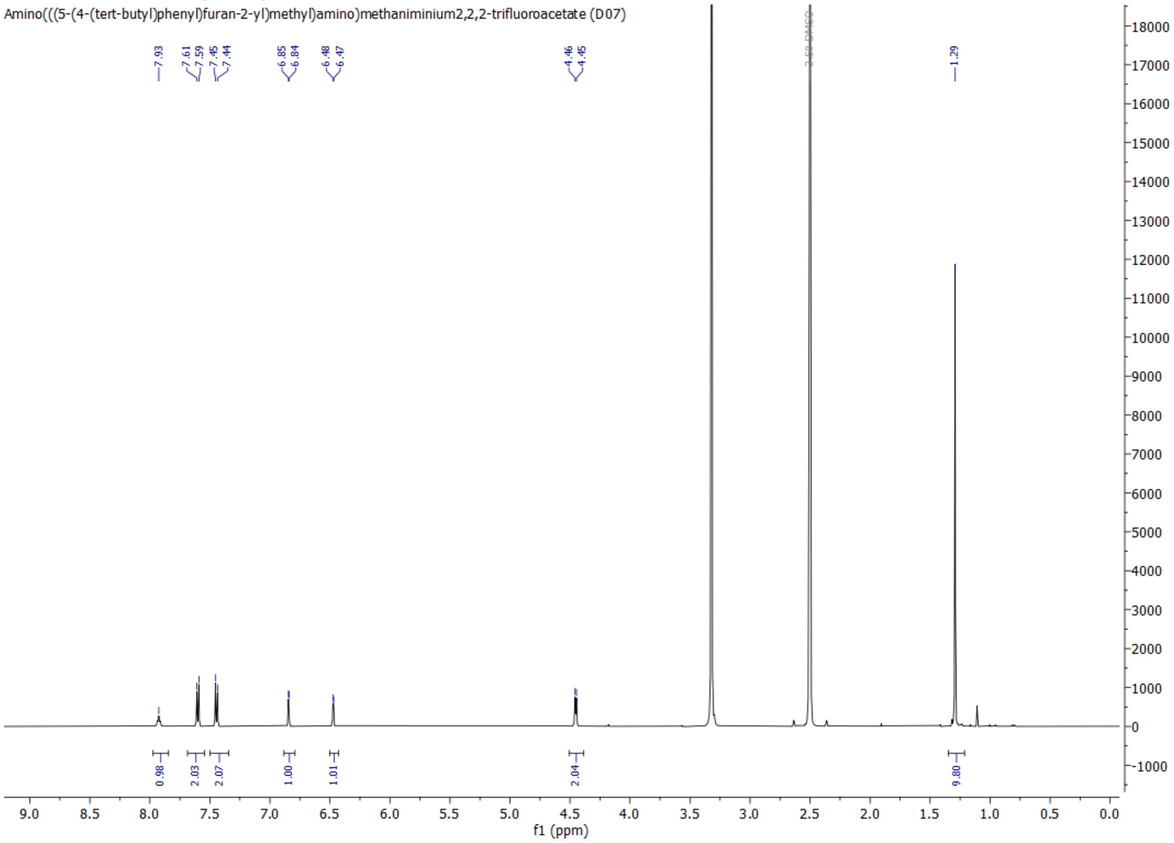

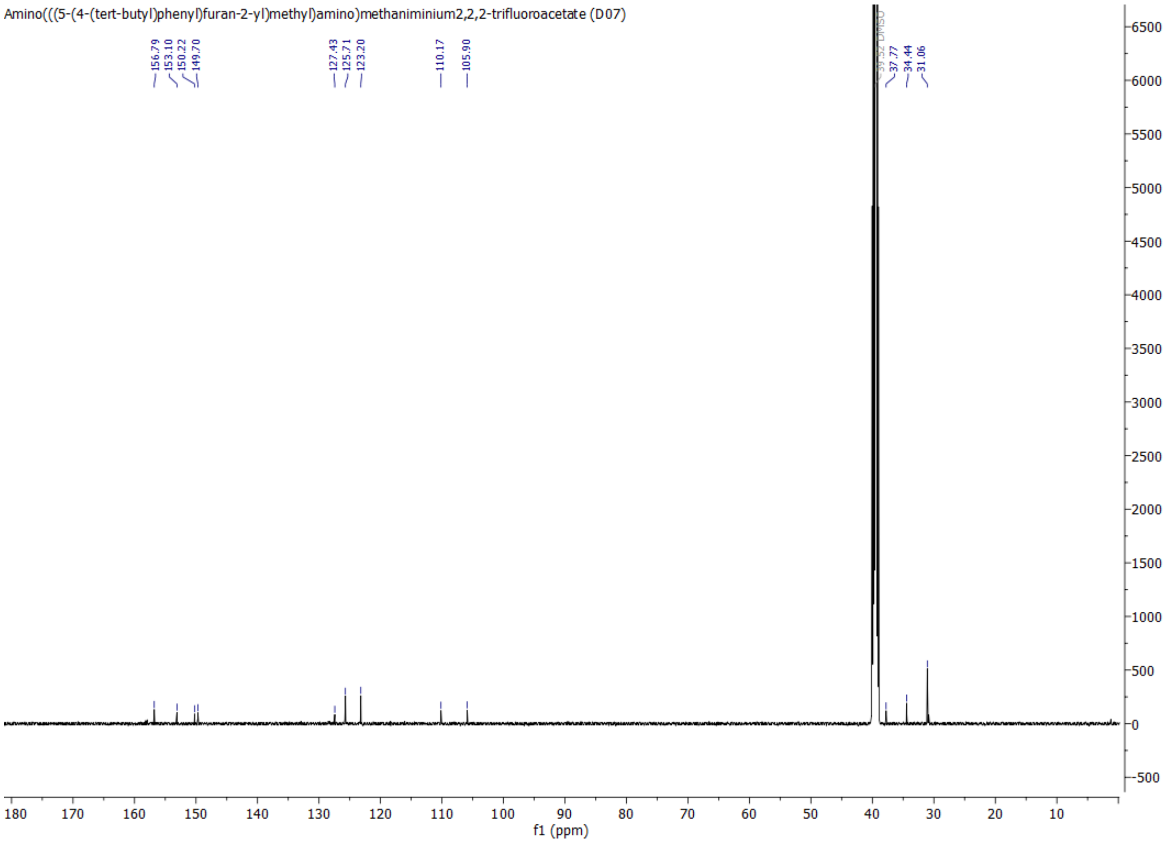
Amino(((5-(4-(tert-butyl)phenyl)furan-2-yl)methyl)amino)methaniminium2,2,2-trifluoroacetate (D07)
156.79
153.10
150.22
149.70
127.43
125.71
123.20
110.17
105.90
39.52 DMSO
37.77
34.44
31.06
f1 (ppm)

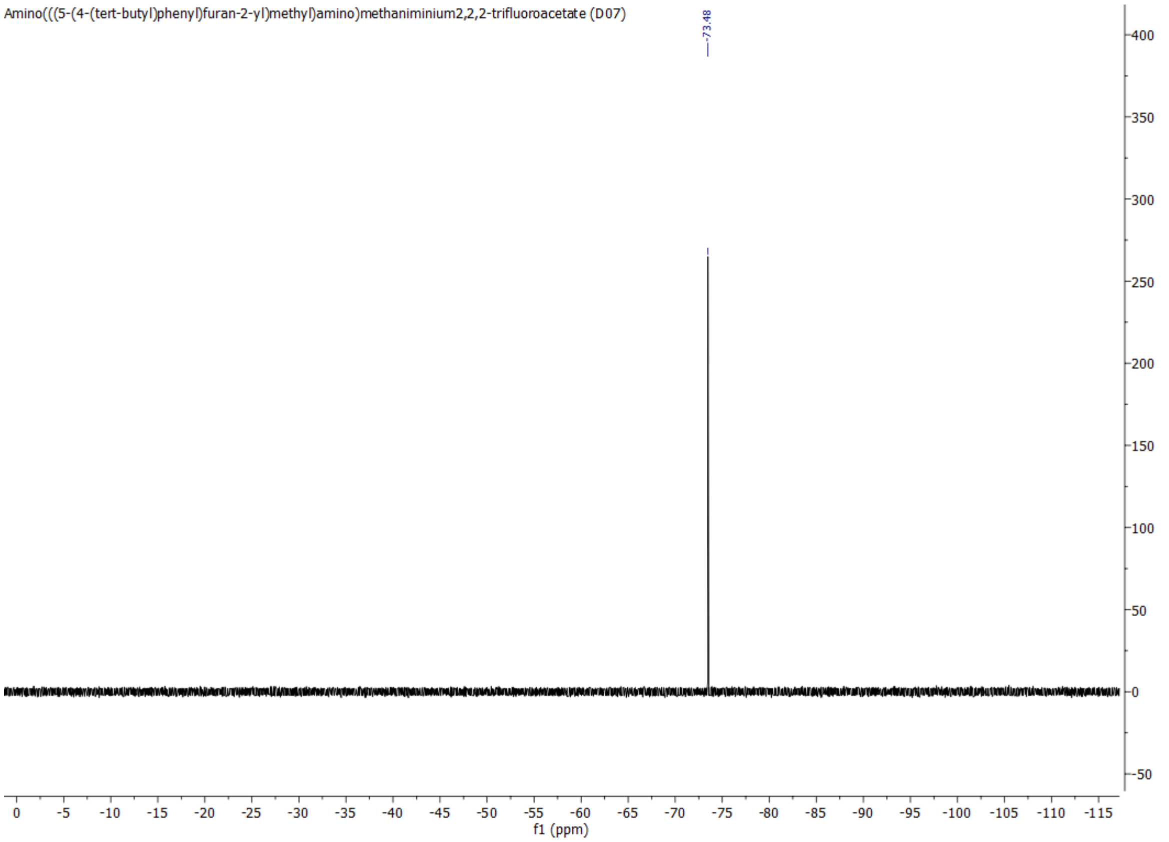
Amino(((5-(4-(tert-butyl)phenyl)furan-2-yl)methyl)amino)methaniminium2,2,2-trifluoroacetate (D07)
-73.48
f1 (ppm)

(5-(4-(Methylsulfonyl)phenyl)furan-2-yl)methanamine (9)

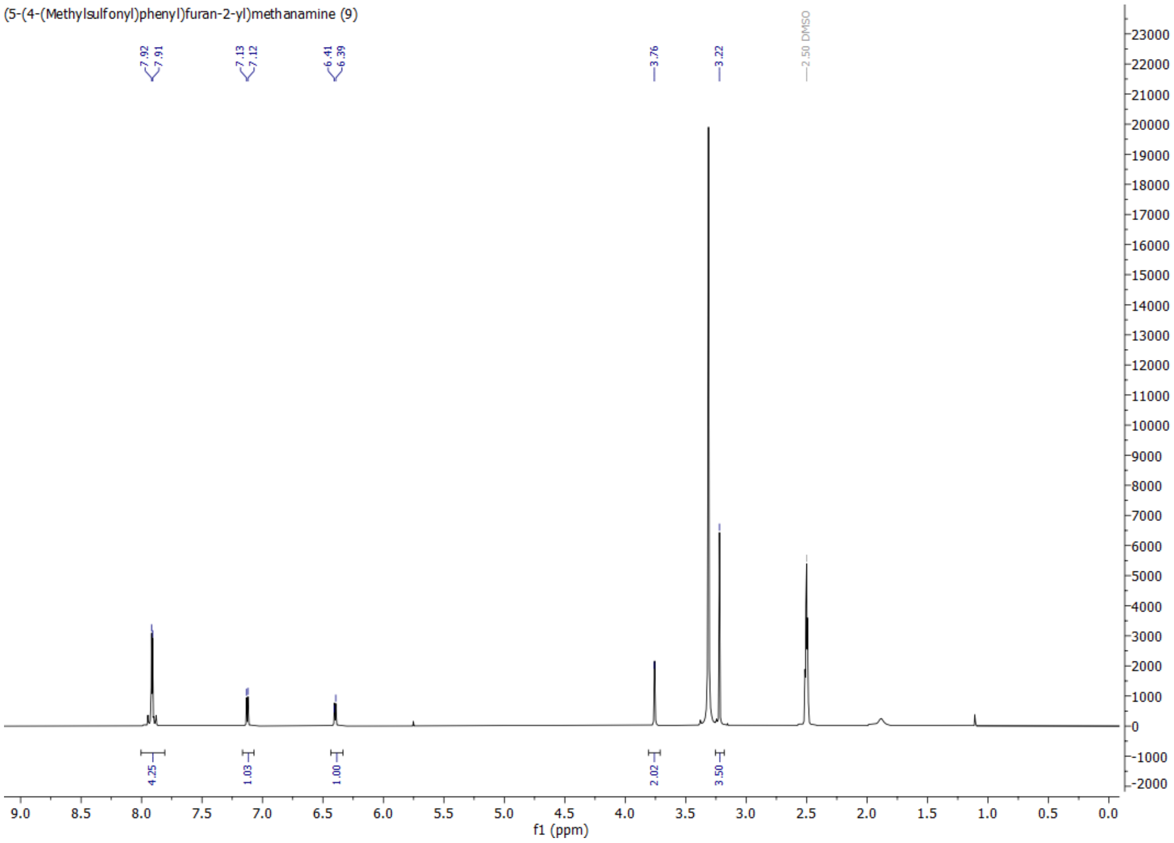

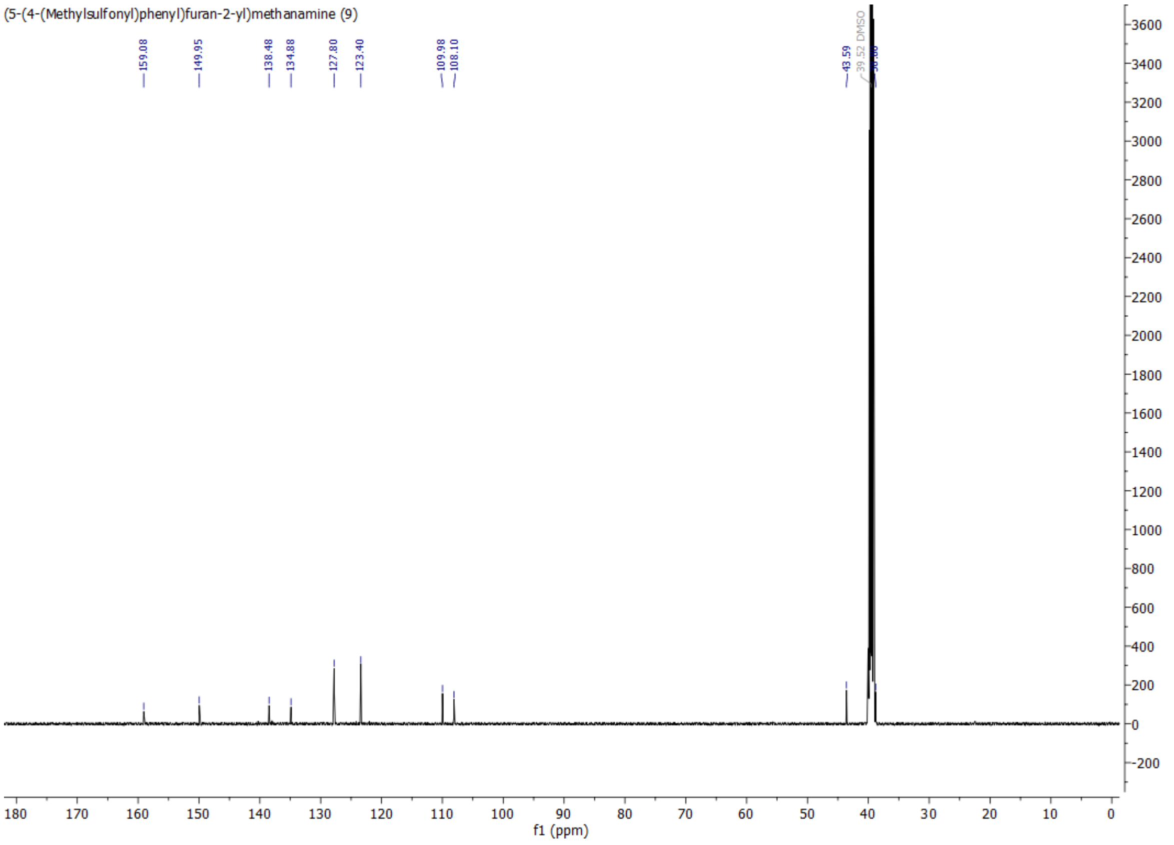

Amino(((5-(4-(methylsulfonyl)phenyl)furan-2-yl)methyl)amino)methaniminium 2,2,2-trifluoro-acetate (D08)

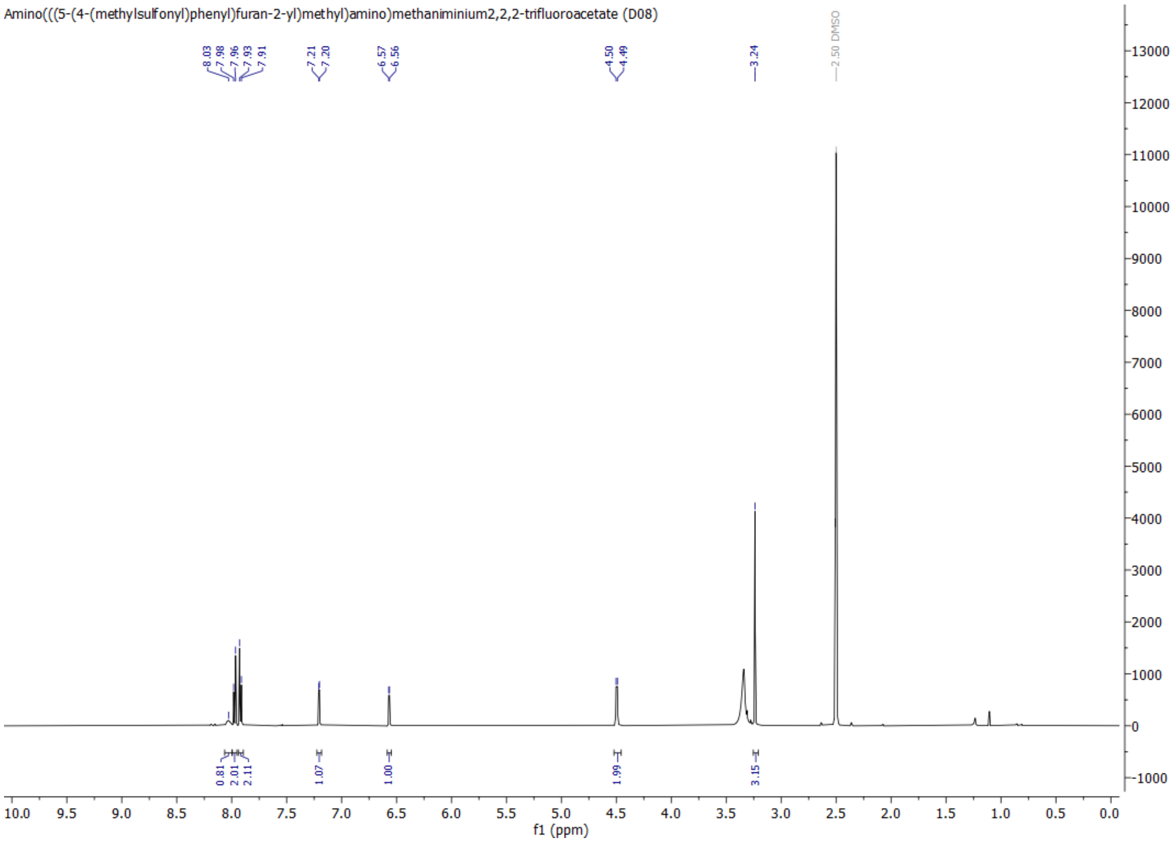

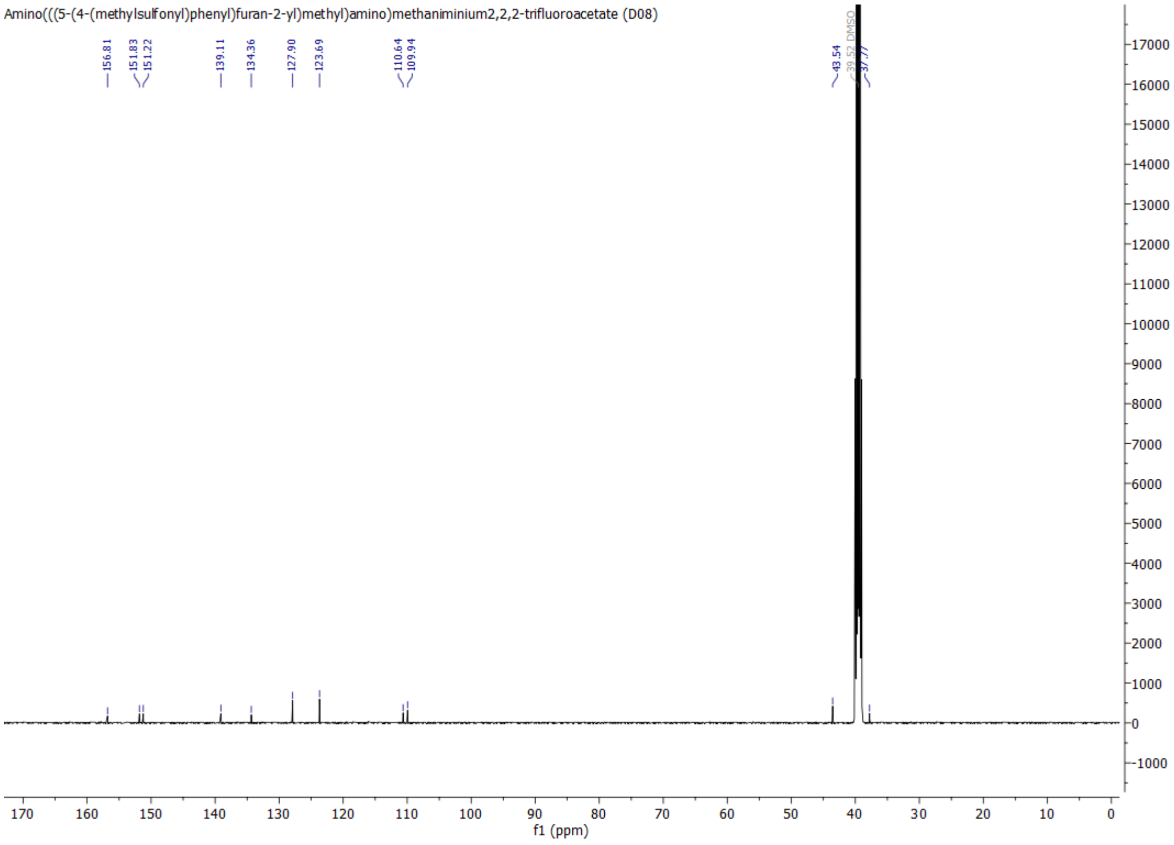

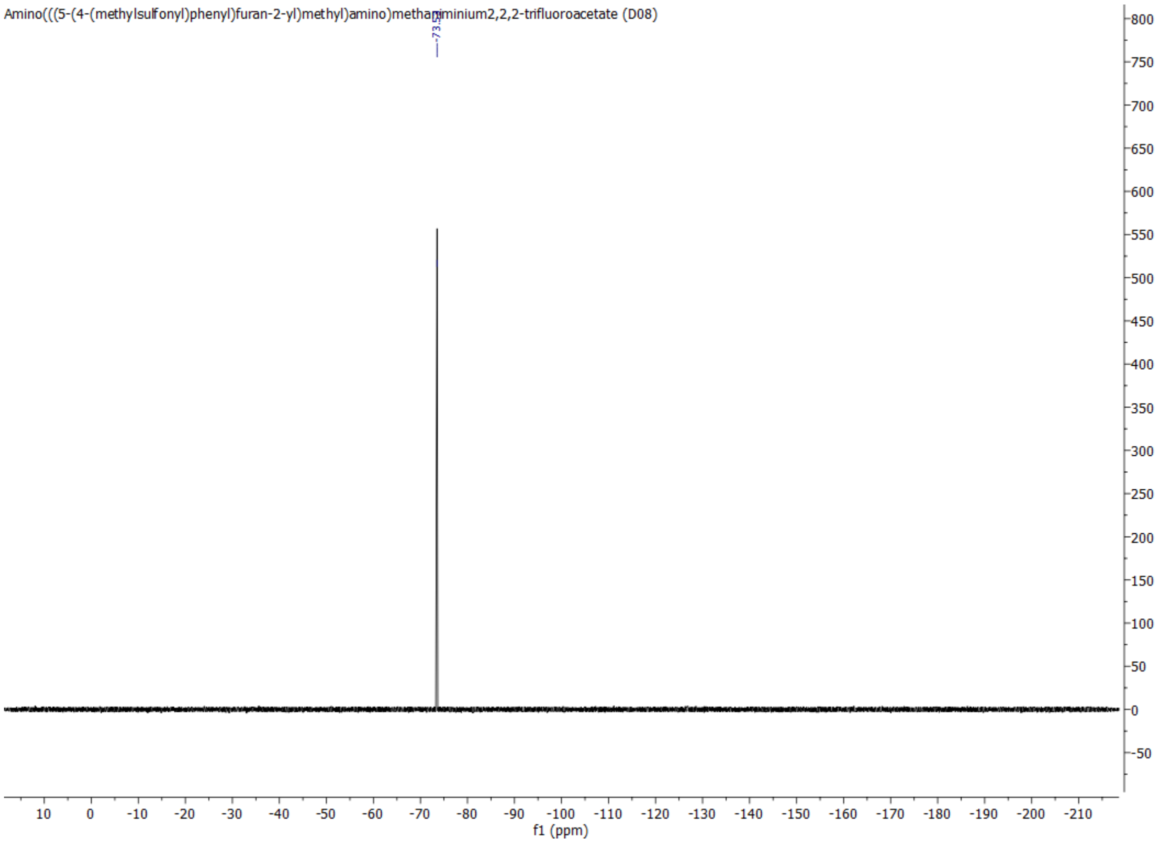

Amino((furan-2-ylmethyl)amino)methaniminium 2,2,2-trifluoroacetate (D09)

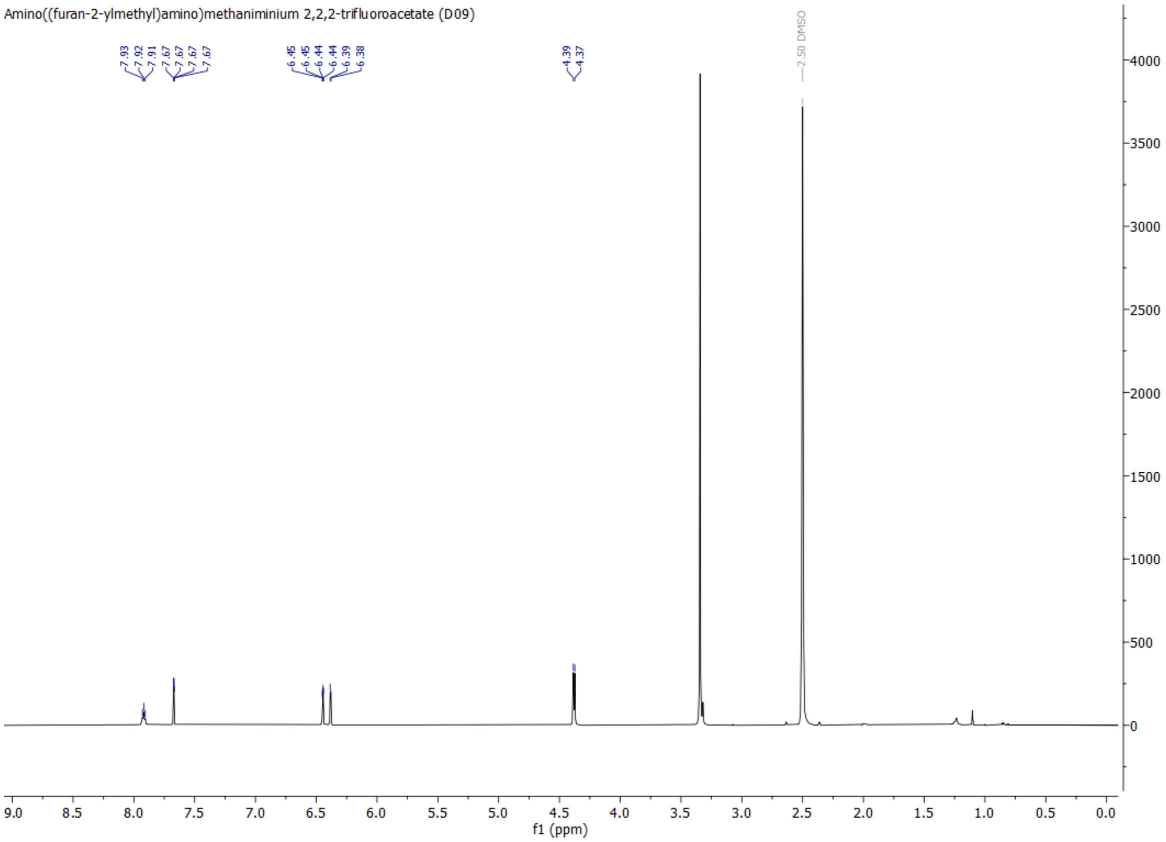

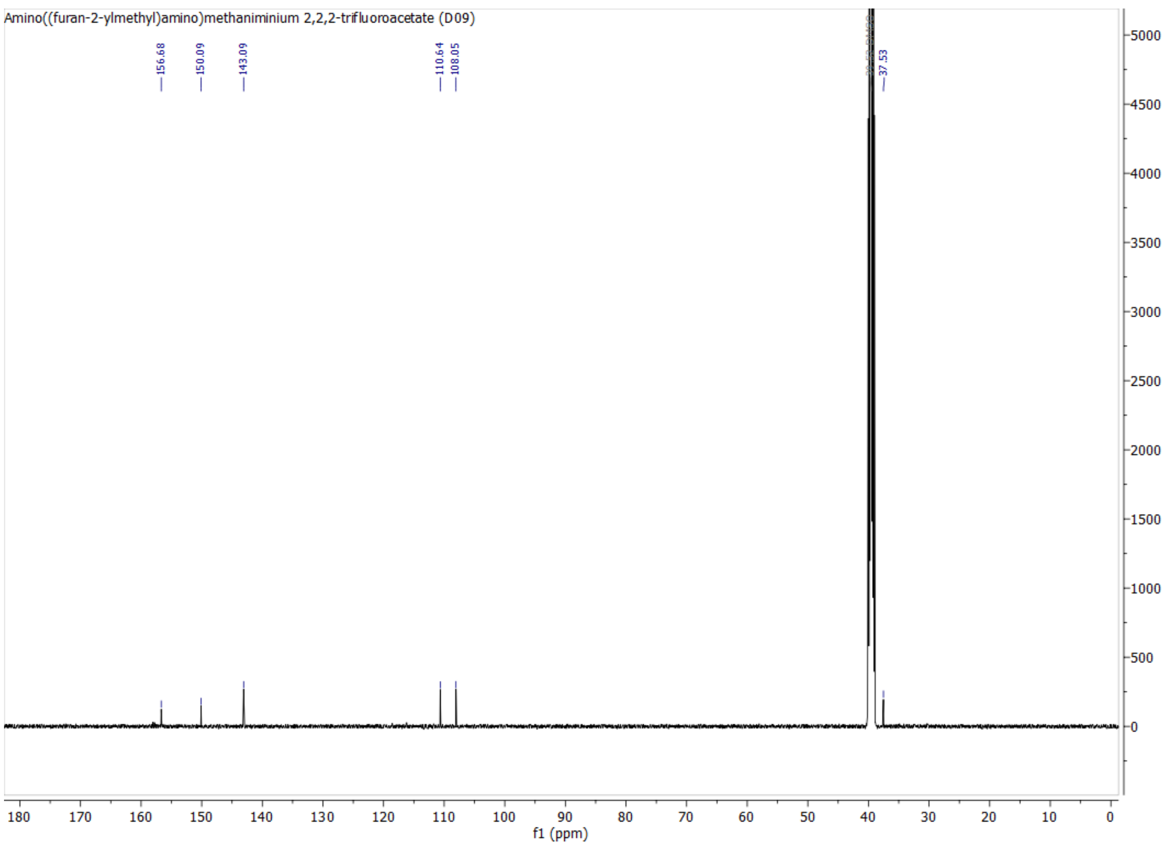

Amino((furan-2-ylmethyl)amino)methaniminium 2,2,2-trifluoroacetate (D09)
156.68
150.09
143.09
110.64
108.05
37.53
f1 (ppm)

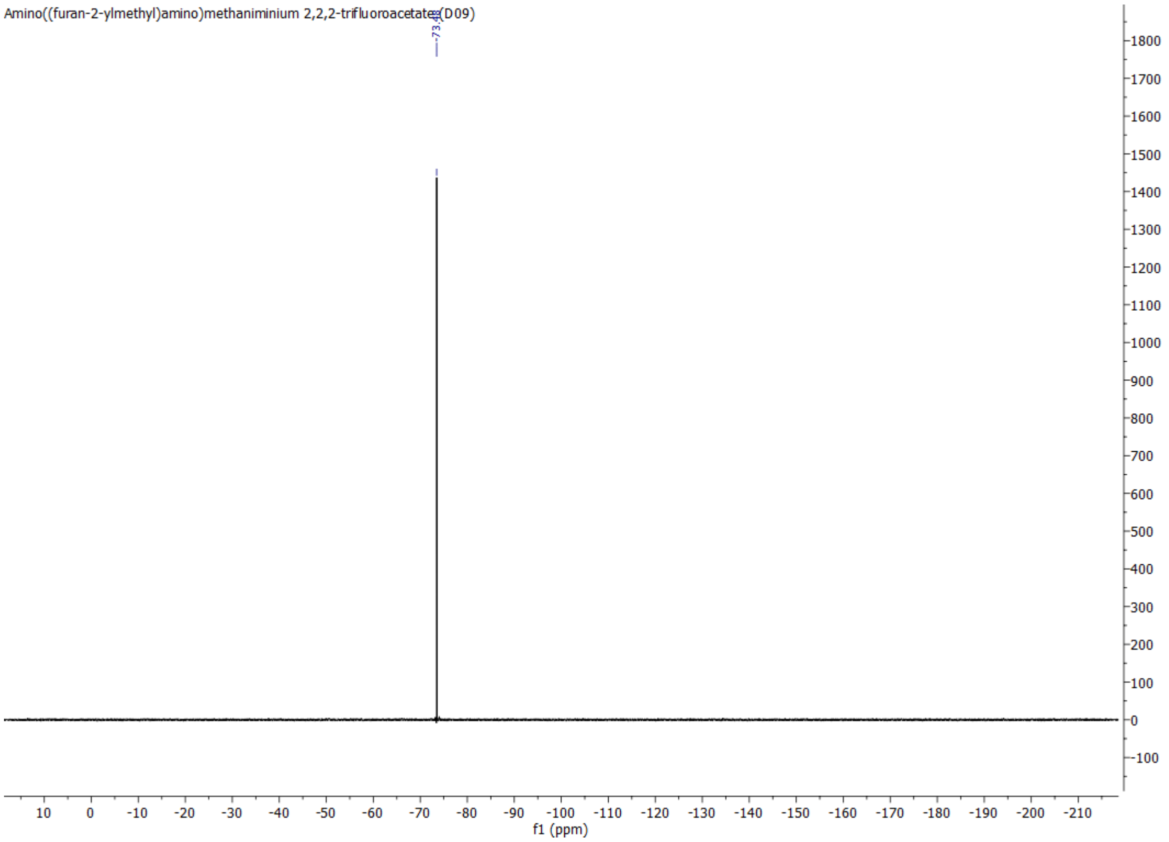

Amino((furan-2-ylmethyl)amino)methaniminium 2,2,2-trifluoroacetate (D09)
f1 (ppm)

5-(4-Bromophenyl)thiophene-2-carbaldehyde (10)

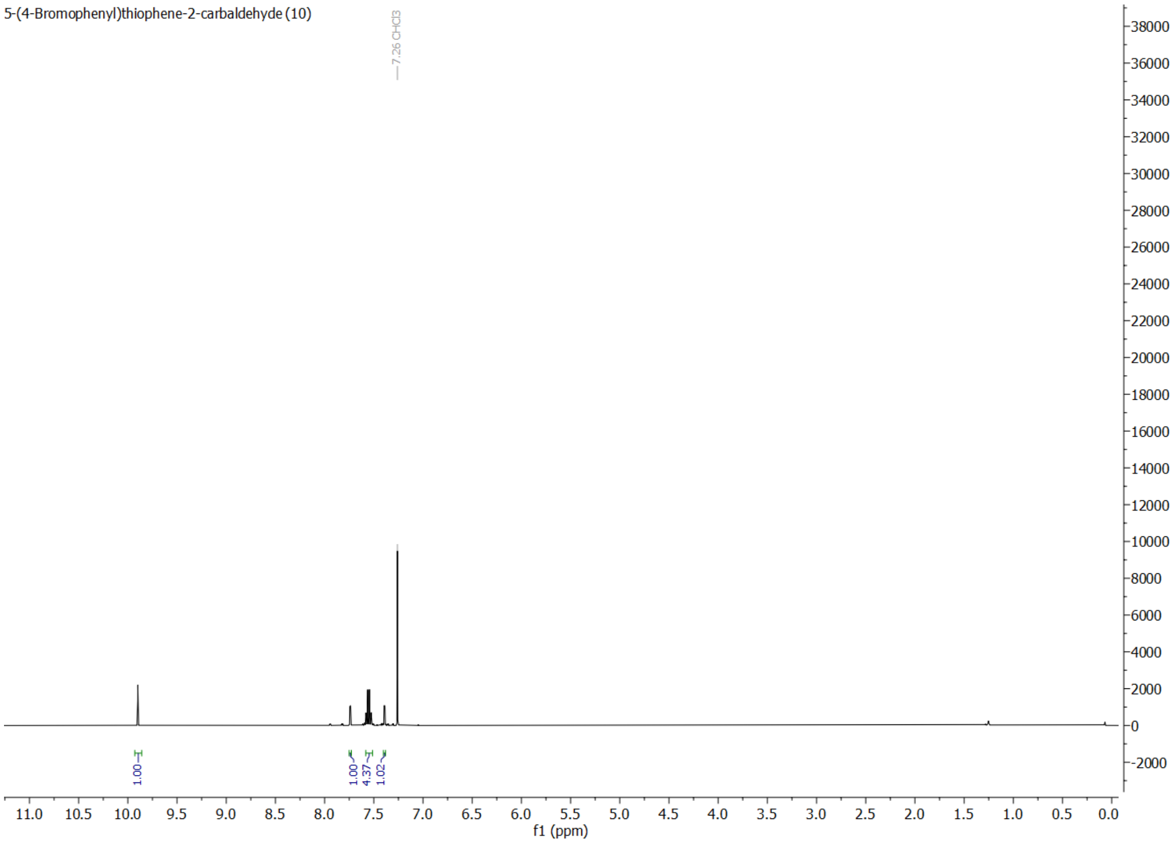

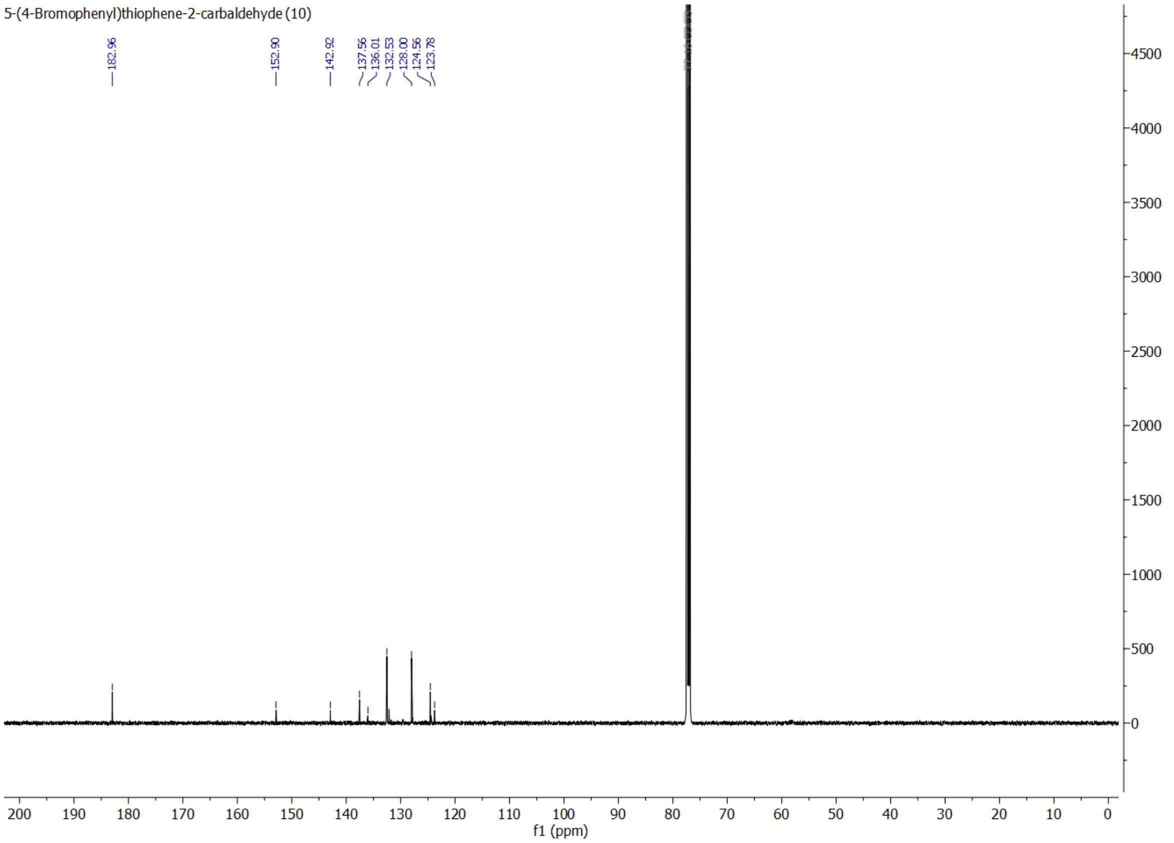

(5-(4-Bromophenyl)thiophen-2-yl)methanamine (12)

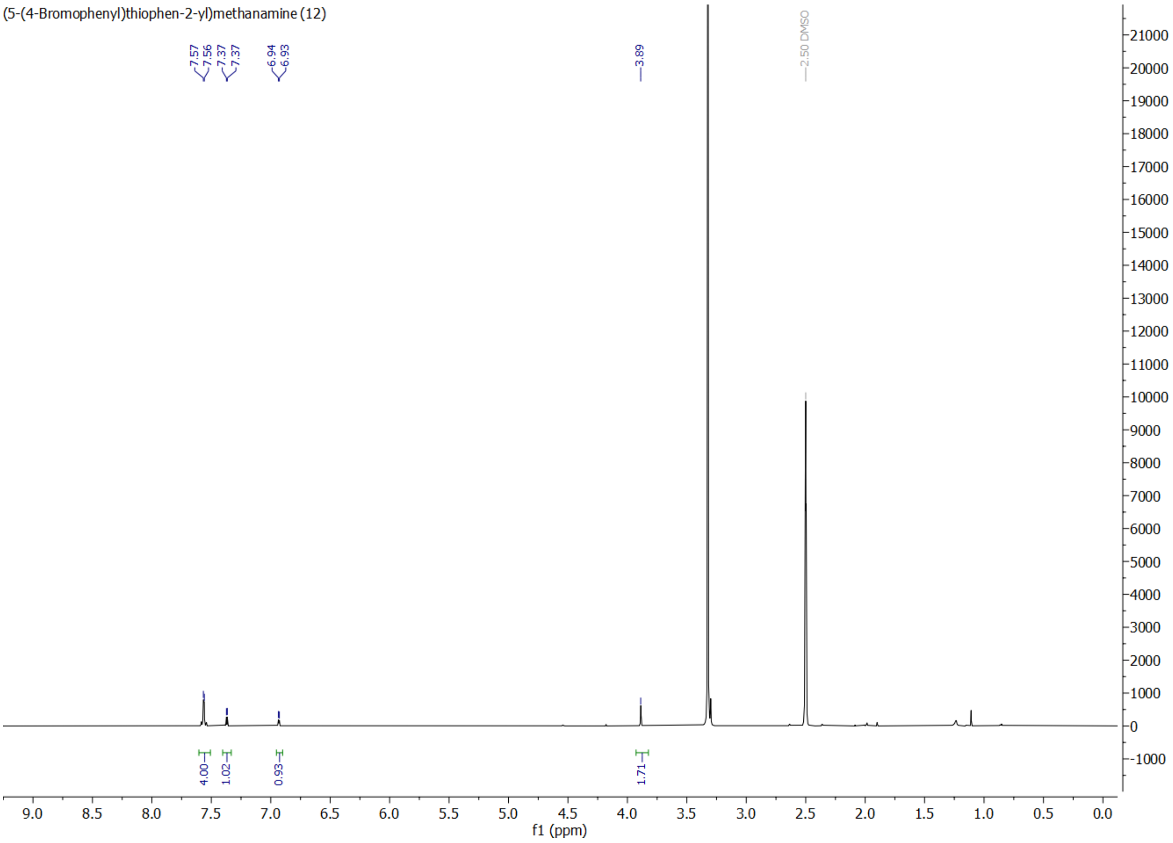

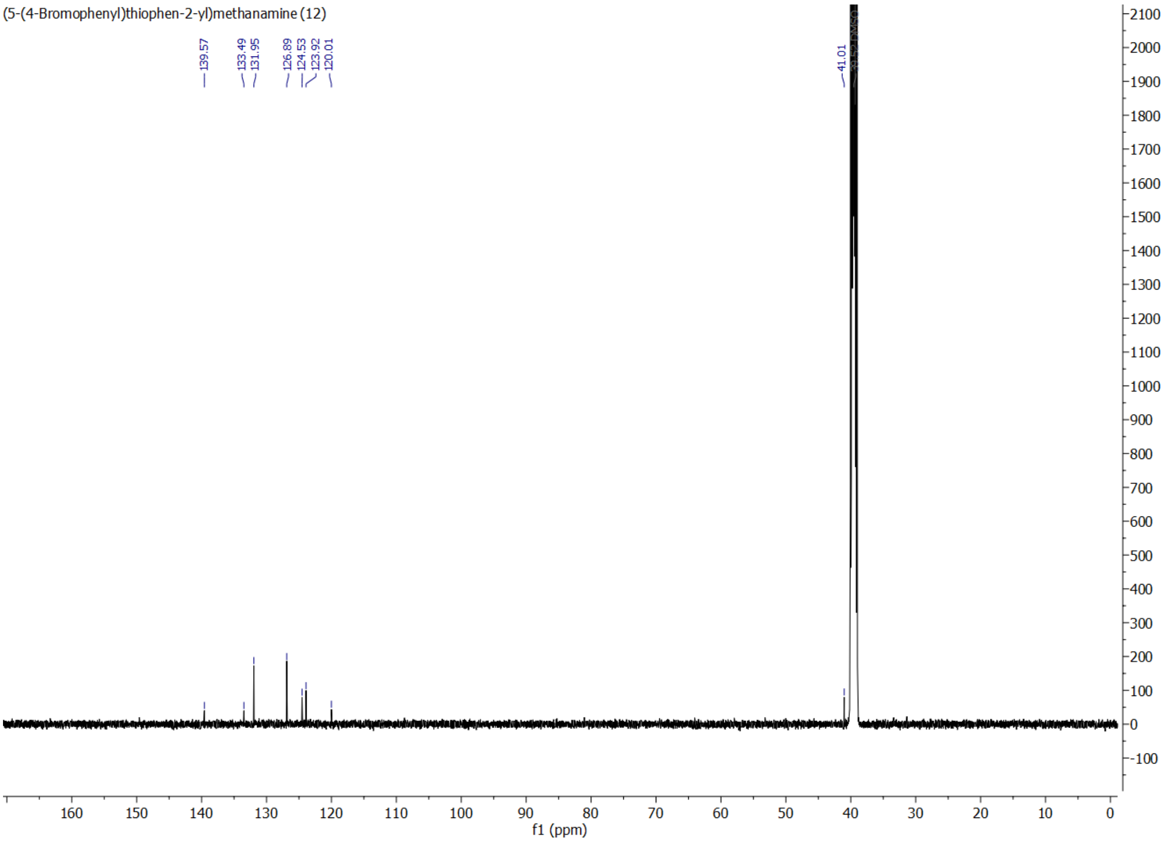

Amino(((5-(4-bromophenyl)thiophen-2-yl)methyl)amino)methaniminium 2,2,2-tri-fluoroacetate (D10)

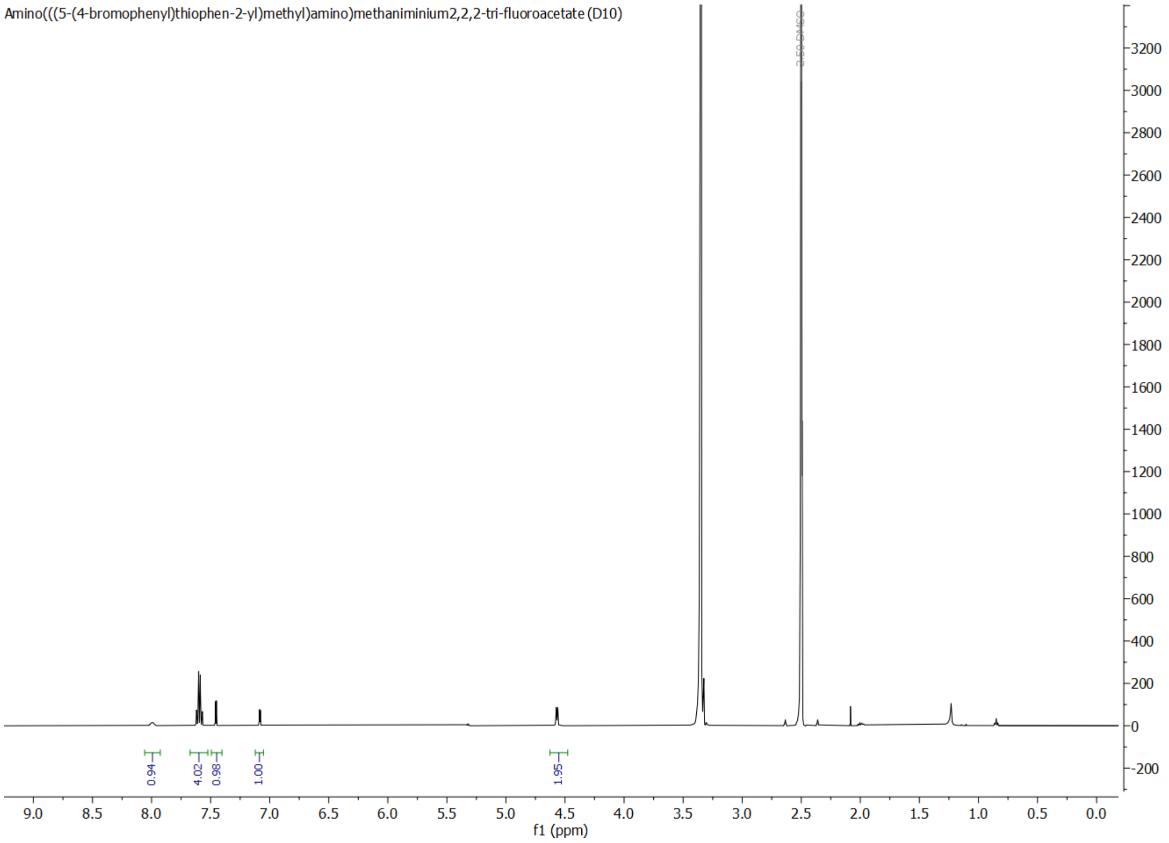

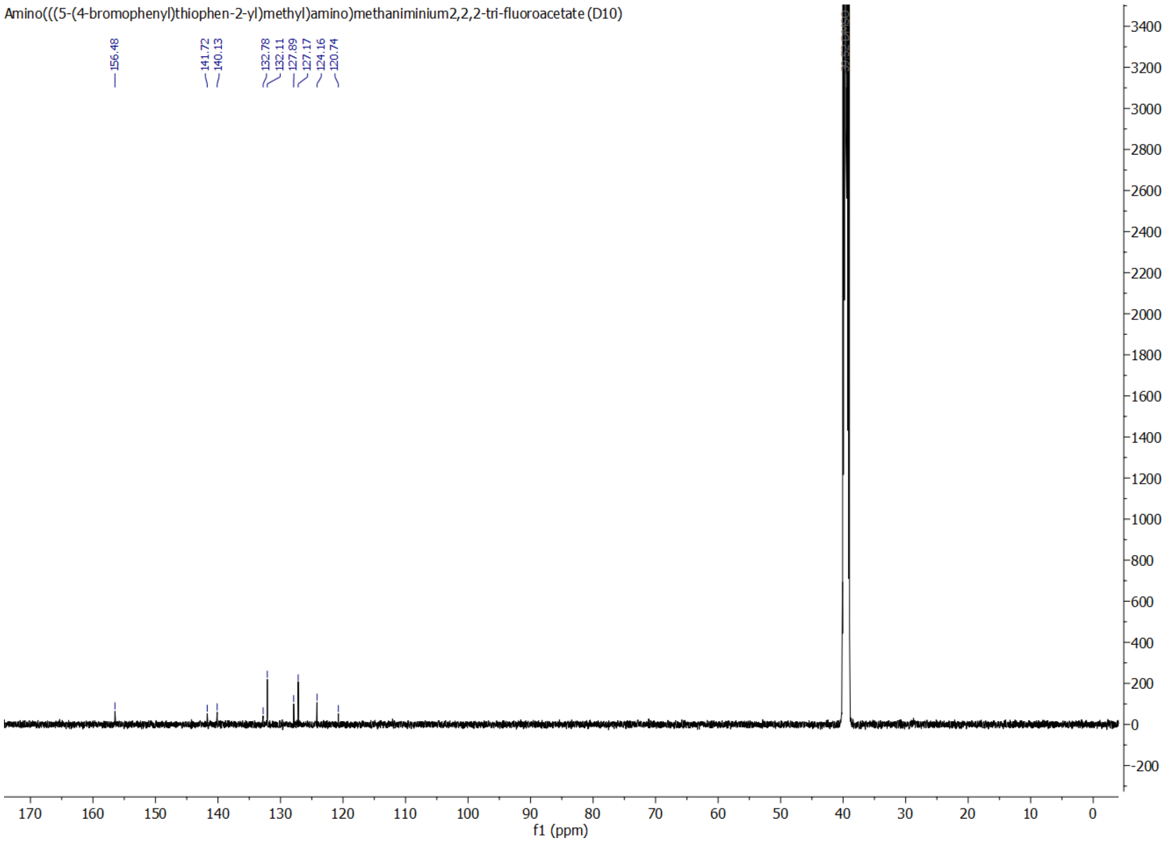

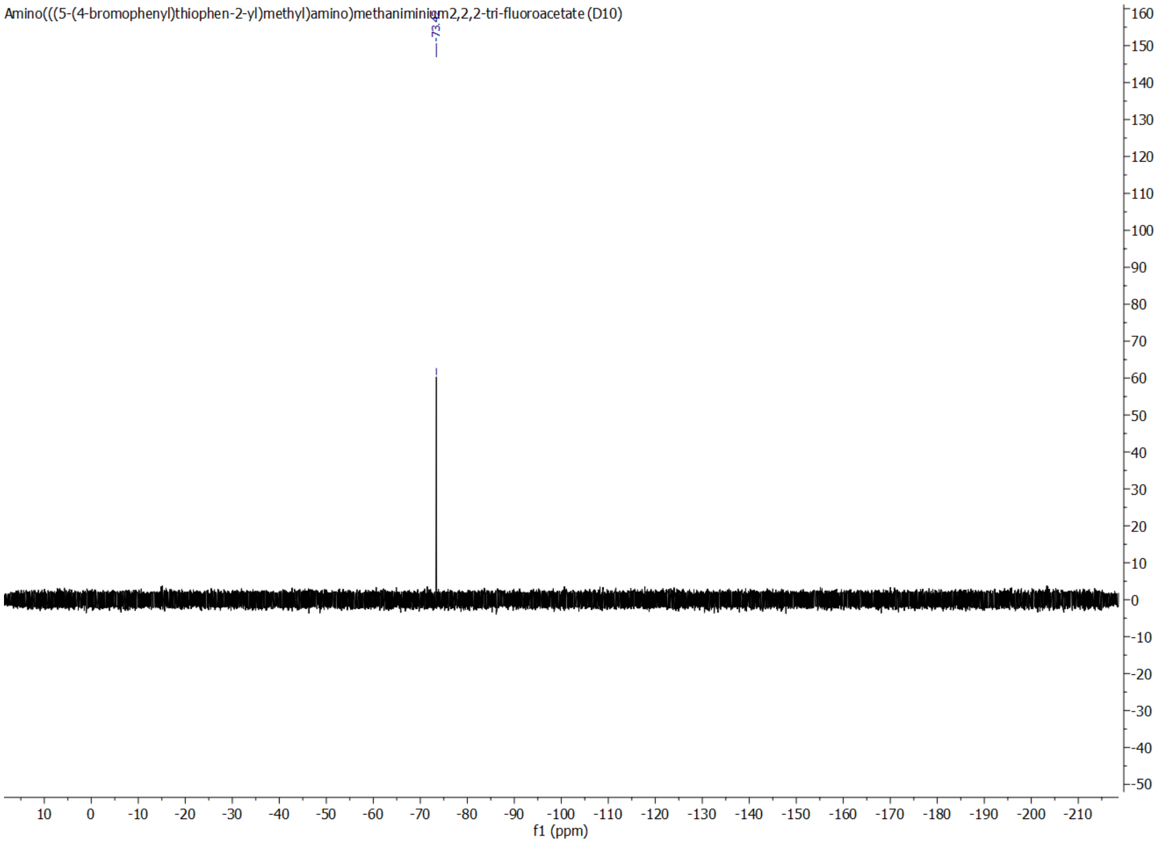

2-(4-Bromophenyl)thiazole-5-carbaldehyde (13)

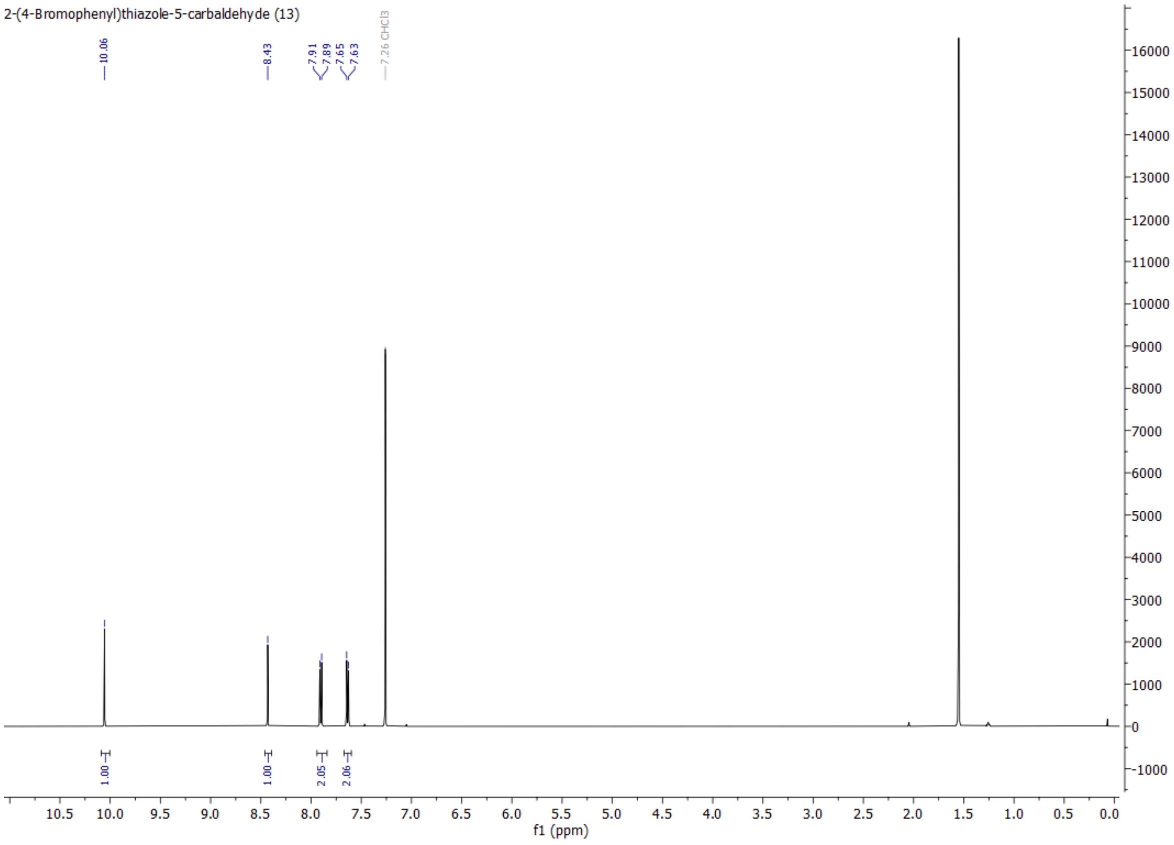

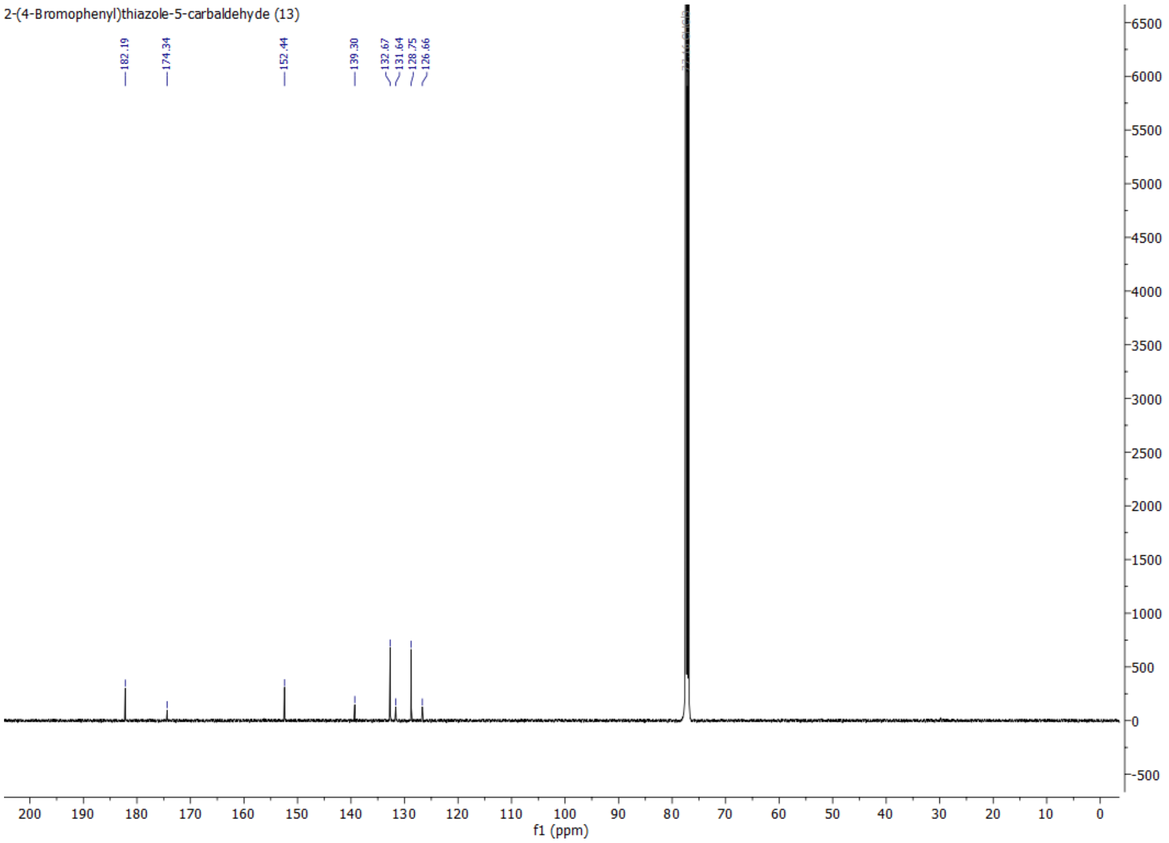

(2-(4-Bromophenyl)thiazol-5-yl)methanamine (15)

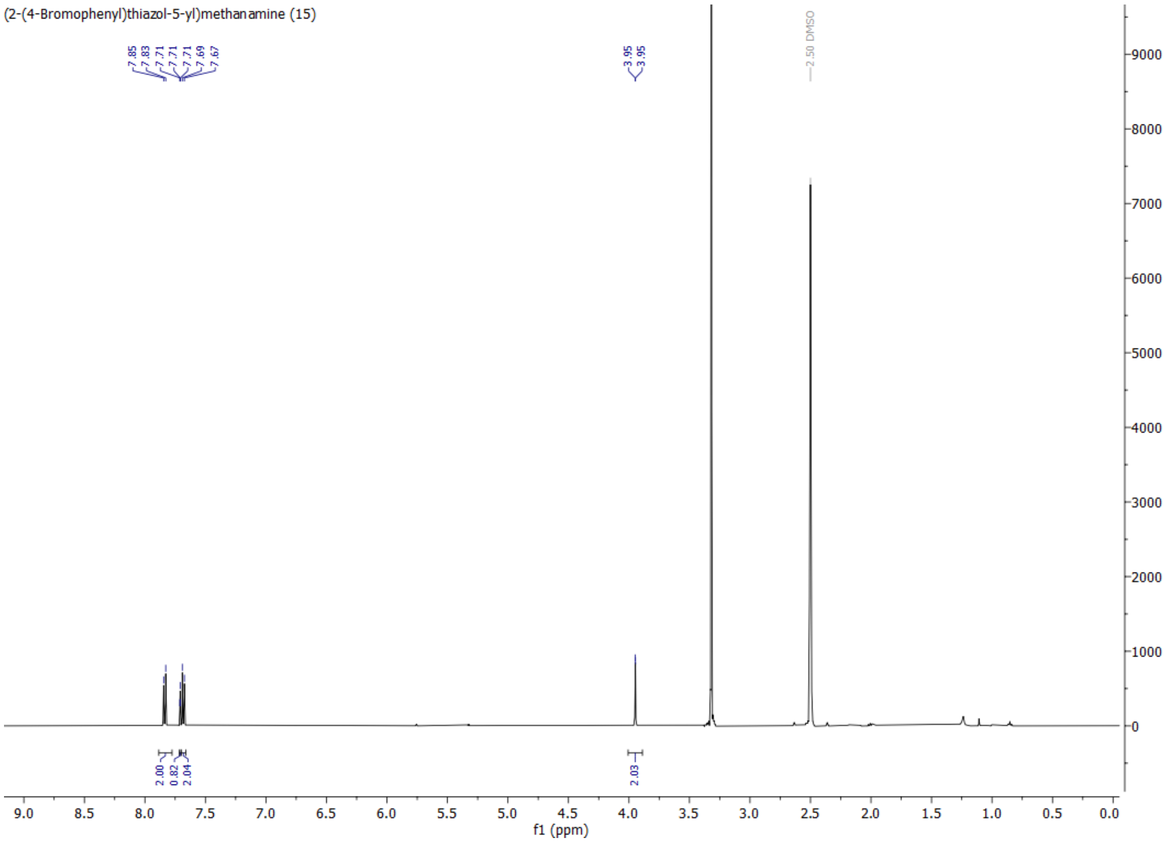

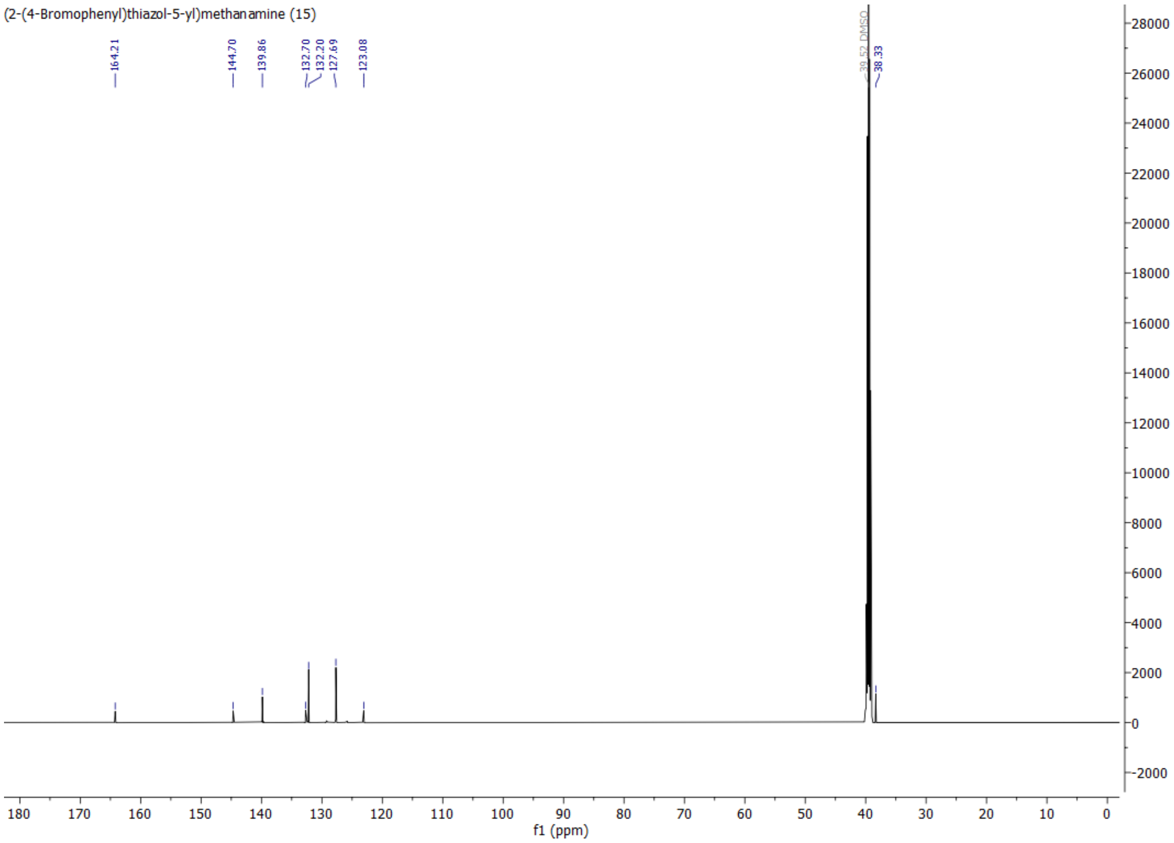

Amino(((2-(4-bromophenyl)thiazol-5-yl)methyl)amino)methaniminium 2,2,2-trifluoro-acetate (D11)

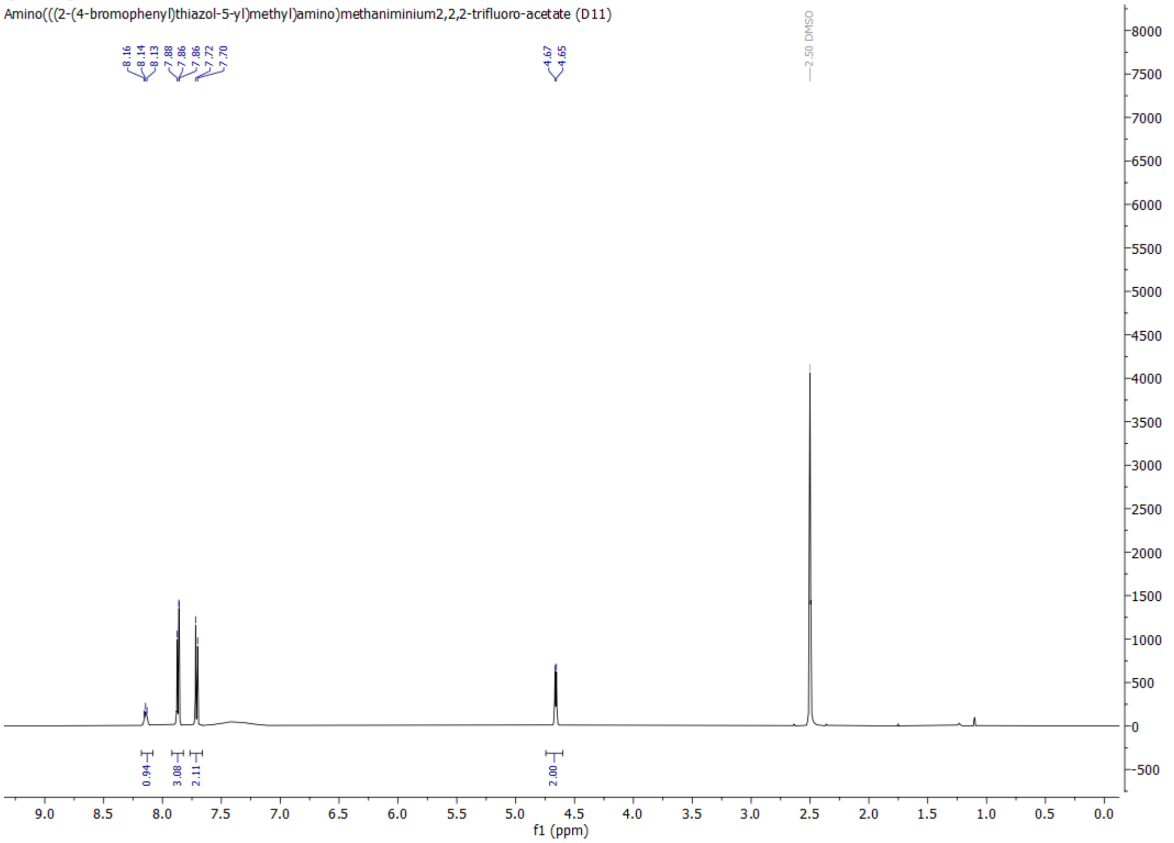

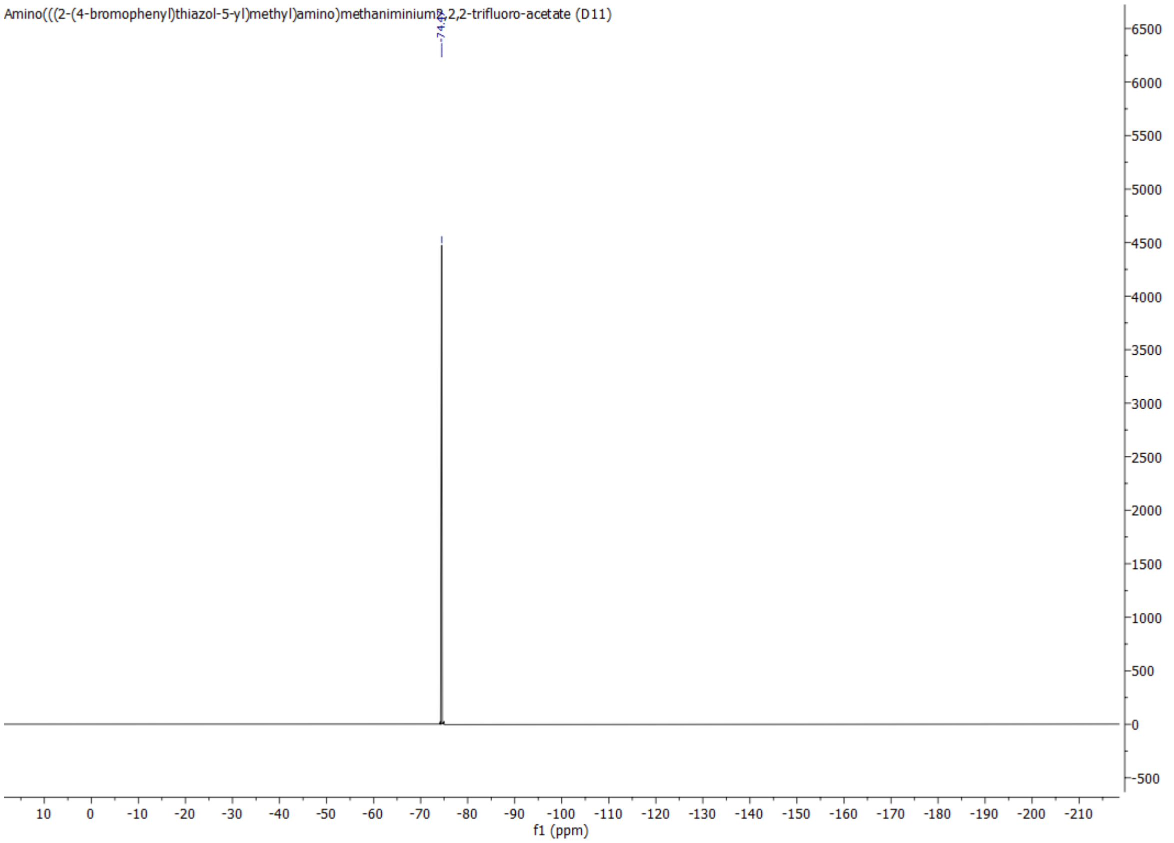

1-(4-Bromophenyl)-1*H*-pyrazole-5-carbaldehyde (16)

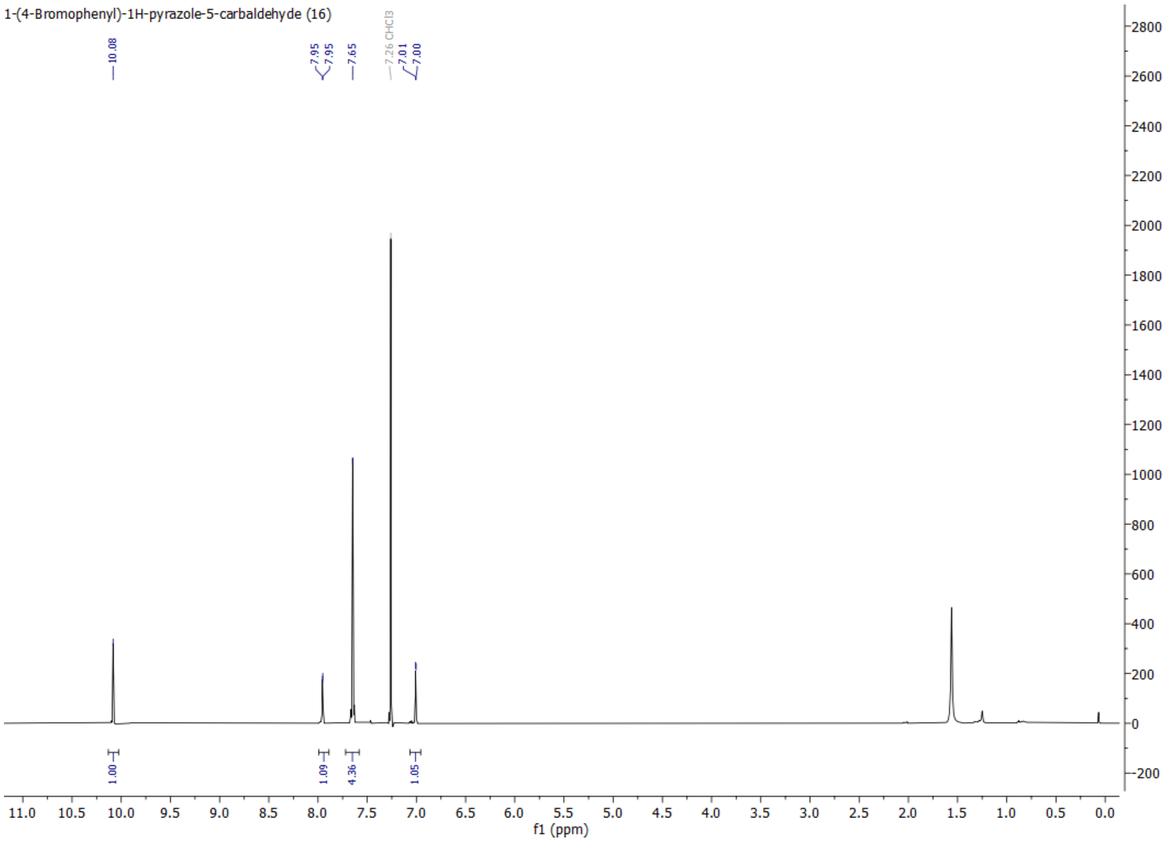

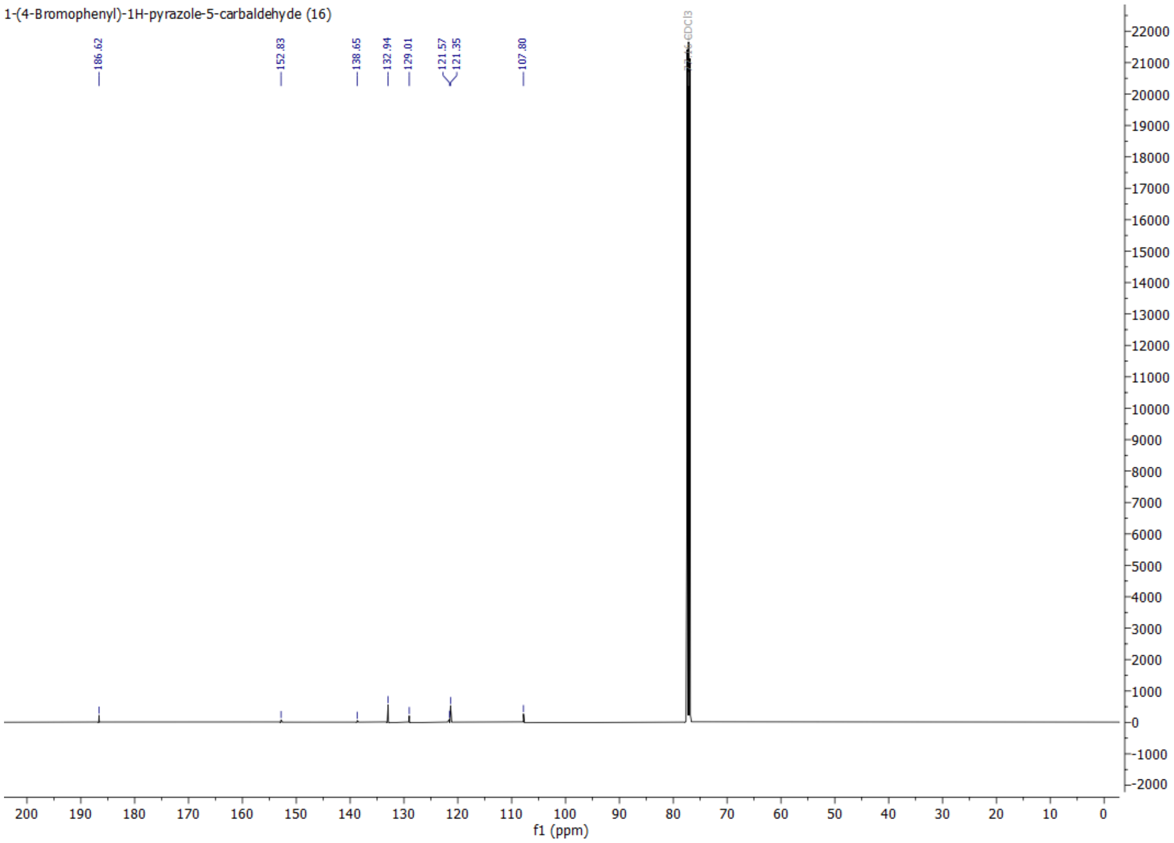

(1-(4-Bromophenyl)-1*H*-pyrazol-5-yl)methanamine (18)

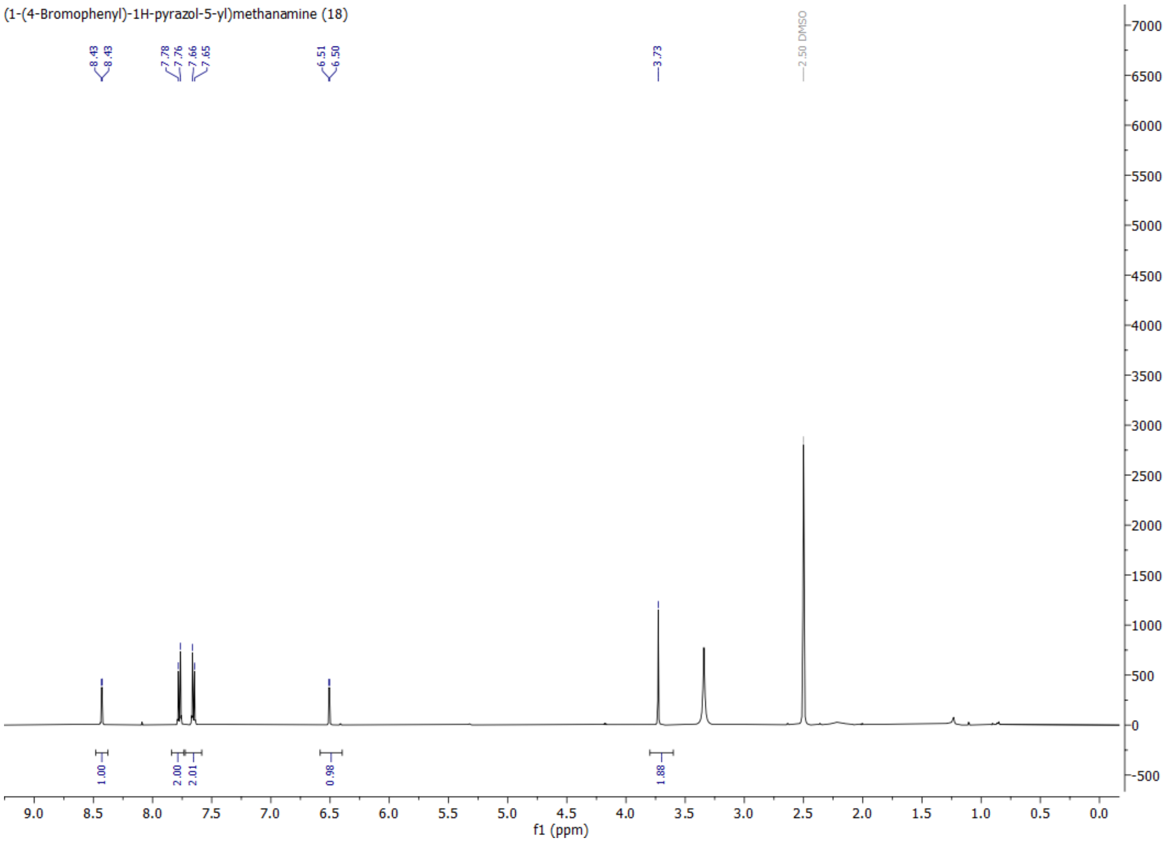

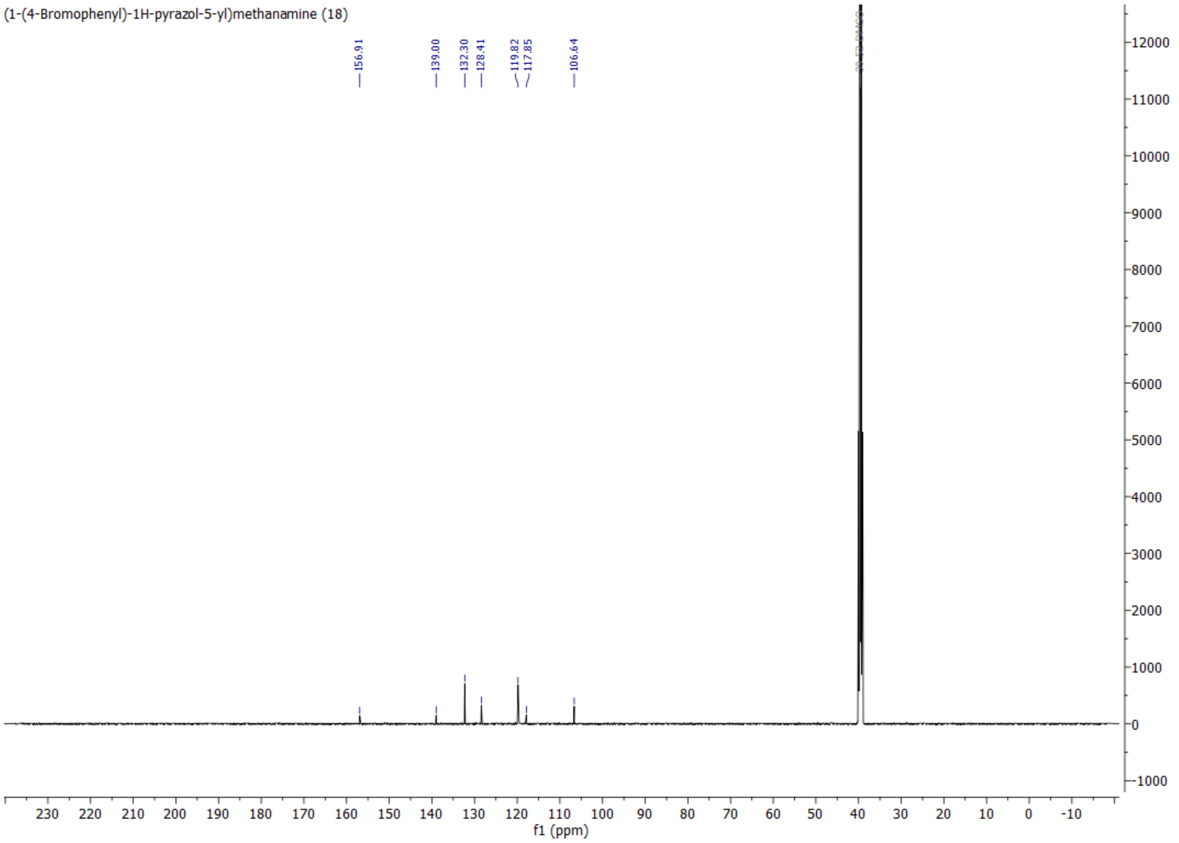

Amino(((1-(4-bromophenyl)-1*H*-pyrazol-5-yl)methyl)amino)methaniminium 2,2,2-trifluoro-acetate (D12)

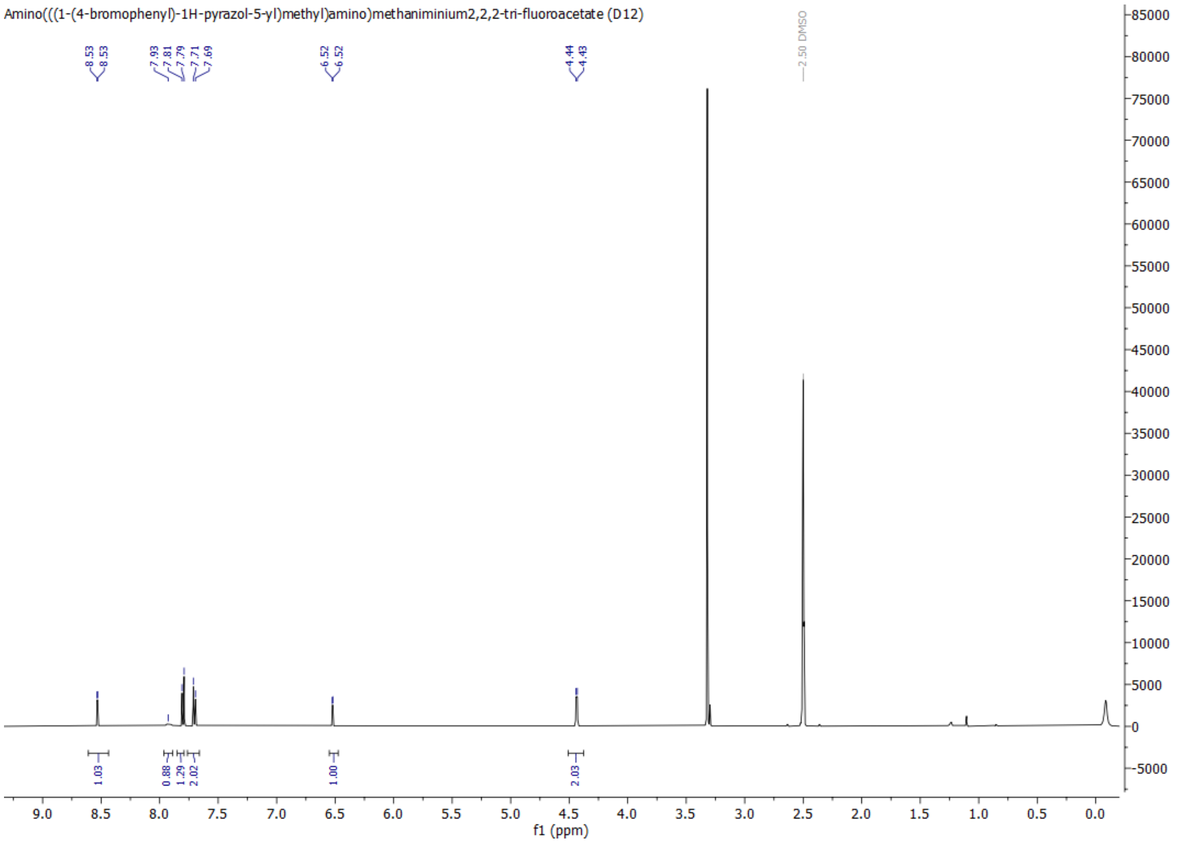

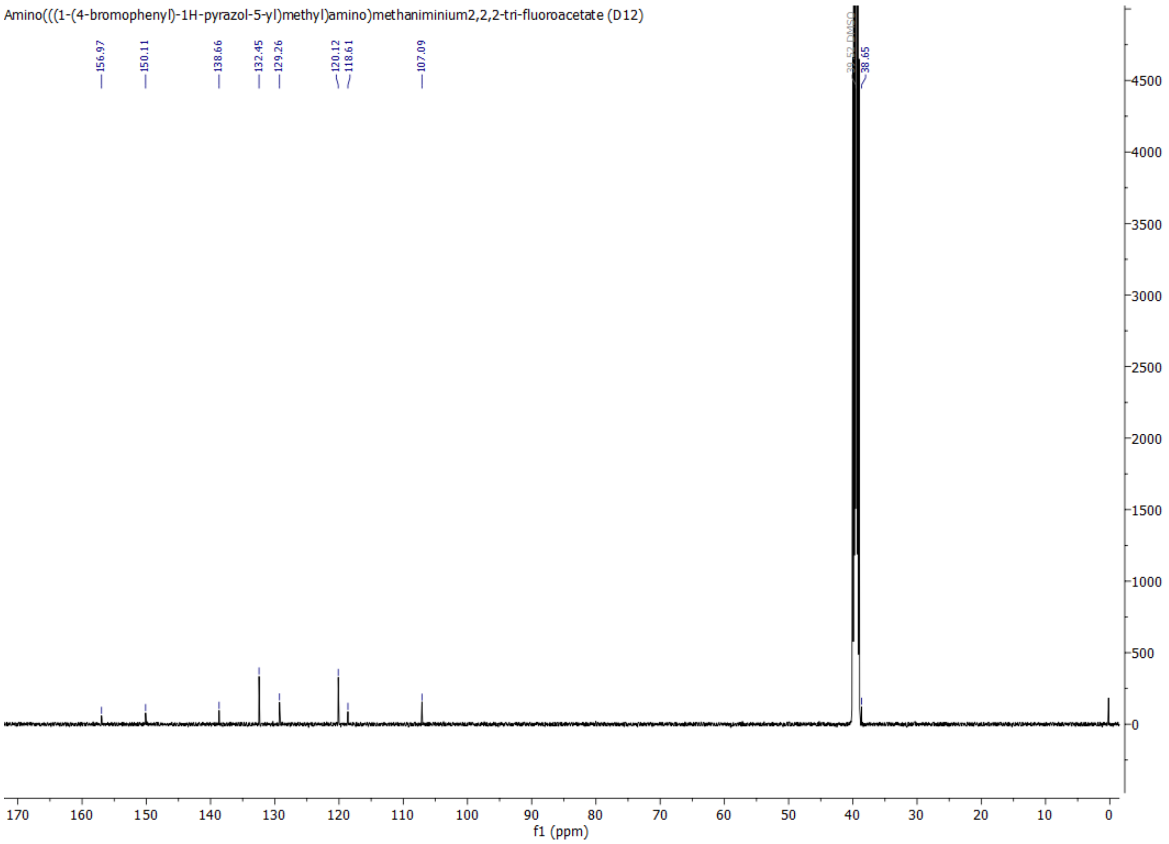
Amino(((1-(4-bromophenyl)-1H-pyrazol-5-yl)methyl)amino)methaniminium2,2,2-tri-fluoroacetate (D12)
156.97
150.11
138.66
132.45
129.26
120.12
118.61
107.09
39.52 DMSO
38.65
f1 (ppm)

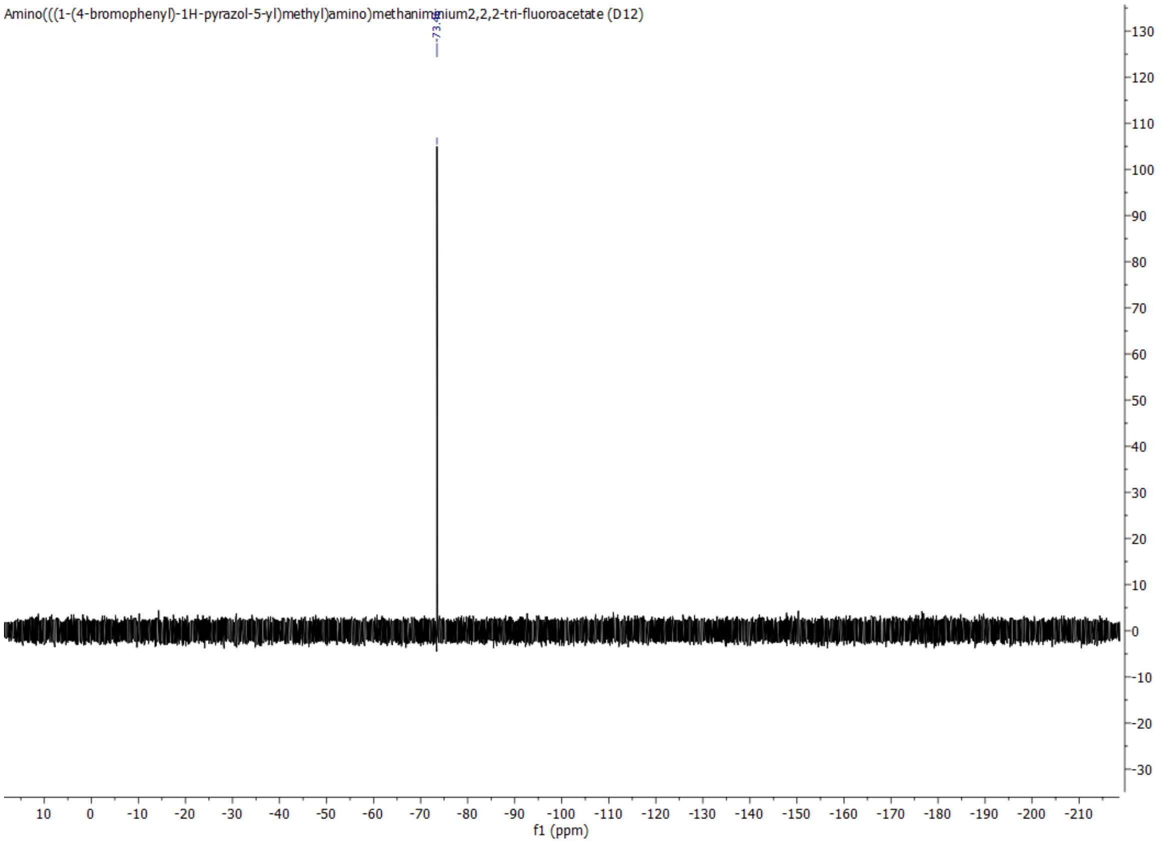
Amino(((1-(4-bromophenyl)-1H-pyrazol-5-yl)methyl)amino)methaniminium2,2,2-tri-fluoroacetate (D12)
f1 (ppm)

5-(Pyridin-2-yl)thiophene-2-carbaldehyde (19)

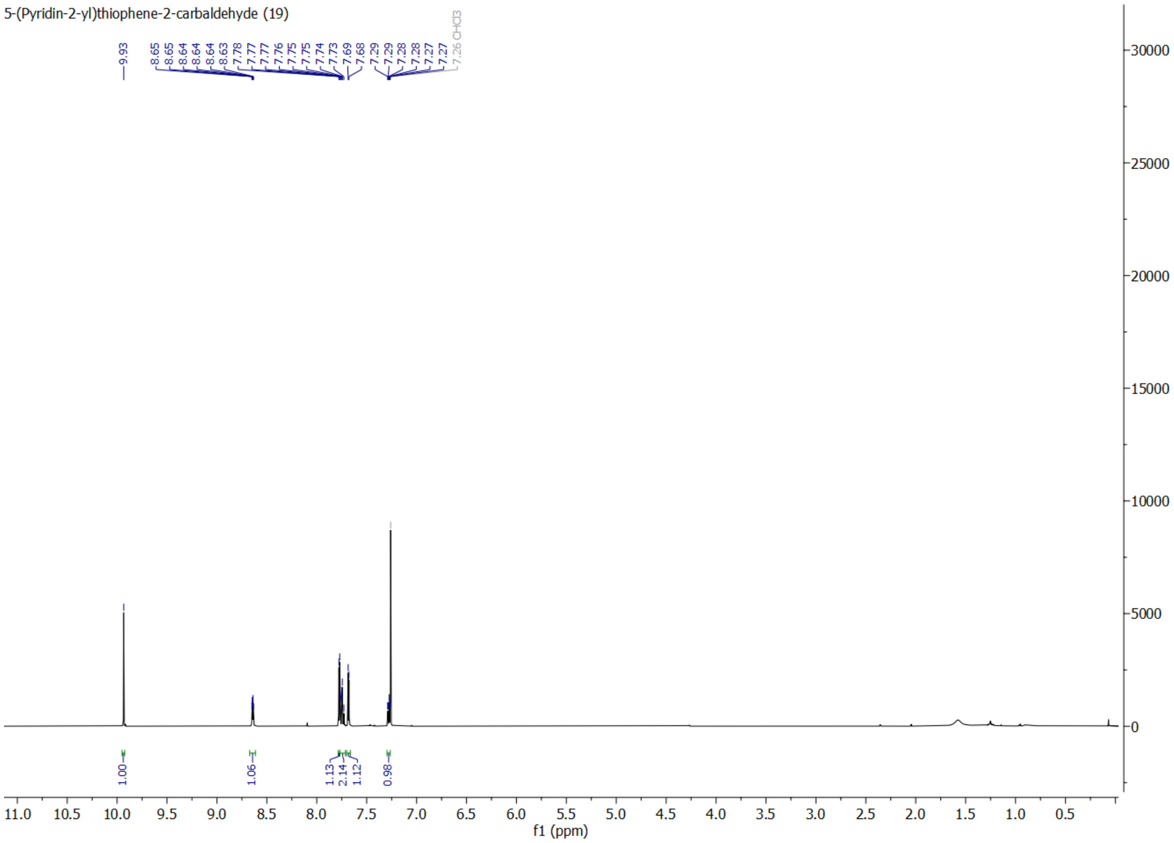

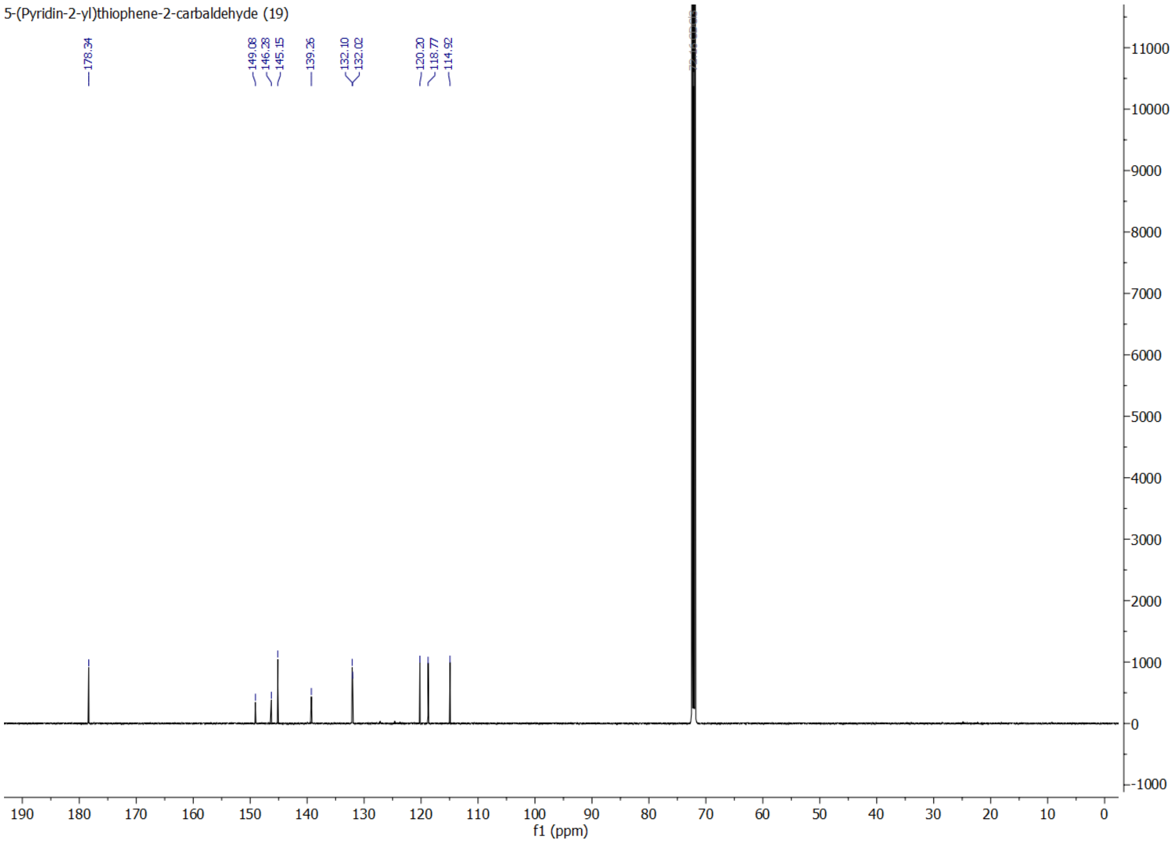

(5-(Pyridin-2-yl)thiophen-2-yl)methanamine (21)

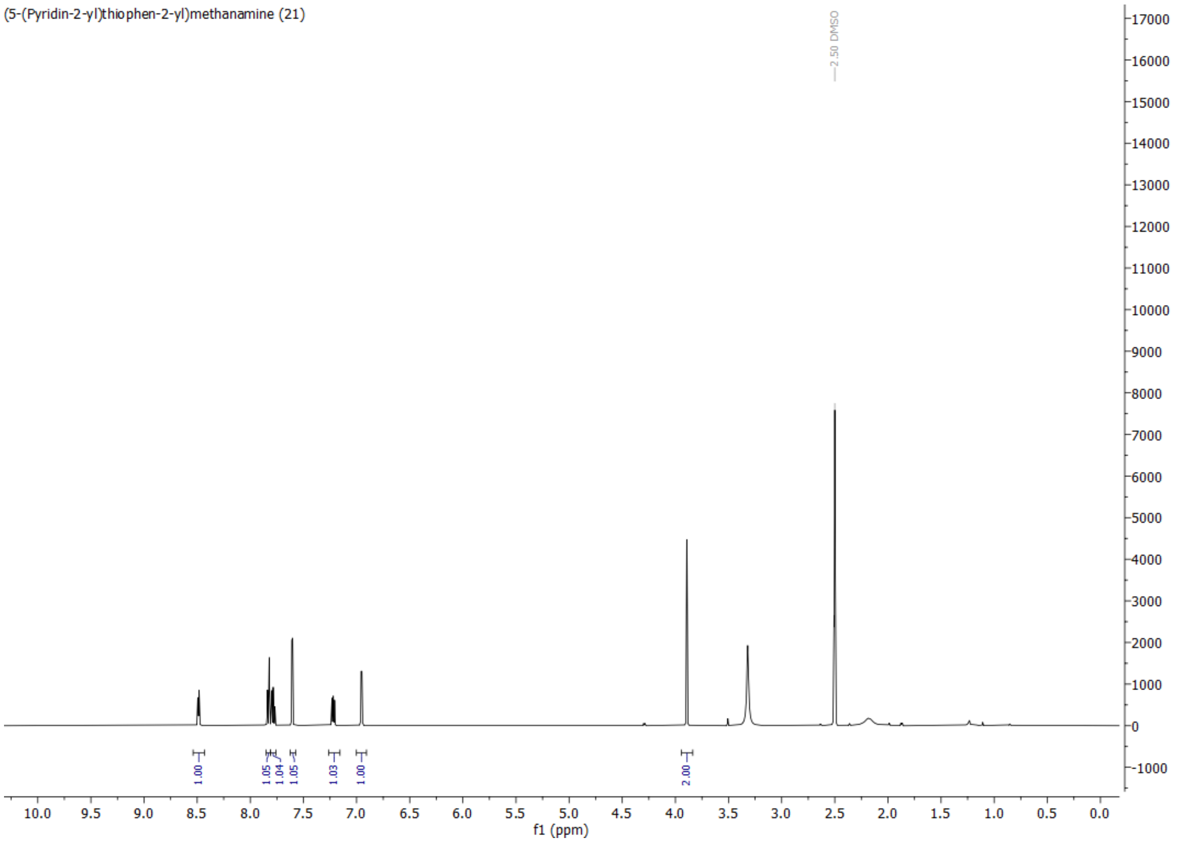

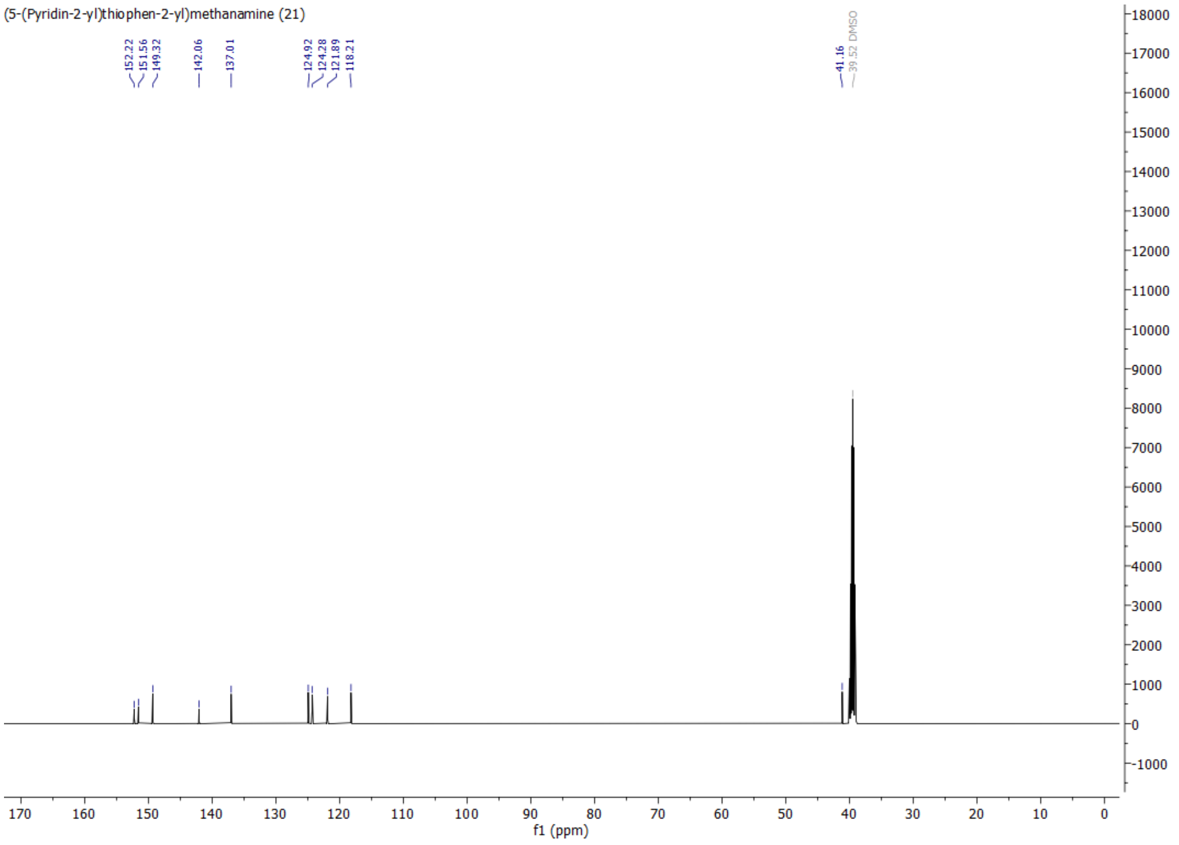

Amino(((5-(pyridin-2-yl)thiophen-2-yl)methyl)amino)methaniminium 2,2,2-trifluoroacetate (D13)

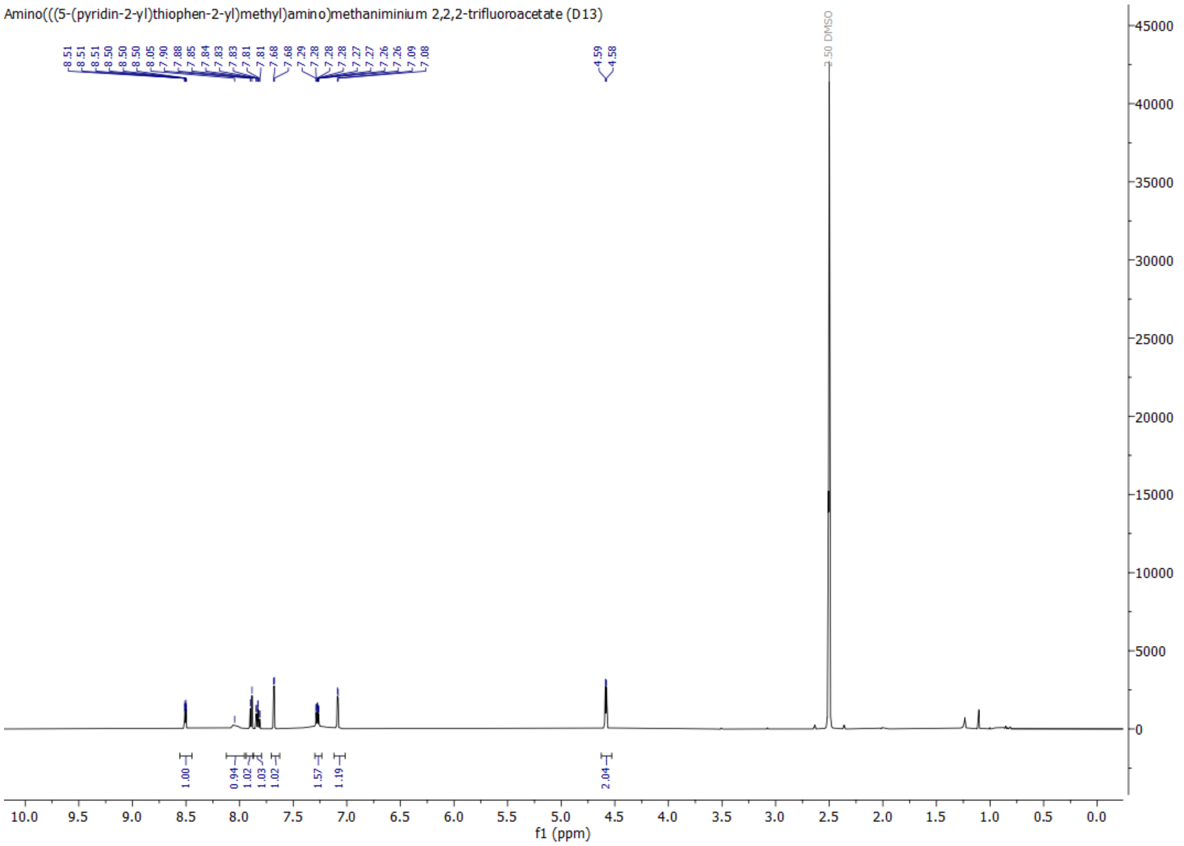

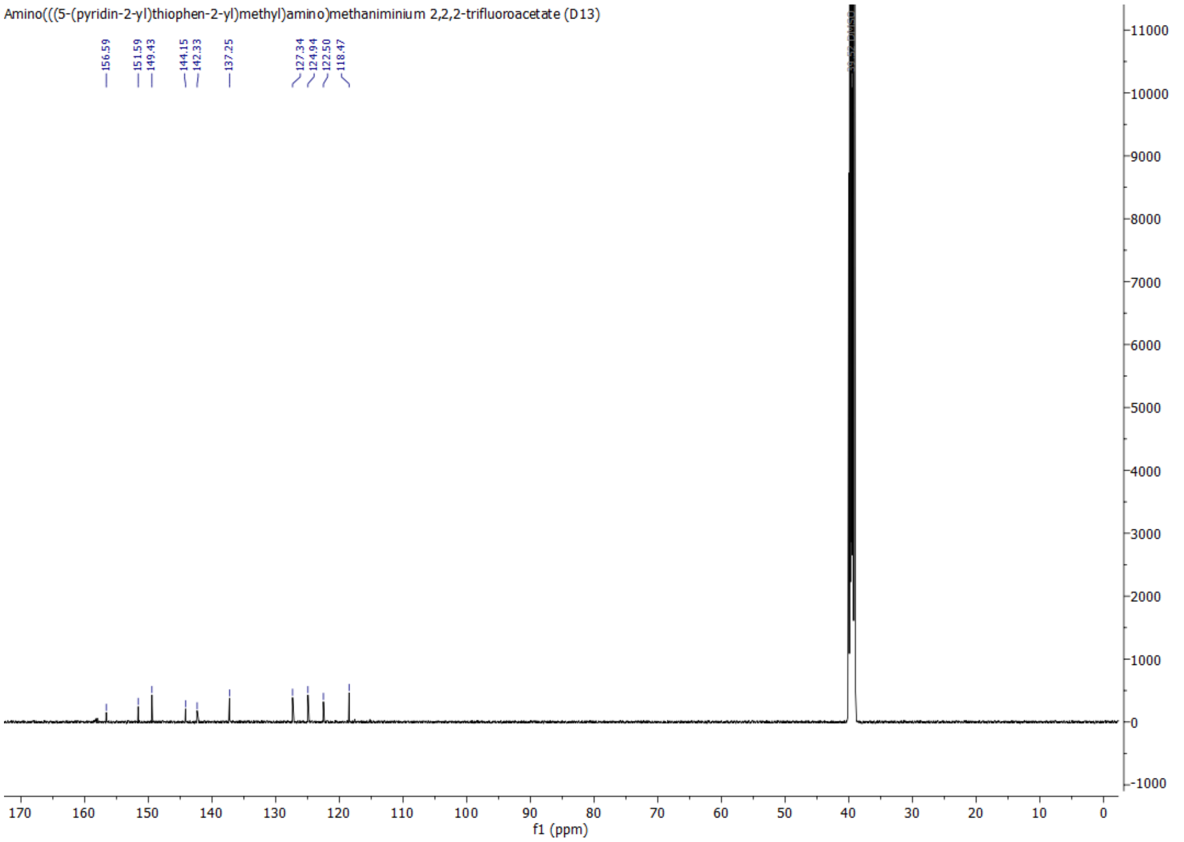

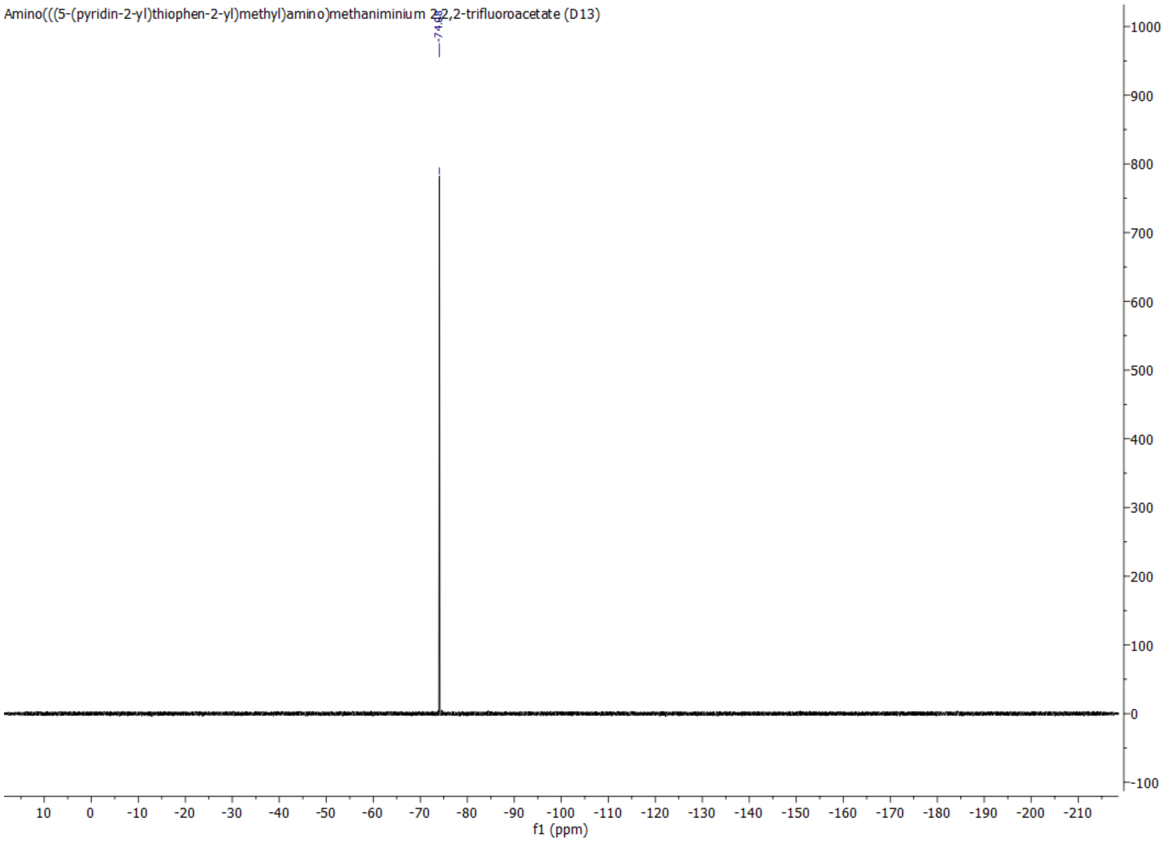

Ethyl 5-(4-bromophenyl)furan-2-carboxylate (22)

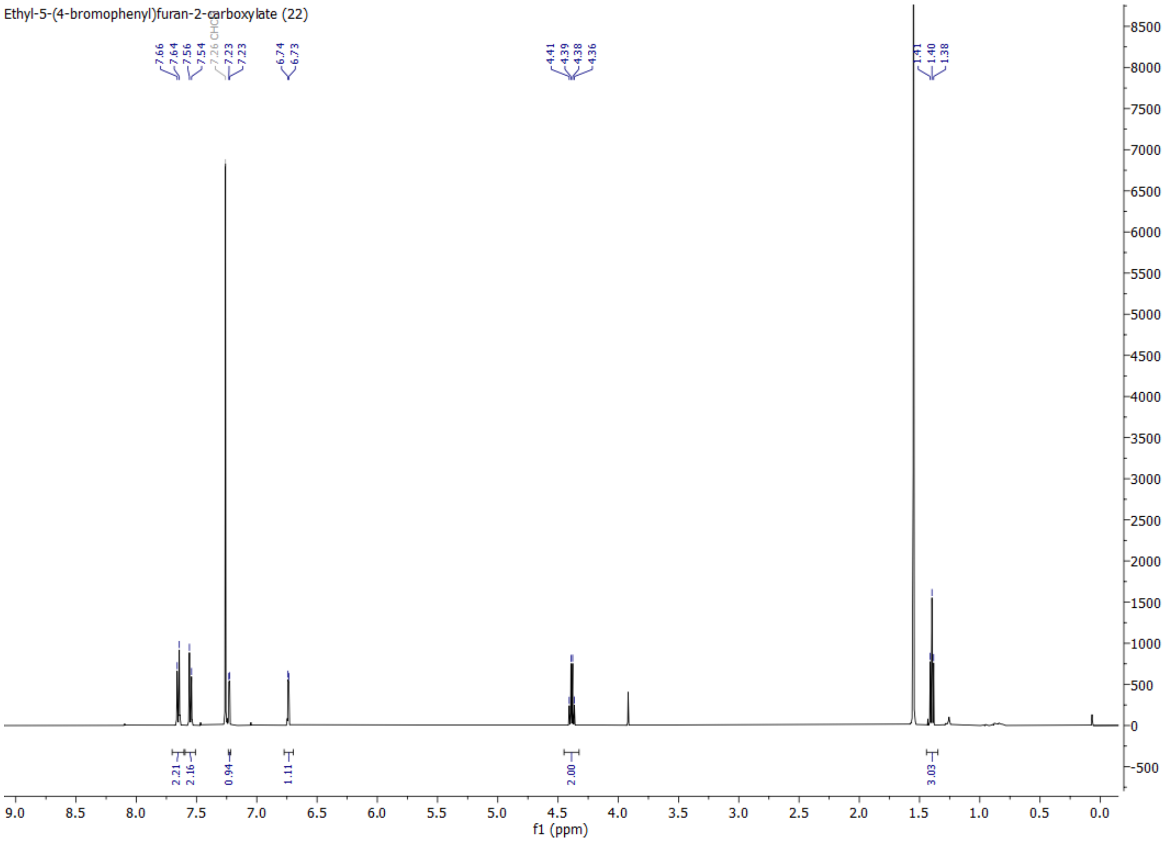

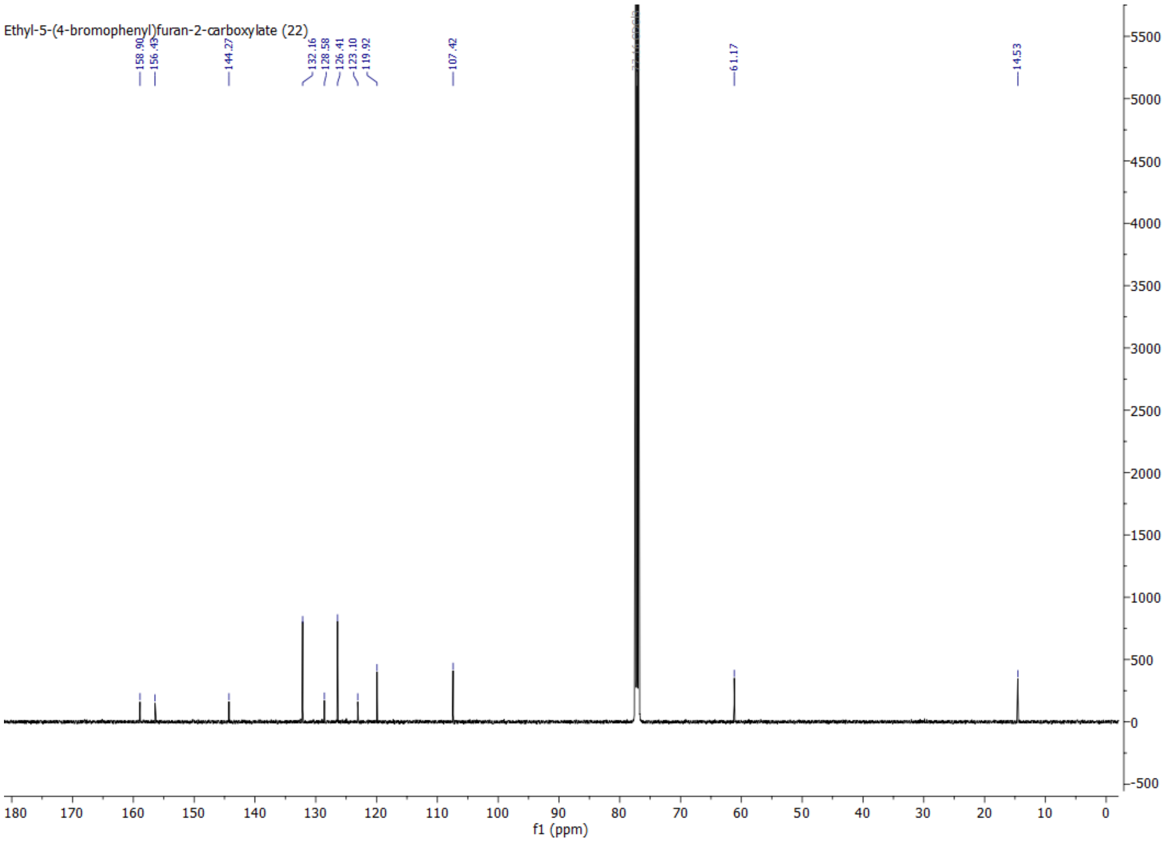

5-(4-Bromophenyl)-*N*-carbamimidoylfuran-2-carboxamide (D15)

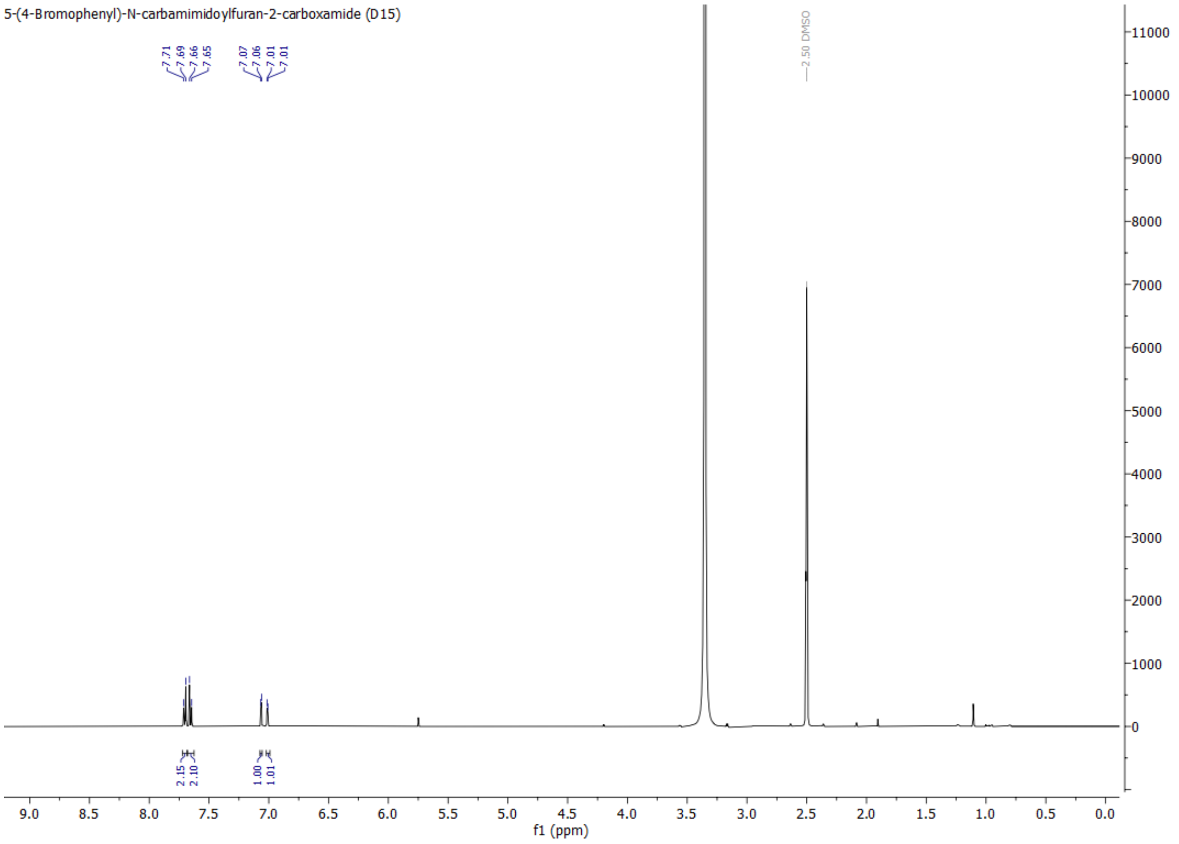

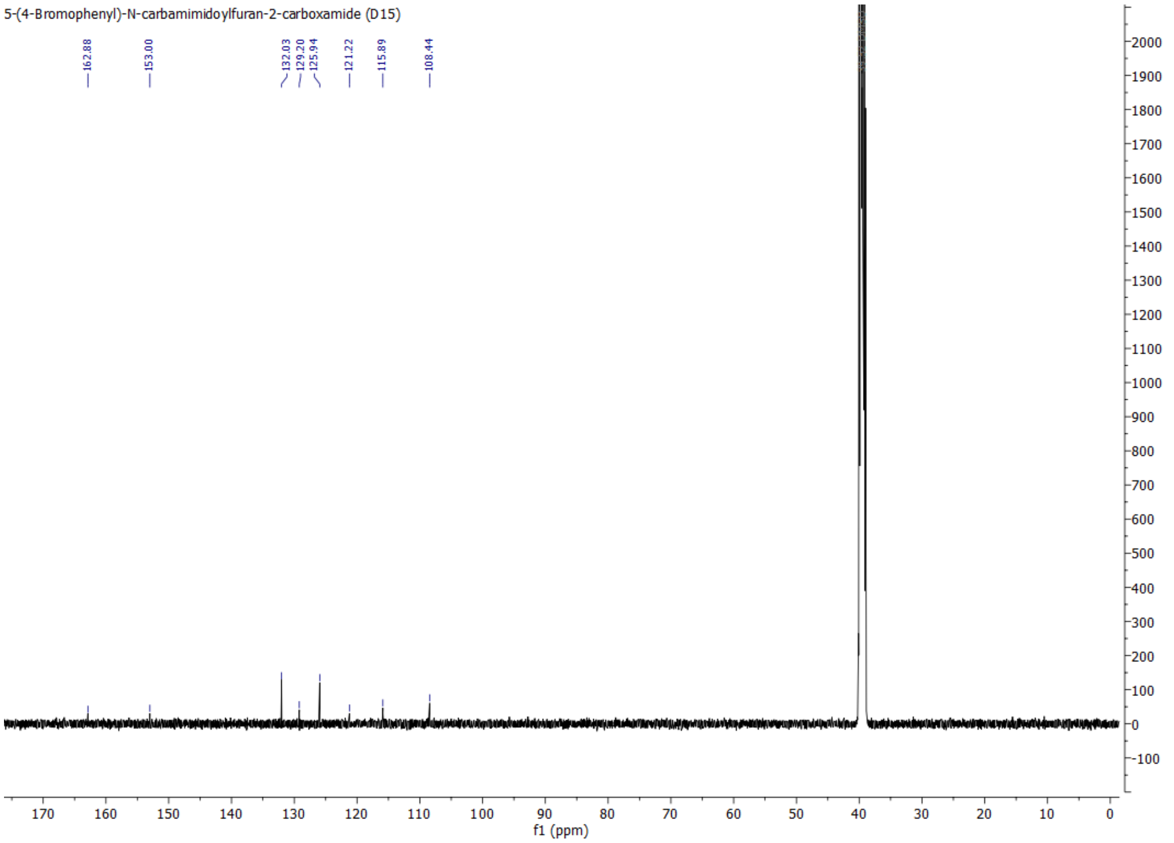

5-(4-Bromophenyl)furan-2-carbaldehyde (23)

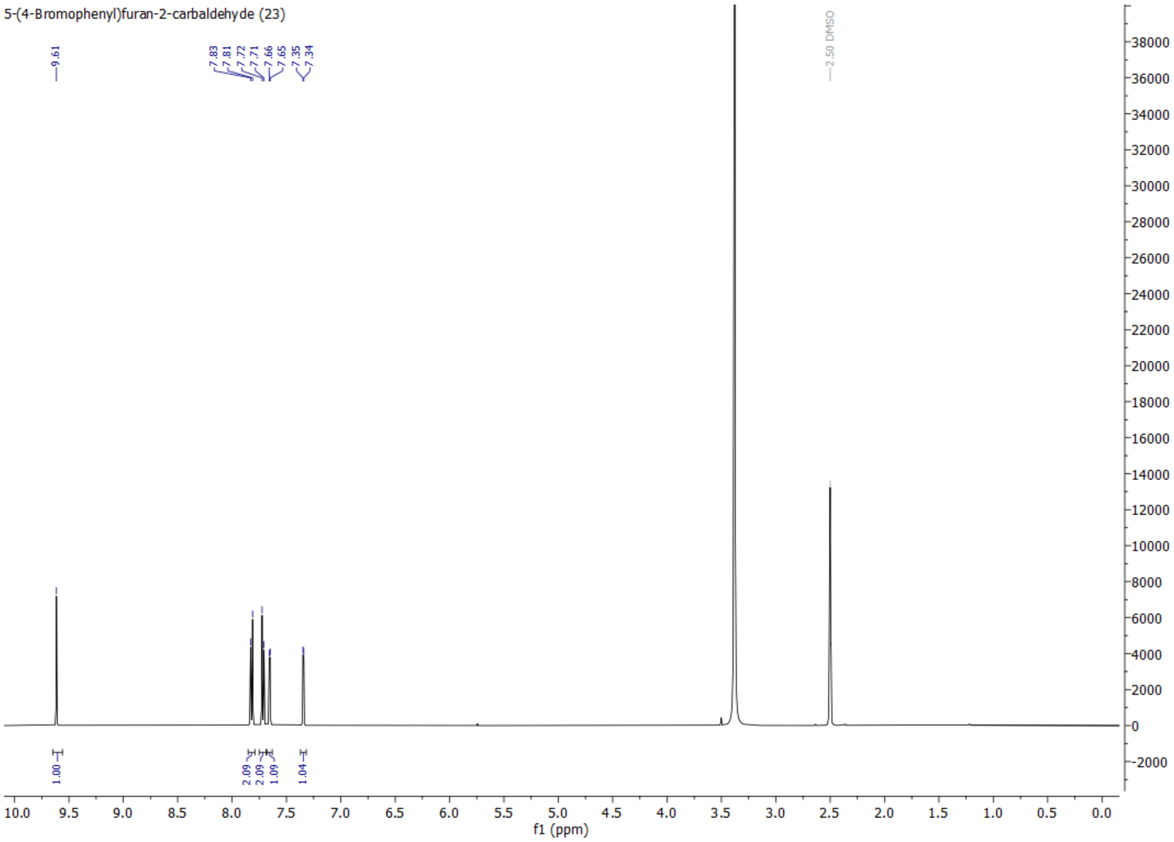

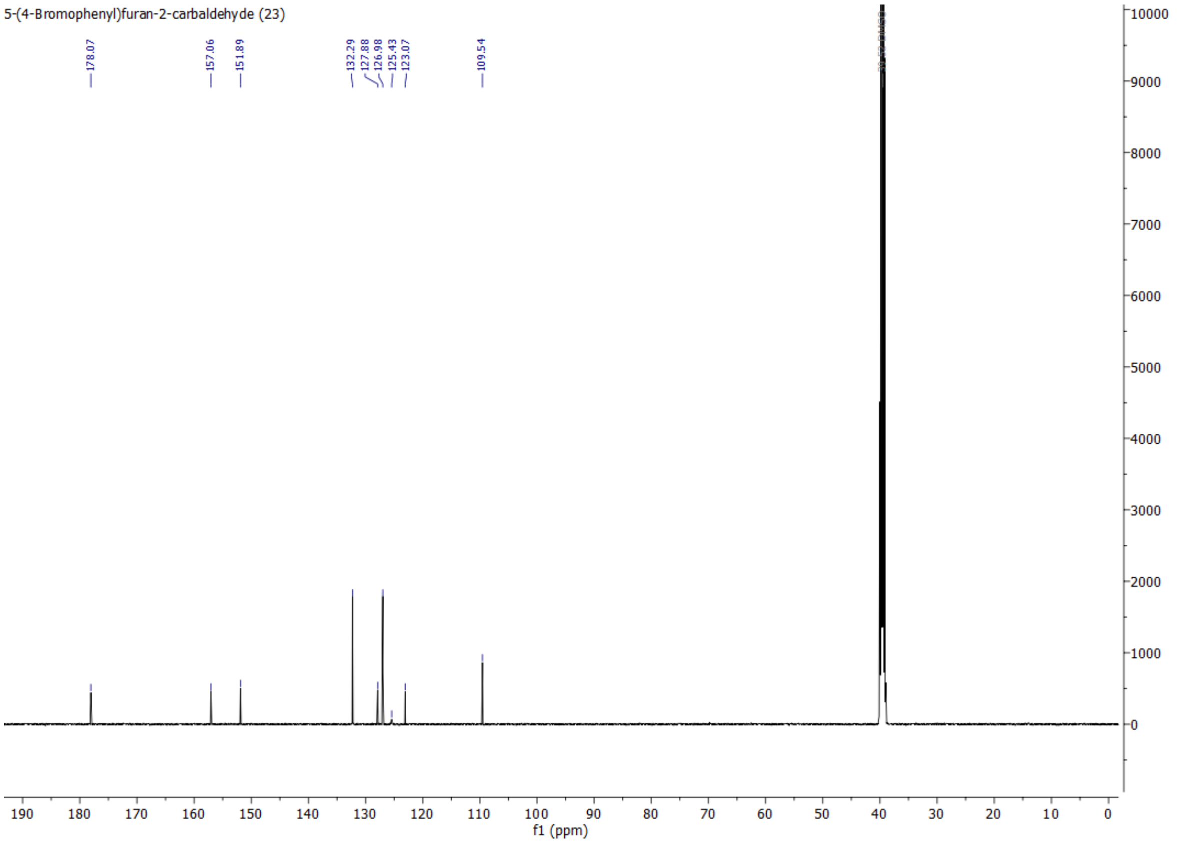

1-((5-(4-Bromophenyl)furan-2-yl)methyl)-1*H*-imidazol-2-aminium 2,2,2-trifluoroacetate (D16)

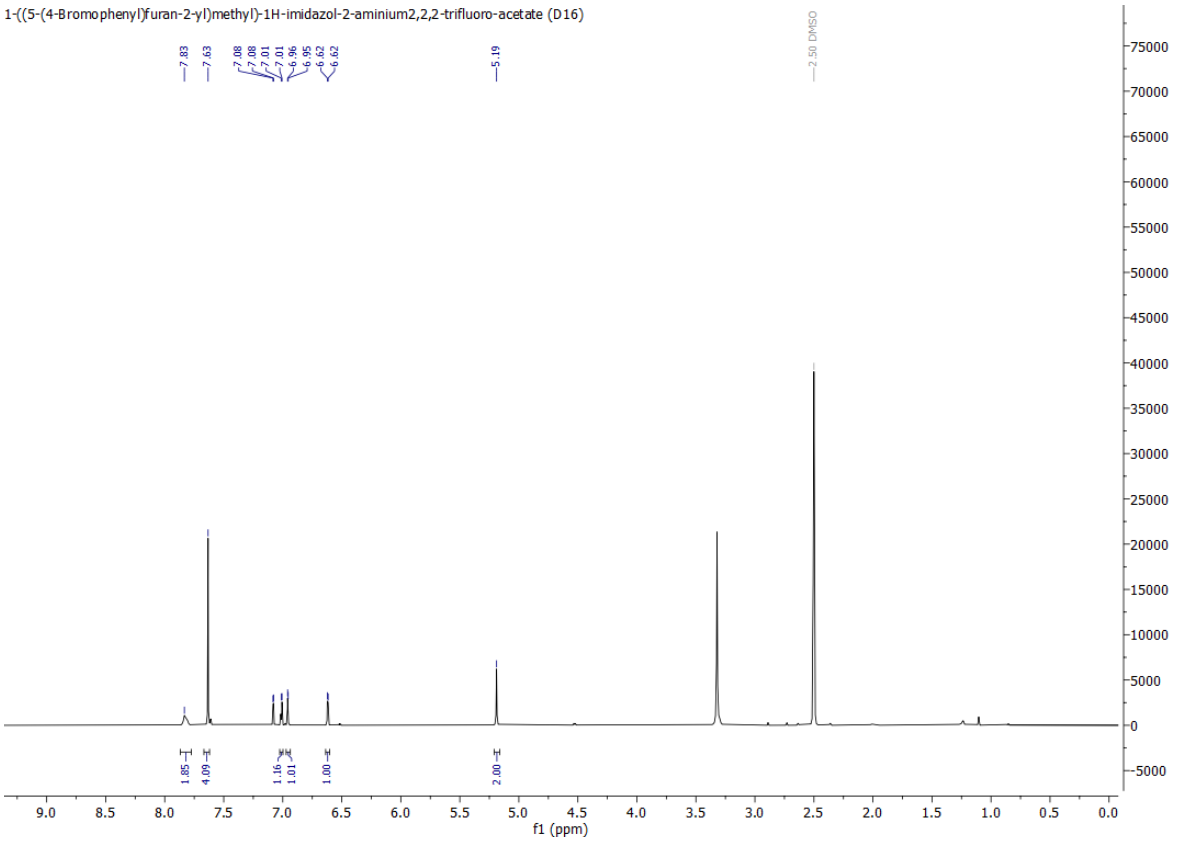

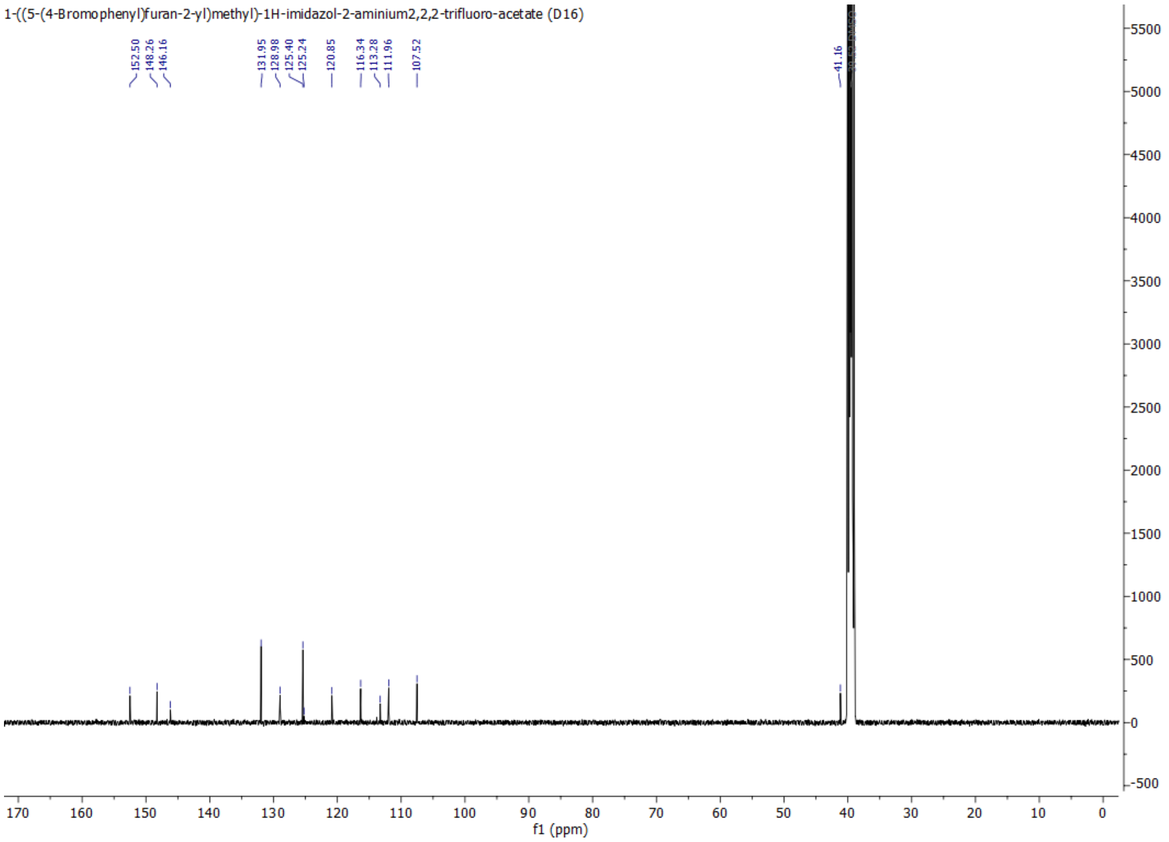
1-((5-(4-Bromophenyl)furan-2-yl)methyl)-1H-imidazol-2-aminium2,2,2-trifluoro-acetate (D16)
152.50
148.26
146.16
131.95
128.98
125.40
125.24
120.85
116.34
113.28
111.96
107.52
41.16
f1 (ppm)

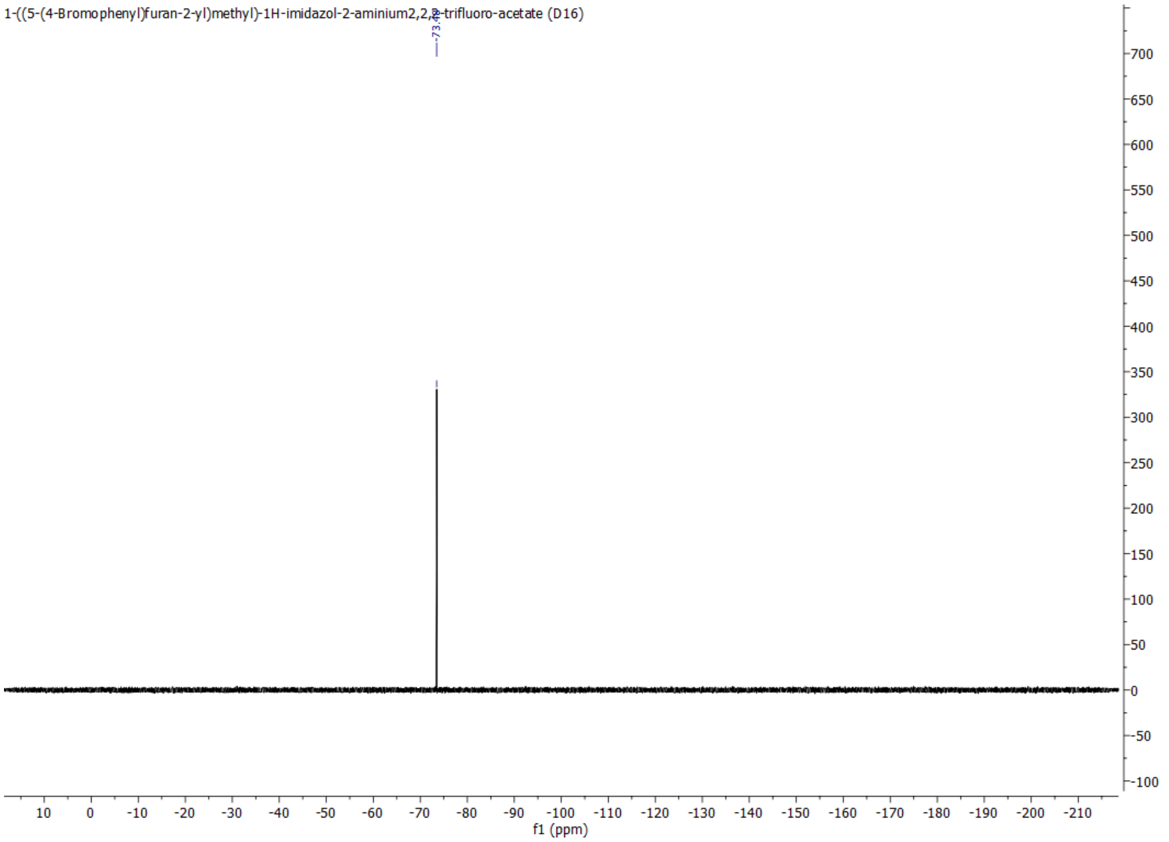
1-((5-(4-Bromophenyl)furan-2-yl)methyl)-1H-imidazol-2-aminium2,2,2-trifluoro-acetate (D16)
f1 (ppm)

Amino((1-(5-(4-bromophenyl)furan-2-yl)ethyl)amino)methaniminium 2,2,2-trifluoro-acetate (D17)

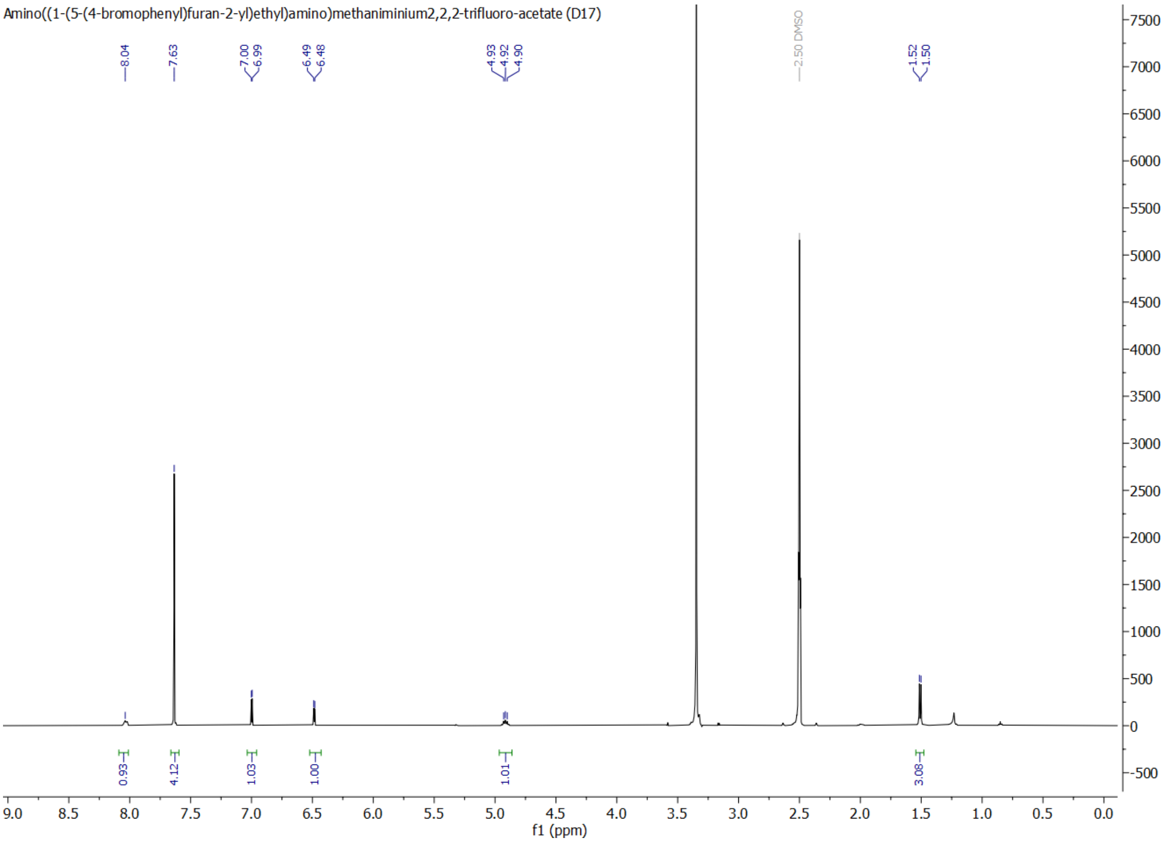

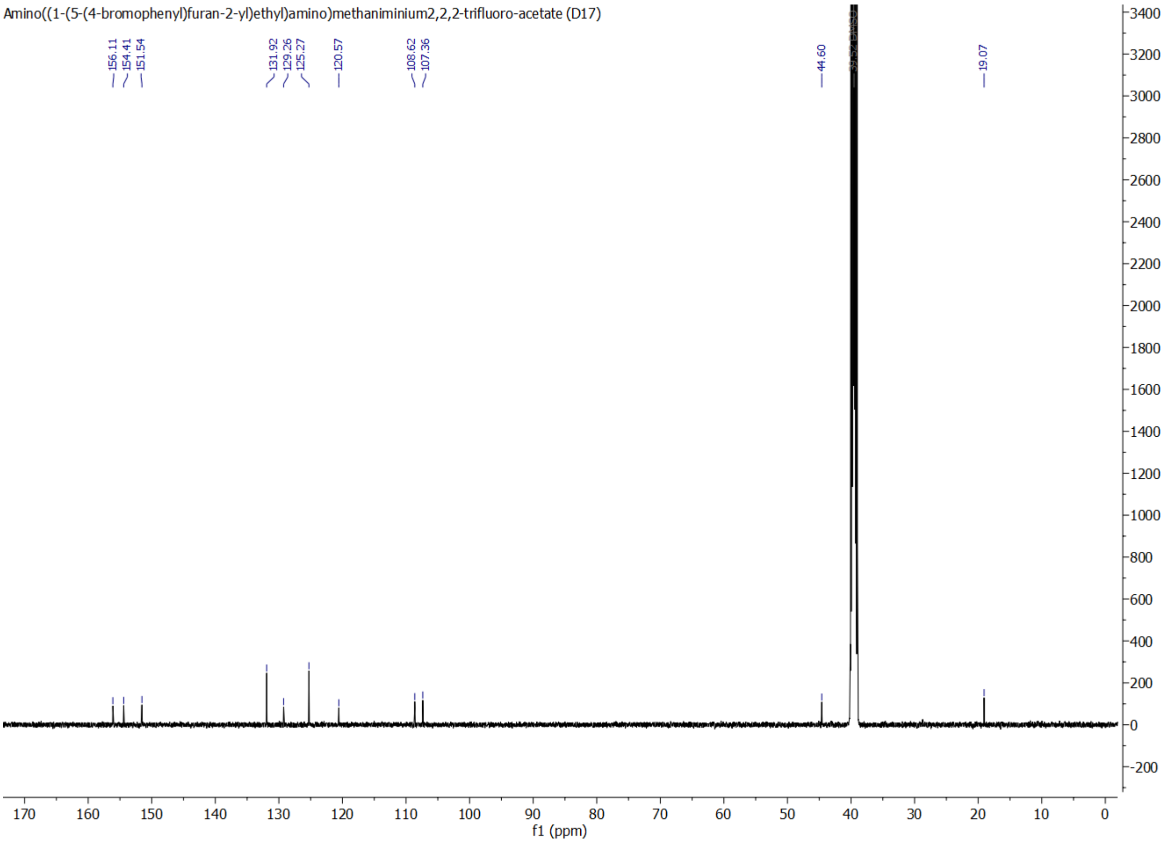

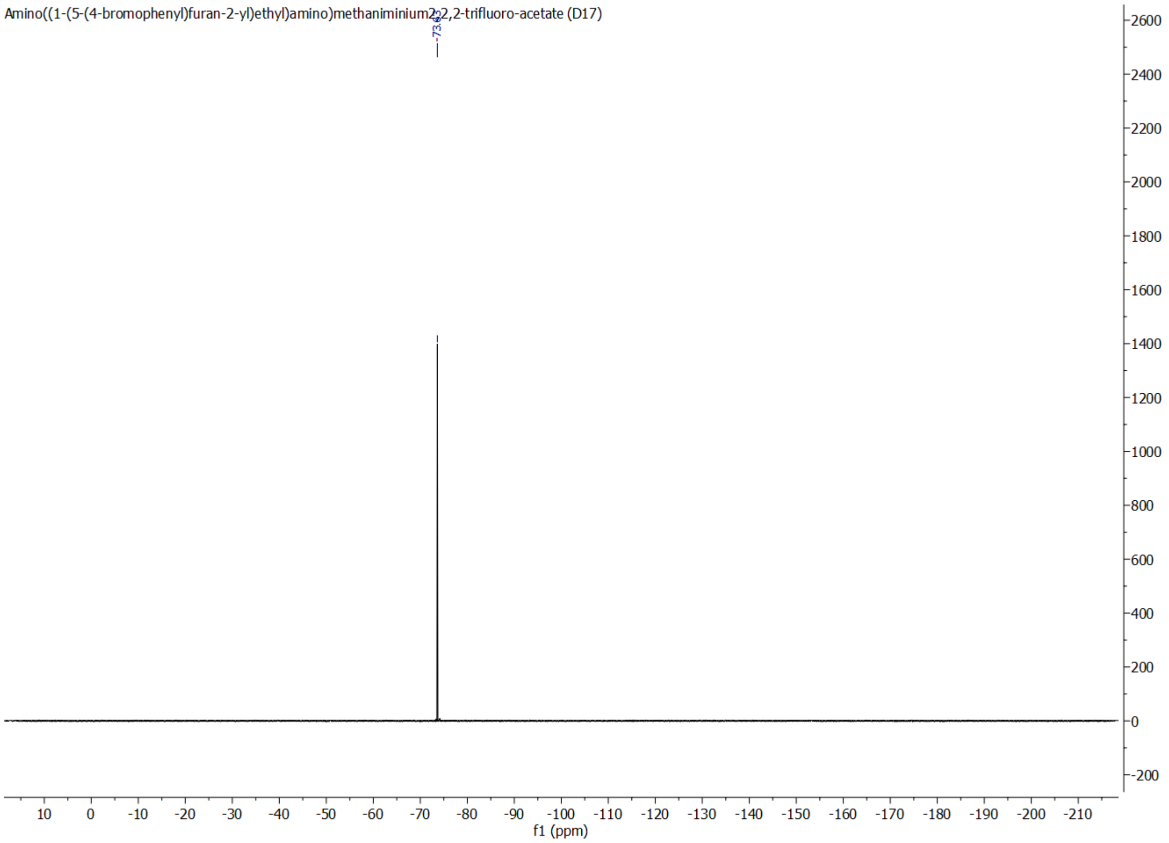

2-(5-(4-Bromophenyl)furan-2-yl)ethan-1-amine (27)

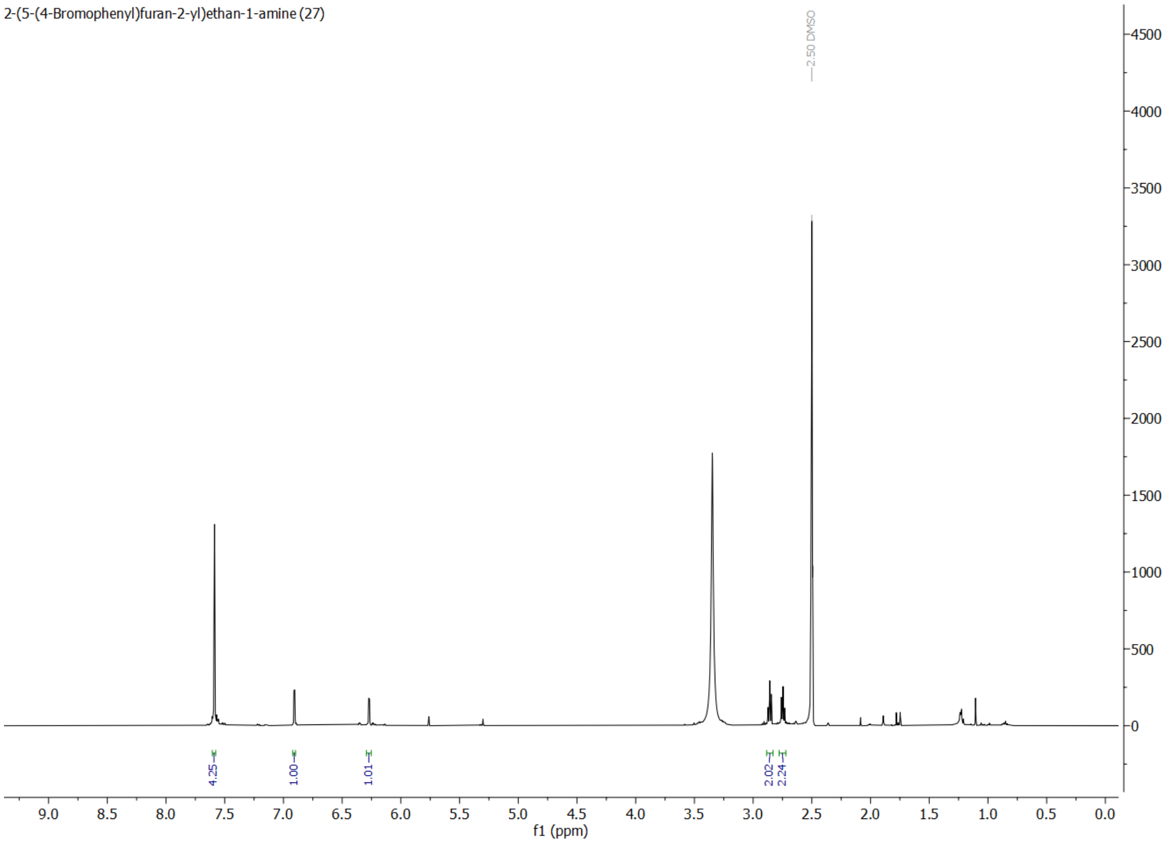

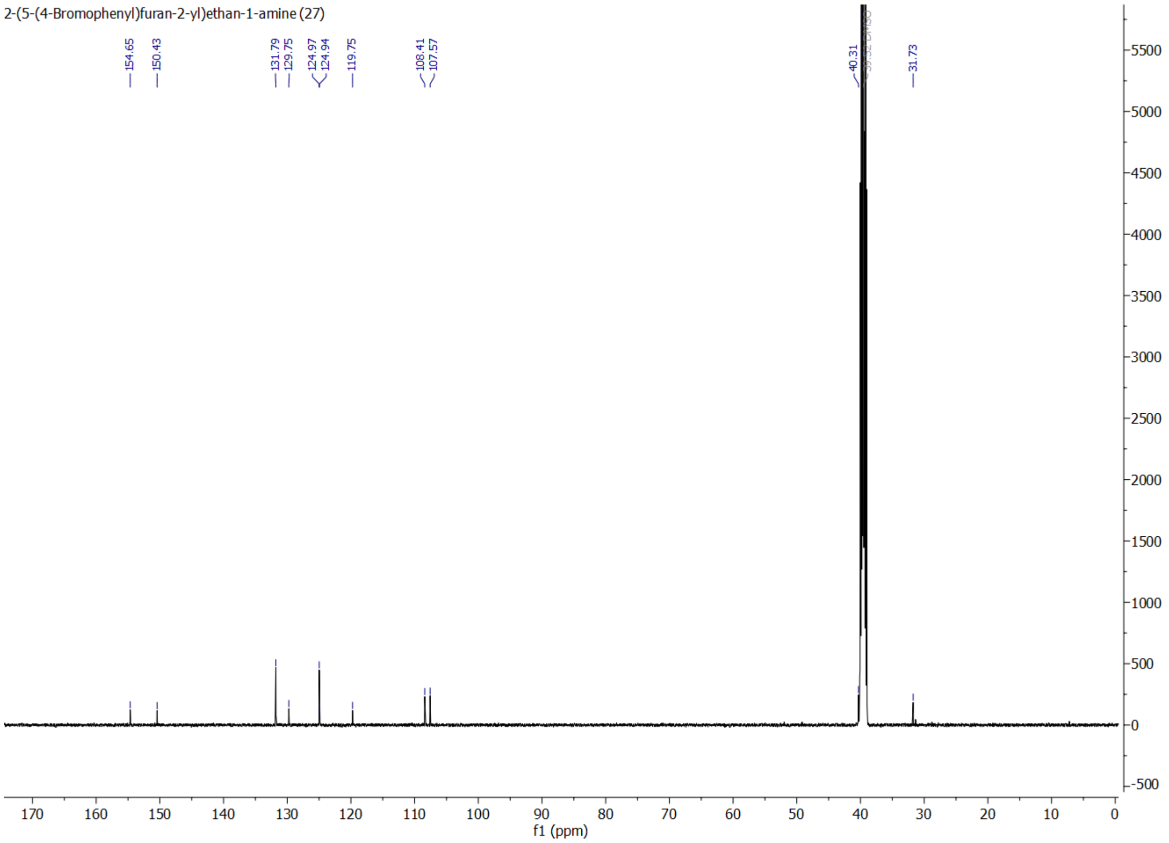

Amino((2-(5-(4-bromophenyl)furan-2-yl)ethyl)amino)methaniminium 2,2,2-trifluoro-acetate (D18)

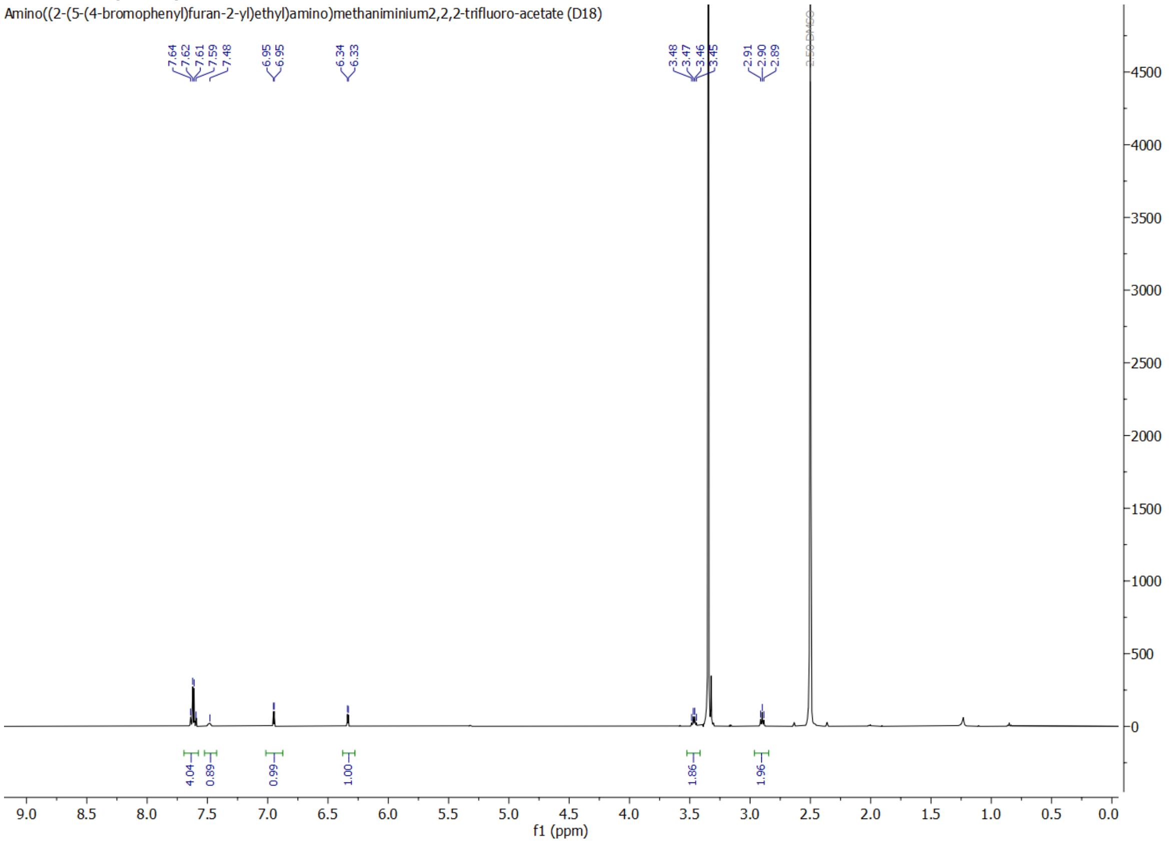

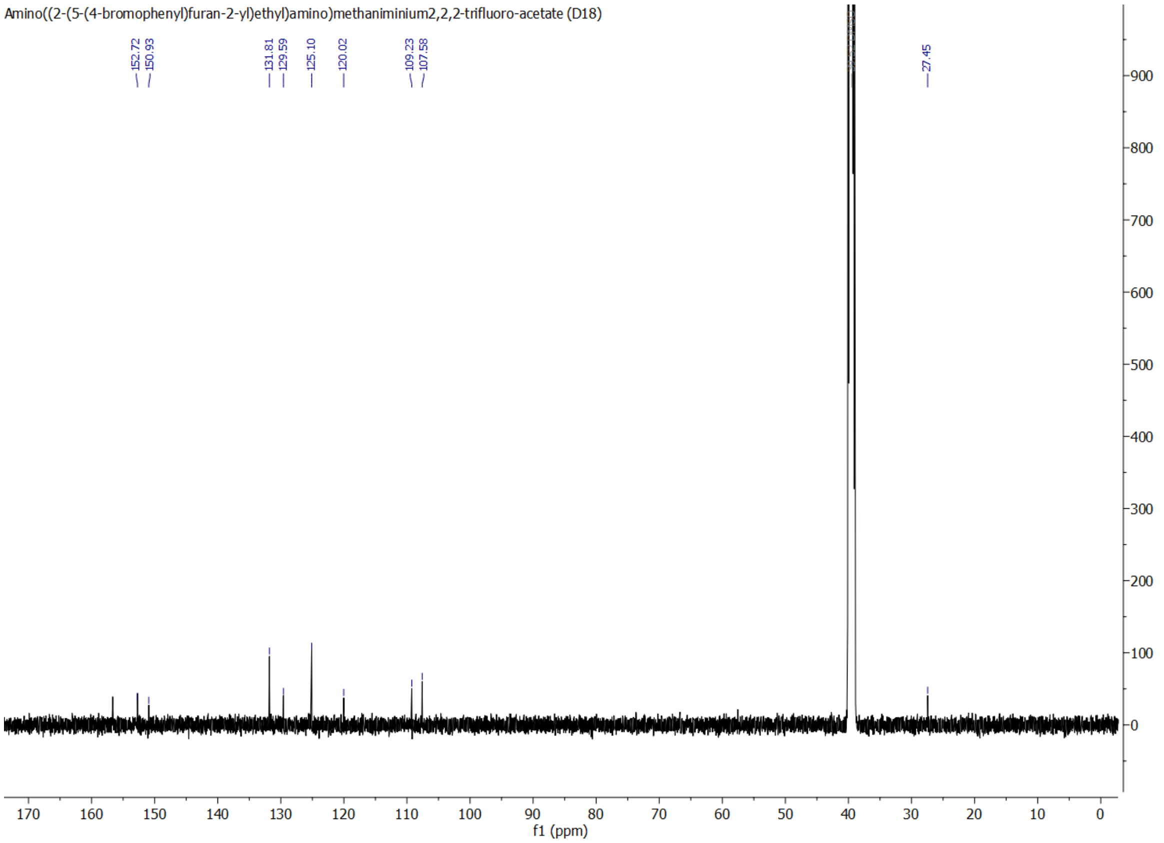
Amino((2-(5-(4-bromophenyl)furan-2-yl)ethyl)amino)methaniminium2,2,2-trifluoro-acetate (D18)
152.72
150.93
131.81
129.59
125.10
120.02
109.23
107.58
27.45
f1 (ppm)

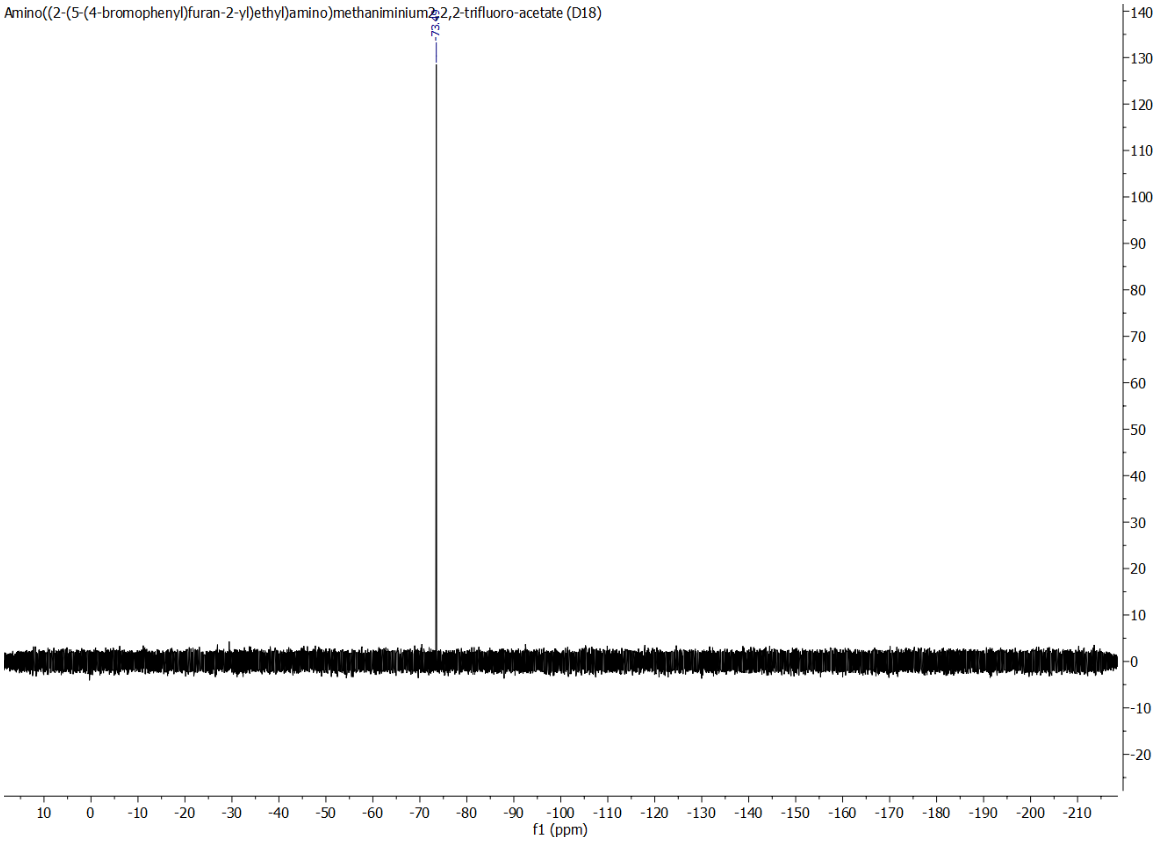
Amino((2-(5-(4-bromophenyl)furan-2-yl)ethyl)amino)methaniminium2,2,2-trifluoro-acetate (D18)
f1 (ppm)

Methyl 4-(5-(aminomethyl)furan-2-yl)benzoate (29)

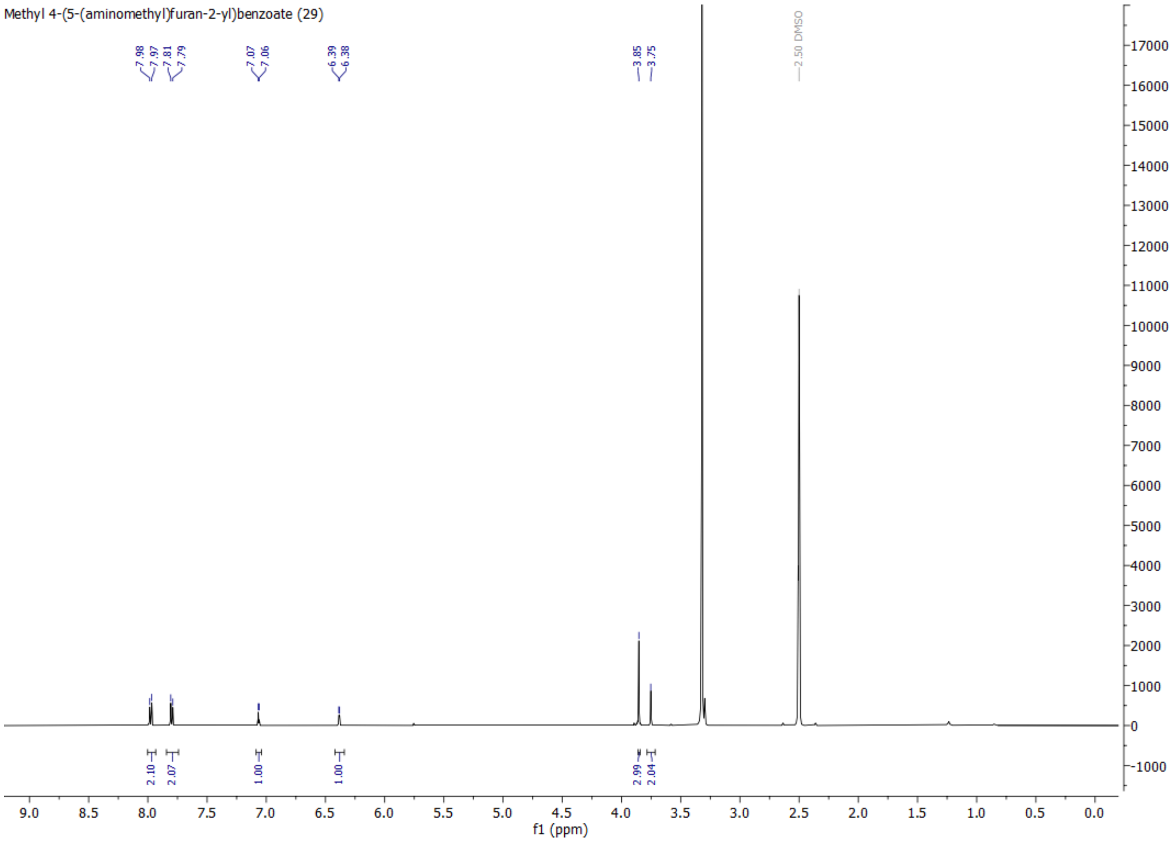

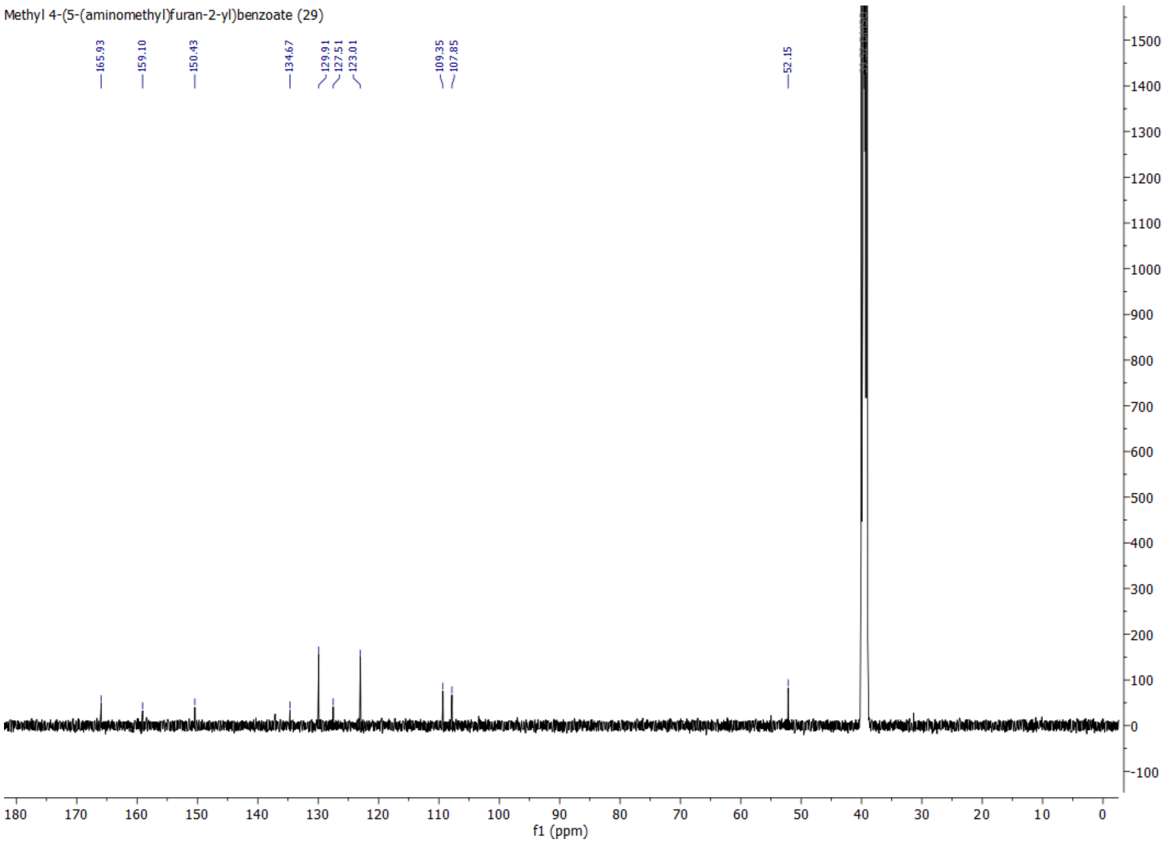

Amino(((5-(4-(methoxycarbonyl)phenyl)furan-2-yl)methyl)amino)methaniminium chloride (30)

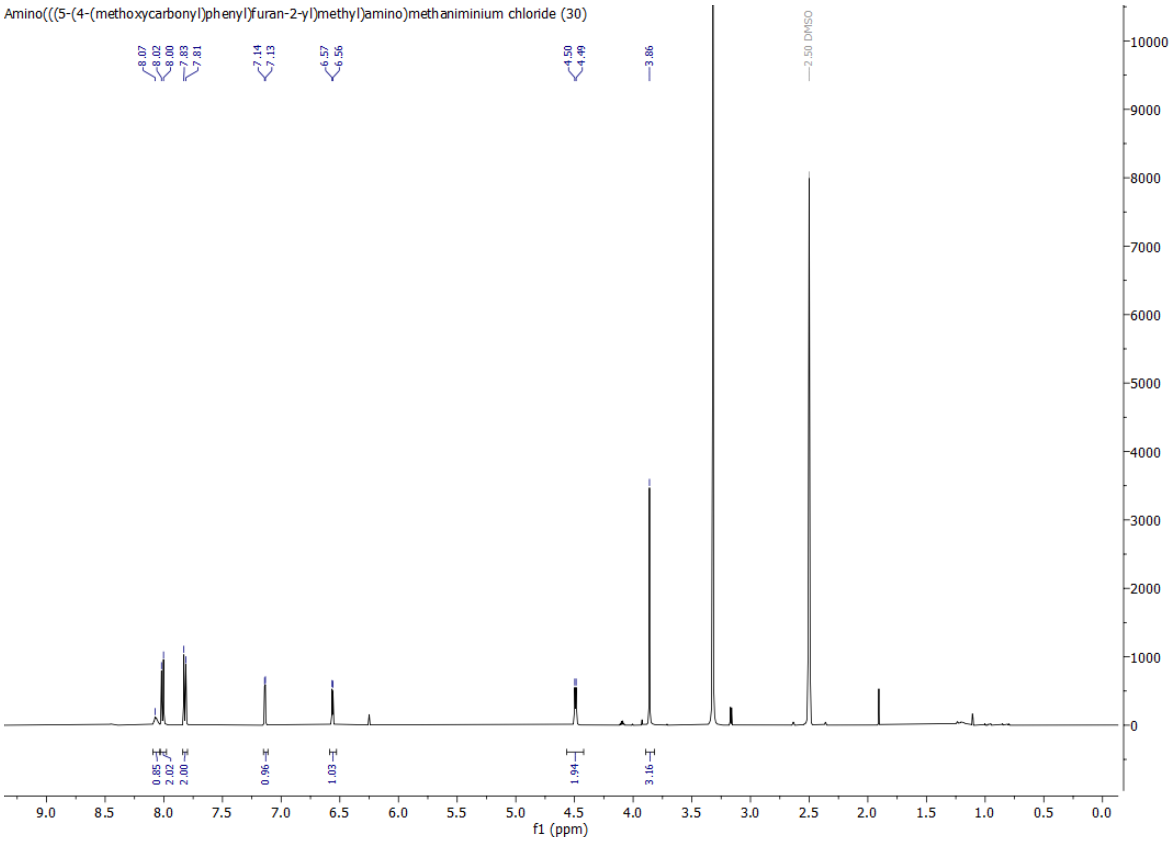

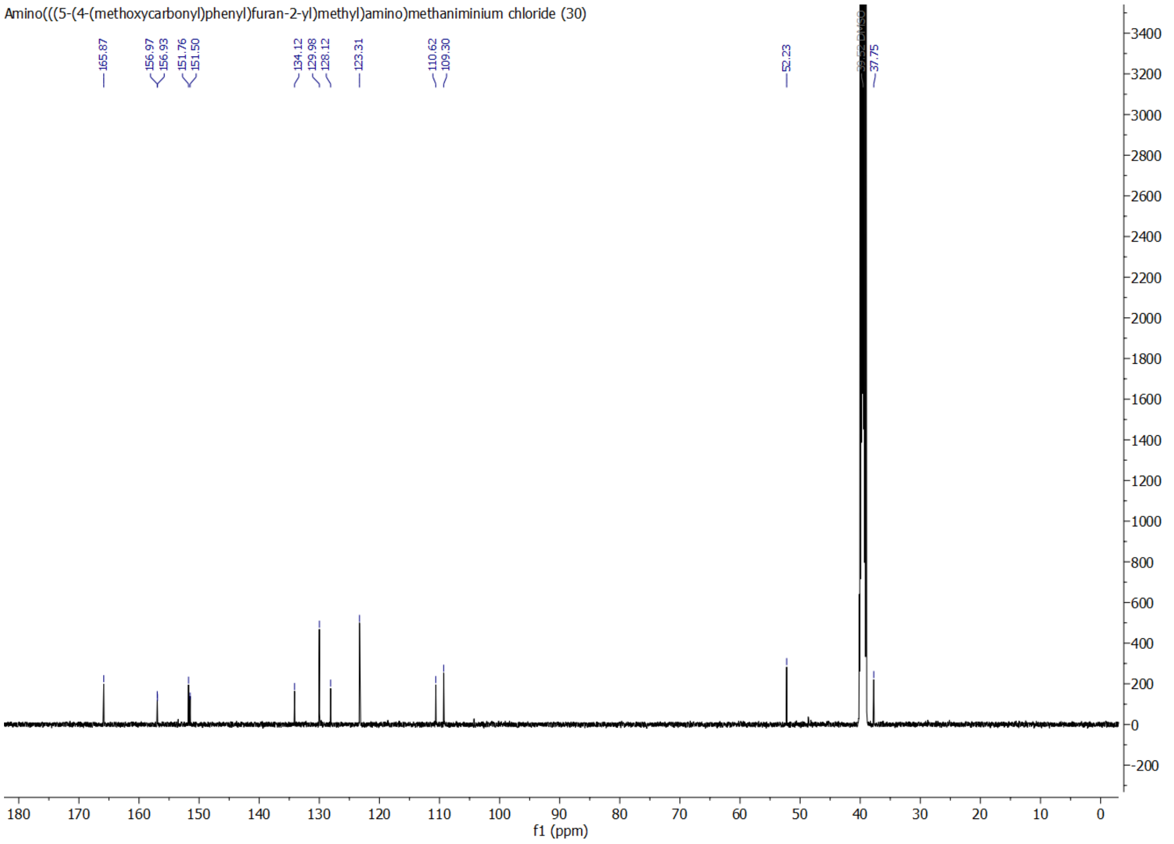

Amino(((5-(4-carboxyphenyl)furan-2-yl)methyl)amino)methaniminium chloride (31)

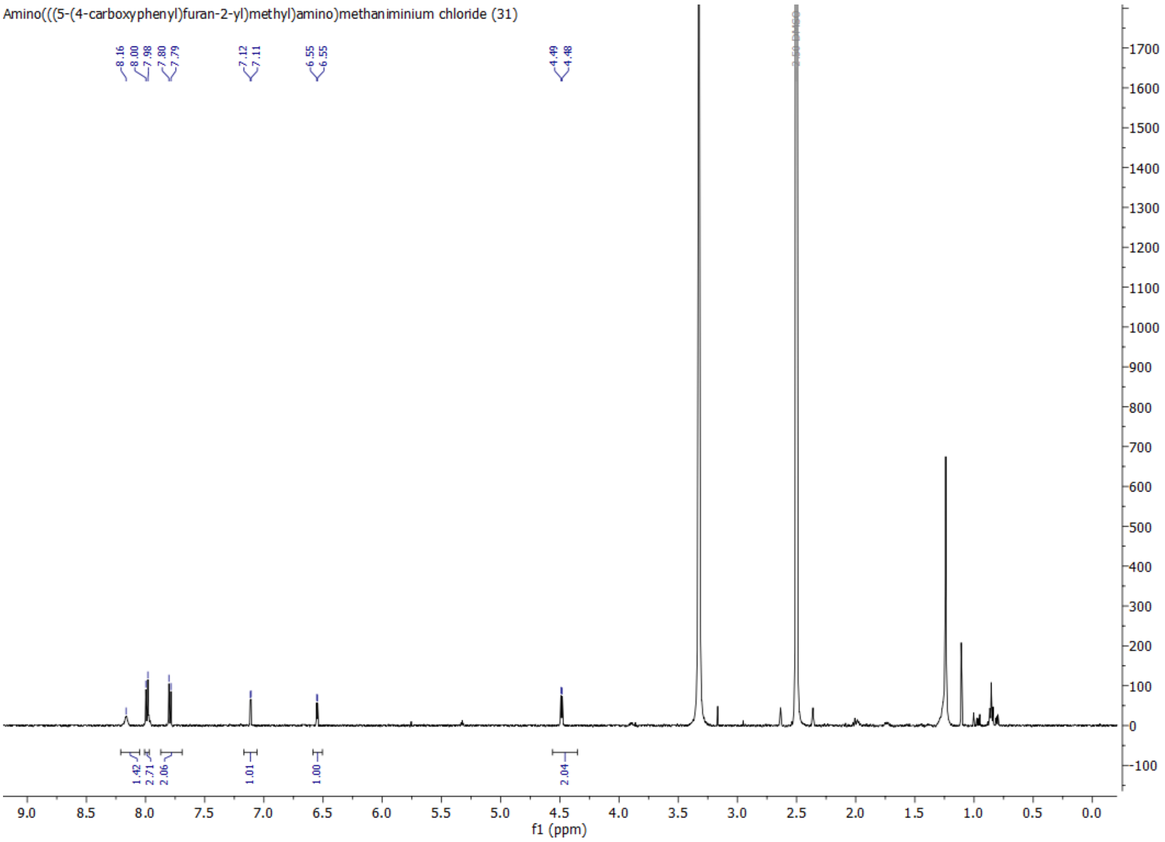

Amino(((5-(4-((2-(3-(but-3-yn-1-yl)-3*H*-diazirin-3-yl)ethoxy)carbonyl)phenyl)furan-2-yl)methyl)amino)methaniminium 2,2,2-trifluoroacetate (L15-P)

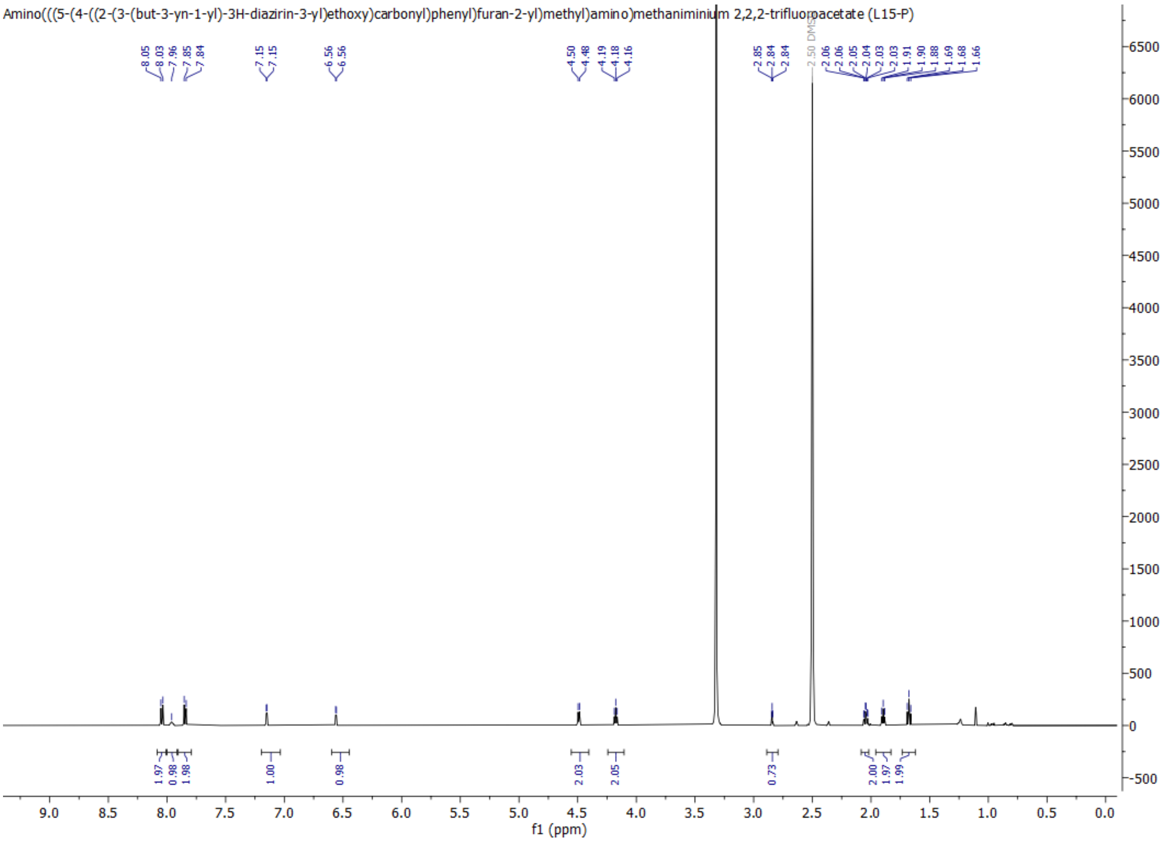

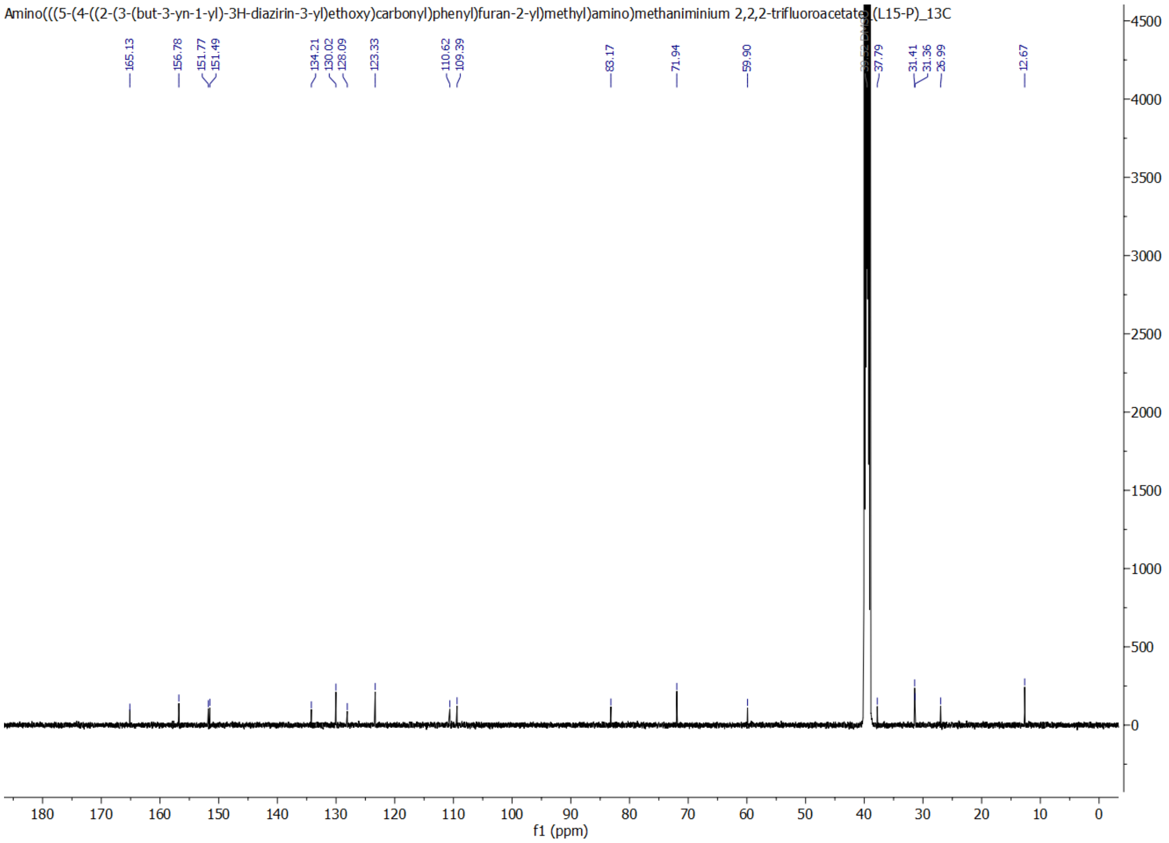
Amino(((5-(4-((2-(3-(but-3-yn-1-yl)-3H-diazirin-3-yl)ethoxy)carbonyl)phenyl)furan-2-yl)methyl)amino)methaniminium 2,2,2-trifluoroacetate (L15-P)_13C
165.13
156.78
151.77
151.49
134.21
130.02
128.09
123.33
110.62
109.39
83.17
71.94
59.90
37.79
31.41
31.36
26.99
12.67
f1 (ppm)

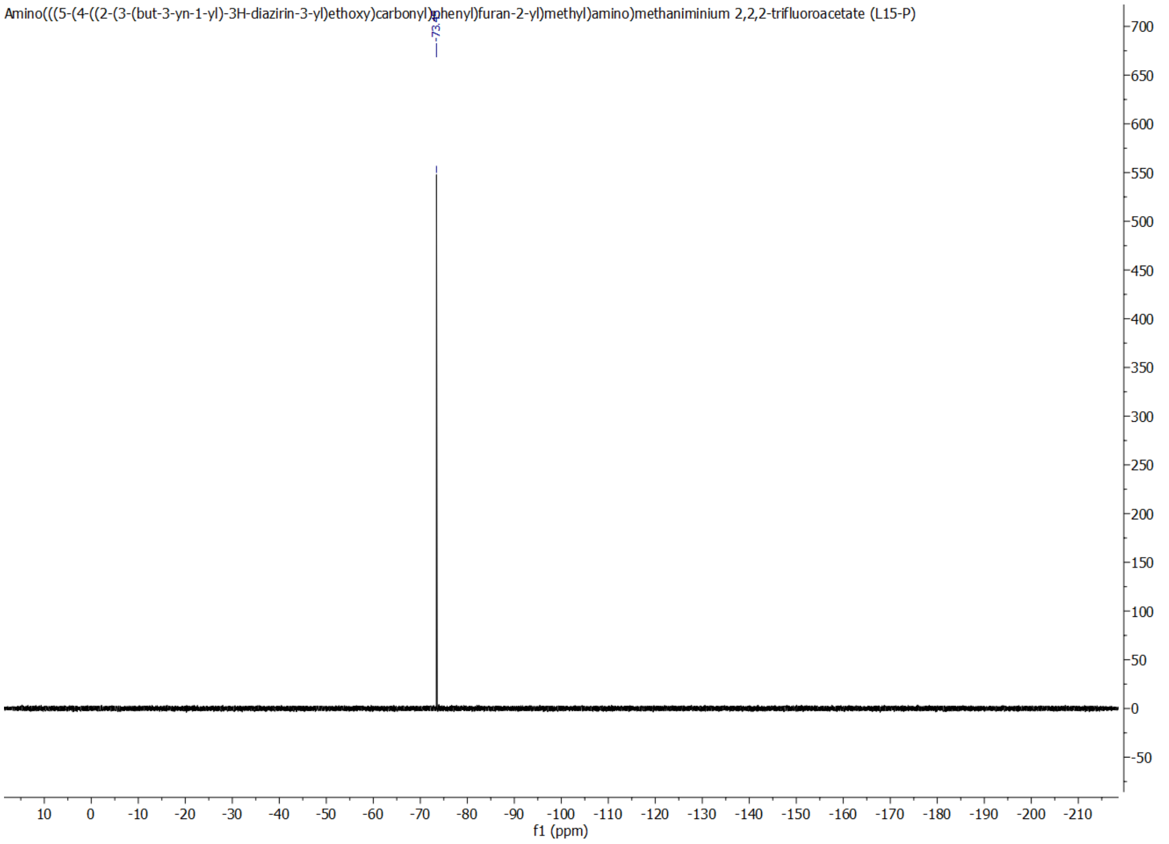
Amino(((5-(4-((2-(3-(but-3-yn-1-yl)-3H-diazirin-3-yl)ethoxy)carbonyl)phenyl)furan-2-yl)methyl)amino)methaniminium 2,2,2-trifluoroacetate (L15-P)
f1 (ppm)

SC-XRD structure report for compound 16.

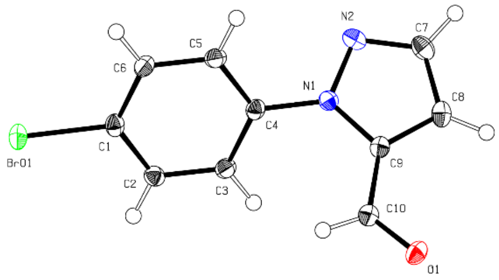

A colorless, rod-shaped crystal of $C_{10}H_7BrN_2O$ coated with perfluorinated ether and fixed on top of a Kapton micro sampler was used for X-ray crystallographic analysis. The X-ray intensity data were collected at 100(2) K on a Bruker D8 VENTURE three-angle diffractometer with a TXS rotating anode with MoK_α radiation (λ=0.71073 Å) using APEX4.[62] The diffractometer was equipped with a Helios optic monochromator, a Bruker PHOTON III detector, and an Oxford Cryostream low temperature device.

A matrix scan was used to determine the initial lattice parameters. All data were integrated with the Bruker SAINT V8.40B software package using a narrow-frame algorithm, and the reflections were corrected for Lorentz and polarisation effects, scan speed, and background.[63] The integration of the data using a monoclinic unit cell yielded 21860 reflections within a 2θ range [°] of 5.33 to 52.87 (0.80 Å), of which 1903 were independent. The data were corrected for absorption effects, including odd and even ordered spherical harmonics by the multi-scan method (SADABS 2016/2).[64] Space group assignment was based upon systematic absences, E statistics, and successful structure refinement.

The structure was solved by direct methods using SHELXT and refined by full-matrix least-squares methods against F^2 by minimizing $\Sigma w(F_o^2 - F_c^2)^2$ using SHELXL in conjunction with SHELXLE.[65-67] All non-hydrogen atoms were refined with anisotropic displacement parameters. Hydrogen atoms were refined isotropically on calculated positions using a riding model with their U_{iso} values constrained to 1.5 times the U_{eq} of their pivot atoms for terminal sp^3 carbon atoms and a C–H distance of 0.98 Å. Non-methyl hydrogen atoms were refined using a riding model with methylene, aromatic, and other C–H distances of 0.99 Å, 0.95 Å, and 1.00 Å, respectively, and U_{iso} values constrained to 1.2 times the U_{eq} of their pivot atoms.

Neutral atom scattering factors for all atoms and anomalous dispersion corrections for the non-hydrogen atoms were taken from International Tables for Crystallography.[68] Supplementary crystallographic data reported in this paper have been deposited with the Cambridge Crystallographic Data Centre (CCDC 2353538) and can be obtained free of charge from The Cambridge Crystallographic Data Centre via www.ccdc.cam.ac.uk/structures.[69] This report and the CIF file were generated using FinalCif.[70]

Table S14: Crystal data and structure refinement for compound 16.

CCDC number	2353538
Empirical formula	$C_{10}H_7BrN_2O$
Formula weight	251.09
Temperature [K]	100(2)
Crystal system	monoclinic
Space group (number)	$P2_1$ (4)
a [Å]	7.5162(15)

b [Å]	4.0623(9)
c [Å]	15.280(3)
α [°]	90
β [°]	90.189(7)
γ [°]	90
Volume [Å^3]	466.55(17)
Z	2
ρ_{calc} [gcm^{-3}]	1.787
μ [mm^{-1}]	4.369
F(000)	248
Crystal size [mm^3]	0.054×0.142×0.473
Crystal colour	colorless
Crystal shape	rod
Radiation	MoK_α (λ=0.71073 Å)
2θ range [°]	5.33 to 52.87 (0.80 Å)
Index ranges	−9 ≤ h ≤ 9<br>−5 ≤ k ≤ 5<br>−19 ≤ l ≤ 19
Reflections collected	21860
Independent reflections	1903<br>R_{int} = 0.0392<br>R_{sigma} = 0.0263
Completeness to θ = 25.242°	99.5 %
Data / Restraints / Parameters	1903 / 1 / 128
Goodness-of-fit on F^2	1.082
Final *R* indexes [*I*≥2σ(*I*)]	R_1 = 0.0159<br>wR_2 = 0.0383
Final *R* indexes [all data]	R_1 = 0.0161<br>wR_2 = 0.0384
Largest peak/hole [eÅ^{-3}]	0.26/−0.28

References

(1) Perez-Riverol, Y.; Bai, J.; Bandla, C.; García-Seisdedos, D.; Hewapathirana, S.; Kamatchinathan, S.; Kundu, Deepti J.; Prakash, A.; Frericks-Zipper, A.; Eisenacher, M.; Walzer, M.; Wang, S.; Brazma, A.; Vizcaíno, J. A. The PRIDE database resources in 2022: a hub for mass spectrometry-based proteomics evidences. *Nucleic Acids Res.* **2021**, *50* (D1), D543-D552.

(2) Li, H. Minimap2: pairwise alignment for nucleotide sequences. *Bioinformatics* **2018**, *34* (18), 3094-3100.

(3) Hadkar, U. B.; Hadkar, A. S. Critical Micelle Concentration of Surfactant Using Hadkar Factor. *Indian J. Pharm. Educ. Res.* **2015**, *49* (2), 134-139.

(4) Dai, S.; Tam, K. C. Isothermal titration calorimetric studies of alkyl phenol ethoxylate surfactants in aqueous solutions. *Colloids Surf.* **2003**, *229* (1-3), 157-168.

(5) Chattopadhyay, A.; Harikumar, K. G. Dependence of critical micelle concentration of a zwitterionic detergent on ionic strength: implications in receptor solubilization. *FEBS Lett* **1996**, *391* (1-2), 199-202.

(6) Qin, X.; Liu, M.; Yang, D.; Zhang, X. Concentration-Dependent Aggregation of CHAPS Investigated by NMR Spectroscopy. *J. Phys. Chem. B* **2010**, *114* (11), 3863-3868.

(7) Chen, S.-Y.; Fiedler, M. K.; Gronauer, T. F.; Omelko, O.; von Wrisberg, M.-K; Wang, T.; Schneider, S.; Sieber, S. A.; Zacharias, M. Unraveling the mechanism of small molecule induced activation of Staphylococcus aureus signal peptidase IB. *Commun. Biol.* **2024**, Accepted Article.

(8) Fey, P. D.; Endres, J. L.; Yajjala, V. K.; Widhelm, T. J.; Boissy, R. J.; Bose, J. L.; Bayles, K. W. A genetic resource for rapid and comprehensive phenotype screening of nonessential Staphylococcus aureus genes. *mBio* **2013**, *4* (1), e00537-00512.

(9) Szklarczyk, D.; Gable, A. L.; Nastou, K. C.; Lyon, D.; Kirsch, R.; Pyysalo, S.; Doncheva, N. T.; Legeay, M.; Fang, T.; Bork, P.; Jensen, L. J.; von Mering, C. The STRING database in 2021: customizable protein-protein networks, and functional characterization of user-uploaded gene/measurement sets. *Nucleic Acids Res.* **2021**, *49* (D1), D605-D612.

(10) Kanehisa, M.; Goto, S. KEGG: kyoto encyclopedia of genes and genomes. *Nucleic Acids Res.* **2000**, *28* (1), 27-30.

(11) Apweiler, R.; Bairoch, A.; Wu, C. H.; Barker, W. C.; Boeckmann, B.; Ferro, S.; Gasteiger, E.; Huang, H.; Lopez, R.; Magrane, M.; Martin, M. J.; Natale, D. A.; O'Donovan, C.; Redaschi, N.; Yeh, L.-S. L.UniProt: the Universal Protein knowledgebase. *Nucleic Acids Res* **2004**, *32* (Database issue), D115-119.

(12) The UniProt Consortium. UniProt: the Universal Protein Knowledgebase in 2023. *Nucleic Acids Res.* **2022**, *51* (D1), D523-D531.

(13) Kong, A. T.; Leprevost, F. V.; Avtonomov, D. M.; Mellacheruvu, D.; Nesvizhskii, A. I. MSFragger: ultrafast and comprehensive peptide identification in mass spectrometry-based proteomics. *Nat. Methods* **2017**, *14* (5), 513-520.

(14) Chang, H. Y.; Kong, A. T.; da Veiga Leprevost, F.; Avtonomov, D. M.; Haynes, S. E.; Nesvizhskii, A. I. Crystal-C: A Computational Tool for Refinement of Open Search Results. *J. Proteome Res.* **2020**, *19* (6), 2511-2515.

(15) Yu, F.; Teo, G. C.; Kong, A. T.; Haynes, S. E.; Avtonomov, D. M.; Geiszler, D. J.; Nesvizhskii, A. I. Identification of modified peptides using localization-aware open search. *Nat. Commun.* **2020**, *11* (1), 4065-4073.

(16) Geiszler, D. J.; Kong, A. T.; Avtonomov, D. M.; Yu, F.; Leprevost, F. D. V.; Nesvizhskii, A. I. PTM-Shepherd: Analysis and Summarization of Post-Translational and Chemical Modifications From Open Search Results. *Mol. Cell Proteomics* **2021**, *20*, 100018-100031.

(17) Teo, G. C.; Polasky, D. A.; Yu, F.; Nesvizhskii, A. I. Fast Deisotoping Algorithm and Its Implementation in the MSFragger Search Engine. *J. Proteome Res.* **2021**, *20* (1), 498-505.

(18) Le, P.; Kunold, E.; Macsics, R.; Rox, K.; Jennings, M. C.; Ugur, I.; Reinecke, M.; Chaves-Moreno, D.; Hackl, M. W.; Fetzer, C.; Mandl, F. A. M.; Lehmann, J.; Korotkov, V. S.; Hacker, S. M.; Kuster, B.; Antes, I.; Pieper, D. H.; Rohde, M.; Wuest, W. M.; Medina, E.; Sieber, S.

A.Repurposing human kinase inhibitors to create an antibiotic active against drug-resistant Staphylococcus aureus, persisters and biofilms. *Nat. Chem.* **2020**, *12* (2), 145-158.
(19) Zanon, P. R. A.; Lewald, L.; Hacker, S. M. Isotopically Labeled Desthiobiotin Azide (isoDTB) Tags Enable Global Profiling of the Bacterial Cysteinome. *Angew. Chem. Int. Ed.* **2020**, *59* (7), 2829-2836.
(20) Kowalska-Krochmal, B.; Dudek-Wicher, R. The Minimum Inhibitory Concentration of Antibiotics: Methods, Interpretation, Clinical Relevance. *Pathogens* **2021**, *10* (2), 165-195.
(21) Te Winkel, J. D.; Gray, D. A.; Seistrup, K. H.; Hamoen, L. W.; Strahl, H. Analysis of Antimicrobial-Triggered Membrane Depolarization Using Voltage Sensitive Dyes. *Front. Cell Dev. Biol.* **2016**, *4*, 29-38.
(22) Smith, P. A.; Romesberg, F. E. Mechanism of action of the arylomycin antibiotics and effects of signal peptidase I inhibition. *Antimicrob. Agents Chemother.* **2012**, *56* (10), 5054-5060.
(23) Kaul, M.; Parhi, A. K.; Zhang, Y.; LaVoie, E. J.; Tuske, S.; Arnold, E.; Kerrigan, J. E.; Pilch, D. S. A bactericidal guanidinomethyl biaryl that alters the dynamics of bacterial FtsZ polymerization. *J. Med. Chem.* **2012**, *55* (22), 10160-10176.
(24) O'Neill, A. J.; Cove, J. H.; Chopra, I. Mutation frequencies for resistance to fusidic acid and rifampicin in Staphylococcus aureus. *J. Antimicrob. Chemother.* **2001**, *47* (5), 647-650.
(25) Bushnell, B.; Rood, J.; Singer, E. BBMerge - Accurate paired shotgun read merging via overlap. *PLoS One* **2017**, *12* (10), e0185056.
(26) Bankevich, A.; Nurk, S.; Antipov, D.; Gurevich, A. A.; Dvorkin, M.; Kulikov, A. S.; Lesin, V. M.; Nikolenko, S. I.; Pham, S.; Prjibelski, A. D.; Pyshkin, A. V.; Sirotkin, A. V.; Vyahhi, N.; Tesler, G.; Alekseyev, M. A.; Pevzner, P. A. SPAdes: a new genome assembly algorithm and its applications to single-cell sequencing. *J. Comput. Biol.* **2012**, *19* (5), 455-477.
(27) Becker, T.; Wiest, A.; Telek, A.; Bejko, D.; Hoffmann-Röder, A.; Kielkowski, P. Transforming Chemical Proteomics Enrichment into a High-Throughput Method Using an SP2E Workflow. *JACS Au* **2022**, *2* (7), 1712-1723.
(28) Coscia, F.; Doll, S.; Bech, J. M.; Schweizer, L.; Mund, A.; Lengyel, E.; Lindebjerg, J.; Madsen, G. I.; Moreira, J. M.; Mann, M. A streamlined mass spectrometry-based proteomics workflow for large-scale FFPE tissue analysis. *J. Pathol.* **2020**, *251* (1), 100-112.
(29) Demichev, V.; Messner, C. B.; Vernardis, S. I.; Lilley, K. S.; Ralser, M. DIA-NN: neural networks and interference correction enable deep proteome coverage in high throughput. *Nat. Methods* **2020**, *17* (1), 41-44.
(30) Tyanova, S.; Temu, T.; Sinitcyn, P.; Carlson, A.; Hein, M. Y.; Geiger, T.; Mann, M.; Cox, J. The Perseus computational platform for comprehensive analysis of (prote)omics data. *Nat. Methods* **2016**, *13* (9), 731-740.
(31) P. R. A. Zanon, F. Y., P. Musacchio, L. Lewald, M. Zollo, K. Krauskopf, D. Mrdović,; P. Raunft, T. E. M., M. Cigler, C. Chang, K. Lang, F. D. Toste, A. I. Nesvizhskii, S.; Hacker, M. Profiling the proteome-wide selectivity of diverse electrophiles. *ChemRxiv* **2021**. Preprint Article DOI: 10.26434/chemrxiv-2021-w7rss-v2.
(32) da Veiga Leprevost, F.; Haynes, S. E.; Avtonomov, D. M.; Chang, H. Y.; Shanmugam, A. K.; Mellacheruvu, D.; Kong, A. T.; Nesvizhskii, A. I. Philosopher: a versatile toolkit for shotgun proteomics data analysis. *Nat. Methods* **2020**, *17* (9), 869-870.
(33) Yu, F.; Haynes, S. E.; Nesvizhskii, A. I. IonQuant Enables Accurate and Sensitive Label-Free Quantification With FDR-Controlled Match-Between-Runs. *Mol. Cell Proteomics* **2021**, *20*, 100077-100089.
(34) Therien, A. G.; Huber, J. L.; Wilson, K. E.; Beaulieu, P.; Caron, A.; Claveau, D.; Deschamps, K.; Donald, R. G.; Galgoci, A. M.; Gallant, M.; Gu, X.; Kevin, N. J.; Lafleur, J.; Leavitt, P. S.; Lebeau-Jacob, C.; Lee, S. S.; Lin, M. M.; Michels, A. A.; Ogawa, A. M.; Painter, R. E.; Parish, C. A.; Park, Y.-W.; Benton-Perdomo, L.; Petcu, M.; Phillips, J. W.; Powels, M. A.; Skorey, K. I.; Tam, J.; Tan, C. M.; Young, K.; Wong, S.; Waddell, S. T.; Miesel, L. Broadening the spectrum of β-lactam antibiotics through inhibition of signal peptidase type I. *Antimicrob. Agents Chemother.* **2012**, *56* (9), 4662-4670.
(35) Rao, S.; Bockstael, K.; Nath, S.; Engelborghs, Y.; Anné, J.; Geukens, N. Enzymatic investigation of the Staphylococcus aureus type I signal peptidase SpsB - implications for the search for novel antibiotics. *FEBS J.* **2009**, *276* (12), 3222-3234.

(36) Trott, O.; Olson, A. J. AutoDock Vina: improving the speed and accuracy of docking with a new scoring function, efficient optimization, and multithreading. *J. Comput. Chem.* **2010**, *31* (2), 455-461.
(37) Lee, J.; Cheng, X.; Swails, J. M.; Yeom, M. S.; Eastman, P. K.; Lemkul, J. A.; Wei, S.; Buckner, J.; Jeong, J. C.; Qi, Y.; Jo, S.; Pande, V. S.; Case, D. A.; Brooks, C. L. III; MacKerell, A. D. Jr.; Klauda, J. B.; Im, W. CHARMM-GUI Input Generator for NAMD, GROMACS, AMBER, OpenMM, and CHARMM/OpenMM Simulations Using the CHARMM36 Additive Force Field. *J. Chem. Theory Comput.* **2016**, *12* (1), 405-413.
(38) Tian, C.; Kasavajhala, K.; Belfon, K. A. A.; Raguette, L.; Huang, H.; Migues, A. N.; Bickel, J.; Wang, Y.; Pincay, J.; Wu, Q.; Simmerling, C. ff19SB: Amino-Acid-Specific Protein Backbone Parameters Trained against Quantum Mechanics Energy Surfaces in Solution. *J. Chem. Theory Comput.* **2020**, *16* (1), 528-552.
(39) Dickson, C. J.; Walker, R. C.; Gould, I. R. Lipid21: Complex Lipid Membrane Simulations with AMBER. *J. Chem. Theory Comput.* **2022**, *18* (3), 1726-1736.
(40) Wang, J.; Wolf, R. M.; Caldwell, J. W.; Kollman, P. A.; Case, D. A. Development and testing of a general amber force field. *J. Comput. Chem.* **2004**, *25* (9), 1157-1174.
(41) Izadi, S.; Anandakrishnan, R.; Onufriev, A. V. Building Water Models: A Different Approach. *J. Phys. Chem. Lett.* **2014**, *5* (21), 3863-3871.
(42) Jakalian, A.; Jack, D. B.; Bayly, C. I. Fast, efficient generation of high-quality atomic charges. AM1-BCC model: II. Parameterization and validation. *J. Comput. Chem.* **2002**, *23* (16), 1623-1641.
(43) Wang, J.; Wang, W.; Kollman, P. A.; Case, D. A. Automatic atom type and bond type perception in molecular mechanical calculations. *J Mol Graph Model* **2006**, *25* (2), 247-260.
(44) D.A. Case, H. M. A., K. Belfon, I.Y. Ben-Shalom, J.T. Berryman, S.R. Brozell, D.S. Cerutti, T.E. Cheatham, III, G.A. Cisneros, V.W.D. Cruzeiro, T.A. Darden, R.E. Duke, G. Giambasu, M.K. Gilson, H. Gohlke, A.W. Goetz, R. Harris, S. Izadi, S.A. Izmailov, K. Kasavajhala, M.C. Kaymak, E. King, A. Kovalenko, T. Kurtzman, T.S. Lee, S. LeGrand, P. Li, C. Lin, J. Liu, T. Luchko, R. Luo, M. Machado, V. Man, M. Manathunga, K.M. Merz, Y. Miao, O. Mikhailovskii, G. Monard, H. Nguyen, K.A. O'Hearn, A. Onufriev, F. Pan, S. Pantano, R. Qi, A. Rahnamoun, D.R. Roe, A. Roitberg, C. Sagui, S. Schott-Verdugo, A. Shajan, J. Shen, C.L. Simmerling, N.R. Skrynnikov, J. Smith, J. Swails, R.C. Walker, J. Wang, J. Wang, H. Wei, R.M. Wolf, X. Wu, Y. Xiong, Y. Xue, D.M. York, S. Zhao, and P.A. Kollman. Amber 2022, University of California, San Francisco. 2022.
(45) Goga, N.; Rzepiela, A. J.; de Vries, A. H.; Marrink, S. J.; Berendsen, H. J. C. Efficient Algorithms for Langevin and DPD Dynamics. *J. Chem. Theory Comput.* **2012**, *8* (10), 3637-3649.
(46) Berendsen, H. J. C.; Postma, J. P. M.; van Gunsteren, W. F.; DiNola, A.; Haak, J. R. Molecular dynamics with coupling to an external bath. *J. Chem. Phys.* **1984**, *81* (8), 3684-3690.
(47) Andersen, H. C. Rattle: A "velocity" version of the shake algorithm for molecular dynamics calculations. *J. Comput. Phys.* **1983**, *52* (1), 24-34.
(48) Balusek, C.; Hwang, H.; Lau, C. H.; Lundquist, K.; Hazel, A.; Pavlova, A.; Lynch, D. L.; Reggio, P. H.; Wang, Y.; Gumbart, J. C. Accelerating Membrane Simulations with Hydrogen Mass Repartitioning. *J. Chem. Theory Comput.* **2019**, *15* (8), 4673-4686.
(49) Roe, D. R.; Cheatham, T. E., III. PTRAJ and CPPTRAJ: Software for Processing and Analysis of Molecular Dynamics Trajectory Data. *J. Chem. Theory Comput.* **2013**, *9* (7), 3084-3095.
(50) Fulmer, G. R.; Miller, A. J. M.; Sherden, N. H.; Gottlieb, H. E.; Nudelman, A.; Stoltz, B. M.; Bercaw, J. E.; Goldberg, K. I. NMR Chemical Shifts of Trace Impurities: Common Laboratory Solvents, Organics, and Gases in Deuterated Solvents Relevant to the Organometallic Chemist. *Organometallics* **2010**, *29* (9), 2176-2179.
(51) Fürst, M. C. D.; Sauer, C. S.; Moriyama, T.; Kamimura, A.; Heinrich, M. R. Synthesis of 6-Arylpyridin-3-ols by Oxidative Rearrangement of (5-Arylfurfuryl)amines. *Eur. J. Org. Chem.* **2016**, *2016* (18), 3051-3055.
(52) An, T.; Kang, B.; Kang, S.; Pac, J.; Youk, J.; Lin, D.; Lee, Y. Guanidine cyclic diimides and their polymers. *Chem. Commun.* **2019**, *55* (69), 10222-10225.

(53) Fürst, M. C. D.; Gans, E.; Böck, M. J.; Heinrich, M. R. Visible-Light-Induced, Catalyst-Free Radical Arylations of Arenes and Heteroarenes with Aryldiazonium Salts. *Chemistry* **2017**, *23* (61), 15312-15315.
(54) Fehler, S. K.; Maschauer, S.; Höfling, S. B.; Bartuschat, A. L.; Tschammer, N.; Hübner, H.; Gmeiner, P.; Prante, O.; Heinrich, M. R. Fast and Efficient 18F-Labeling by [18F]Fluorophenylazocarboxylic Esters. *Chem. Eur. J.* **2014**, *20* (2), 370-375..
(55) Yano, J. K.; Denton, T. T.; Cerny, M. A.; Zhang, X.; Johnson, E. F.; Cashman, J. R. Synthetic Inhibitors of Cytochrome P-450 2A6: Inhibitory Activity, Difference Spectra, Mechanism of Inhibition, and Protein Cocrystallization. *J. Med. Chem.* **2006**, *49* (24), 6987-7001.
(56) Krake, S. H.; Martinez, P. D. G.; McLaren, J.; Ryan, E.; Chen, G.; White, K.; Charman, S. A.; Campbell, S.; Willis, P.; Dias, L. C. Novel inhibitors of Plasmodium falciparum based on 2,5-disubstituted furans. *Eur. J. Med. Chem.* **2017**, *126*, 929-936.
(57) Ajdačić, V.; Senerovic, L.; Vranić, M.; Pekmezovic, M.; Arsic-Arsnijevic, V.; Veselinovic, A.; Veselinovic, J.; Šolaja, B. A.; Nikodinovic-Runic, J.; Opsenica, I. M. Synthesis and evaluation of thiophene-based guanylhydrazones (iminoguanidines) efficient against panel of voriconazole-resistant fungal isolates. *Bioorg. Med. Chem.* **2016**, *24* (6), 1277-1291.
(58) Zeng, R.; Li, Q.; Li, Z.; Li, X.; Xie, C.; Su, X.; Tang, D. Benzo[e]indolium derivatives in aqueous solutions: Reaction with bisulfite and successive interaction with Cu2+ and Hg2+. *Spectrochimica Acta Part A: Molecular and Biomolecular Spectroscopy* **2018**, *202*, 324-332.
(59) Zhu, Y.; Xu, P.; Gong, Y. Triflic Acid-Catalyzed Cycloisomerization Reactions of Donor-Acceptor Cyclopropanes: Access to Alkyl 5-Arylfuran-2-carboxylates. *J. Org. Chem.* **2016**, *81* (11), 4829-4834.
(60) Lee, S.; Yi, K. Y.; Hwang, S. K.; Lee, B. H.; Yoo, S.-e.; Lee, K. (5-Arylfuran-2-ylcarbonyl)guanidines as Cardioprotectives through the Inhibition of Na+/H+ Exchanger Isoform-1. *J. Med. Chem.* **2005**, *48* (8), 2882-2891.
(61) Hashmi, A. S. K.; Wölfle, M.; Ata, F.; Hamzic, M.; Salathé, R.; Frey, W. Gold Catalysis: Dihydroisobenzofurans and Isochromanes by the Intramolecular Furan/Alkyne Reaction. *Adv. Synth. Catal.* **2006**, *348* (16-17), 2501-2508.
(62) APEX4 Suite of Crystallographic Software, V.-., Bruker AXS Inc., Madison, Wisconsin, USA, 2021.
(63) Bruker. *SAINT*, V8.40B, Bruker AXS Inc.,. Madison, Wisconsin, USA, 2021.
(64) Krause, L.; Herbst-Irmer, R.; Sheldrick, G. M.; Stalke, D. Comparison of silver and molybdenum microfocus X-ray sources for single-crystal structure determination. *J. Appl. Crystallogr.* **2015**, *48* (1), 3-10.
(65) Sheldrick, G. SHELXT - Integrated space-group and crystal-structure determination. *Acta Crystallogr. A* **2015**, *71* (1), 3-8.
(66) Sheldrick, G. Crystal structure refinement with SHELXL. *Acta Crystallogr. C* **2015**, *71* (1), 3-8.
(67) Hubschle, C. B.; Sheldrick, G. M.; Dittrich, B. ShelXle: a Qt graphical user interface for SHELXL. *J. Appl. Crystallogr.* **2011**, *44* (6), 1281-1284.
(68) Prince, E. E. International Union of Crystallography. 193–199, Ed.; Chester, England.
(69) Groom, C. R.; Bruno, I. J.; Lightfoot, M. P.; Ward, S. C. The Cambridge Structural Database. *Acta Crystallogr. B* **2016**, *72* (2), 171-179.
(70) Kratzert, D. *FinalCif*, V125, https://dkratzert.de/finalcif.html.
